# Hydroelementation and Phosphinidene Transfer: Reactivity of Phosphagermenes and Phosphastannenes Towards Small Molecule Substrates

**DOI:** 10.1002/chem.202301542

**Published:** 2023-10-09

**Authors:** Matthew J. Reveley, Joey Feld, Diana Temerova, Eric S. Yang, Jose M. Goicoechea

**Affiliations:** ^1^ Department of Chemistry University of Oxford Chemistry Research Laboratory 12 Mansfield Rd. Oxford OX1 3TA UK; ^2^ Department of Chemistry Indiana University 800 E. Kirkwood Ave. Bloomington IN., 47405 USA

**Keywords:** phosphagermene, phosphastanne, phosphinidene, double bonds, metathesis

## Abstract

We describe the facile synthesis of [(Me_3_Si)_2_CH]_2_E=PMes* (E=Ge, Sn) from the reaction of the tetrylenes with the phospha‐Wittig reagent, Me_3_P−PMes*. Their reactivity towards a range of substrates with protic and hydridic E−H bonds (E=N, O, Si) is described. In addition to hydroelementation reactions of the E=P bonds, we show that these compounds, particularly [(Me_3_Si)_2_CH]_2_Sn=PMes*, also act as base‐stabilized phosphinidenes, allowing phosphinidene transfer to other nucleophiles.

## Graphical abstract

The synthesis of [(Me_3_Si)_2_CH]_2_E=PMes* (E=Ge, Sn) from the reaction of the tetrylenes with a phospha‐Wittig reagent is described. Reactivity studies reveal that they are capable of undergoing hydroelementation reactions across the E=P bond and of acting as masked phosphinidene sources.

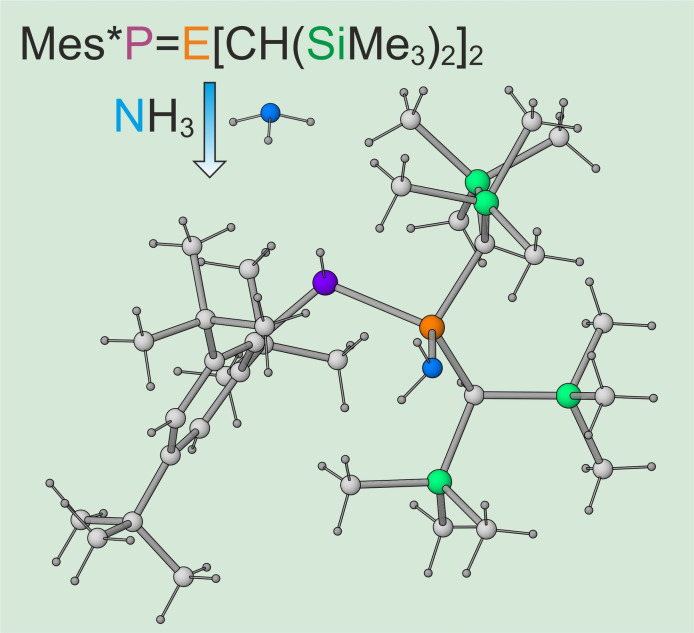

## Introduction

Compounds containing multiple bonds between the heavier main group elements (i. e. those with a principal quantum number *n*>2) have been of interest since the seminal discovery of molecules such as Lappert's distannene ([(Me_3_Si)_2_CH]_2_Sn=Sn[CH(SiMe_3_)_2_]_2_),[^1^, ^2^] West's disilene (Mes_2_Si=SiMes_2_; Mes=2,4,6‐trimethylphenyl),^[3]^ and Yoshifuji's diphosphene (Mes*P=PMes*; Mes*=2,4,6‐tri‐*tert*‐butylphenyl),^[4]^ to name a few landmark examples. Due to the weak nature of the π bonds in such molecules (relative to E−E σ bonds), they typically require kinetic and thermodynamic stabilization through use of bulky, strongly σ‐donating ligands. Weak secondary interactions, particularly dispersion forces, are also known to play a critical role in stabilizing these highly reactive compounds.[^5^, ^6^] This class of molecules has received increased attention over the last twenty years on account of their potential applications in both small molecule activation and catalysis.^[7]^

Compared to the heavier alkene analogues, R_2_E^IV^=E^IV^R_2_ (where E^IV^=group 14 element),^[8]^ compounds with heteroatomic double bonds between heavier main group elements are rarer still, because the difference in electronegativity between the elements confers a more polar, and hence, more reactive bond.^[9]^ Such species are more challenging to access as the reduction protocols typically used to synthesize compounds with E−E multiple bonds are not viable. An elegant strategy for the synthesis of compounds with E^III^=N (E^III^=Al−In) bonds was first reported by Power and co‐workers in 2001 involving the reaction of the group 13 carbenoid Ga(Nacnac) (Nacnac=HC[C(Me)N(Dipp)]_2_; Dipp=2,6‐diisopropylphenyl) with sterically encumbered organic azides (which afforded the targeted compounds with concomitant liberation of dinitrogen).[^10^, ^11^] This inspired us, and others, to explore the decarbonylation of phosphaketenes (R−P=C=O), themselves valence isoelectronic with azides, for the synthesis of compounds with Ga=P bonds.[^12^, ^13^] In parallel, a related strategy involving phosphine displacement from phospha‐Wittig reagents to access Al=P and Ga=P bonds was developed by Hering‐Junghans and colleagues.^[14]^ Compounds with E^III^=P (E^III^=Al, Ga) double bonds have gone on to show interesting reactivity towards a number of industrially relevant small molecule substrates including ammonia, carbon dioxide, alkenes and alkynes.[^15^, ^16^, ^17^]

**Figure 1:**
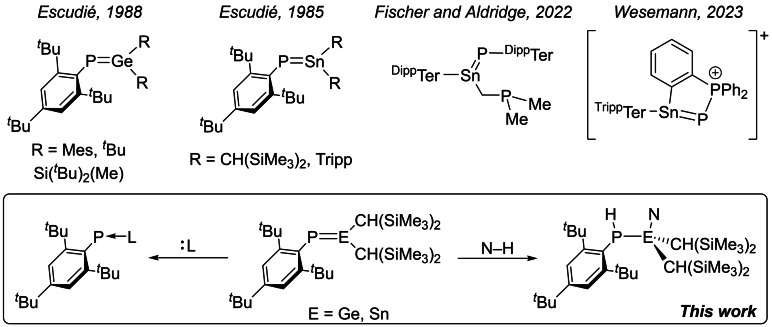
Known phosphagermenes and phosphastannenes.

These studies prompted us to ask whether similar approaches could be used to access species with E^IV^=P double bonds, a class of compounds which remain relatively unexplored. Early work by Escudié and co‐workers showed that phosphagermenes such as Mes_2_Ge=PMes* were isolable and could be structurally authenticated by single‐crystal X‐ray diffraction (Figure 1).^[18]^ Phosphasilenes would appear several years later,^[19]^ and have been extensively studied since.^[20]^ Tin‐containing analogues, i. e. phosphastannenes, were first reported in 1985,^[21]^ but until recently, had not been structurally authenticated.[^22^, ^23^, ^24^] Fischer, Aldridge and co‐workers showed that the reaction of phospha‐Wittig reagents, Me_3_P−PAr (Ar=Mes*, ^Mes^Ter, ^Dipp^Ter), with ^R^TerSn{N(SiMe_3_)_2_} could be used to access compounds with a Sn=P double bond.^[23]^ Stannaimines can also be accessed by reaction of ^R^TerSn{N(SiMe_3_)_2_} with azides.^[25]^ In this work, we show that a related transformation, involving phosphine‐dissociation from a phospha‐Wittig reagent (Me_3_P−PMes*) by monomeric germylenes and stannylenes, can also be used as a convenient method with which to access compounds with heteroatomic Ge=P and Sn=P double bonds. Despite having been known since the late 1980s, there are a dearth of reactivity studies on compounds with E=P double bonds. Herein we show that such highly polarized double bonds readily react with small molecule substrates including ammonia, amines, water and silanes in hydroelementation reactions.

## Results and Discussion

**Scheme 1:**
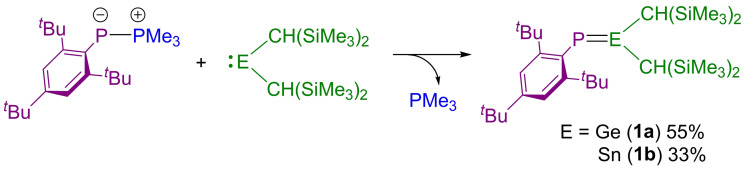
Synthesis of **1 a** and **1 b**.

The phospha‐Wittig (or phosphoranylidenephosphine) reagent Me_3_P−PMes* was synthesized according to a previously reported procedure.[^26^, ^27^] This species was reacted in a 1 : 1 ratio with E[CH(SiMe_3_)_2_]_2_ (E=Ge, Sn)^[2]^ to quantitively afford the phosphagermene and phosphastannene compounds [(SiMe_3_)_2_CH]_2_E=PMes*, where E=Ge (**1 a**) or Sn (**1 b**), as pictured in Scheme 1. *In situ* monitoring of the reaction mixtures by ^31^P{^1^H} NMR spectroscopy revealed the formation of novel compounds with resonances at 171.5 and 202.8 ppm for **1 a** and **1 b**, respectively, along with the formation of free PMe_3_. The ^1^H and ^13^C{^1^H} NMR spectra of compounds **1 a** and **1 b** are also in keeping with the proposed structures. Of note is that the methine proton resonances of the CH(SiMe_3_)_2_ groups are magnetically inequivalent (**1 a**: 1.44 and 0.93 ppm; **1 b**: 1.14, 0.74 ppm), indicating restricted rotation about the E−P bond, which is indicative of the formation of a double bond between the tetrel element and phosphorus. The same is also true of the methine carbon centers which appear as two distinct resonances in the ^13^C{^1^H} NMR spectra (**1 a**: 26.36 and 24.07 ppm; **1 b**: 34.38 and 25.31 ppm).

**Figure 2:**
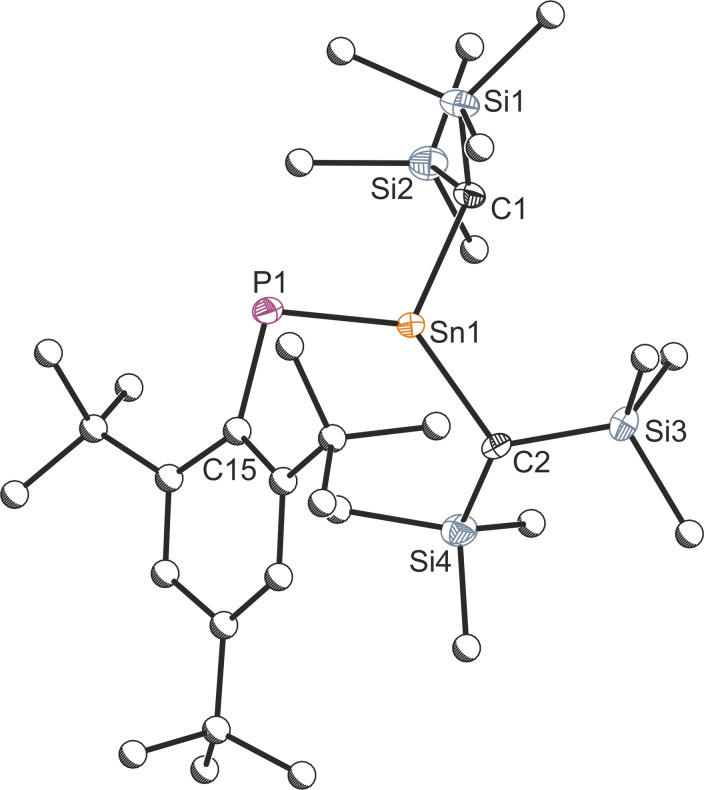
Single crystal X‐ray structure of **1 b**. Anisotropic displacement ellipsoids depicted at 50 % probability. All hydrogen atoms removed for clarity. Carbon atoms of Mes* and Me substituents pictured as spheres of arbitrary radius. Selected bond lengths (Å) and angles (°): P1−Sn1 2.343(1), P1−C15 1.859(4), Sn1−C1 2.166(4), Sn1−C2 2.165(4); C15−P1−Sn1 103.26(12), P1−Sn1−C1 113.54(11), P1−Sn1−C2 128.28(9), C1−Sn1−C2 117.60(14).

The structures of **1 a** and **1 b** were corroborated by single crystal X‐ray diffraction.^[28]^ It is worth noting at this stage that **1 b** has previously been synthesized by Escudié and co‐workers using a different synthetic strategy,^[21a]^ but has never been structurally authenticated (see Figure 2). The crystal structures of **1 a** and **1 b** reveal short Ge−P and Sn−P bonds of 2.157(1) and 2.343(1) Å, respectively, consistent with significant double bond character.^[29]^ It is worth noting that the sum of bond angles around the tetrel element centers is 359.7° and 359.4°, for **1 a** and **1 b**, respectively, which is agreeable with NMR evidence suggesting the presence of a P=E double bond.

With a convenient (albeit moderate‐yielding) synthesis of **1 a**/**1 b** in hand, we were interested to explore the reactivity of these compounds. Despite having been known for almost forty years, the reactivity of phosphagermenes and phosphastannenes has been the subject of limited study, in part due to the historic difficulties associated with their synthesis. This contrasts with a recent increase in activity studying the chemistry of compounds containing homoatomic multiple bonds such as digermynes,^[30]^ diborynes,^[31]^ and dialumenes,^[32]^ all of which have been shown to react with small molecule substrates including dihydrogen. Compounds with heteroatomic multiple bonds should, in principle, be more reactive as the polarity difference is anticipated to further favor heterolytic cleavage of inert bonds.

**Scheme 2:**
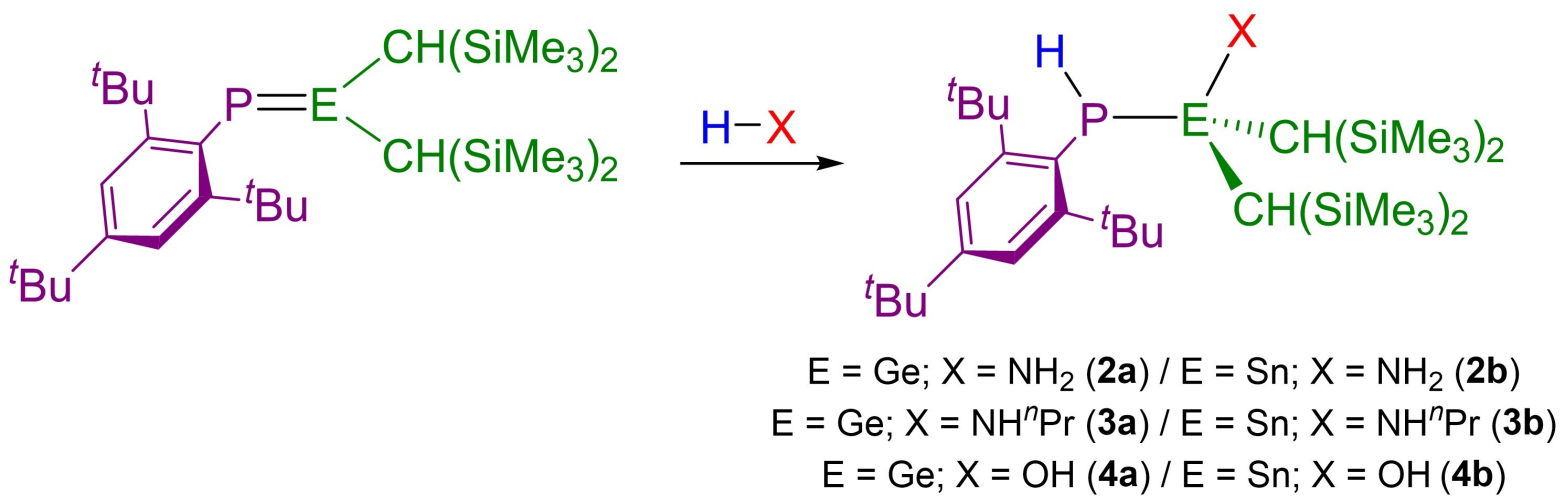
Reactivity of **1 a** and **1 b** towards NH_3_, NH_2_^*n*^Pr and H_2_O.

**Figure 3:**
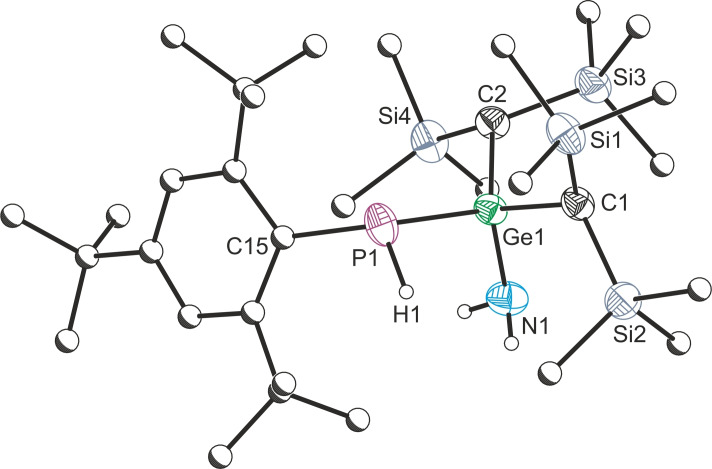
Single crystal X‐ray structure of **2 a**. Anisotropic displacement ellipsoids depicted at 50 % probability. All hydrogen atoms (except H1 and on those on the NH_2_ moiety) removed for clarity. Carbon atoms of Mes* and Me substituents pictured as spheres of arbitrary radius. Selected bond lengths (Å) and angles (°): P1−Ge1 2.394(1), P1−C15 1.861(2), P1−H1 1.29(5), Ge1−N1 1.883(2), Ge1−C1 1.997(2), Ge1−C2 1.981(2); C15−P1−Ge1 117.39(6), C15−P1−H1 109(2), P1−Ge1−N1 112.30(7), P1−Ge1−C1 99.88(6), P1−Ge1−C2 120.25(6).

Exposure of a solution of **1 a** to 1 bar of ammonia at room temperature in C_6_D_6_ caused no immediate reaction. However, upon heating at 80 °C for 22 hours, the solution decolorized and the ^31^P NMR spectrum showed a new doublet resonance at −95.4 ppm (^1^ *J*_P−H_=214 Hz) which collapses to a singlet upon proton decoupling (Scheme 2). The ^1^H NMR spectrum shows a resonance at 0.53 ppm for the NH_2_ moiety. The structure of the 1,2‐activation product **2 a** was confirmed by single‐crystal X‐ray diffraction, with regioselectivity in accordance with the ^δ+^Ge=P^δ−^ bond polarization (Figure 3).

Next, the ability of **1 a** to activate other protic E−H bonds was explored. Solutions of **1 a** react with *n*‐propylamine in C_6_D_6_ over 12 days at 80 °C to give **3 a** (δ(^31^P) NMR: −101.9 ppm; ^1^ *J*_P−H_=214 Hz), or with H_2_O in THF at ambient temperature to yield **4 a** (δ(^31^P) NMR: −96.1 ppm; ^1^ *J*_P−H_=209 Hz). In each case, the NMR spectra obtained were entirely as expected, and the products were characterized by multi‐element NMR spectroscopy. These compounds were also structurally authenticated by single crystal X‐ray diffraction (see Supporting Information for details). Our attempts to react **1 a** with *i*‐propylamine, or with aromatic amines (*p*‐anisidine or *p*‐toluidine) were unsuccessful, even upon heating for several days.

We next turned our attention towards investigating the reactivity of **1 b**. Escudié and co‐workers previously noted the high reactivity of **1 b** towards a variety of E−H bonds (E=C, N, O, S, Cl), however in many cases the outcome of such reactions remained unclear.^[33]^ Given our success with the reaction of **1 a** with ammonia, we sought to investigate the reactivity of **1 b** in more detail and by targeting more challenging substrates.

Exposure of a C_6_D_6_ solution of **1 b** to 1 bar of ammonia causes it to immediately decolorize. The ^31^P NMR spectrum displays a new doublet resonance (−113.6 ppm; ^1^ *J*_P−H_=206 Hz) corresponding to **2 b**, which is at a lower frequency compared to **2 a**. The NH_2_ moiety could also be located as a broad resonance in the ^1^H NMR spectrum (0.13 ppm). The ^119^Sn−^31^P coupling constant (^1^ *J*_Sn−P_=1019 Hz) is significantly lower than that recorded for **1 b** (^1^ *J*_Sn−P_=2292 Hz), reflecting a reduction in bond order. The NMR data compares favorably with the HCl and MeOH activation products reported by Escudié and co‐workers, e. g. [(SiMe_3_)_2_CH]_2_Sn(Cl)P(H)Mes* (δ(^31^P) NMR: −100.3 ppm; ^1^ *J*_P−H_=195 Hz) and [(SiMe_3_)_2_CH]_2_Sn(OMe)P(H)Mes* (δ(^31^P) NMR: −116.0 ppm; ^1^ *J*_P−H_=205 Hz).^[21a]^ **1 b** also rapidly reacts with *n*‐propylamine or H_2_O in C_6_D_6_ to give **3 b** (δ(^31^P) NMR: −117.6 ppm; ^1^ *J*_P−H_=206 Hz, ^1^ *J*_Sn−P_=973 Hz) or **4 b** (δ(^31^P) NMR: −111.2 ppm; ^1^ *J*_P−H_=205 Hz, ^1^ *J*_Sn−P_=1073 Hz), respectively. The latter reaction was also previously explored by Escudié although no analytical data for the resulting water‐activation product were reported.

**Scheme 3:**
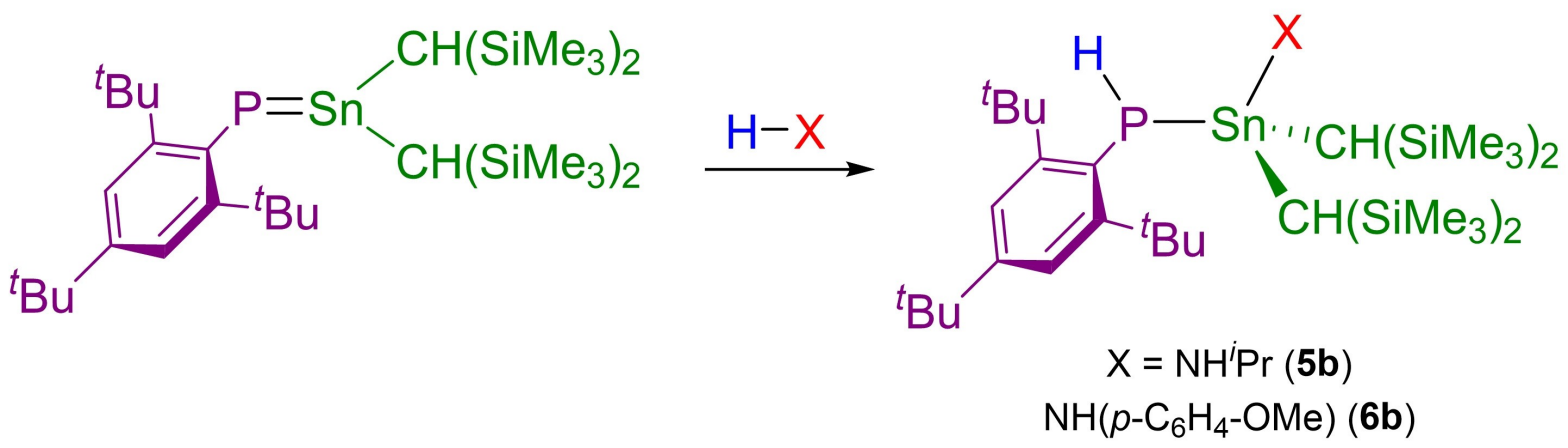
Reactivity of **1 b** towards NH_2_^*i*^Pr and *p*‐anisidine.

In contrast to the phosphagermene **1 a**, **1 b** also reacts with *i*‐propylamine in C_6_D_6_ at 80 °C for 20 minutes to give **5 b** (δ(^31^P) NMR: −103.2 ppm; ^1^ *J*_P−H_=207 Hz, ^1^ *J*_Sn−P_=959 Hz) or with *p*‐anisidine over 9 hours at the same temperature to give **6 b** (δ(^31^P) NMR: −117.9 ppm; ^1^ *J*_P−H_=207 Hz, ^1^ *J*_Sn−P_=1010 Hz) as pictured in Scheme 3. We note that upon leaving samples of **5 b** under a dynamic vacuum, the ^31^P NMR spectrum indicates reformation of **1 b** (~10 % by integration) suggesting that the reaction of **1 b** with *i*‐propylamine is reversible. All of the aforementioned products (**2 b**–**6 b**) were fully characterized by multi‐element NMR spectroscopy and single‐crystal X‐ray diffraction (see Supporting Information).

The comparative reactivities of **1 a** and **1 b** were probed using Density Functional Theory (DFT) calculations (see the Supporting Information for full details). For both compounds the HOMO and LUMO correspond to the E=P π bonding orbital and π* anti‐bonding orbital, respectively, though the HOMO‐LUMO gap of **1 a** (7.61 eV) is moderately higher than that of **1 b** (7.07 eV), indicating the increased reactivity for the π bond of the latter. The Wiberg Bond Indices (WBI) of the Ge−P bond in **1 a** (1.66) and the Sn−P bond in **1 b** (1.48) are both indicative of E=P double bond character, and the somewhat lower Sn=P bond order is consistent with its higher reactivity. Natural Population Analysis (NPA) of **1 a** and **1 b** shows a significant difference in E=P bond polarity. In **1 a**, the phosphorus atom has a partial negative charge of −0.16 and the germanium atom has a partial positive charge of 1.24, whereas in **1 b**, the difference in charge is much greater (P: −0.34, Sn: 1.48). This is also reflected in the polarity of the Natural Bond Orbitals (NBOs) corresponding to the Ge=P and Sn=P π and π* orbitals (π(Ge=P): 69.81 % P, 30.19 % Ge; π(Sn=P): 74.64 % P, 25.36 % Sn). The mechanism of ammonia activation by **1 a** and **1 b** was also investigated computationally and suggests a σ‐bond metathesis pathway with a higher activation barrier for **1 a** (29.7 kcal mol^−1^) than for **1 b** (20.5 kcal mol^−1^).

With these results showing the significantly increased reactivity of **1 b** compared to **1 a**, we sought to investigate whether secondary amines could be activated across the Sn=P double bond. While Hering‐Junghans and co‐workers reported the activation of piperidine using phospha‐Wittig reagents to form secondary aminophosphines,^[34]^ heating solutions of **1 b** with piperidine led only to decomposition by formation of Mes*PH_2_ (as determined by ^31^P NMR spectroscopy). We therefore turned our attention towards reacting **1 a**/**b** with amines containing more acidic N−H bonds, and selected imidazole for this purpose.

**Scheme 4:**
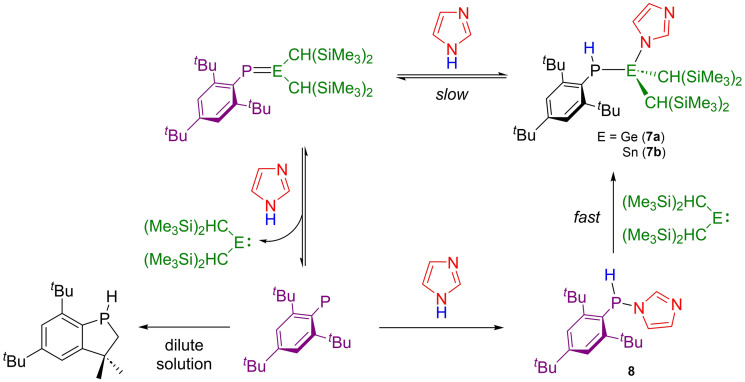
Simplified scheme describing the reactivity of **1 a** and **1 b** towards imidazole. For full experimental details see SI.

**Figure 4:**
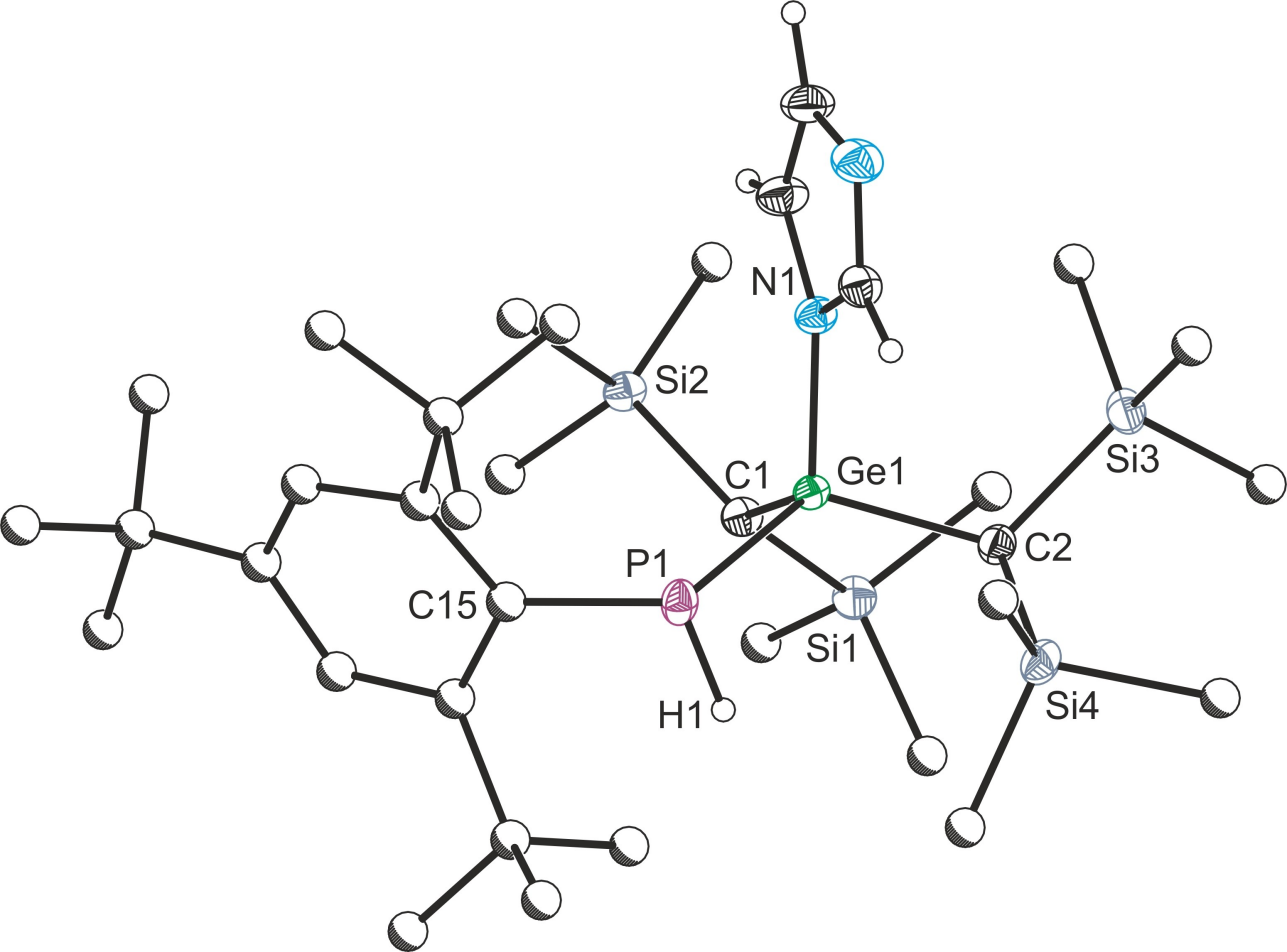
Single crystal X‐ray structure of **7 a**. Anisotropic displacement ellipsoids depicted at 50 % probability. All hydrogen atoms except H1 and imidazole protons removed for clarity. Carbon atoms of Mes* and Me substituents pictured as spheres of arbitrary radius. Selected bond lengths (Å) and angles (°): P1−Ge1 2.388(1), P1−C15 1.856(2), P1−H1 1.28(3), Ge1−N1 1.917(2), Ge1−C1 1.981(2), Ge1−C2 1.987(2); C15−P1−Ge1 109.98(4), C15−P1−H1 103.2(11), P1−Ge1−N1 103.88(3), P1−Ge1−C1 111.24(4), P1−Ge1−C2 109.40(4).

Imidazole reacts with **1 a** over 14 days at 80 °C to give **7 a** along with small amounts of unreacted **1 a** (~10 %) as pictured in Scheme 4. The ^31^P NMR spectrum shows a new resonance at −89.1 ppm (^1^ *J*_P−H_=219 Hz), in agreement with the obtained X‐ray structure (Figure 4). Our attempts to scale up the reaction led to an intractable mixture of products as determined by ^31^P NMR spectroscopy, including Mes*P=PMes* and Mes*PH_2_, precluding the bulk isolation of **7 a**. The diphosphene Mes*P=PMes* has been previously observed to result from phosphinidene dimerization, indicating that **1 a** may dissociate to form PMes*.

**Figure 5:**
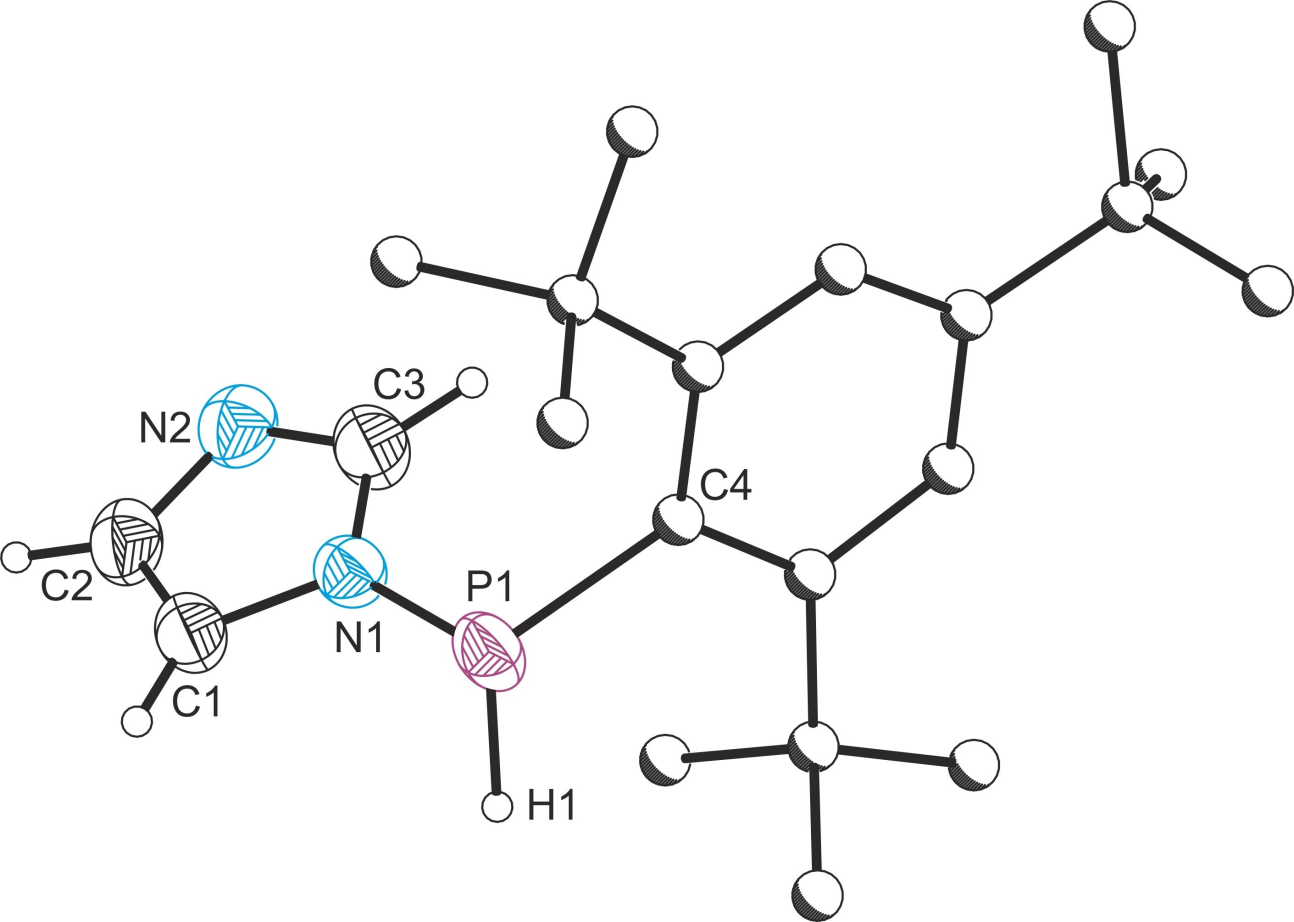
Single crystal X‐ray structure of **8**. Anisotropic displacement ellipsoids depicted at 50 % probability. Anisotropic displacement ellipsoids depicted at 50 % probability. All hydrogen atoms except H1 and imidazole protons removed for clarity. Carbon atoms of Mes* and Me substituents pictured as spheres of arbitrary radius. Selected bond lengths (Å) and angles (°): P1−N1 1.775(6), P1−C4 1.843(5), P1−H1 1.36(3); N1−P1−C4 102.1(2), N1−P1−H1 93.2(17), C4−P1−H1 115(3).

The addition of imidazole to a C_6_D_6_ solution of **1 b** caused the solution to rapidly decolorize. In contrast to the reactivity observed for other amines, two resonances are observed in the ^31^P NMR spectrum. The first, at −101.7 ppm (^1^ *J*_P−H_=209 Hz), displays ^119/117^Sn−^31^P satellites (^1^ *J*_Sn−P_ ~1165 Hz) consistent with the presence of a Sn−P single bond, and is therefore likely to correspond to the 1,2‐activation product **7 b** (Scheme 4). The second resonance is at significantly higher frequency (−7.9 ppm, ^1^ *J*_P−H_=239 Hz) and does not display ^119/117^Sn satellites. The identity of this product can be confirmed as the imidazole‐substituted phosphine **8**, which can be independently synthesized from Me_3_P−PMes* and imidazole (see Supporting Information). The structure of **8** was confirmed by single‐crystal X‐ray diffraction (Figure 5). We propose that following initial formation of **7 b**, small amounts of **1 b** are reformed by an equilibrium process (calc: ΔG^≠^_forward_=13.7 kcal mol^−1^; ΔG^≠^_reverse_=21.4 kcal mol^−1^). This allows for dissociation of the Sn=P double bond to transiently form the phosphinidene PMes* along with Sn[CH(SiMe_3_)_2_]_2_. Oxidative addition of the phosphinidene across the N−H bond of imidazole then forms **8**, while the back reaction is prevented by decomposition of the tin fragment (see Supporting Information for mechanistic investigations). As a result of the complexity of this reaction mixture, we were unable to isolate **7 b**, although comparison with the NMR spectra of **2 b**–**6 b** strongly supports formation of this compound.

We then moved on to investigate the reactivity of **1 a** and **1 b** towards hydridic E−H bonds. **1 a** was unreactive towards phenylsilane even upon heating, whereas **1 b** reacts with excess PhSiH_3_ at 80 °C for 3 days to form products consistent with phosphinidene generation (Mes*P=PMes* and 3,3‐dimethyl‐5,7‐di‐*tert*‐butylphosphaindane, the latter of which formed by C−H activation of a *tert*‐butyl group by the phosphinidene).[^35^, ^36^, ^37^] A singlet resonance at −147.5 ppm lacking tin satellites was also observed in the ^31^P{^1^H} NMR spectrum, which appears as a doublet of multiplets upon proton coupling (^1^ *J*_P−H_=215 Hz). We tentatively assign this resonance as the product of oxidative addition by the phosphinidene across the Si−H bond in phenylsilane to form Mes*P(H)(SiH_2_Ph) on account of similarities in its NMR spectra with other literature‐reported silyl‐phosphines such as PhP(H)(SiH_2_Ph) (δ(^31^P) NMR: −144.9 ppm) and PhP(H){SiH_2_(p‐tol)} (δ(^31^P) NMR: −140.2 ppm).^[38]^ Unfortunately, crystals of Mes*P(H)(SiH_2_Ph) could not be isolated, and we were unable to determine the fate of the tin fragment in this reaction.

Nevertheless, these results provide evidence that in addition to the expected mode of reactivity involving activation of substrates across the E=P double bond, **1 a** and **1 b** also display reactivity characteristic of base‐stabilized phosphinidenes. A phosphagallene which retains significant base‐stabilized phosphinidene character has recently been reported by Hering‐Junghans and co‐workers,^[14b]^ but its reaction with protic substrates resulted in cleavage of the Ga−P bond. To the best of our knowledge, the retention of reactivity across the double bond with protic substrates in addition to phosphinidene‐like behavior has not been reported for Ge=P or Sn=P double bonds. With this in mind, we turned our attention towards employing these compounds as phosphinidene transfer reagents.

Heating **1 b** at 80 °C for 3 days, or UV irradiation of **1 a**/**b**, causes dissociation of the E=P double bond and transiently forms PMes*, which is observed by NMR spectroscopy after activation of one of the C−H bonds of the *tert*‐butyl groups, i. e. 3,3‐dimethyl‐5,7‐di‐*tert*‐butylphosphaindane. The corresponding tetrylene is also formed in this process. The dissociation of **1 a** and **1 b** were investigated computationally on the singlet and triplet potential energy surfaces. The minimum energy crossing point (MECP) for thermal dissociation was found to be prohibitively high in energy for **1 a** (40.7 kcal mol^−1^), though achievable for **1 b** (34.3 kcal mol^−1^), consistent with the observed thermal and photochemical reactivity.

As imidazole is proposed to initiate dissociation in the absence of irradiation or heating, we investigated whether this was possible with other reagents. Fischer, Aldridge and co‐workers reported that the formation of an Sn=P bond in the reactions they studied could be prevented by addition of an NHC, which interacts with the vacant orbital at tin. Addition of excess IMe_4_ to a solution of **1 b** in C_6_D_6_ causes immediate formation of a new product with a singlet resonance in the ^31^P{^1^H} NMR spectrum at −47.6 ppm, corresponding to the known NHC‐phosphinidene adduct Mes*P−IMe_4_.^[26]^ The formation of Mes*P−IMe_4_ by this route is significantly faster than from the phospha‐Wittig reagent Me_3_P−PMes* in spite of the increased steric hindrance from the stannylene fragment. We presume that this reaction proceeds by a similar mechanism to that described by Fischer et al. but were unable to identify the fate of the tin‐containing fragment.

Finally, we wanted to investigate whether **1 b** could be used as a phosphinidene source in the synthesis of other heavy double bonds. An example of a useful heavy double bond metathesis reaction was reported by Scheschkewitz and co‐workers using Ge=Ge double bonds to yield a polydigermene.^[39]^ Although phospha‐Wittig reagents have been shown to be successful synthons in the synthesis of E=P double bonds, the use of heavy double bonds themselves to form other heavy heteroatomic double bonds is, to the best of our knowledge, unprecedented. As a proof of concept, we reacted **1 b** with Ge[CH(SiMe_3_)_2_]_2_. At room temperature, no reaction was observed between **1 b** and germylene after 3 days, in line with the increased steric demand compared to the smaller IMe_4_. However, heating at 80 °C for 18 hours led to the conversion of **1 b** to **1 a** in addition to small amounts of 3,3‐dimethyl‐5,7‐di‐*tert*‐butylphosphaindane. In the ^1^H NMR spectrum, formation of free stannylene is evident, suggesting that this reaction does not proceed through a decomposition pathway akin to that for imidazole. This reaction demonstrates an example of phosphinidene metathesis from **1 b** to form other E=P double bonds.

## Conclusions

In summary, we show the facile synthesis of phosphatetrylenes **1 a** and **1 b** from phospha‐Wittig reagents and the corresponding tetrylene. Their reactivity towards a range of protic and hydridic E−H substrates (E=N, O, Si) was compared, showing that the more polar heteroatomic double bond in **1 b** is able to more effectively activate a wider range of small molecules. In addition to traditional “double bond” character, we show that **1 a**, and particularly **1 b**, also possess reactivity modes as base‐stabilized phosphinidenes, allowing phosphinidene metathesis towards other nucleophiles. The varied reactivity modes shown by E^IV^=P (E^IV^=Ge, Sn) double bonds offers novel routes towards activating small molecules, which may provide opportunities to react with more challenging substrates crucial to the development of industrially relevant catalytic processes. Future work will target the use of **1 a** and **1 b** as phosphinidene transfer reagents to access novel E=P bonds, further exploring the scope of their phosphinidene reactivity.

## Experimental Section

For spectra and more detailed spectral assignments, please refer to the Supporting Information document.

**Synthetic methods**: All reactions and product manipulations were carried out using standard Schlenk‐line techniques under an inert atmosphere of argon, or in a dinitrogen filled glovebox (MBraun UNIlab glovebox maintained at <0.1 ppm H_2_O and <0.1 ppm O_2_). The compounds Ge[(CH(SiMe_3_)_2_]_2_,^[2]^ Sn[(CH(SiMe_3_)_2_]_2_,^[1]^ Me_3_P−PMes*,^[26]^ and IMe_4_ ^[40]^ (IMe_4_=C[N(Me)C(Me)]_2_) were prepared according to previously reported procedures. Toluene (Sigma Aldrich, HPLC grade), hexane (Sigma Aldrich, HPLC grade), and pentane (Sigma Aldrich, HPLC grade) were purified using an MBraun SPS‐800 solvent system. THF (Sigma Aldrich, HPLC grade) was distilled over sodium/benzophenone. Hexamethyldisiloxane (Sigma Aldrich, ≥99.5 %) and C_6_D_6_ (Aldrich, 99.5 %) were dried over CaH_2_ and degassed. All dry solvents were stored under argon in gas‐tight ampoules. All solvents were stored over activated 3 Å molecular sieves. ^*n*^PrNH_2_ (Alfa Aesar, 99 %+), ^*i*^PrNH_2_ (Alfa Aesar, 99 %+) and PhSiH_3_ (Chem Cruz, 97 %) were distilled and degassed prior to storage over activated 3 Å molecular sieves. *Para*‐anisidine (Alfa Aesar, 99 %) was sublimed prior to use. Imidazole (Sigma Aldrich, >99 %) was sublimed then recrystalised from acetone/pentane prior to use. NH_3_ (BOC, 99.98 %) was used as purchased without further purification.

**Characterization techniques**: NMR spectra were acquired on a Bruker AVIII HD 500 MHz NMR spectrometer (^1^H 500 MHz, ^31^P 202 MHz, ^119^Sn 186 MHz), Bruker AVIII 400 MHz NMR spectrometer (^1^H 400 MHz, ^13^C 101 MHz, ^31^P 162 MHz) or a Bruker Avance NEO 600 MHz NMR spectrometer with a broadband helium cryoprobe (^13^C 151 MHz). ^1^H and ^13^C NMR spectra were referenced to the most downfield solvent resonance (^1^H NMR C_6_D_6_: δ=7.16 ppm; ^13^C NMR C_6_D_6_: δ=128.06 ppm). ^31^P spectra were externally referenced to an 85 % solution of H_3_PO_4_ in H_2_O. Raman spectra were acquired on a Thermo Scientific DXR3 SmartRaman spectrometer using a 785 nm laser. Elemental analyses were carried out by London Metropolitan University (London, U.K.). Samples (approx. 10 mg) were submitted in sealed vials under an inert atmosphere.

Synthesis of [(Me_3_Si)_2_CH]_2_Ge=PMes* (1 a): Ge[CH(SiMe_3_)_2_]_2_ (100 mg, 0.255 mmol) and Me_3_P−PMes* (89.9 mg, 0.255 mmol) were stirred for an hour in toluene until all the solids were dissolved. All volatiles were removed under vacuum and the product was recrystallised from hexane to yield yellow crystals (94 mg, 0.14 mmol, 54.9 % yield). Anal. calculated for C_32_H_67_GePSi_4_: C, 57.55; H, 10.11; N, 0. Found: C, 57.38; H, 10.15; N, 0. ^1^H NMR (400 MHz, C_6_D_6_): δ (ppm) 7.49 (s, 2H; ArC*H*), 1.79 (s, 18H; *o*‐C(C*H*_3_)_3_), 1.44 (s, 1H, GeC*H*), 1.38 (s, 9H; *p*‐C(C*H*_3_)_3_), 0.93 (d, 1H, ^3^ *J*_P−H_=15.4 Hz; GeC*H*) 0.43 (s, 18H; Si(C*H*_3_)_3_), 0.05 (s, 18H; Si(C*H*_3_)_3_). ^13^C NMR (101 MHz, C_6_D_6_): δ (ppm) 154.75 (*o*‐Ar*C*), 147.99 (*p*‐Ar*C*), 136.04 (*i*‐Ar*C*), 121.79 (*m*‐Ar*C*), 39.12 (*o*‐*C*(CH_3_)_3_), 34.85 (*p*‐*C*(CH_3_)_3_), 33.84 (d, ^4^ *J*_P−C_=7.72 Hz; *o*‐C(*C*H_3_)_3_), 31.81 (*p*‐C(*C*H_3_)_3_), 26.36 (d, ^2^ *J*_P−C_=23.1 Hz; Ge*C*H), 24.07 (d, ^2^ *J*_P−C_=14.2 Hz; Ge*C*H), 3.99 (d, ^4^ *J*_P−C_=4.0 Hz; Si(*C*H_3_)_3_), 3.83 (Si(*C*H_3_)_3_). ^31^P NMR (162 MHz, C_6_D_6_): δ (ppm) 171.5 (s). UV‐vis: λ_max_ (nm) 323, 358 (shoulder).

Synthesis of [(Me_3_Si)_2_CH]_2_Sn=PMes* (1 b): Sn[CH(SiMe_3_)_2_]_2_ (100 mg, 0.229 mmol) and Me_3_P−PMes* (81 mg, 0.23 mmol) were stirred in toluene (5 mL) until all the solids were dissolved. All volatiles were removed under vacuum and the product was recrystallised from hexane/hexamethyldisiloxane to yield red crystals. (54.4 mg, 0.076 mmol, 33.2 % yield). Anal. calculated for C_32_H_67_PSnSi_4_: C, 53.84; H, 9.46; N, 0.00. Found: C, 53.78; H, 9.50; N, 0.00. ^1^H NMR (400 MHz, C_6_D_6_): δ (ppm) 7.50 (s, 2H; ArC*H*), 1.82 (s, 18H; *o*‐C(C*H*_3_)_3_), 1.38 (s, 9H; *p*‐C(C*H*_3_)_3_), 1.14 (s, 1H; SnC*H*), 0.74 (d, ^3^ *J*_P−H_=7.9 Hz, 1H; SnC*H*), 0.41 (s, 18H; Si(C*H*_3_)_3_), 0.03 (s, 18H; Si(C*H*_3_)_3_). ^13^C NMR (151 MHz, C_6_D_6_): δ (ppm) 154.68 (*o*‐Ar*C*), 148.25 (*p*‐Ar*C*), 135.50 (d, ^1^ *J*_P−C_=82.9 Hz; *i*‐Ar*C*), 121.50 (*m*‐Ar*C*), 39.18 (*o*‐*C*(CH_3_)_3_), 34.83 (*p*‐*C*(CH_3_)_3_), 34.38 (d, ^2^ *J*_P−C_=22.6 Hz; Sn*C*H), 33.89 (d, ^4^ *J*_P−C_=7.8 Hz; *o*‐C(*C*H_3_)_3_), 31.96 (*p*‐C(*C*H_3_)_3_), 25.31 (Sn*C*H), 4.34 (d, ^4^ *J*_P−C_=3.5 Hz; Si(*C*H_3_)_3_), 4.07 (Si(*C*H_3_)_3_). ^31^P NMR (162 MHz, C_6_D_6_): δ (ppm) 202.8 (s). ^119^Sn NMR (186 MHz, C_6_D_6_): δ (ppm) 660.4 (d, ^1^ *J*_Sn−P_=2292 Hz). UV‐vis: λ_max_ (nm) 412.

Synthesis of [(Me_3_Si)_2_CH]_2_Ge(NH_2_)P(H)Mes* (2 a): **1 b** (30 mg, 0.045 mmol) was dissolved in toluene in an NMR tube and put under 1 bar of NH_3_. The tube was heated to 80 °C for 22 hours, becoming almost colourless. The volatiles were removed *in vacuo* and the solids extracted with hexane (3×0.5 mL). A small amount of hexamethyldisiloxane (0.1 mL) was added. Slow evaporation of the hexane/hexamethyldisiloxane solution at room temperature yielded colourless crystals which were washed with cold HMDSO and dried *in vacuo* (14.1 mg, 0.021 mmol, 45.8 % yield). Anal. calculated for C_32_H_70_GeNP_1_Si_4_: C, 56.12; H, 10.30; N, 2.05. Found: C, 55.88; H, 10.51; N, 1.74. ^1^H NMR (400 MHz, C_6_D_6_): δ (ppm) 7.48 (d, ^4^ *J*_P−H_=2.4 Hz, 2H; ArC*H*), 4.83 (d, ^1^ *J*_P−H_=213.6 Hz, 1H; P*H*), 1.68 (s, 18H; *o*‐C(C*H*_3_)_3_), 1.33 (s, 9H; *p*‐C(C*H*_3_)_3_), 0.53 (s, 2H; GeN*H*_2_), 0.36 (s(br), 18H; Si(C*H*_3_)_3_), 0.21 (s, 18H; Si(C*H*_3_)_3_). Note: GeC*H* protons were observed in 2D spectra but were not assigned in 1D spectra due to overlap with peaks at 0.36 ppm. ^13^C NMR (162 MHz, C_6_D_6_): δ (ppm) 155.71 (*o*‐Ar*C*), 148.19 (*p*‐Ar*C*), 129.62 (d, ^1^ *J*_P−C_=49.4 Hz; *i*‐Ar*C*), 122.61 (d, ^3^ *J*_P−C_=3.3 Hz; *m*‐Ar*C*), 38.83 (*o*‐*C*(CH_3_)_3_), 34.85 (*p*‐*C*(CH_3_)_3_), 34.21 (*o*‐C(*C*H_3_)_3_), 31.57 (*p*‐C(*C*H_3_)_3_), 12.36 (Ge*C*H), 11.48 (Ge*C*H), 4.69 (Si(*C*H_3_)_3_), 4.66 (Si(*C*H_3_)_3_). ^31^P NMR (162 MHz, C_6_D_6_): δ (ppm) −95.4 (d, ^1^ *J*_P−H_=214 Hz).

**Synthesis of [(Me_3_Si)_2_CH]_2_Ge(NH** ^***n***^ Pr)P(H)Mes* (3 a): **1 b** (50 mg, 0.075 mmol) was dissolved in toluene (0.6 mL) in an NMR tube before *n*‐propylamine (0.01 mL, in excess) was added and the solution heated at 80 °C for 12 days. The volatiles were removed *in vacuo* and the solid residue was extracted with hexane (3×0.5 mL). Slow evaporation of the hexane solution at room temperature yielded colourless cubic crystals (25.9 mg, 0.036 mmol, 48.0 % yield). Anal. calculated for C_35_H_76_GeNPSi_4_: C, 57.83; H, 10.54; N, 1.93. Found: C, 57.88; H, 10.18; N, 1.91. ^1^H NMR (400 MHz, C_6_D_6_): δ (ppm) 7.45 (d, ^4^ *J*_P−H_=2.5 Hz, 2H; ArC*H*), 4.94 (d, ^1^ *J*_P−H_=214 Hz; P*H*), 2.92 (m, 2H; NCH_2_C*H*_2_CH_3_), 1.69 (s, 18H; *o*‐C(C*H*_3_)_3_), 1.49 (m(br), 2H; NC*H*_2_CH_2_CH_3_), 1.34 (s, 9H; *p*‐C(C*H*_3_)_3_), 0.94 (t, ^1^ *J*_H−H_=7.4 Hz, 3H; NCH_2_CH_2_C*H*_3_) 0.78 (t, ^1^ *J*_H−H_=7.5 Hz, 1H; N*H*CH_2_), 0.38 (s, 18H; Si(C*H*_3_)_3_), 0.28 (m, 18H; Si(C*H*_3_)_3_). Note: GeC*H* protons were observed in 2D spectra but were not assigned in 1D spectra due to overlap with peaks at 0.35 and 0.21 ppm. ^13^C NMR (151 MHz, C_6_D_6_): δ (ppm): 155.09 (d, ^2^ *J*_P−C_=6.7 Hz; *o*‐Ar*C*), 147.62 (*p*‐Ar*C*), 130.52 (d, ^1^ *J*_P−C_=48.7 Hz; *i*‐Ar*C*), 122.32 (*m*‐Ar*C*), 47.57 (NCH_2_ *C*H_2_CH_3_), 38.70 (*o*‐*C*(CH_3_)_3_), 34.79 (*p*‐*C*(CH_3_)_3_), 33.86 (d, ^4^ *J*_P−C_=7.1 Hz; *o*‐C(*C*H_3_)_3_), 31.65 (*p*‐C(*C*H_3_)_3_), 27.19 (N*C*H_2_CH_2_CH_3_), 11.72 (NCH_2_CH_2_ *C*H_3_), 10.81 (Ge*C*H), 9.57 (Ge*C*H), 5.49 (Si(*C*H_3_)_3_), 5.46 (Si(*C*H_3_)_3_), 5.18 (Si(*C*H_3_)_3_). ^31^P NMR (162 MHz, C_6_D_6_): δ (ppm) −101.9 (d, ^1^ *J*_P−H_=214 Hz).

Synthesis of [(Me_3_Si)_2_CH]_2_Ge(OH)P(H)Mes* (4 a): 1 b was made *in situ* by stirring Ge[CH(SiMe_3_)_2_]_2_ (33 mg, 0.0851 mmol) and Me_3_P−PMes* (30 mg, 0.0851 mmol) in toluene (1 mL) until all the solids were dissolved. The solution was put under reduced pressure to remove the generated PMe_3_ and re‐dissolved in THF (0.5 mL). Degassed H_2_O (0.02 mL, 1.11 mmol) was added and the solution was left stirring for 3 h. The volatiles were removed and was extracted with hexane (3×0.5 mL). A small amount of hexamethyldisiloxane (0.1 mL) was added. Slow evaporation of the hexane/hexamethyldisiloxane solution at room temperature yielded colourless crystals. (25.6 mg, 0.037 mmol, 43.5 % yield). Anal. calculated for C_35_H_69_GeOPSi_4_: C, 56.04; H, 10.14; N, 0.00. Found: C, 55.98; H, 10.31; N, 0.00. ^1^H NMR (400 MHz, C_6_D_6_): δ (ppm) 7.48 (s, 2H; ArC*H*), 5.11 (d, ^1^ *J*_P−H_=210 Hz; P*H*), 1.64 (s, 18H; *o*‐C(C*H*_3_)_3_), 1.31 (s, 9H; *p*‐C(C*H*_3_)_3_), 0.95 (s, 1H; GeC*H*), 0.45 (br, 2H; GeC*H*), 0.36 (d, *J*=11.9 Hz, 18H; Si(C*H*_3_)_3_), 0.21 (d, *J*=19.2 Hz, 18H; Si(C*H*_3_)_3_). ^13^C NMR (151 MHz, C_6_D_6_): δ (ppm) 155.88 (*o*‐Ar*C*), 148.42 (*p*‐Ar*C*), 127.62 (*i*‐Ar*C*), 123.03 (*m*‐Ar*C*), 39.07 (*o*‐*C*(CH_3_)_3_), 34.84 (*p*‐*C*(CH_3_)_3_), 34.29 (br; *o*‐C(*C*H_3_)_3_), 31.38 (*p*‐C(*C*H_3_)_3_), 14.60 (Ge*C*H), 13.33 (Ge*C*H), 4.66 (Si(*C*H_3_)_3_), 4.36 (Si(*C*H_3_)_3_). ^31^P NMR (162 MHz, C_6_D_6_): δ (ppm) −96.1 (d, ^1^ *J*_P−H_=209 Hz).

Synthesis of [(Me_3_Si)_2_CH]_2_Sn(NH_2_)P(H)Mes* (2 b): 1 b was prepared *in situ* by reacting Sn[CH(SiMe_3_)_2_]_2_ (75 mg, 0.171 mmol) with Me_3_P−PMes* (60.4mg, 0.171 mmol) in toluene (2 mL). The volatiles were removed *in vacuo* to remove the generated PMe_3_ and the solids redissolved in toluene (2 mL). The solution was degassed using the freeze‐pump‐thaw method and put under 1 bar NH_3_ gas. The solution immediately decolourised. The volatiles were removed *in vacuo* and the solids extracted with *n*‐pentane (3×1 mL). A small amount of hexamethyldisiloxane (0.1 mL) was added. Slow evaporation of the *n*‐pentane/hexamethyldisiloxane solution at room temperature yielded colourless crystals which were filtered and washed with a small amount of cold HMDSO (37.2 mg, 0.051 mmol, 29.8 %). Anal. calculated for C_32_H_70_NPSi_4_Sn: C, 52.58; H, 9.65; N, 1.92. Found: C, 52.91; H, 9.55; N, 1.66. ^1^H NMR (400 MHz, C_6_D_6_): δ (ppm) 7.48 (d, ^4^ *J*_P−H_=2.5 Hz, 2H; ArC*H*), 5.05 (d, ^1^ *J*_P−H_=206 Hz, 1H; P*H*), 1.70 (s, 18H; *o*‐C(C*H*_3_)_3_), 1.32 (s, 9H; *p*‐C(C*H*_3_)_3_), 0.35 (s, 9H; Si(C*H*_3_)_3_), 0.33 (s, 9H; Si(C*H*_3_)_3_), 0.19 (s, 18H; Si(C*H*_3_)_3_), 0.13 (s (br), 2H, N*H*_2_). Note: SnC*H* protons were observed in 2D spectra but were not assigned in 1D spectra due to overlap with peaks at 0.19 ppm. ^13^C NMR (151 MHz, C_6_D_6_): δ (ppm) 155.27 (*o*‐Ar*C*), 148.55 (*p*‐Ar*C*), 122.51 (d, ^4^ *J*_P−C_=3.3 Hz; *m*‐Ar*C*), 38.50 (*o*‐*C*(CH_3_)_3_), 34.91 (*p*‐*C*(CH_3_)_3_), 33.75 (d, ^4^ *J*_P−C_=7.1 Hz; *o*‐C(*C*H_3_)_3_), 31.52 (*p*‐C(*C*H_3_)_3_), 10.08 (Sn*C*H), 9.37 (d, ^3^ *J*_P−C_=6.7 Hz, Sn*C*H), 4.52 (Si(*C*H_3_)_3_), 4.42 (Si(*C*H_3_)_3_). Note: Mes* *ipso*‐Ar*C* could not be located. ^31^P NMR (162 MHz, C_6_D_6_): δ (ppm) −113.6 (d, ^1^ *J*_P−H_=206 Hz). ^119^Sn NMR (186 MHz, C_6_D_6_): δ (ppm) 56.1 (d, ^1^ *J*_Sn−P_=1019 Hz).

**Synthesis of [(Me_3_Si)_2_CH]_2_Sn(NH** ^*n*^ Pr)P(H)Mes* (3 b): 1 b was made *in situ* by stirring Sn[CH(SiMe_3_)_2_]_2_ (37 mg, 0.0851 mmol) and Me_3_P−PMes* (30 mg, 0.0851 mmol) in toluene (3 mL) until all the solids were dissolved. The solution was put under reduced pressure to remove the generated PMe_3_ and re‐dissolved in toluene (3 mL). *n‐*propylamine (0.02 mL, 1.11 mmol) was added and the solution was stirred for 20 mins, during which the solution decolourised. The volatiles were removed and was extracted with hexane (3×0.5 mL). A small amount of hexamethyldisiloxane (0.1 mL) was added. Slow evaporation of the hexane/hexamethyldisiloxane solution at room temperature yielded colourless cubic crystals (24.7 mg, 0.032 mmol, 37.6 % yield). Anal. calculated for C_35_H_76_NPSnSi_4_: C, 54.38; H, 9.91; N, 1.81. Found: C, 54.54; H, 10.22; N, 1.78. ^1^H NMR (600 MHz, C_6_D_6_): δ (ppm) 7.46 (s, ^4^ *J*_P−H_=2.5 Hz, 2H; ArC*H*), 5.04 (d, ^1^ *J*_P−H_=205.2 Hz; P*H*), 3.07–2.96 (m, 2H; NC*H*_2_CH_2_CH_3_), 1.70 (s, 18H; *o*‐C(C*H*_3_)_3_), 1.59–1.47 (m, 2H; NCH_2_C*H*_2_CH_3_), 1.33 (s, 9H; *p*‐C(C*H*_3_)_3_), 0.96 (t, ^3^ *J*_H−H_=7.4 Hz, 3H; NCH_2_CH_2_C*H*_3_), 0.41 (s, 1H; N*H*), 0.36 (s, 18H; Si(C*H*_3_)_3_), 0.33 (s(br), 9H; Si(C*H*_3_)_3_), 0.19 (s(br), 9H; Si(C*H*_3_)_3_). ^13^C NMR (151 MHz, C_6_D_6_): δ (ppm) 154.85 (*o*‐Ar*C*), 147.84 (*p*‐Ar*C*), 129.35 (d, ^1^ *J*_P−C_=50.7 Hz; *i*‐Ar*C*), 122.34 (*m*‐Ar*C*), 49.50 (N*C*H_2_), 38.65 (*o*‐*C*(CH_3_)_3_), 34.85 (*p*‐*C*(CH_3_)_3_), 33.63 (d, ^5^ *J*_P−C_=7.3 Hz; *o*‐C(*C*H_3_)_3_, 31.64 (*p*‐C(*C*H_3_)_3_, 28.78 (NCH_2_ *C*H_2_CH_3_), 11.58 (NCH_2_CH_2_ *C*H_3_), 9.08 (Sn*C*H), 7.89 (Sn*C*H), 5.00 (Si(*C*H_3_)_3_), 4.95 (Si(*C*H_3_)_3_), 4.76 (Si(*C*H_3_)_3_). ^31^P NMR (162 MHz, C_6_D_6_): δ (ppm) −117.6 (d, ^1^ *J*_P−H_=206 Hz). ^119^Sn NMR (186 MHz, C_6_D_6_): δ (ppm) 49.4 (d, ^1^ *J*_Sn−P_=973 Hz).

Synthesis of [(Me_3_Si)_2_CH]_2_Sn(OH)P(H)Mes* (4 b): 1 b was made *in situ* by stirring Sn[CH(SiMe_3_)_2_]_2_ (37 mg, 0.0851 mmol) and Me_3_P−PMes* (30 mg, 0.0851 mmol) in toluene (1 mL) until all the solids were dissolved. The solution was put under reduced pressure to remove the generated PMe_3_ and re‐dissolved in toluene (0.5 mL). Degassed H_2_O (0.02 mL, 1.11 mmol) was added and the solution was shaken for 30 seconds. The solution immediately decolourised. The volatiles were removed and the solids were extracted with hexane (3×0.5 mL). Slow evaporation of the hexane solution at room temperature yielded colourless crystals (15 mg, 0.0205 mmol, 24.1 % yield). Anal. calculated for C_32_H_69_OPSnSi_4_: C, 52.51; H, 9.50; N, 0.00. Found: C, 52.96; H, 9.84; N, 0.00. ^1^H NMR (400 MHz, C_6_D_6_): δ (ppm) 7.48 (s, 2H; ArC*H*), 5.11 (d, ^1^ *J*_P−H_=205.5 Hz; P*H*), 1.68 (s, 18H; *o*‐C(C*H*_3_)_3_), 1.30 (s, 9H; *p*‐C(C*H*_3_)_3_), 0.36 (s, 18H; Si(C*H*_3_)_3_), 0.30 (s, 1H, SnC*H*), 0.25 (s, 1H; SnC*H*), 0.19 (m, 18H; Si(C*H*_3_)_3_). Note: We were unable to observe the SnO*H* resonance. ^13^C NMR (151 MHz, C_6_D_6_): δ (ppm) 155.48 (*o*‐Ar*C*), 149.00 (*p*‐Ar*C*), 126.96 (*i*‐Ar*C*), 122.73 (*m*‐Ar*C*), 122.71 (*m*‐Ar*C*), 38.55 (*o*‐*C*(CH_3_)_3_), 34.92 (*p*‐*C*(CH_3_)_3_), 33.77 (d, ^5^ *J*_P−C_=7.2 Hz; *o*‐C(*C*H_3_)_3_), 31.49 (*p*‐C(*C*H_3_)_3_), 13.99 (Sn*C*H), 13.16 (Sn*C*H), 4.38 (Si(*C*H_3_)_3_), 4.32 (Si(*C*H_3_)_3_). ^31^P NMR (162 MHz, C_6_D_6_): δ (ppm) −111.2 (d, ^1^ *J*_P−H_=205 Hz). ^119^Sn NMR (186 MHz, C_6_D_6_): δ (ppm) 80.7 (d, ^1^ *J*_Sn−P_=1073 Hz).

**Synthesis of [(Me_3_Si)_2_CH]_2_Sn(NH** ^***i***^ Pr)P(H)Mes* (5 b): 1 b was generated *in situ* by stirring Sn[CH(SiMe_3_)_2_]_2_ (75 mg, 0.171 mmol) and Me_3_P−PMes* (60.4 mg, 0.171 mmol) in toluene (2 mL) until the solids dissolved. The volatiles were removed *in vacuo* followed by redissolving the remaining solids in toluene (2 mL). Isopropylamine (0.05 mL, 0.582 mmol, in excess) was added and the solution heated at 80 °C for 20 minutes, causing the colour to fade. After stirring at room temperature for a further 30 minutes, the volatiles were removed *in vacuo* and the solids extracted with pentane (3×1 mL). A small amount of hexamethyldisiloxane (0.1 mL) was added. Slow evaporation of the pentane/hexamethyldisiloxane solution at room temperature gave an orange oil that was cooled to −35 °C for 24 hours. Pentane (3 drops) was added, crystals were formed, and the solution cooled again to −35 °C for 2 hours. The liquid was decanted and the resulting crystals were washed sequentially with a small amount of cold toluene then cold HMDSO to yield pale yellow/orange crystals. (16.3 mg, 0.021 mmol, 12.3 % yield). Anal. calculated for C_35_H_76_N_1_P_1_Si_4_Sn_1_: C, 54.38; H, 9.91; N, 1.81. Found: C, 53.19; H, 9.70; N, 1.68. Note: We observed reformation of **1 b** (ca. 10 % by ^31^P{^1^H} NMR spectroscopy) after leaving samples of **5 b** either under a dynamic vacuum for extended periods or upon recrystallisation, which is accompanied by a colour change to red/orange. We were thus unable to obtain samples of **5 b** pure enough to obtain satisfactory elemental analysis results. ^1^H NMR (400 MHz, C_6_D_6_): δ (ppm) 7.45 (d, ^4^ *J*_P−H_=2.5 Hz, 2H; ArC*H*), 5.01 (d, ^1^ *J*_P−H_=208 Hz, 1H; P*H*), 3.25 (m, 1H; N*H*), 1.70 (s, 18H; *o*‐C(C*H*_3_)_3_), 1.34 (s, 9H; *p*‐C(C*H*_3_)_3_), 1.22 (dd, ^2^ *J*_H−H_=11.6 Hz, ^3^ *J*_H−H_=6.1 Hz, 6H; N(H)C(H)(C*H*_3_)_2_), 0.39 (s, 9H; Si(C*H*_3_)_3_), 0.36 (s(br), 18H; Si(C*H*_3_)_3_), 0.18 (s, 9H; Si(C*H*_3_)_3_). Note: SnC*H* protons were observed in 2D spectra but were not assigned in 1D spectra due to overlap with peaks at 0.18 ppm. ^13^C NMR (151 MHz, C_6_D_6_): δ (ppm) 154.37 (d, ^2^ *J*_P−C_=6.5 Hz; *o*‐Ar*C*), 147.72 (*p*‐Ar*C*), 129.77 (d, ^1^ *J*_P−C_=50.5 Hz; *i*‐Ar*C*), 122.20 (s(br); *m*‐Ar*C*), 48.18 (N(H)*C*(H)(CH_3_)_2_), 38.53 (*ortho*‐*C*(CH_3_)_3_), 34.79 (Mes* *para*‐*C*(CH_3_)_3_), 33.74 (d, ^4^ *J*_P−C_=7.0 Hz; Mes* *ortho*‐ C(*C*H_3_)_3_), 31.70 (Mes* *para*‐ C(*C*H_3_)_3_), 30.29 (NC(H)(*C*H_3_)_2_), 29.97 (NC(H)(*C*H_3_)_2_), 8.81 (Sn*C*H), 8.01 (Sn*C*H), 5.22 (Si(*C*H_3_)_3_), 5.14 (Si(*C*H_3_)_3_), 4.98 (Si(*C*H_3_)_3_). ^31^P NMR (162 MHz): δ (ppm) −103.2 (d, ^1^ *J*_P−H_=207 Hz). ^119^Sn NMR (186 MHz, C_6_D_6_): δ (ppm) 40.4 (d, ^1^ *J*_Sn−P_=959 Hz).

Synthesis of [(Me_3_Si)_2_CH]_2_Sn(NHC_6_H_4_OMe)P(H)Mes* (6 b): 1 b (30 mg, 0.042 mmol) and *p*‐anisidine (5.2 mg, 0.042 mmol) were dissolved in toluene (0.5 mL) and heated at 80 °C for 11 hours, causing the colour to fade to yellow/orange. The volatiles were removed *in vacuo* followed by extraction of the solids with pentane (3×0.5 mL). A small amount of hexamethyldisiloxane (0.1 mL) was added. Slow evaporation of the pentane/hexamethyldisiloxane solution at room temperature yielded off‐white crystals which were washed with cold HMDSO and dried *in vacuo* (10.2 mg, 0.012 mmol, 28.6 % yield). Anal. calculated for C_39_H_76_NOPSi_4_Sn: C, 55.96; H, 9.15; N, 1.67. Found: C, 54.89; H, 8.81; N, 1.38. ^1^H NMR (400 MHz, C_6_D_6_): δ (ppm) 7.45 (d, ^4^ *J*_P−H_=2.5 Hz, 2H; ArC*H*), 6.86 (m, 2H; *p*‐anisidine *m‐*C*H*), 6.67–6.62 (m, 2H; *p*‐anisidine *o*‐C*H*), 5.68–4.96 (d, ^1^ *J*_P−H_=207.8 Hz, 1H; P*H*), 3.41 (s, 3H; *p*‐anisidine OC*H*_3_), 3.12 (s, 1H; SnN*H*), 1.62 (s, 18H; *o*‐C(C*H*_3_)_3_), 1.31 (s, 9H; *p*‐C(C*H*_3_)_3_), 0.39 (s, 2H; SnC*H*), 0.33 (s, 9H; Si(C*H*_3_)_3_), 0.28 (s, 18H; Si(C*H*_3_)_3_), 0.21 (s, 9H; Si(C*H*_3_)_3_). ^13^C NMR (151 MHz, C_6_D_6_): δ (ppm) 155.63 (d, ^2^ *J*_P−C_=7.3 Hz; *o*‐Ar*C*), 152.25 (*p*‐anisidine Ar*C*OCH_3_), 148.32 (*p*‐Ar*C*), 145.36 (*p*‐anisidine Ar*C*N), 127.58 (d, ^1^ *J*_P−C_=50.0 Hz; *i*‐Ar*C*), 122.54 (*m*‐Ar*C*), 117.89 (*p*‐anisidine *o‐*Ar*C*), 115.17 (*p*‐anisidine *m‐*Ar*C*), 55.40 (*p*‐anisidine O*C*H_3_), 38.64 (*o*‐*C*(CH_3_)_3_), 34.84 (*p*‐*C*(CH_3_)_3_), 33.80 (d, ^4^ *J*_P−C_=7.2 Hz; *o*‐C(*C*H_3_)_3_), 31.59 (*p*‐C(*C*H_3_)_3_), 10.11 (Sn*C*H), 9.22 (Sn*C*H), 5.06 (m, Si(*C*H_3_)_3_), 4.79 (d, *J*=9.9 Hz, Si(*C*H_3_)_3_). ^31^P NMR (162 MHz, C_6_D_6_): δ (ppm) −117.9 (d, ^1^ *J*_P−H_=207 Hz). ^119^Sn NMR (186 MHz, C_6_D_6_): δ (ppm) 22.4 (d, ^1^ *J*_Sn−P_=1010 Hz).

Attempted synthesis of [(Me_3_Si)_2_CH]_2_Ge(C_3_H_3_N_2_)P(H)Mes* (7 a): Method A (NMR scale): **1 a** (10 mg, 0.015 mmol) was dissolved in C_6_D_6_ in a J. Young NMR tube. Imidazole (1.5 mg, 0.022 mmol) was added and the solution heated at 80 °C for 14 days. The ^31^P NMR spectrum was recorded without further purification, showing almost full conversion to **7 a** in addition to small amount of unreacted **1 a** (<10 %). The volatiles were removed *in vacuo* followed by extraction of the solids with hexane (3×0.5 mL). Crystals suitable for single crystal X‐ray diffraction were grown from a concentrated hexane solution at room temperature, but too few could be isolated for a yield to be recorded. ^31^P NMR (162 MHz, C_6_D_6_): δ (ppm) −89.1 (d, ^1^ *J*_P−H_=219 Hz). Method B (Preparative scale): **1 a** was prepared *in situ* by combining Ge[CH(SiMe_3_)_2_]_2_ (75 mg, 0.192 mmol) and Me_3_P−PMes* (67.5 mg, 0.192 mmol) in toluene (2 mL) and stirring until all of the solids dissolved. The volatiles were removed *in vacuo* and the solid residue redissolved in toluene (2 mL). Imidazole (13 mg, 0.191 mmol) was then added and the solution heated at 80 °C for 14 days. An aliquot (0.5 mL) of the solution revealed the formation of a mixture of products including Mes*P=PMes* and Mes*PH_2_, in addition to small amounts of **7 a** and incomplete consumption of **1 a** by ^31^P{^1^H} NMR spectroscopy.

Attempted synthesis of [(Me_3_Si)_2_CH]_2_Sn(C_3_H_3_N_2_)P(H)Mes* (7 b): Despite repeated attempts, the isolation of a product proposed as **7 b** on the basis of NMR spectroscopy was unsuccessful as it appears to decompose slowly at room temperature and more rapidly at elevated temperatures to form **8**. Method A (NMR scale): **1 b** (10 mg, 0.014 mmol) was combined in an NMR tube with an excess of imidazole (3 mg, 0.044 mmol) and dissolved in C_6_D_6_. The solution immediately decolourised. The ^31^P NMR spectrum of the solution was taken without further purification (Figure S41). Heating the solution at 80 °C for 4 hours results in complete conversion to **8** by ^31^P{^1^H} NMR spectroscopy. ^31^P NMR (162 MHz, C_6_D_6_): δ (ppm) −101.7 (d, ^1^ *J*_P−H_=209 Hz). Method B (Preparative scale): **1 b** (30 mg, 0.042 mmol) was dissolved in toluene (1 mL). 0.5 mL of a freshly prepared imidazole solution in DFB (5.7 mg mL^−1^, 0.042 mmol) was added dropwise and the solution immediately decolourised. Isolation of the product was attempted by removal of the volatiles *in vacuo* followed by extraction of the solids with *n*‐pentane (1 mL×3). A small amount of hexamethyldisiloxane (0.1 mL) was added. Slow evaporation of the pentane/hexamethyldisiloxane solution at room temperature yielded colourless solids which were washed with a small amount of cold HMDSO (Yield: 7.0 mg). The NMR spectra of the solids showed **8** as the primary product along with smaller amounts of **1 b** and Mes*PH_2_.

Synthesis of Mes*P(H)[N(CH)_2_NCH] (8): Me_3_P−PMes* (40 mg, 0.114 mmol) and imidazole (7.8 mg, 0.115 mmol) were combined in toluene and sonicated at room temperature for 30 minutes. Removal of the volatiles *in vacuo* followed by recrystallisation from hexane yielded colourless crystals (19.5 mg, 0.057 mmol, 50 % yield). Anal. calculated for C_21_H_33_N_2_P: C, 73.22; H, 9.66; N, 8.13. Found: C, 72.58; H, 10.18; N, 8.14. ^1^H NMR (400 MHz, C_6_D_6_): δ (ppm) 7.47 (d, ^4^ *J*_P−H_=2.7 Hz, 2H; ArC*H*), 6.91 (d, *J*=238.6 Hz, 1H; P*H*), 6.32–6.29 (m, 1H; NC*H*N(CH)_2_), 1.31 (s, 18H; *o*‐C(C*H*_3_)_3_), 1.23 (s, 9H; *p*‐C(C*H*_3_)_3_). Imidazole C4 and C5 protons were observed in 2D spectra but were not assigned in 1D spectra due to overlap with the residual solvent peak. ^13^C NMR (151 MHz, C_6_D_6_): δ (ppm) 156.49 (d, ^2^ *J*_P−C_=8.8 Hz; *o*‐Ar*C*), 152.26 (*p*‐Ar*C*), 141.62 (d, ^2^ *J*_P−C_=11.3 Hz; imidazole *C*5), 131.31 (d, ^3^ *J*_P−C_=3.3 Hz; imidazole *C*4), 127.54 (d, ^1^ *J*_P−C_=27.0 Hz; *i*‐Ar*C*), 123.08 (br; *m*‐Ar*C*), 122.07 (d, ^2^ *J*_P−C_=7.9 Hz; imidazole *C*2), 38.45 (br; *o*‐*C*(CH_3_)_3_), 35.11 (*p*‐*C*(CH_3_)_3_), 33.57 (d, ^4^ *J*_P−C_=7.3 Hz; *o*‐C(*C*H_3_)_3_), 31.23 (*p*‐C(*C*H_3_)_3_). ^31^P NMR (162 MHz, C_6_D_6_): δ (ppm) −8.0 (d, ^1^ *J*_P−H_=239 Hz).

## Supporting Information

Additional references cited in the Supporting Information.[^41^, ^42^, ^43^, ^44^, ^45^, ^46^, ^47^, ^48^, ^49^, ^50^, ^51^, ^52^, ^53^, ^54^, ^55^]

## Conflict of interest

The authors declare no conflict of interest.

1

## Supporting information

As a service to our authors and readers, this journal provides supporting information supplied by the authors. Such materials are peer reviewed and may be re‐organized for online delivery, but are not copy‐edited or typeset. Technical support issues arising from supporting information (other than missing files) should be addressed to the authors.

Supporting Information

## Data Availability

The data that support the findings of this study are available in the supplementary material of this article.

## References

[1] M. F. Lappert , P. J. Davidson , J. Chem. Soc. Chem. Commun. 1973, 317.

[2] D. E. Goldberg , D. H. Harris , M. F. Lappert , K. M. Thomas , J. Chem. Soc. Chem. Commun. 1976, 261–262.

[3] R. West , M. J. Fink , J. Michl , Science 1981, 214, 1343–1344. 17812259 10.1126/science.214.4527.1343

[4] M. Yoshifuji , I. Shima , N. Inamoto , K. Hirotsu , T. Higuchi , J. Am. Chem. Soc. 1981, 103, 4587–4589.

[5] J. D. Guo , D. J. Liptrot , S. Nagase , P. P. Power , Chem. Sci. 2015, 6, 6235–6244. 30090241 10.1039/c5sc02707a PMC6054042

[6] R. Sedlak , O. A. Stasyuk , C. Fonseca Guerra , J. Řezáč , A. Růžička , P. Hobza , J. Chem. Theory Comput. 2016, 12, 1696–1704. 26953594 10.1021/acs.jctc.6b00065

[7] For a recent review article see: C. Weetman , Chem. Eur. J. 2021, 27, 1941–1954.32757381

[8] R. C. Fischer , P. P. Power , Chem. Rev. 2010, 110, 3877–3923. 20672858 10.1021/cr100133q

[9] V. Ya Lee , A. Sekiguchi , Organometallics 2004, 23, 2822–2834.

[10] N. J. Hardman , C. Cui , H. W. Roesky , W. H. Fink , P. P. Power , Angew. Chem. Int. Ed. 2001, 40, 2172–2174. 10.1002/1521-3773(20010601)40:11<2172::AID-ANIE2172>3.0.CO;2-Y 29712210

[11] Further examples of E=N double bonds (E=Al, Ga, In):

[11a] R. J. Wright , A. D. Phillips , T. L. Allen , W. H. Fink , P. P. Power , J. Am. Chem. Soc. 2003, 125, 1694–1695; 12580583 10.1021/ja029422u

[11b] R. J. Wright , M. Brynda , J. C. Fettinger , A. R. Betzer , P. P. Power , J. Am. Chem. Soc. 2006, 128, 12498–12509; 16984201 10.1021/ja063072k

[11c] J. Li , X. Li , W. Huang , H. Hu , J. Zhang , C. Cui , Chem. Eur. J. 2012, 18, 15263–15266; 23129126 10.1002/chem.201203298

[11d] A. Heilmann , J. Hicks , P. Vasko , J. M. Goicoechea , S. Aldridge , Angew. Chem. Int. Ed. 2020, 59, 4897–4901; 10.1002/anie.201916073 31999037

[11e] M. D. Anker , R. J. Schwamm , M. P. Coles , Chem. Commun. 2020, 56, 2288–2291; 10.1039/c9cc09214e 31984981

[11f] J. D. Queen , S. Irvankoski , J. C. Fettinger , H. M. Tuononen , P. P. Power , J. Am. Chem. Soc. 2021, 143, 6351–6356. 33882237 10.1021/jacs.1c02463 PMC8154528

[12a] D. W. N. Wilson , J. Feld , J. M. Goicoechea , Angew. Chem. Int. Ed. 2020, 59, 20914–20918; 10.1002/anie.202008207 PMC7693089 32615007

[12b] D. W. N. Wilson , W. Myers , J. M. Goicoechea , Dalton Trans. 2020, 49, 15249–15255. 33084675 10.1039/d0dt03174g

[13] M. K. Sharma , C. Wölper , G. Haberhauer , S. Schulz , Angew. Chem. Int. Ed. 2021, 60, 6784–6790. 10.1002/anie.202014381 PMC7986129 33368922

[14a] M. Fischer , S. Nees , T. Kupfer , J. T. Goettel , H. Braunschweig , C. Hering-Junghans , J. Am. Chem. Soc. 2021, 143, 4106–4111; 33691065 10.1021/jacs.1c00204

[14b] T. Taeufer , F. Dankert , D. Michalik , J. Pospech , J. Bresien , C. Hering-Junghans , Chem. Sci. 2023, 14, 3018–3023. 36937589 10.1039/d2sc06292e PMC10016425

[15] J. Feld , D. W. N. Wilson , J. M. Goicoechea , Angew. Chem. Int. Ed. 2021, 60, 22057–22061. 10.1002/anie.202109334 PMC8518045 34383991

[16a] M. K. Sharma , C. Wölper , G. Haberhauer , S. Schulz , Angew. Chem. Int. Ed. 2021, 60, 21784–21788; 10.1002/anie.202108370 PMC8519123 34324782

[16b] M. K. Sharma , C. Wölper , S. Schulz , Dalton Trans. 2022, 51, 1612–1616. 34994365 10.1039/d1dt04299h

[17] S. Nees , T. Wellnitz , F. Dankert , M. Härterich , S. Dotzauer , M. Feldt , H. Braunschweig , C. Hering-Junghans , Angew. Chem. Int. Ed. 2023, 62, e202215838. 10.1002/anie.202215838 36516342

[18a] M. Dräger , J. Escudié , C. Couret , H. Ranaivonjatovo , J. Stage , Organometallics 1988, 7, 1010–1013;

[18b] H. Ranaivonjatovo , J. Escudié , C. Couret , J. Satgé , J. Organomet. Chem. 1991, 415, 327–333;

[18c] V. Ya Lee , M. Kawai , A. Sekiguchi , H. Ranaivonjatovo , J. Escudié , Organometallics 2009, 28, 4262–4265.

[19] H. R. G. Bender , E. Niecke , M. Nieger , J. Am. Chem. Soc. 1993, 115, 3314–3315.

[20] V. Nesterov , N. C. Breit , S. Inoue , Chem. Eur. J. 2017, 23, 12014–12039. 28379639 10.1002/chem.201700829

[21a] C. Couret , J. Escudié , J. Satgé , A. Raharinirina , J. D. Andriamizaka , J. Am. Chem. Soc. 1985, 107, 8280–8281;

[21b] H. Ranaivonjatovo , J. Escudié , C. Couret , J. Satgé , J. Chem. Soc. Chem. Commun. 1992, 1047–1048.

[22] V. Nesterov , R. Baierl , F. Hanusch , A. E. Ferao , S. Inoue , J. Am. Chem. Soc. 2019, 141, 14576–14580. 31476856 10.1021/jacs.9b08741

[23] M. Fischer , M. M. D. Roy , L. L. Wales , M. A. Ellwanger , A. Heilmann , S. Aldridge , J. Am. Chem. Soc. 2022, 144, 8908–8913. 35536684 10.1021/jacs.2c03302 PMC9136930

[24] M. Zweigart , C. Wenzel , K. Eichele , H. Schubert , L. Wesemann , Angew. Chem. Int. Ed. 2023, 62, e202304200. 10.1002/anie.202304200 37186011

[25] M. Fischer , M. M. D. Roy , L. L. Wales , M. A. Ellwanger , C. McManus , A. F. Roper , A. Heilmann , S. Aldridge , Angew. Chem. Int. Ed. 2022, 134, e202211616. 10.1002/anie.202211616 PMC9828258 36161749

[26] P. Gupta , J.-E. Siewert , T. Wellnitz , M. Fischer , W. Baumann , T. Beweries , C. Hering-Junghans , Dalton Trans. 2021, 50, 1838–1844. 33471018 10.1039/d1dt00071c

[27] For early studies on phospha-Wittig reagents see:

[27a] A. Marinetti , F. Mathey , Angew. Chem. Int. Ed. Engl. 1988, 27, 1382–138;

[27b] S. Shah , J. D. Protasiewicz , Chem. Commun. 1998, 1585–1586;

[27c] E. Urnėžius , S. Shah , J. D. Protasiewicz , Phosphorus Sulfur Silicon Relat. Elem. 1999, 144(4), 137–139;

[27d] S. Shah , M. C. Simpson , R. C. Smith , J. D. Protasiewicz , J. Am. Chem. Soc. 2001, 123, 6925–6926; 11448199 10.1021/ja015767l

[27e] J. D. Protasiewicz , Eur. J. Inorg. Chem. 2012, 2012, 4539–4549;

[27f] K. Takeuchi , H.-o. Taguchi , I. Tanigawa , S. Tsujimoto , T. Matsuo , H. Tanaka , K. Yoshizawa , F. Ozawa , Angew. Chem. Int. Ed. 2016, 55, 15347–15350. 10.1002/anie.201609515 27860032

[28] Deposition Numbers 2263036 (for **1 a**), 2263037 (for **1 b**), 2263038 (for **2 a**⋅C_6_D_6_), 2263039 (for **2 b**), 2263040 (for **3 a**), 2263041 (for **3 b**), 2263042 (**4 a**⋅C_6_D_6_), 2263043 (**4 b**⋅C_6_D_6_), 2263044 (**5 b**), 2263045 (**6 b**), 2263046 (**7 a**), 2263047 (**8**) contain the supplementary crystallographic data for this paper. These data are provided free of charge by the joint Cambridge Crystallographic Data Centre and Fachinformationszentrum Karlsruhe Access Structures service.

[29] P. Pyykkö , M. Atsumi , Chem. Eur. J. 2009, 15, 12770–12779. 19856342 10.1002/chem.200901472

[30] G. H. Spikes , J. C. Fettinger , P. P. Power , J. Am. Chem. Soc. 2005, 127(35), 12232–12233. 16131195 10.1021/ja053247a

[31] M. Arrowsmith , J. Böhnke , H. Braunschweig , M. A. Celik , T. Dellermann , K. Hammond , Chem. Eur. J. 2016, 21, 17169–17172. 10.1002/chem.201604094 27685839

[32] C. Weetman , A. Porzelt , P. Bag , F. Hanusch , S. Inoue , Chem. Sci. 2020, 11, 4817–4827. 34122939 10.1039/d0sc01561j PMC8159210

[33] J. Escudié , C. Couret , M. Andrianarson , A. Raharinirina , J. Satgé , Phosphorus Sulfur Relat. Elem. 1987, 30, 377–380.

[34] F. Dankert , J.-E. Siewert , P. Gupta , F. Weigend , C. Hering-Junghans , Angew. Chem. Int. Ed. 2022, 61, e202207064. 10.1002/anie.202207064 PMC9400956 35594171

[35] Y. Masaaki , S. Takahiro , I. Naoki , Chem. Lett. 1988, 17, 1735–1738.

[36] J. D. Masuda , A. J. Hoskin , T. W. Graham , C. Beddie , M. C. Fermin , N. Etkin , D. W. Stephan , Chem. Eur. J. 2006, 12, 8696–8707. 16952126 10.1002/chem.200600429

[37] M. Fischer , F. Reiß , C. Hering-Junghans , Chem. Commun. 2021, 57, 5626–5629. 10.1039/d1cc01305j 33989372

[38] A. J. Roering , S. N. MacMillan , J. M. Tanski , R. Waterman , Inorg. Chem. 2007, 46, 6855–6857. 17649993 10.1021/ic7013144

[39] L. Klemmer , A.-L. Thömmes , M. Zimmer , V. Huch , B. Morgenstern , D. Scheschkewitz , Nat. Chem. 2021, 13, 373–377. 33649497 10.1038/s41557-021-00639-9

[40] N. Kuhn , T. Kratz , Synthesis 1993, 6, 561–562.

[41] *CrysAlisPro*, Agilent Technologies, Version 1.171.41.117a.

[42a] G. M. Sheldrick, *SHELXL97, Programs for Crystal Structure Analysis (Release 97–2)*, Institut für Anorganische Chemie der Universität, Tammanstrasse 4, D-3400 Göttingen, Germany **1998**;

[42b] G. M. Sheldrick , Acta Crystallogr. Sect. A 1990, 46, 467–473;

[42c] G. M. Sheldrick , Acta Crystallogr. Sect. A 2008, 64, 112–122. 18156677 10.1107/S0108767307043930

[43] F. Neese , F. Wennmohs , U. Becker , C. Riplinger , J. Chem. Phys. 2020, 152, 224108. 32534543 10.1063/5.0004608

[44] F. Neese , Wiley Interdiscip. Rev.: Comput. Mol. Sci. 2018, 8, 1–6. 10.1002/wcms.1370 PMC6220962 30450129

[45] F. Neese , Wiley Interdiscip. Rev.: Comput. Mol. Sci. 2012, 2, 73–78.

[46] R. Izsák , F. Neese , J. Chem. Phys. 2011, 135, 144105. 22010696 10.1063/1.3646921

[47] F. Neese , F. Wennmohs , A. Hansen , U. Becker , Chem. Phys. 2009, 356, 98–109.

[48] M. Bühl , C. Reimann , D. A. Pantazis , T. Bredow , F. Neese , J. Chem. Theory Comput. 2008, 4, 1449–1459. 26621431 10.1021/ct800172j

[49] D. A. Pantazis , X. Y. Chen , C. R. Landis , F. Neese , J. Chem. Theory Comput. 2008, 4, 908–919. 26621232 10.1021/ct800047t

[50] Y. S. Lin , G. De Li , S. P. Mao , J. Da Chai , J. Chem. Theory Comput. 2013, 9, 263–272. 26589028 10.1021/ct300715s

[51] M. Bühl , C. Reimann , D. A. Pantazis , T. Bredow , F. Neese , J. Chem. Theory Comput. 2008, 4, 1449–1459. 26621431 10.1021/ct800172j

[52] D. A. Pantazis , X. Y. Chen , C. R. Landis , F. Neese , J. Chem. Theory Comput. 2008, 4, 908–919. 26621232 10.1021/ct800047t

[53] J. Baker , J. Comput. Chem. 1986, 7, 385–395.

[54] N. Harvey , M. Aschi , H. Schwarz , W. Koch , Theor. Chem. Acc. 1998, 99, 95–99.

[55] E. D. Glendening, J. K. Badenhoop, A. E. Reed, J. E. Carpenter, J. A. Bohmann, C. M. Morales, P. Karafiloglou, C. R. Landis, F. Weinhold, *NBO 7.0* **2018**.

