# Supplementary material for: Hydroelementation and Phosphinidene Transfer: Reactivity of Phosphagermenes and Phosphastannenes Towards Small Molecule Substrates
Source: Chemistry. 2023 Oct 9;29(68):e202301542. doi: 10.1002/chem.202301542 (PMC10946619; doi:10.1002/chem.202301542)
Supplement: Supplementary file 1 — Supporting Information [file CHEM-29-0-s001.pdf]

Chemistry–A European Journal

Supporting Information

Hydroelementation and Phosphinidene Transfer: Reactivity of Phosphagermenes and Phosphastannenes Towards Small Molecule Substrates

Matthew J. Reveley, Joey Feld, Diana Temerova, Eric S. Yang, and Jose M. Goicoechea*

CONTENTS

1. Experimental Section 3

1.1 Synthesis of novel compounds 3

1.1.1. Synthesis of $[(Me_3Si)_2CH]_2Ge{=}PMes^*$ (**1a**) 3

1.1.2. Synthesis of $[(Me_3Si)_2CH]_2Sn{=}PMes^*$ (**1b**) 7

1.1.3. Synthesis of $[(Me_3Si)_2CH]_2Ge(NH_2)P(H)Mes^*$ (**2a**) 11

1.1.4. Synthesis of $[(Me_3Si)_2CH]_2Ge(NH^nPr)P(H)Mes^*$ (**3a**) 14

1.1.5. Synthesis of $[(Me_3Si)_2CH]_2Ge(OH)P(H)Mes^*$ (**4a**) 17

1.1.6. Synthesis of $[(Me_3Si)_2CH]_2Sn(NH_2)P(H)Mes^*$ (**2b**) 20

1.1.7. Synthesis of $[(Me_3Si)_2CH]_2Sn(NH^nPr)P(H)Mes^*$ (**3b**) 23

1.1.8. Synthesis of $[(Me_3Si)_2CH]_2Sn(OH)P(H)Mes^*$ (**4b**) 26

1.1.9. Synthesis of $[(Me_3Si)_2CH]_2Sn(NH^iPr)P(H)Mes^*$ (**5b**) 29

1.1.10 Synthesis of $[(Me_3Si)_2CH]_2Sn(NHC_6H_4OMe)P(H)Mes^*$ (**6b**) 32

1.1.11. Attempted synthesis of $[(Me_3Si)_2CH]_2Ge(C_3H_3N_2)P(H)Mes^*$ (**7a**) 35

1.1.12. Attempted synthesis of $[(Me_3Si)_2CH]_2Sn(C_3H_3N_2)P(H)Mes^*$ (**7b**) 37

1.1.13. Synthesis of $Mes^*P(H)[N(CH)_2NCH]$ (**8**) 41

1.1.14. The reaction between **1b** and $PhSiH_3$ 43

1.1.15. Generation of PMes* from thermolysis or photolysis of **1b** 43

1.1.16. Generation of PMes* from photolysis of **1a** 45

1.1.17. Reaction of **1b** with IMe_4 to form $Mes^*P–IMe_4$ 47

1.1.18. Metathesis of **1b** with $Ge[CH(SiMe_3)_2]_2$ to give **1a** 47

1.2. Mechanistic investigations into the formation of **7b** and **8** 49

1.2.1. The reaction of **1b** with sub-stoichiometric amounts of imidazole in DFB 51

1.2.2. The reaction between $Sn[CH(SiMe_3)_2]_2$ and imidazole 52

1.2.3. The reaction of **8** and $Sn[CH(SiMe_3)_2]_2$ 53

1.2.4. The reaction of **8** and $Ge[CH(SiMe_3)_2]_2$ 55

1.2.5. The reaction of $Mes^*P(H)(NH^iPr)$ and $Sn[CH(SiMe_3)_2]_2$ 56

1.2.6. The reaction between **5b** and imidazole 58

2. Single crystal X-ray diffraction data 60

3. Computational details 72
3.1. General computational methods 72
3.2. Electronic structure calculations 73
3.3. Computed mechanisms 74
3.4. XYZ coordinates 75
4. References 88

1. Experimental Section

1.1 Synthesis of novel compounds

1.1.1. Synthesis of $[(Me_3Si)_2CH]_2Ge{=}PMes^*$ (1a)

$Ge[CH(SiMe_3)_2]_2$ (100 mg, 0.255 mmol) and $Me_3P{-}PMes^*$ (89.9 mg, 0.255 mmol) were stirred for an hour in toluene until all the solids were dissolved. All volatiles were removed under vacuum and the product was recrystallised from hexane to yield yellow crystals (94 mg, 0.14 mmol, 54.9% yield). Crystals suitable for X-ray diffraction were grown by slow evaporation of a hexane solution at room temperature. Anal. calculated for $C_{32}H_{67}Ge_1P_1Si_4$: C, 57.55; H, 10.11; N, 0. Found: C, 57.38; H, 10.15; N, 0.

^{1}H NMR (400 MHz, C_6D_6): δ (ppm) 7.49 (s, 2H; Mes* ArC*H*), 1.79 (s, 18H; Mes* *ortho*-ArC(CH_3)$_3$), 1.44 (s, 1H, GeC*H*), 1.38 (s, 9H; Mes* *para*-ArC(CH_3)$_3$), 0.93 (d, 1H, $^{3}J_{P–H}$ = 15.4 Hz; GeC*H*) 0.43 (s, 18H; Si(CH_3)$_3$), 0.05 (s, 18H; Si(CH_3)$_3$).

^{13}C NMR (101 MHz, C_6D_6): δ (ppm) 154.75 (Mes* *ortho*-Ar*C*), 147.99 (Mes* *para*-Ar*C*), 136.04 (Mes* *ipso*-Ar*C*), 121.79 (Mes* *meta*-Ar*C*), 39.12 (Mes* *ortho*-*C*(CH$_3$)$_3$), 34.85 (Mes* *para*-*C*(CH$_3$)$_3$), 33.84 (d, $^{4}J_{P–C}$ = 7.72 Hz; Mes* *ortho*-C(*C*H$_3$)$_3$), 31.81 (Mes* *para*-C(*C*H$_3$)$_3$), 26.36 (d, $^{2}J_{P–C}$ = 23.1 Hz; Ge*C*H), 24.07 (d, $^{2}J_{P–C}$ = 14.2 Hz; Ge*C*H), 3.99 (d, $^{4}J_{P–C}$ = 4.0 Hz; Si(*C*H$_3$)$_3$), 3.83 (Si(*C*H$_3$)$_3$).

^{31}P NMR (162 MHz, C_6D_6): δ (ppm) 171.5 (s).

UV-vis: λ_{max} (nm) 323, 358 (shoulder).

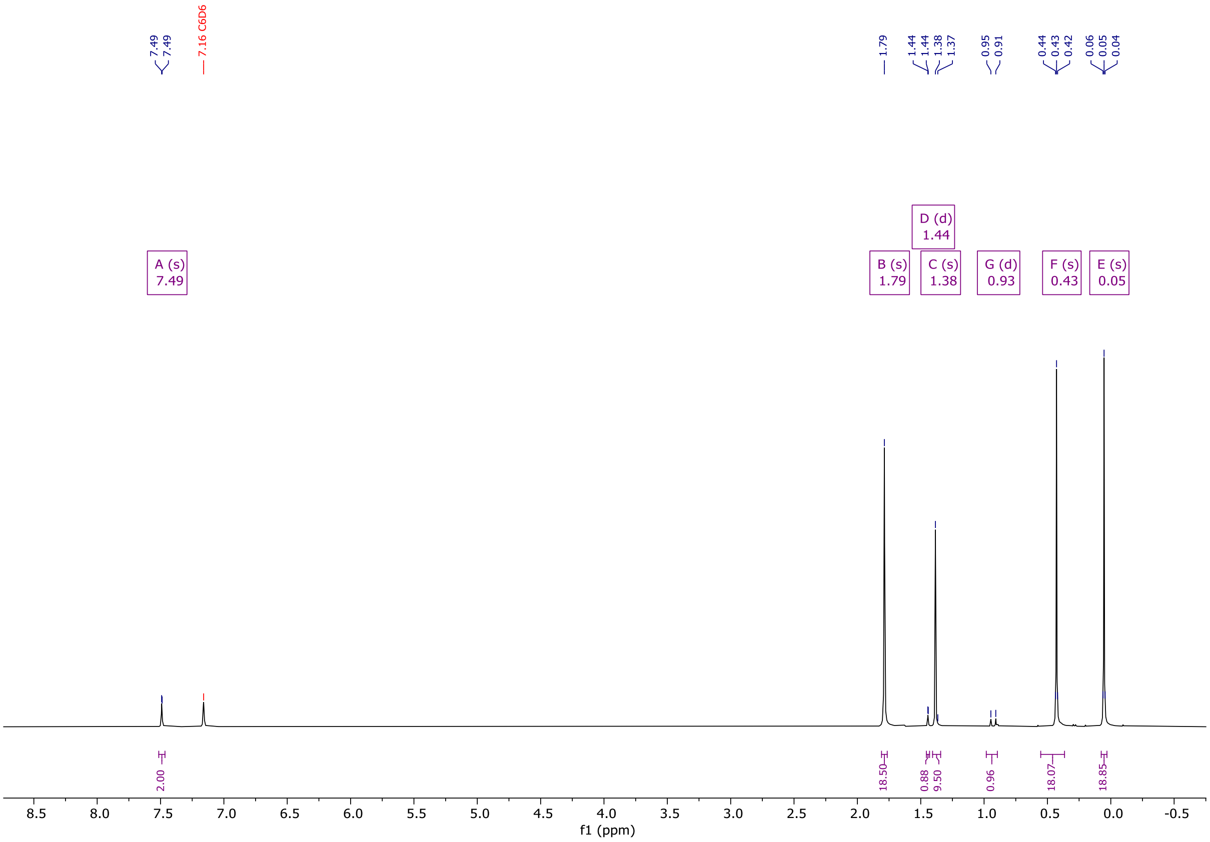

Figure S1. ^{1}H NMR spectrum (400 MHz, 293 K) of **1a** in C_6D_6.

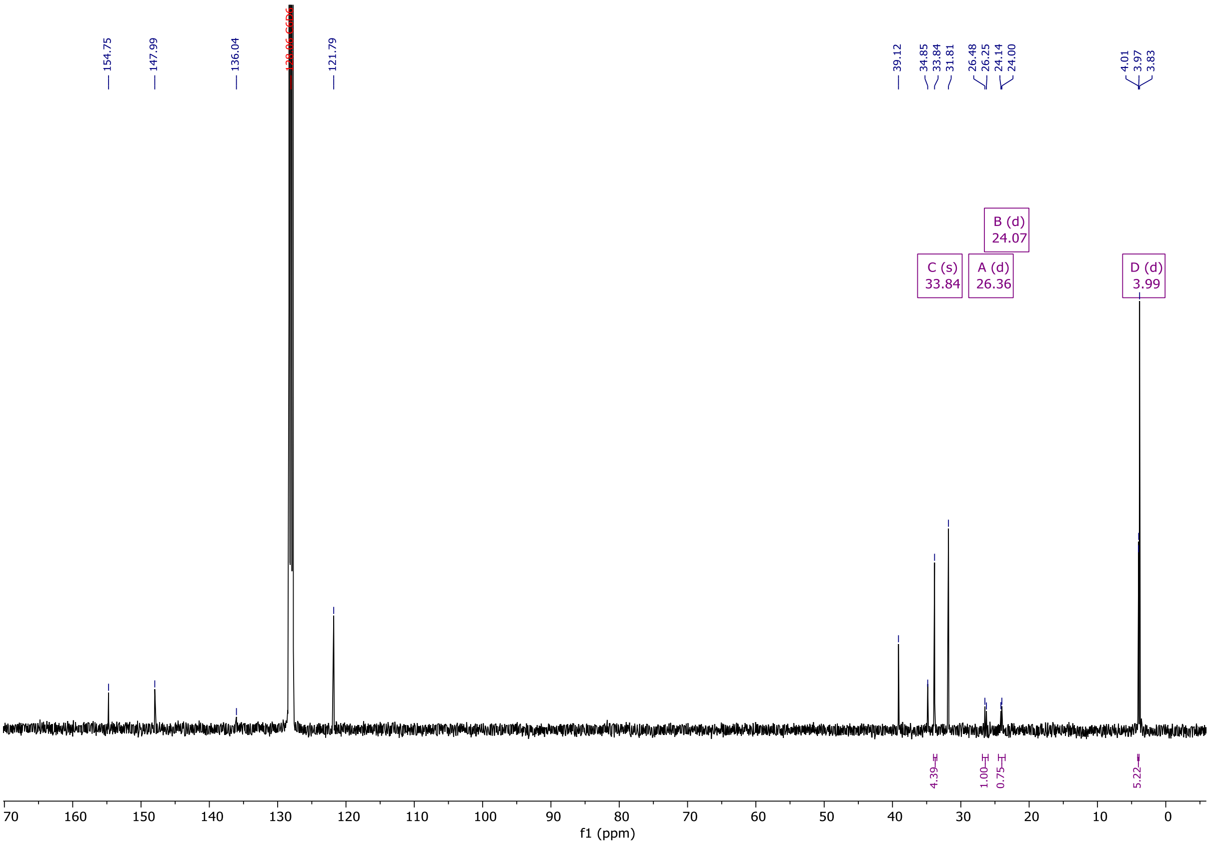

Figure S2. ^{13}C NMR spectrum (101 MHz, 293 K) of **1a** in C_6D_6.

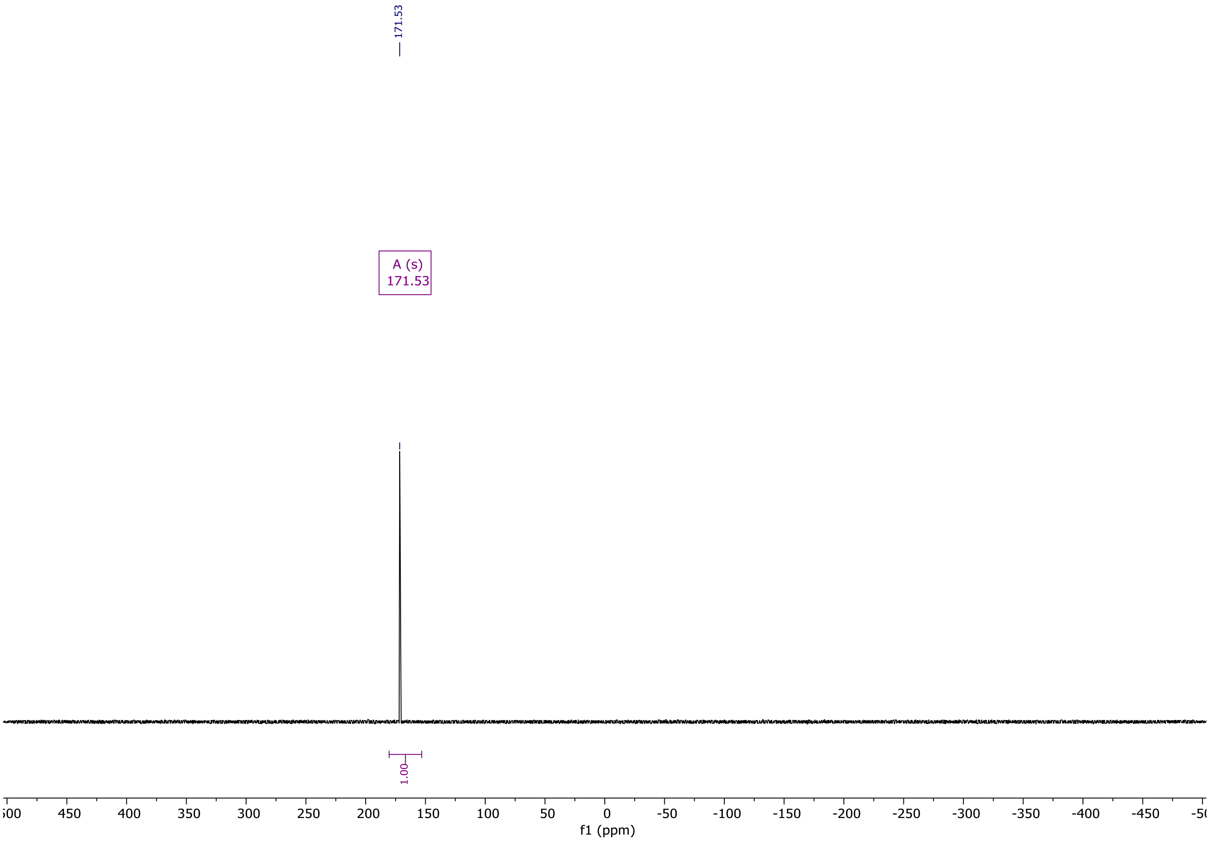

Figure S3. ^{31}P NMR spectrum (162 MHz, 293 K) of **1a** in C_6D_6.

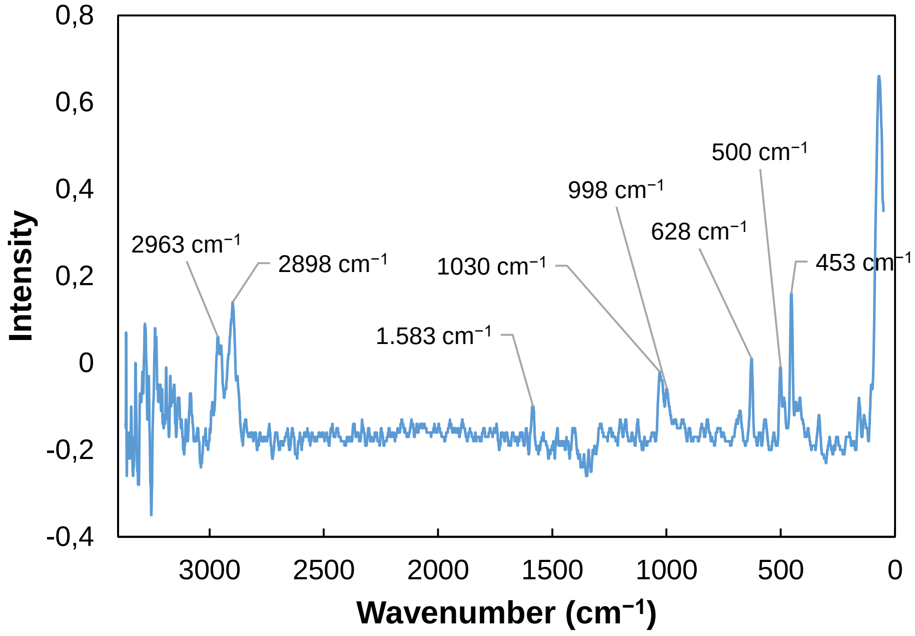

Figure S4. Dispersive Raman spectrum of **1a**.

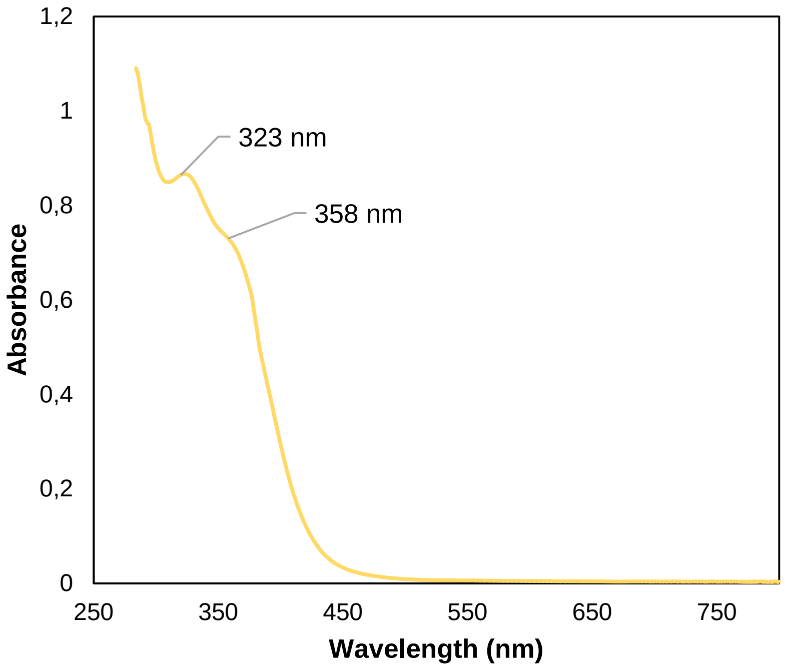

Figure S5. UV-visible absorption spectrum of **1a** in toluene (0.18 mg/mL).

1.1.2. Synthesis of $[(Me_3Si)_2CH]_2Sn{=}PMes^*$ (1b)

$Sn[CH(SiMe_3)_2]_2$ (100 mg, 0.229 mmol) and Me_3P–PMes* (81 mg, 0.23 mmol) were stirred in toluene (5 mL) until all the solids were dissolved. All volatiles were removed under vacuum and the product was recrystallised from hexane/hexamethyldisiloxane to yield red crystals. (54.4 mg, 0.076 mmol, 33.2 yield). Crystals suitable for X-ray diffraction were grown by slow evaporation of a hexane solution at room temperature. Anal. calculated for $C_{32}H_{67}P_1Sn_1Si_4$: C, 53.84; H, 9.46; N, 0.00. Found: C, 53.78; H, 9.50; N, 0.00.

^{1}H NMR (400 MHz, C_6D_6): δ (ppm) 7.50 (s, 2H; Mes* ArC*H*), 1.82 (s, 18H; Mes* *ortho*-C(C*H*$_3$)$_3$), 1.38 (s, 9H; Mes* *para*-C(C*H*$_3$)$_3$), 1.14 (s, 1H; SnC*H*), 0.74 (d, $^3J_{P–H}$ = 7.9 Hz, 1H; SnC*H*), 0.41 (s, 18H; Si(C*H*$_3$)$_3$), 0.03 (s, 18H; Si(C*H*$_3$)$_3$).

^{13}C NMR (151 MHz, C_6D_6): δ (ppm) 154.68 (Mes* *ortho*-Ar*C*), 148.25 (Mes* *para*-Ar*C*), 135.50 (d, $^1J_{P–C}$ = 82.9 Hz; Mes* *ipso*-Ar*C*), 121.50 (Mes* *meta*-Ar*C*), 39.18 (Mes* *ortho*-*C*(CH$_3$)$_3$), 34.83 (Mes* *para*-*C*(CH$_3$)$_3$), 34.38 (d, $^2J_{P–C}$ = 22.6 Hz; Sn*C*H), 33.89 (d, $^4J_{P–C}$ = 7.8 Hz; Mes* *ortho*-C(*C*H$_3$)$_3$), 31.96 (Mes* *para*-C(*C*H$_3$)$_3$), 25.31 (Sn*C*H), 4.34 (d, $^4J_{P–C}$ = 3.5 Hz; Si(*C*H$_3$)$_3$), 4.07 (Si(*C*H$_3$)$_3$).

^{31}P NMR (162 MHz, C_6D_6): δ (ppm) 202.8 (s).

^{119}Sn NMR (186 MHz, C_6D_6): δ (ppm) 660.4 (d, $^1J_{Sn–P}$ = 2292 Hz).

UV-vis: λ_{max} (nm) 412.

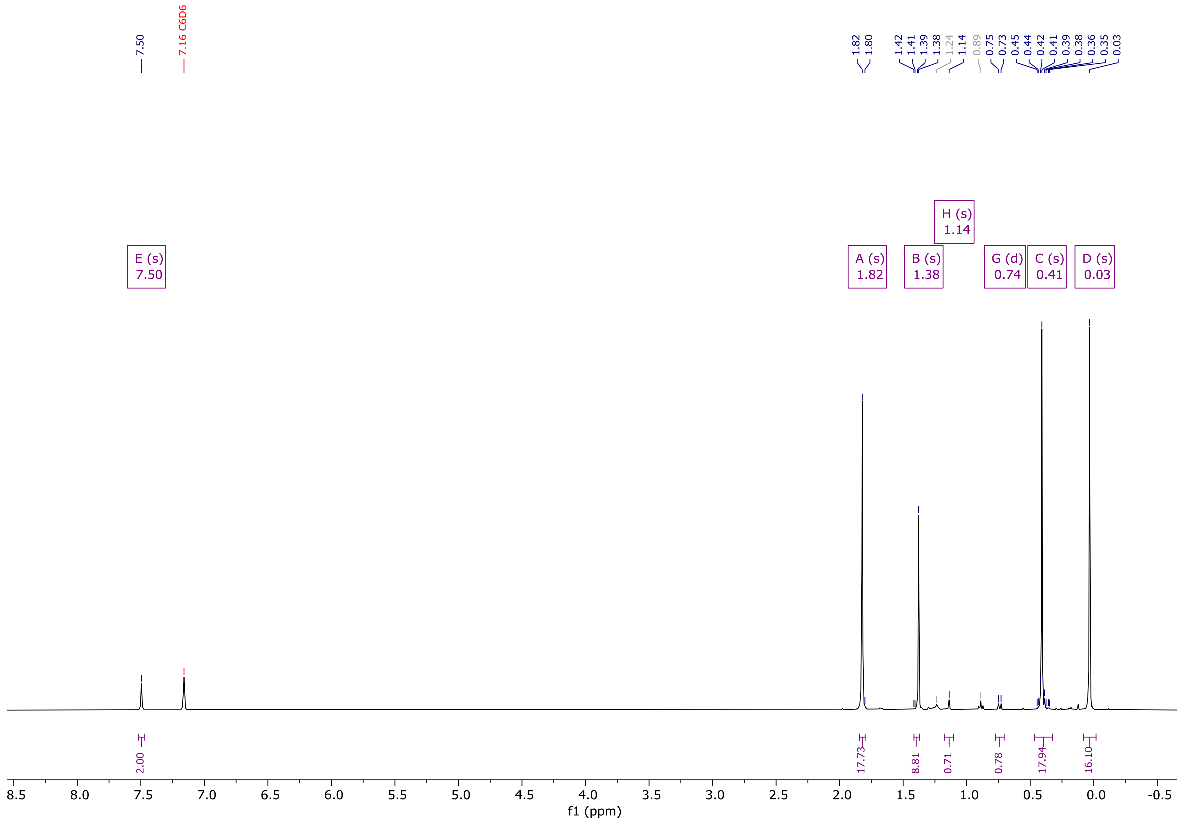

Figure S6. ^{1}H NMR spectrum (400 MHz, 293 K) of **1b** in C_6D_6.

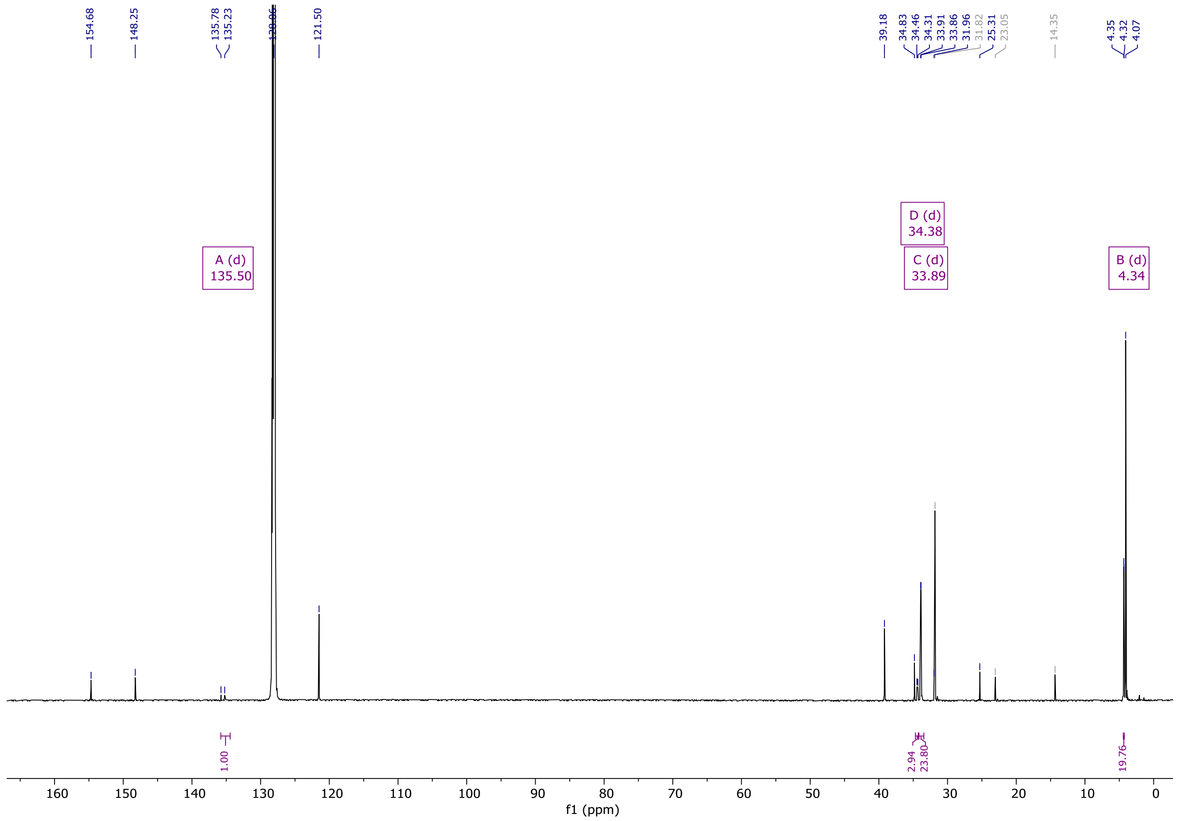

Figure S7. ^{13}C NMR spectrum (151 MHz, 293 K) of **1b** in C_6D_6.

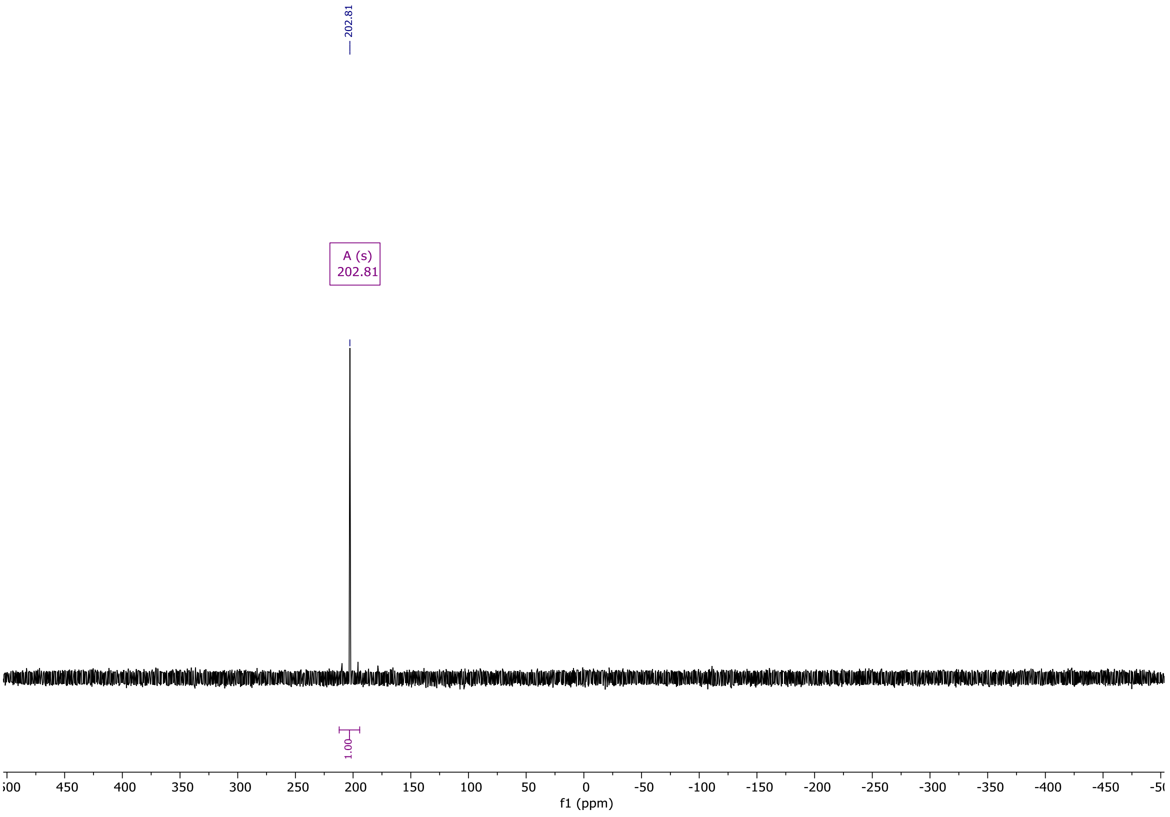

Figure S8. ^{31}P NMR spectrum (162 MHz, 293 K) of **1b** in C_6D_6.

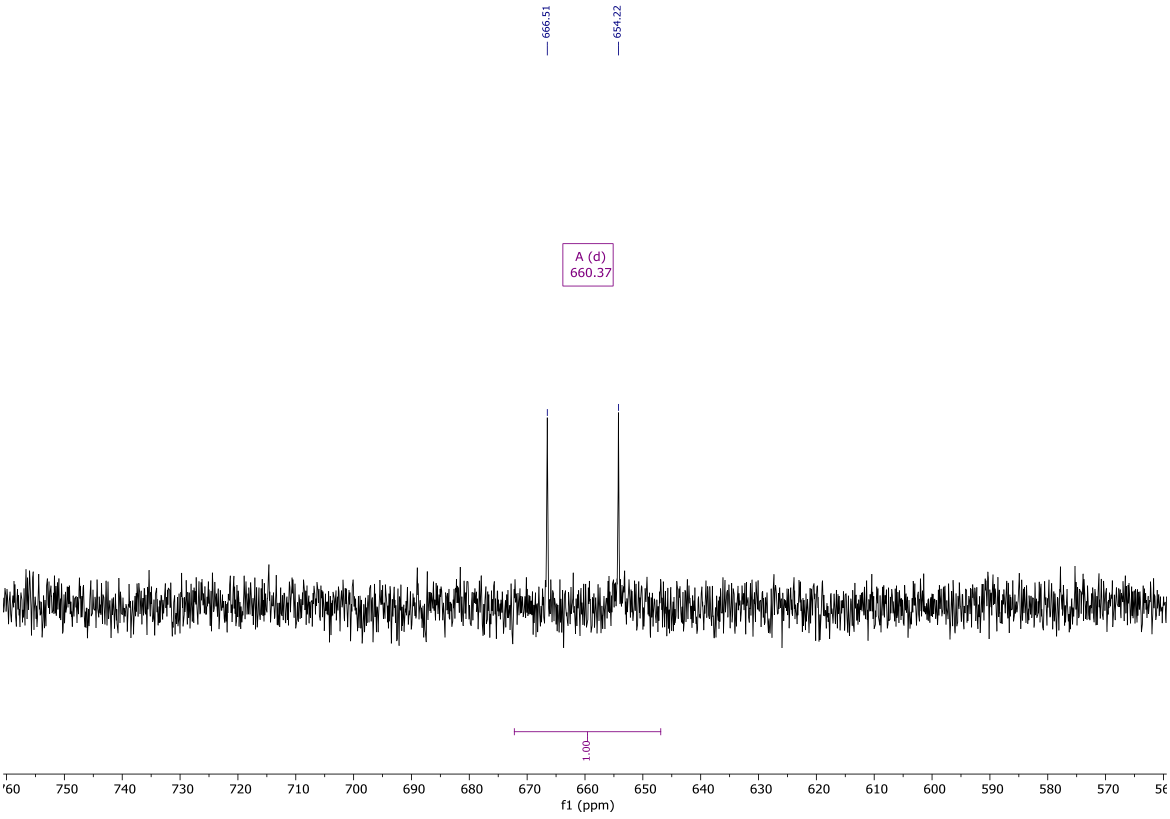

Figure S9. ^{119}Sn NMR spectrum (186 MHz, 293 K) of **1b** in C_6D_6.

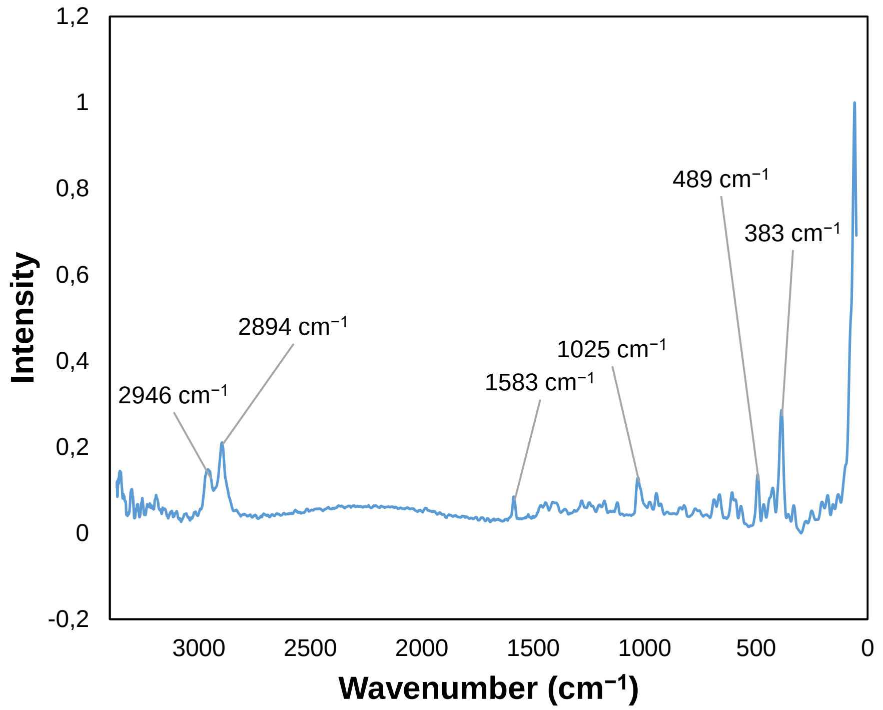

Figure S10. Dispersive Raman spectrum of **1b**.

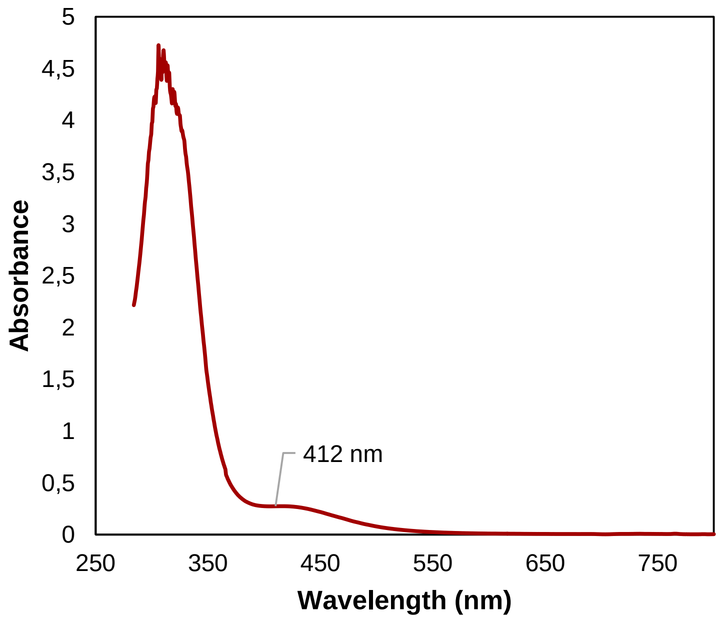

Figure S11. UV-visible absorption spectrum of **1b** in toluene (0.13 mg/mL).

1.1.3. Synthesis of $[(Me_3Si)_2CH]_2Ge(NH_2)P(H)Mes^*$ (2a)

1b (30 mg, 0.045 mmol) was dissolved in toluene in an NMR tube and put under 1 bar of NH_3. The tube was heated to 80 °C for 22 hours, becoming almost colourless. The volatiles were removed *in vacuo* and the solids extracted with hexane (0.5 mL × 3). A small amount of hexamethyldisiloxane (0.1 mL) was added. Slow evaporation of the hexane/hexamethyldisiloxane solution at room temperature yielded colourless crystals which were washed with cold HMDSO and dried *in vacuo* (14.1 mg, 0.021 mmol, 45.8% yield). Crystals suitable for X-ray diffraction were grown from standing of a concentrated C_6D_6 solution at room temperature. Anal. calculated for $C_{32}H_{70}Ge_1N_1P_1Si_4$: C, 56.12; H, 10.30; N, 2.05. Found: C, 55.88; H, 10.51; N, 1.74.

^{1}H NMR (400 MHz, C_6D_6): δ (ppm) 7.48 (d, $^4J_{P–H}$ = 2.4 Hz, 2H; Mes* ArC*H*), 4.83 (d, $^1J_{P–H}$ = 213.6 Hz, 1H; P*H*), 1.68 (s, 18H; Mes* *ortho*-C(CH_3)$_3$), 1.33 (s, 9H; Mes* *para*-C(CH_3)$_3$), 0.53 (s, 2H; GeNH_2), 0.36 (s(br), 18H; Si(CH_3)$_3$), 0.21 (s, 18H; Si(CH_3)$_3$). Note: GeC*H* protons were observed in 2D spectra but were not assigned in 1D spectra due to overlap with peaks at 0.36 ppm.

^{13}C NMR (162 MHz, C_6D_6): δ (ppm) 155.71 (Mes* *ortho*-Ar*C*), 148.19 (Mes* *para*-Ar*C*), 129.62 (d, $^1J_{P–C}$ = 49.4 Hz; Mes* *ipso*-Ar*C*), 122.61 (d, $^3J_{P–C}$ = 3.3 Hz; Mes* *meta*-Ar*C*), 38.83 (Mes* *ortho*-*C*(CH$_3$)$_3$), 34.85 (Mes* *para*-*C*(CH$_3$)$_3$), 34.21 (Mes* *ortho*-C(*C*H$_3$)$_3$), 31.57 (Mes* *para*-C(*C*H$_3$)$_3$), 12.36 (Ge*C*H), 11.48 (Ge*C*H), 4.69 (Si(*C*H$_3$)$_3$), 4.66 (Si(*C*H$_3$)$_3$).

^{31}P NMR (162 MHz, C_6D_6): δ (ppm) –95.4 (d, $^1J_{P–H}$ = 214 Hz).

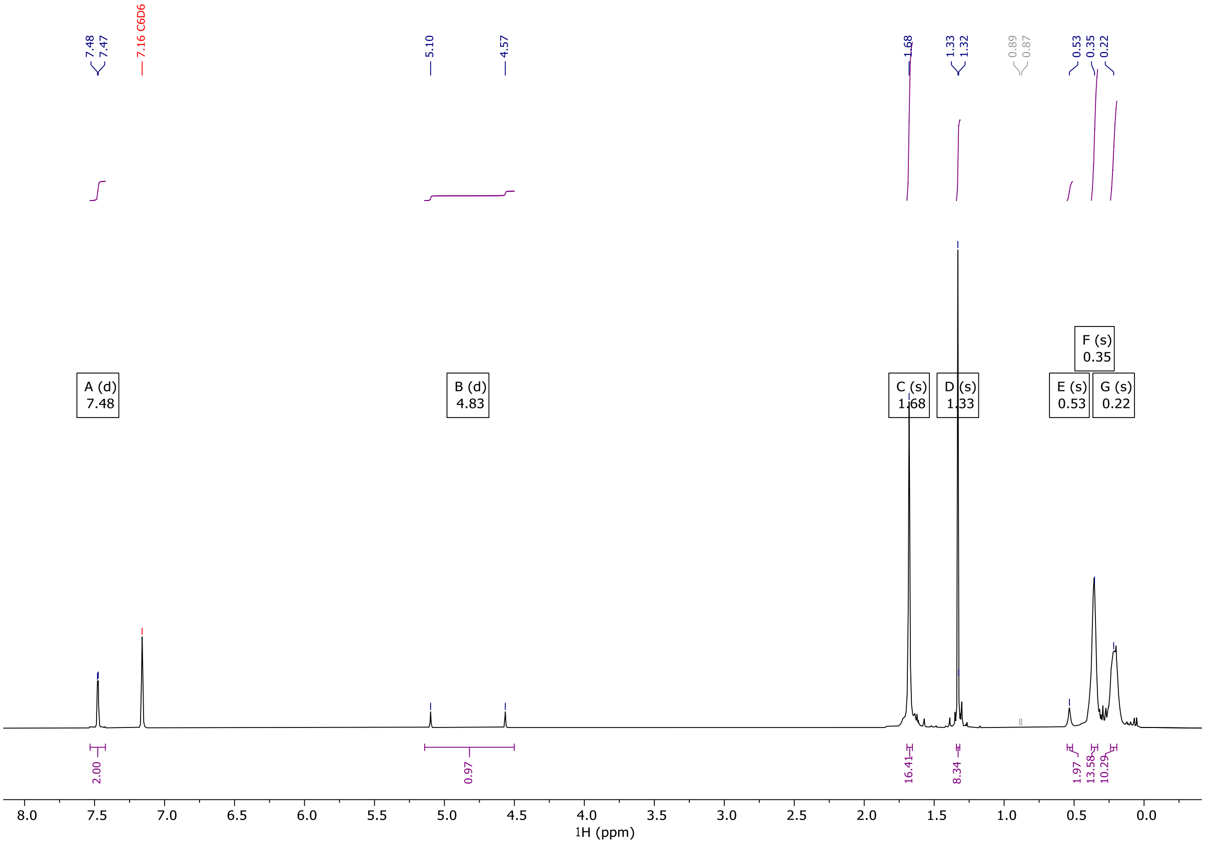

Figure S12. ^{1}H NMR (400 MHz, 293 K) spectrum of **2a** in C_6D_6.

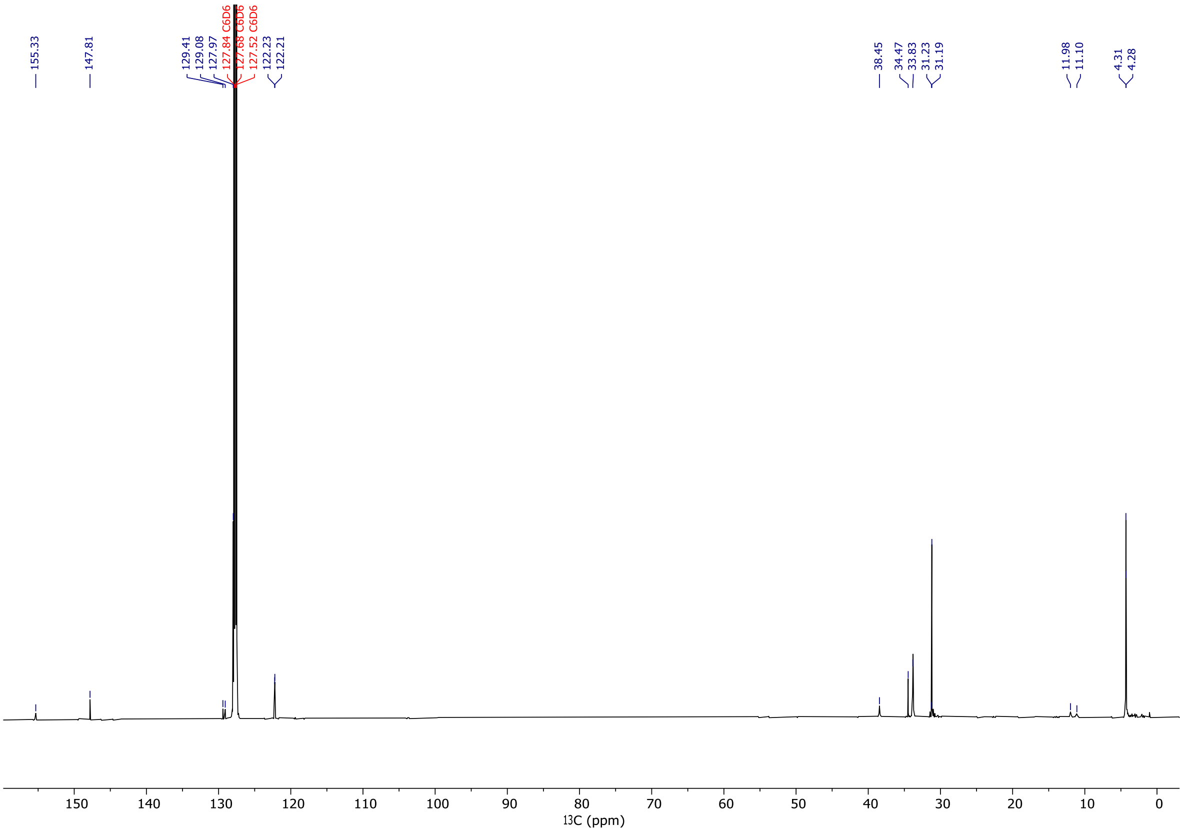

Figure S13. ^{13}C NMR spectrum (151 MHz, 293 K) spectrum of **2a** in C_6D_6.

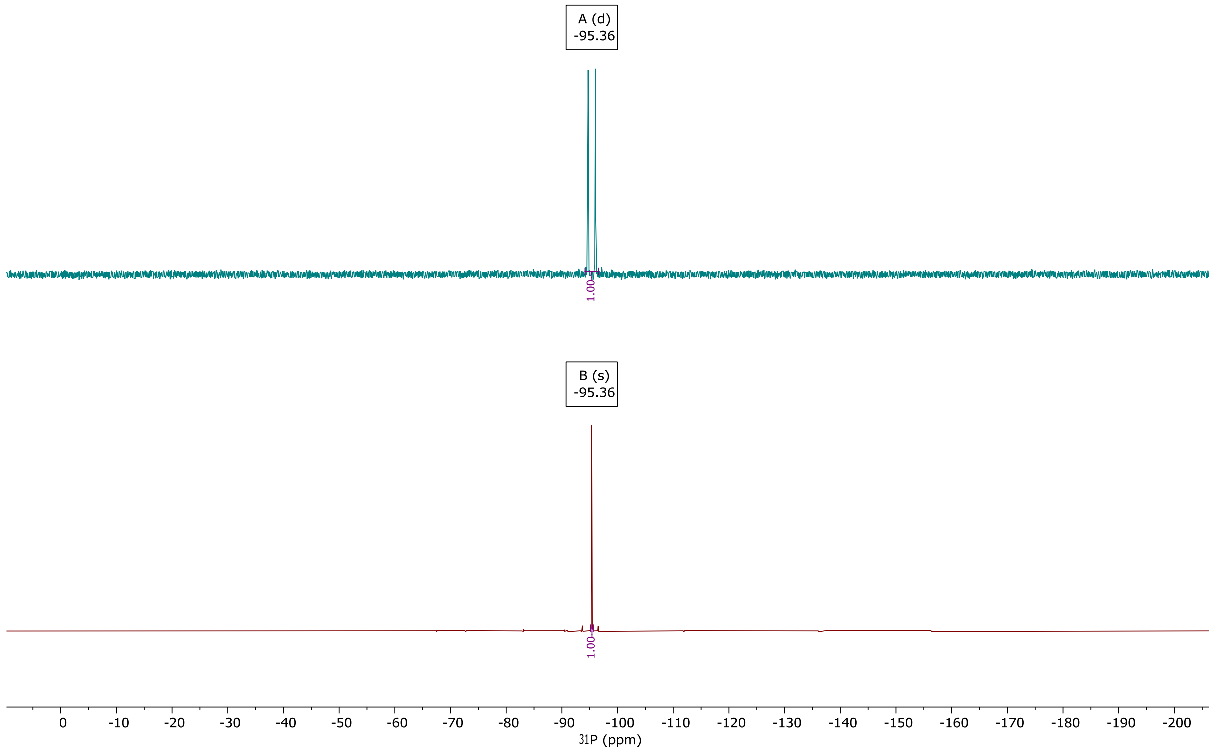

Figure S14. ^{31}P (top) and $^{31}P\{^1H\}$ (bottom) NMR spectra (162 MHz, 293 K) of **2a** in C_6D_6.

1.1.4. Synthesis of $[(Me_3Si)_2CH]_2Ge(NH^{n}Pr)P(H)Mes*$ (3a)

1b (50 mg, 0.075 mmol) was dissolved in toluene (0.6 mL) in an NMR tube before *n*-propylamine (0.01 mL, in excess) was added and the solution heated at 80 °C for 12 days. The volatiles were removed *in vacuo* and the solid residue was extracted with hexane (0.5 mL × 3). Slow evaporation of the hexane solution at room temperature yielded colourless cubic crystals suitable for X-ray diffraction (25.9 mg, 0.036 mmol, 48.0 % yield). Anal. calculated for $C_{35}H_{76}Ge_1N_1P_1Si_4$: C, 57.83; H, 10.54; N, 1.93. Found: C, 57.88; H, 10.18; N, 1.91.

^{1}H NMR (400 MHz, C_6D_6): δ (ppm) 7.45 (d, $^4J_{P\text{–}H}$ = 2.5 Hz, 2H; Mes* ArC*H*), 4.94 (d, $^1J_{P\text{–}H}$ = 214 Hz; P*H*), 2.92 (m, 2H; $NCH_2CH_2CH_3$), 1.69 (s, 18H; Mes* *ortho*-$C(CH_3)_3$), 1.49 (m(br), 2H; $NCH_2CH_2CH_3$), 1.34 (s, 9H; Mes* *para*-$C(CH_3)_3$), 0.94 (t, $^1J_{H\text{–}H}$ = 7.4 Hz, 3H; $NCH_2CH_2CH_3$) 0.78 (t, $^1J_{H\text{–}H}$ = 7.5 Hz, 1H; N*H*CH_2), 0.38 (s, 18H; $Si(CH_3)_3$), 0.28 (m, 18H; $Si(CH_3)_3$). Note: GeC*H* protons were observed in 2D spectra but were not assigned in 1D spectra due to overlap with peaks at 0.35 and 0.21 ppm.

^{13}C NMR (151 MHz, C_6D_6): δ (ppm): 155.09 (d, $^2J_{P\text{–}C}$ = 6.7 Hz; Mes* *ortho*-Ar*C*), 147.62 (Mes* *para*-Ar*C*), 130.52 (d, $^1J_{P\text{–}C}$ = 48.7 Hz; Mes* *ipso*-Ar*C*), 122.32 (Mes* *meta*-Ar*C*), 47.57 ($NCH_2CH_2CH_3$), 38.70 (Mes* *ortho*-$C(CH_3)_3$), 34.79 (Mes* *para*-$C(CH_3)_3$), 33.86 (d, $^4J_{P\text{–}C}$ = 7.1 Hz), Mes* *ortho*-$C(CH_3)_3$), 31.65 (Mes* *para*-$C(CH_3)_3$), 27.19 ($NCH_2CH_2CH_3$), 11.72 ($NCH_2CH_2CH_3$), 10.81 (Ge*C*H), 9.57 (Ge*C*H), 5.49 ($Si(CH_3)_3$), 5.46 ($Si(CH_3)_3$), 5.18 ($Si(CH_3)_3$).

^{31}P NMR (162 MHz, C_6D_6): δ (ppm) –101.9 (d, $^1J_{P\text{–}H}$ = 214 Hz).

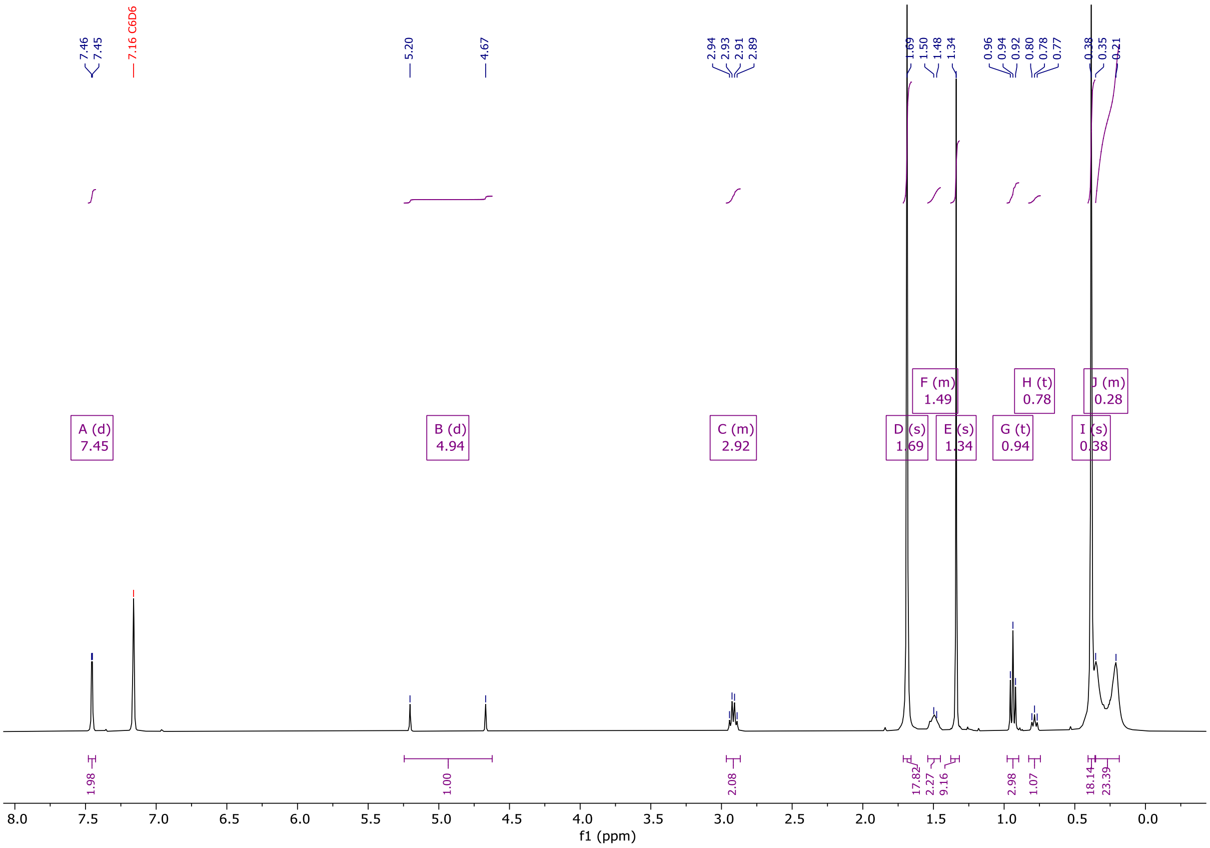

Figure S15. ^{1}H NMR spectrum (400 MHz, 293 K) of **3a** in C_6D_6.

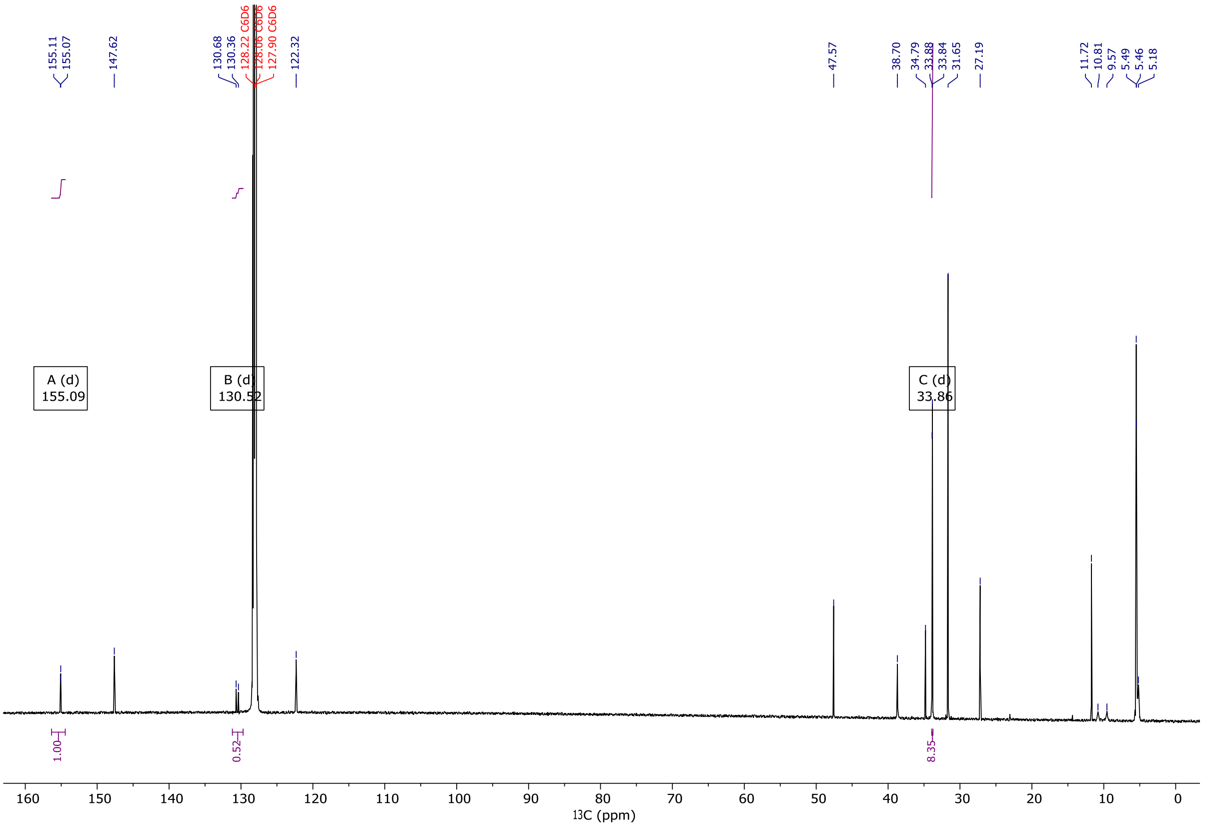

Figure S16. ^{13}C NMR spectrum (151 MHz, 293 K) of **3a** in C_6D_6.

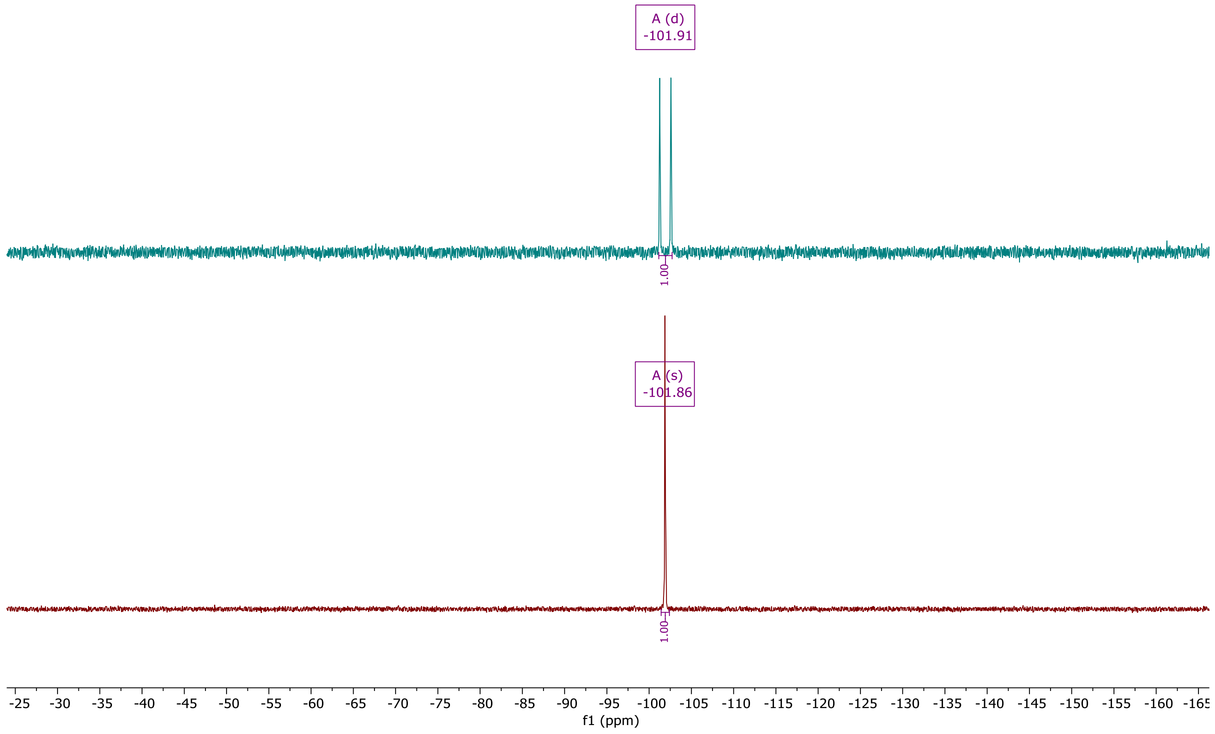

Figure S17. ^{31}P (top) and $^{31}P\{^1H\}$(bottom) NMR spectra (162 MHz, 293 K) of **3a** in C_6D_6.

1.1.5. Synthesis of $[(Me_3Si)_2CH]_2Ge(OH)P(H)Mes^*$ (4a)

1b was made in situ by stirring $Ge[CH(SiMe_3)_2]_2$ (33 mg, 0.0851 mmol) and Me_3P–PMes* (30 mg, 0.0851 mmol) in toluene (1 mL) until all the solids were dissolved. The solution was put under reduced pressure to remove the generated PMe_3 and re-dissolved in THF (0.5 mL). Degassed H_2O (0.02 mL, 1.11 mmol) was added and the solution was left stirring for 3 h. The volatiles were removed and was extracted with hexane (0.5 mL × 3). A small amount of hexamethyldisiloxane (0.1 mL) was added. Slow evaporation of the hexane/hexamethyldisiloxane solution at room temperature yielded colourless cubic crystals. (25.6 mg, 0.037 mmol, 43.5 % yield). Crystals suitable for X-ray diffraction were grown on standing of a benzene solution at room temperature. Anal. calculated for $C_{35}H_{69}Ge_1O_1P_1Si_4$: C, 56.04; H, 10.14; N, 0.00. Found: C, 55.98; H, 10.31; N, 0.00.

^{1}H NMR (400 MHz, C_6D_6): δ (ppm) 7.48 (s, 2H; Mes* ArC*H*), 5.11 (d, $^1J_{P-H}$ = 210 Hz; P*H*), 1.64 (s, 18H; Mes* *ortho*-C(C*H*$_3$)$_3$), 1.31 (s, 9H; Mes* *para*-C(C*H*$_3$)$_3$), 0.95 (s, 1H; GeC*H*), 0.45 (br, 2H; GeC*H*), 0.36 (d, *J* = 11.9 Hz, 18H; Si(C*H*$_3$)$_3$), 0.21 (d, *J* = 19.2 Hz, 18H; Si(C*H*$_3$)$_3$).

^{13}C NMR (151 MHz, C_6D_6): δ (ppm) 155.88 (Mes* *ortho*-Ar*C*), 148.42 (Mes* *para*-Ar*C*), 127.62 (Mes* *ipso*-Ar*C*), 123.03 (Mes* *meta*-Ar*C*), 39.07 (Mes* *ortho*-*C*(CH$_3$)$_3$), 34.84 (Mes* *para*-*C*(CH$_3$)$_3$), 34.29 (br; Mes* *ortho*-C(*C*H$_3$)$_3$), 31.38 (Mes* *para*-C(*C*H$_3$)$_3$), 14.60 (Ge*C*H), 13.33 (Ge*C*H), 4.66 (Si(*C*H$_3$)$_3$), 4.36 (Si(*C*H$_3$)$_3$).

^{31}P NMR (162 MHz, C_6D_6): δ (ppm) –96.1 (d, $^1J_{P-H}$ = 209 Hz).

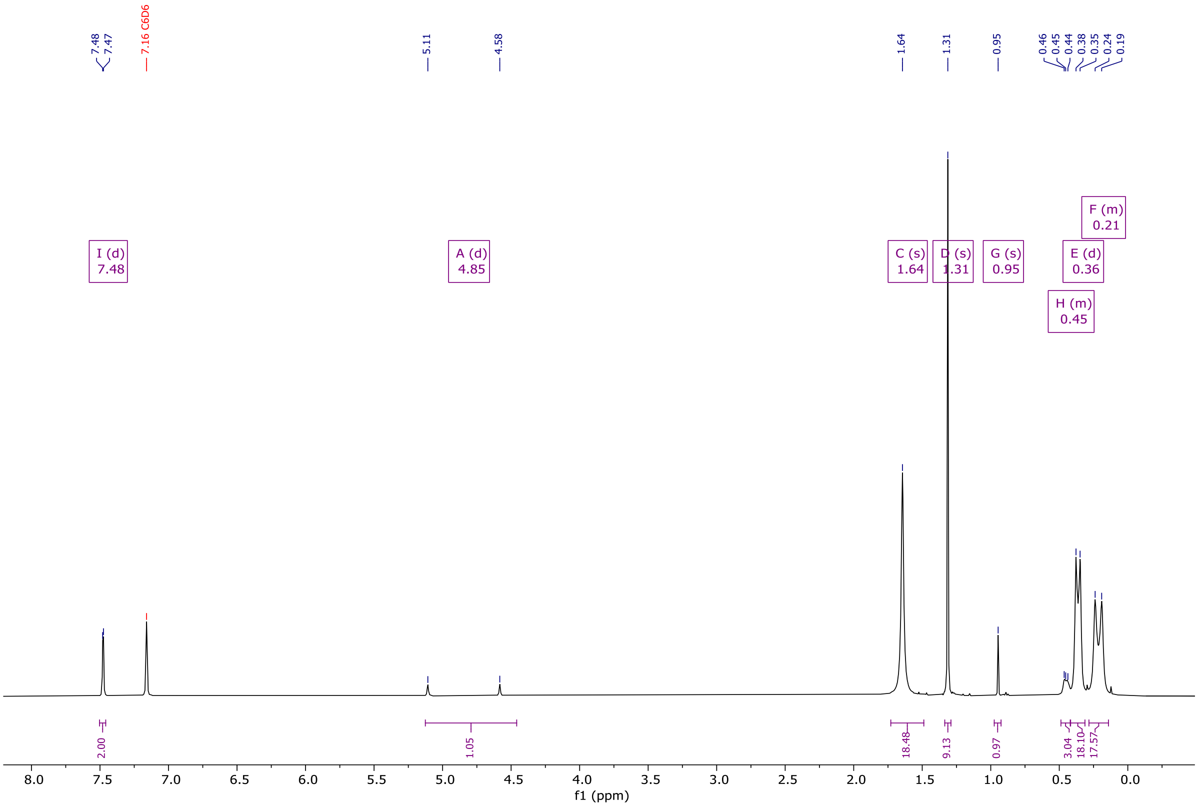

Figure S18. ^{1}H NMR spectrum (400 MHz, 293 K) of **4a** in C_6D_6.

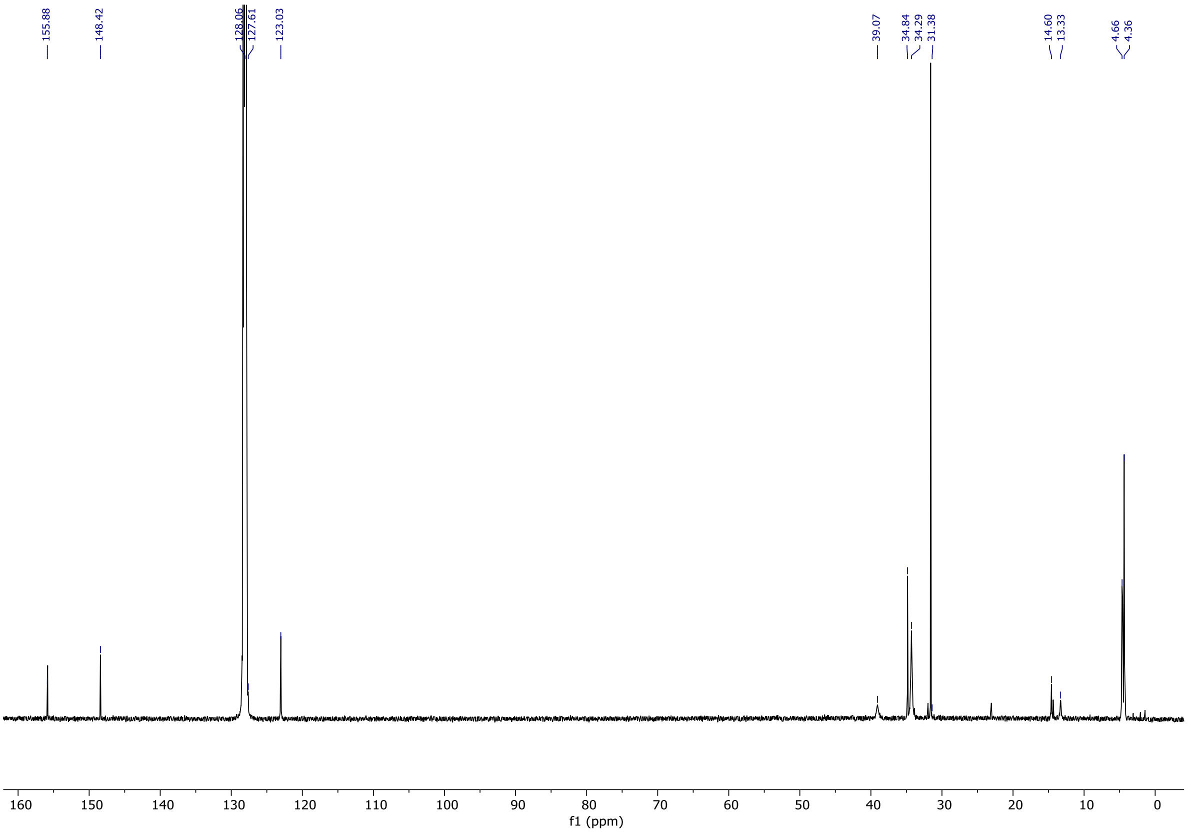

Figure S19. ^{13}C NMR spectrum (151 MHz, 293 K) of **4a** in C_6D_6.

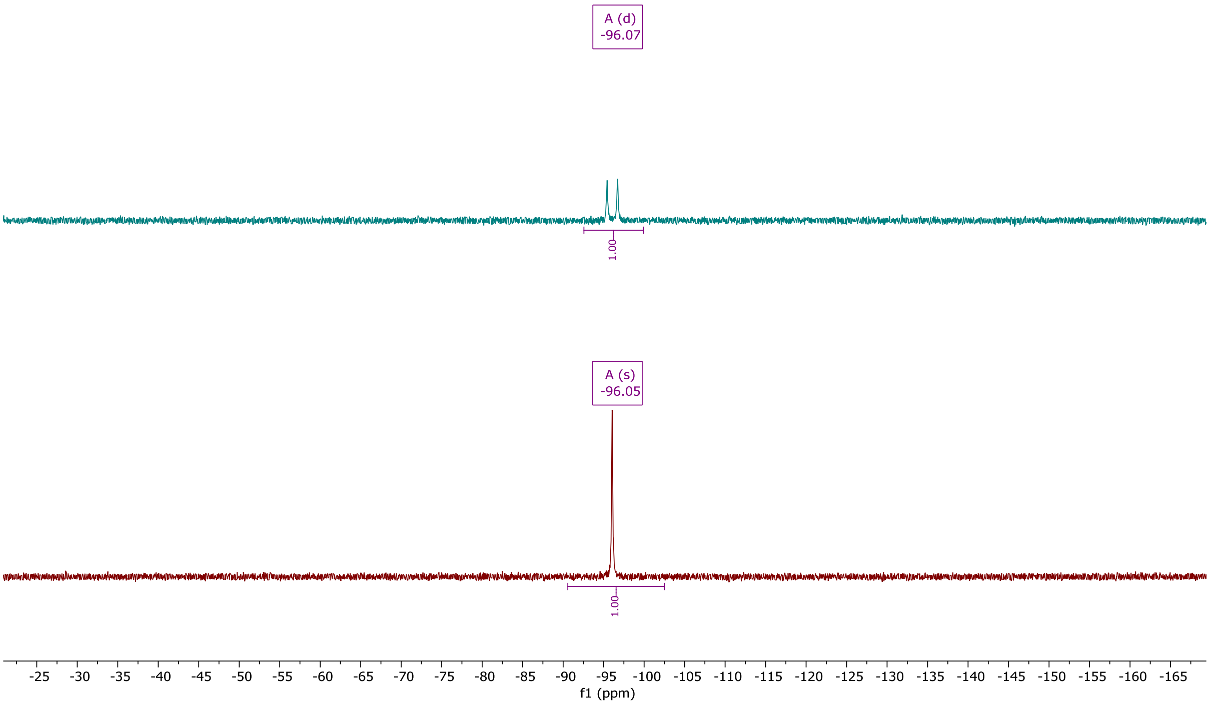

Figure S20. ^{31}P (top) and $^{31}P\{^1H\}$ (bottom) NMR spectra (162 MHz, 293 K) of **4a** in C_6D_6.

1.1.6. Synthesis of $[(Me_3Si)_2CH]_2Sn(NH_2)P(H)Mes^*$ (2b)

1b was prepared *in situ* by reacting $Sn[CH(SiMe_3)_2]_2$ (75 mg, 0.171 mmol) with $Me_3P–PMes^*$ (60.4 mg, 0.171 mmol) in toluene (2 mL). The volatiles were removed *in vacuo* to remove the generated PMe_3 and the solids redissolved in toluene (2 mL). The solution was degassed using the freeze-pump-thaw method and put under 1 bar NH_3 gas. The solution immediately decolourised. The volatiles were removed *in vacuo* and the solids extracted with *n*-pentane (1 mL × 3). A small amount of hexamethyldisiloxane (0.1 mL) was added. Slow evaporation of the *n*-pentane/hexamethyldisiloxane solution at room temperature yielded colourless crystals which were filtered and washed with a small amount of cold HMDSO (37.2 mg, 0.051 mmol, 29.8%). Crystals suitable for X-ray diffraction were grown from standing of a concentrated C_6D_6 solution at room temperature. Anal. calculated for $C_{32}H_{70}N_1P_1Si_4Sn_1$: C, 52.58; H, 9.65; N, 1.92. Found: C, 52.91; H, 9.55; N, 1.66.

^{1}H NMR (400 MHz, C_6D_6): δ (ppm) 7.48 (d, $^4J_{P–H}$ = 2.5 Hz, 2H; Mes* ArC*H*), 5.05 (d, $^1J_{P–H}$ = 206 Hz, 1H; P*H*), 1.70 (s, 18H; Mes* *ortho*-C(CH_3)$_3$), 1.32 (s, 9H; Mes* *para*-C(CH_3)$_3$), 0.35 (s, 9H; Si(CH_3)$_3$), 0.33 (s, 9H; Si(CH_3)$_3$), 0.19 (s, 18H; Si(CH_3)$_3$), 0.13 (s (br), 2H, NH_2). Note: SnC*H* protons were observed in 2D spectra but were not assigned in 1D spectra due to overlap with peaks at 0.19 ppm.

^{13}C NMR (151 MHz, C_6D_6): δ (ppm) 155.27 (Mes* *ortho*-Ar*C*), 148.55 (Mes* *ortho*-Ar*C*), 122.51 (d, $^4J_{P–C}$ = 3.3 Hz; Mes* *meta*-Ar*C*), 38.50 (Mes* *ortho*-*C*(CH$_3$)$_3$), 34.91 (Mes* *para*-*C*(CH$_3$)$_3$), 33.75 (d, $^4J_{P–C}$ = 7.1 Hz; Mes* *ortho*-C(*C*H$_3$)$_3$), 31.52 (Mes* *para*-C(*C*H$_3$)$_3$), 10.08 (Sn*C*H), 9.37 (d, $^3J_{P–C}$ = 6.7 Hz, Sn*C*H), 4.52 (Si(*C*H$_3$)$_3$), 4.42 (Si(*C*H$_3$)$_3$). Note: Mes* *ipso*-Ar*C* could not be located.

^{31}P NMR (162 MHz, C_6D_6): δ (ppm) –113.6 (d, $^1J_{P–H}$ = 206 Hz).

^{119}Sn NMR (186 MHz, C_6D_6): δ (ppm) 56.1 (d, $^1J_{Sn–P}$ = 1019 Hz).

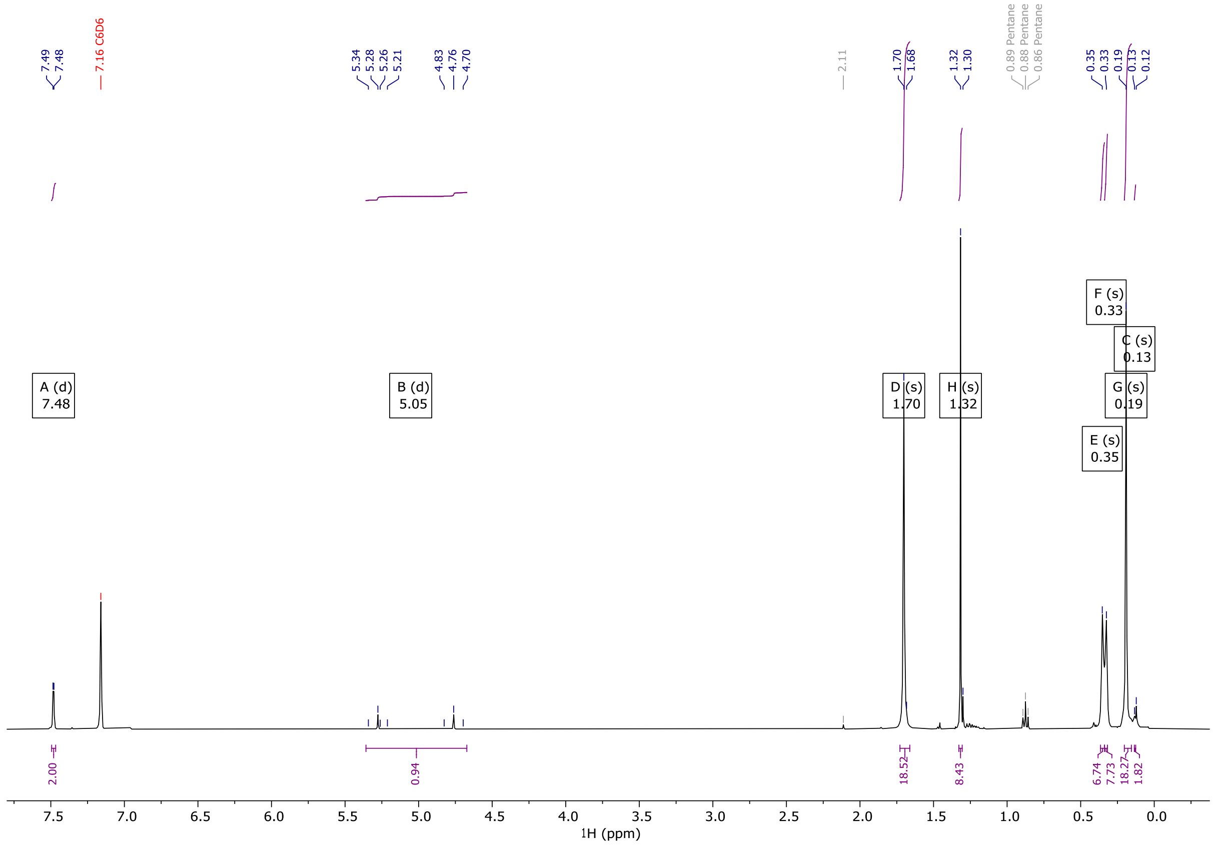

Figure S21. ^{1}H NMR spectrum (400 MHz, 293 K) of **2b** in C_6D_6.

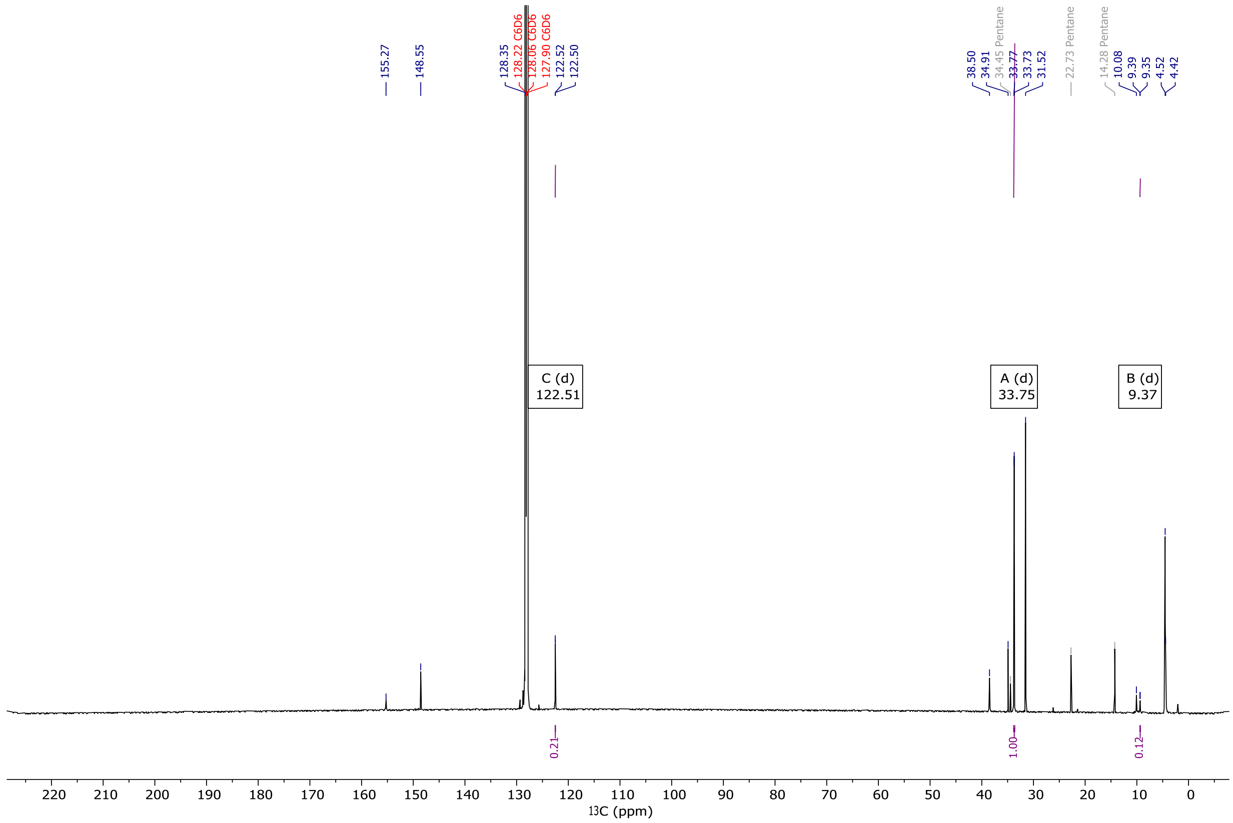

Figure S22. ^{13}C NMR spectrum (151 MHz, 293 K) of **2b** in C_6D_6.

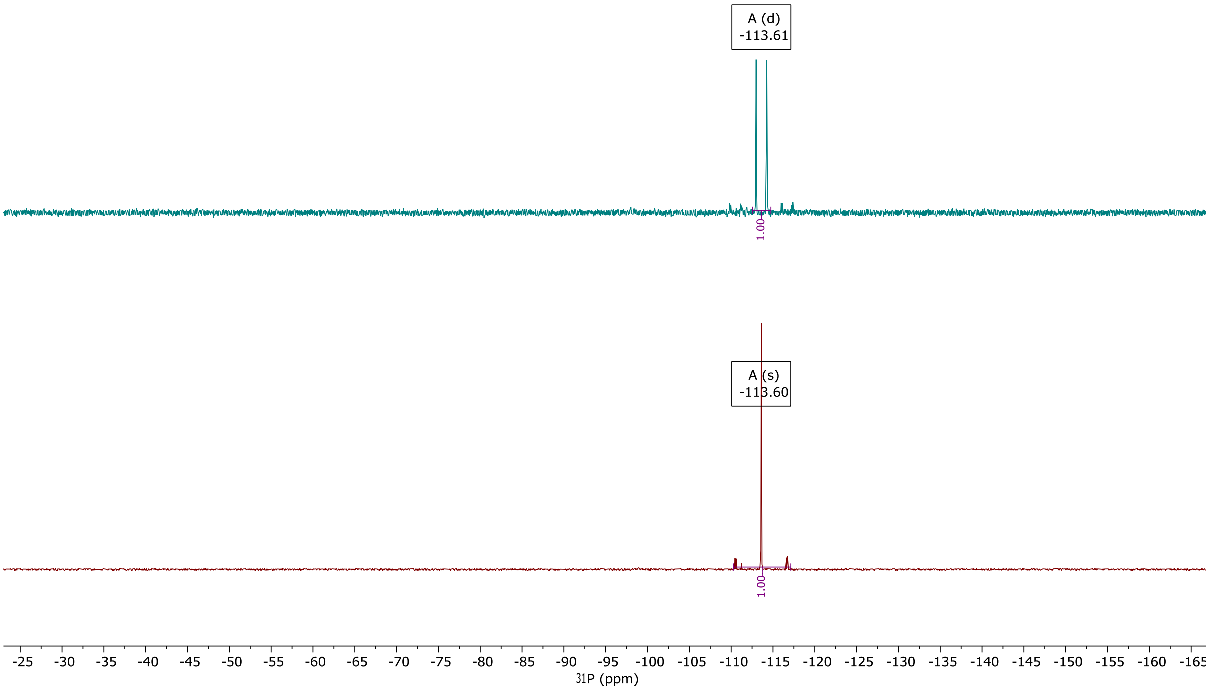

Figure S23. ^{31}P (top) and ^{31}P{^{1}H} (bottom) NMR spectra (162 MHz, 293 K) of **2b** in C_6D_6.

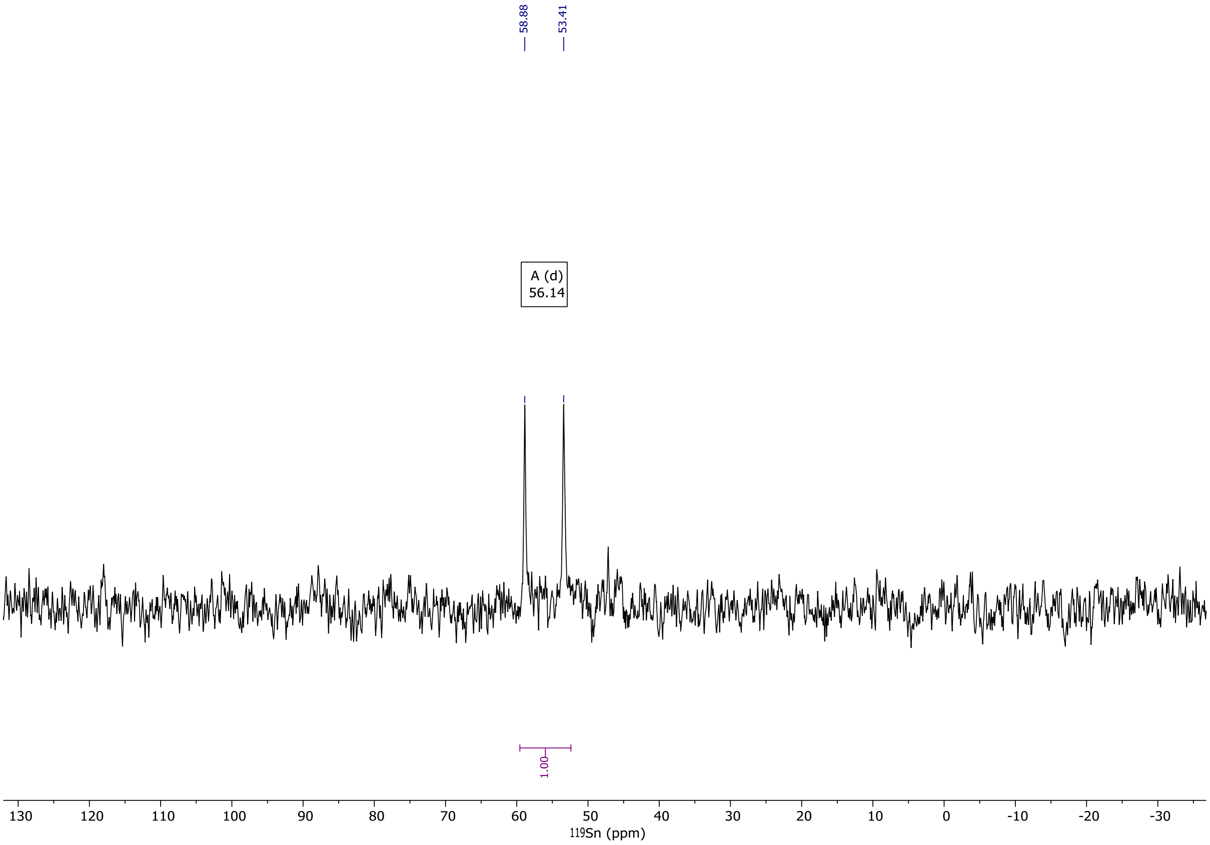

Figure S24. ^{119}Sn NMR spectrum (186 MHz, 293 K) of **2b** in C_6D_6.

1.1.7. Synthesis of $[(Me_3Si)_2CH]_2Sn(NH^nPr)P(H)Mes^*$ (3b)

1b was made in situ by stirring $Sn[CH(SiMe_3)_2]_2$ (37 mg, 0.0851 mmol) and $Me_3P–PMes^*$ (30 mg, 0.0851 mmol) in toluene (3 mL) until all the solids were dissolved. The solution was put under reduced pressure to remove the generated PMe_3 and re-dissolved in toluene (3 mL). npropylamine (0.02 mL, 1.11 mmol) was added and the solution was stirred for 20 mins, during which the solution decolourised. The volatiles were removed and was extracted with hexane (0.5 mL × 3). A small amount of hexamethyldisiloxane (0.1 mL) was added. Slow evaporation of the hexane/hexamethyldisiloxane solution at room temperature yielded colourless cubic crystals (24.7 mg, 0.032 mmol, 37.6 % yield). Crystals suitable for X-ray diffraction were grown by slow evaporation of a hexane solution at room temperature. Anal. calculated for $C_{35}H_{76}N_1P_1Sn_1Si_4$: C, 54.38; H, 9.91; N, 1.81. Found: C, 54.54; H, 10.22; N, 1.78.

^{1}H NMR (600 MHz, C_6D_6): δ (ppm) 7.46 (s, $^4J_{P–H}$ = 2.5 Hz, 2H; Mes* ArC*H*), 5.04 (d, $^1J_{P–H}$ = 205.2 Hz; P*H*), 3.07 – 2.96 (m, 2H; $NCH_2CH_2CH_3$), 1.70 (s, 18H; Mes* *ortho*-$C(CH_3)_3$), 1.59 – 1.47 (m, 2H; $NCH_2CH_2CH_3$), 1.33 (s, 9H; Mes* *para*-$C(CH_3)_3$), 0.96 (t, $^3J_{H–H}$ = 7.4 Hz, 3H; $NCH_2CH_2CH_3$), 0.41 (s, 1H; N*H*), 0.36 (s, 18H; $Si(CH_3)_3$), 0.33 (s(br), 9H; $Si(CH_3)_3$), 0.19 (s(br), 9H; $Si(CH_3)_3$).

^{13}C NMR (151 MHz, C_6D_6): δ (ppm) 154.85 (Mes* *ortho*-Ar*C*), 147.84 (Mes* *para*-Ar*C*), 129.35 (d, $^1J_{P–C}$ = 50.7 Hz; Mes* *ipso*-Ar*C*), 122.34 (Mes* *meta*-Ar*C*), 49.50 (NCH_2), 38.65 (Mes* *ortho*-$C(CH_3)_3$), 34.85 (Mes* *para*-$C(CH_3)_3$), 33.63 (d, $^5J_{P–C}$ = 7.3 Hz; Mes* *ortho*-$C(CH_3)_3$, 31.64 (Mes* *para*-$C(CH_3)_3$, 28.78 ($NCH_2CH_2CH_3$), 11.58 ($NCH_2CH_2CH_3$), 9.08 (Sn*C*H), 7.89 (Sn*C*H), 5.00 ($Si(CH_3)_3$), 4.95 ($Si(CH_3)_3$), 4.76 ($Si(CH_3)_3$).

^{31}P NMR (162 MHz, C_6D_6): δ (ppm) –117.6 (d, $^1J_{P–H}$ = 206 Hz).

^{119}Sn NMR (186 MHz, C_6D_6): δ (ppm) 49.4 (d, $^1J_{Sn–P}$ = 973 Hz).

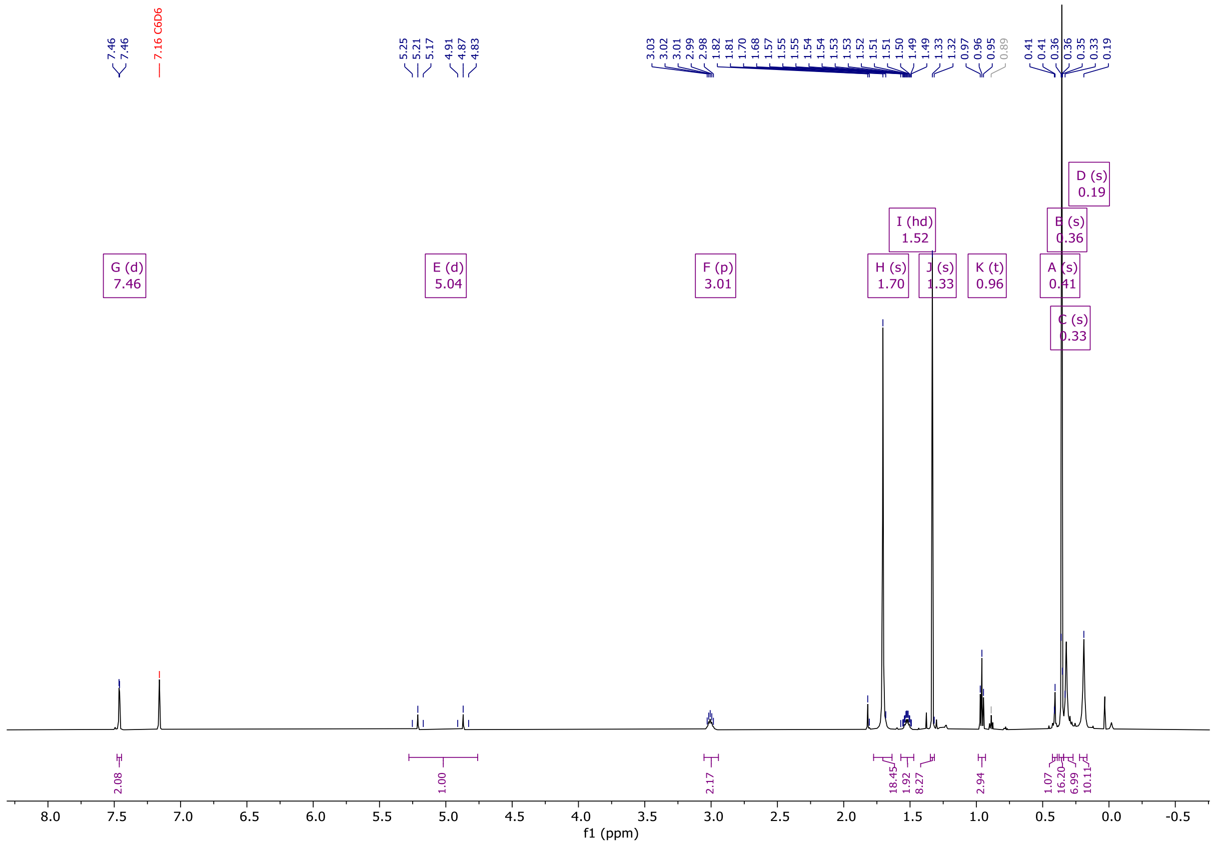

Figure S25. ^{1}H NMR spectrum (600 MHz, 293 K) of **3b** in C_6D_6.

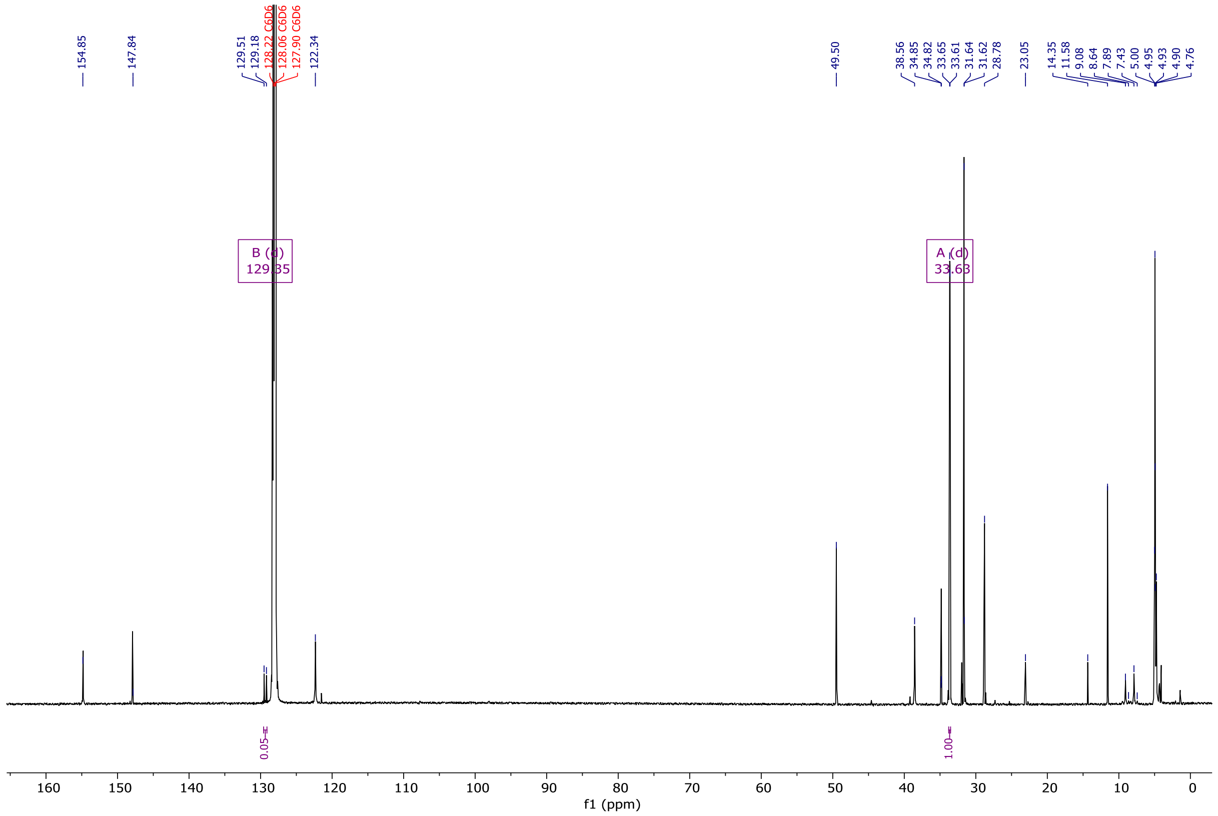

Figure S26. ^{13}C NMR spectrum (151 MHz, 293 K) of **3b** in C_6D_6.

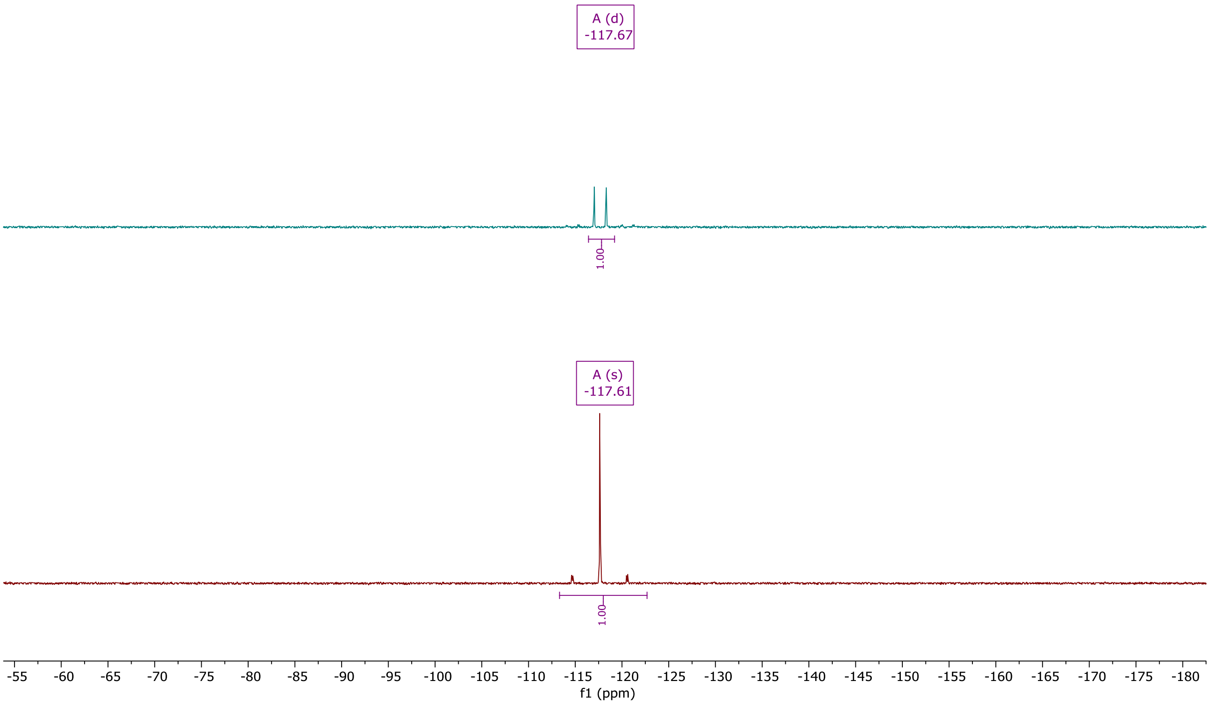

Figure S27. ^{31}P (top) and ^{31}P{^{1}H}(bottom) NMR spectra (162 MHz, 293 K) of **3b** in C_6D_6.

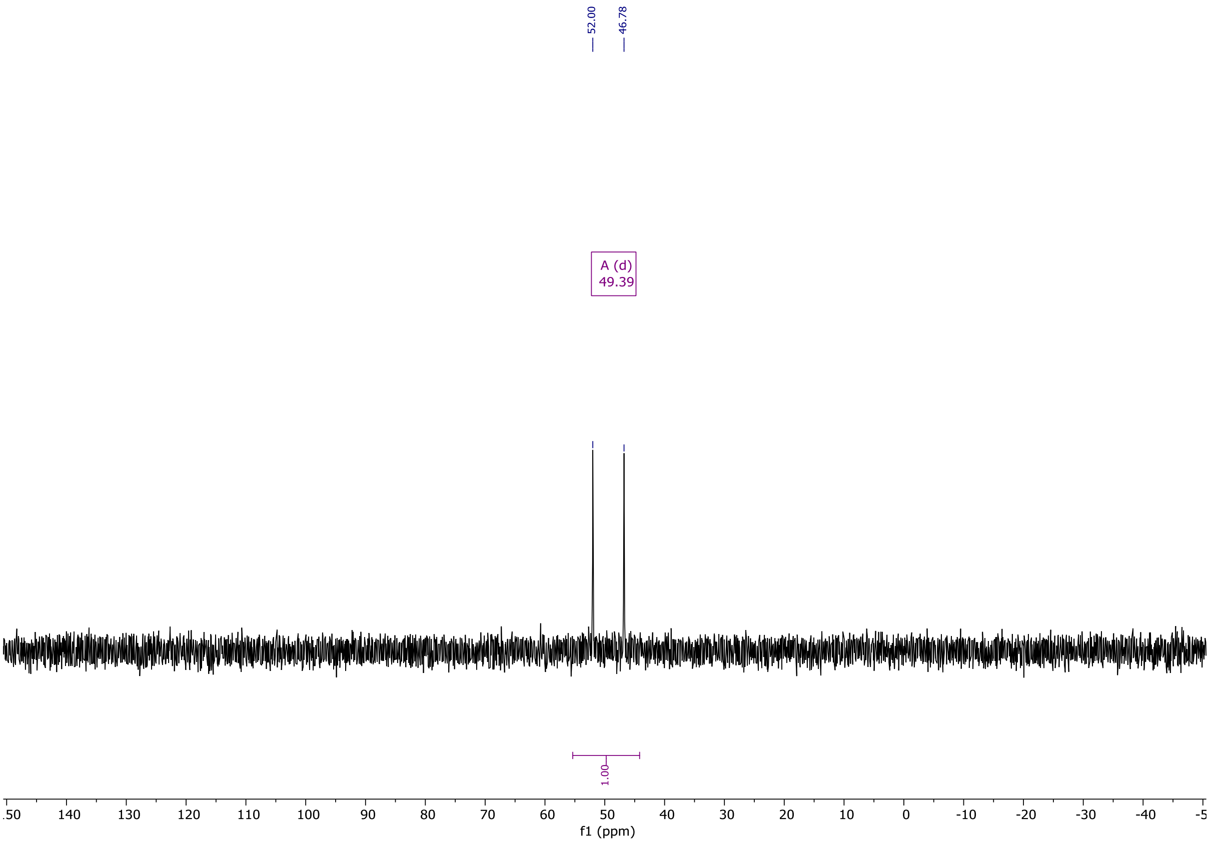

Figure S28. ^{119}Sn NMR spectrum (186 MHz, 293 K) of **3b** in C_6D_6.

1.1.8. Synthesis of $[(Me_3Si)_2CH]_2Sn(OH)P(H)Mes^*$ (4b)

1b was made in situ by stirring $Sn[CH(SiMe_3)_2]_2$ (37 mg, 0.0851 mmol) and Me_3P–PMes* (30 mg, 0.0851 mmol) in toluene (1 mL) until all the solids were dissolved. The solution was put under reduced pressure to remove the generated PMe_3 and re-dissolved in toluene (0.5 mL). Degassed H_2O (0.02 mL, 1.11 mmol) was added and the solution was shaken for 30 seconds. The solution immediately decolourised. The volatiles were removed and the solids were extracted with hexane (0.5 mL × 3). Slow evaporation of the hexane solution at room temperature yielded colourless crystals (15 mg, 0.0205 mmol, 24.1% yield). Crystals suitable for X-ray diffraction were grown on standing of a benzene solution at room temperature. Anal. calculated for $C_{32}H_{69}O_1P_1Sn_1Si_4$: C, 52.51; H, 9.50; N, 0.00. Found: C, 52.96; H, 9.84; N, 0.00.

^{1}H NMR (400 MHz, C_6D_6): δ (ppm) 7.48 (s, 2H; Mes* ArC*H*), 5.11 (d, $^{1}J_{P–H}$ = 205.5 Hz; P*H*), 1.68 (s, 18H; Mes* *ortho*-C(CH_3)$_3$), 1.30 (s, 9H; Mes* *para*-C(CH_3)$_3$), 0.36 (s, 18H; Si(CH_3)$_3$), 0.30 (s, 1H, SnC*H*), 0.25 (s, 1H; SnC*H*), 0.19 (m, 18H; Si(CH_3)$_3$). Note: We were unable to observe the SnO*H* resonance.

^{13}C NMR (151 MHz, C_6D_6): δ (ppm) 155.48 (Mes* *ortho*-Ar*C*), 149.00 (Mes* *para*-Ar*C*), 126.96 (Mes* *ipso*-Ar*C*), 122.73 (Mes* *meta*-Ar*C*), 122.71 (Mes* *meta*-Ar*C*), 38.55 (Mes* *ortho*-*C*(CH$_3$)$_3$), 34.92 (Mes* *para*-*C*(CH$_3$)$_3$), 33.77 (d, $^{5}J_{P–C}$ = 7.2 Hz; Mes* *ortho*-C(*C*H$_3$)$_3$), 31.49 (Mes* *para*-C(*C*H$_3$)$_3$), 13.99 (Sn*C*H), 13.16 (Sn*C*H), 4.38 (Si(*C*H$_3$)$_3$), 4.32 (Si(*C*H$_3$)$_3$).

^{31}P NMR (162 MHz, C_6D_6): δ (ppm) –111.2 (d, $^{1}J_{P–H}$ = 205 Hz).

^{119}Sn NMR (186 MHz, C_6D_6): δ (ppm) 80.7 (d, $^{1}J_{Sn–P}$ = 1073 Hz).

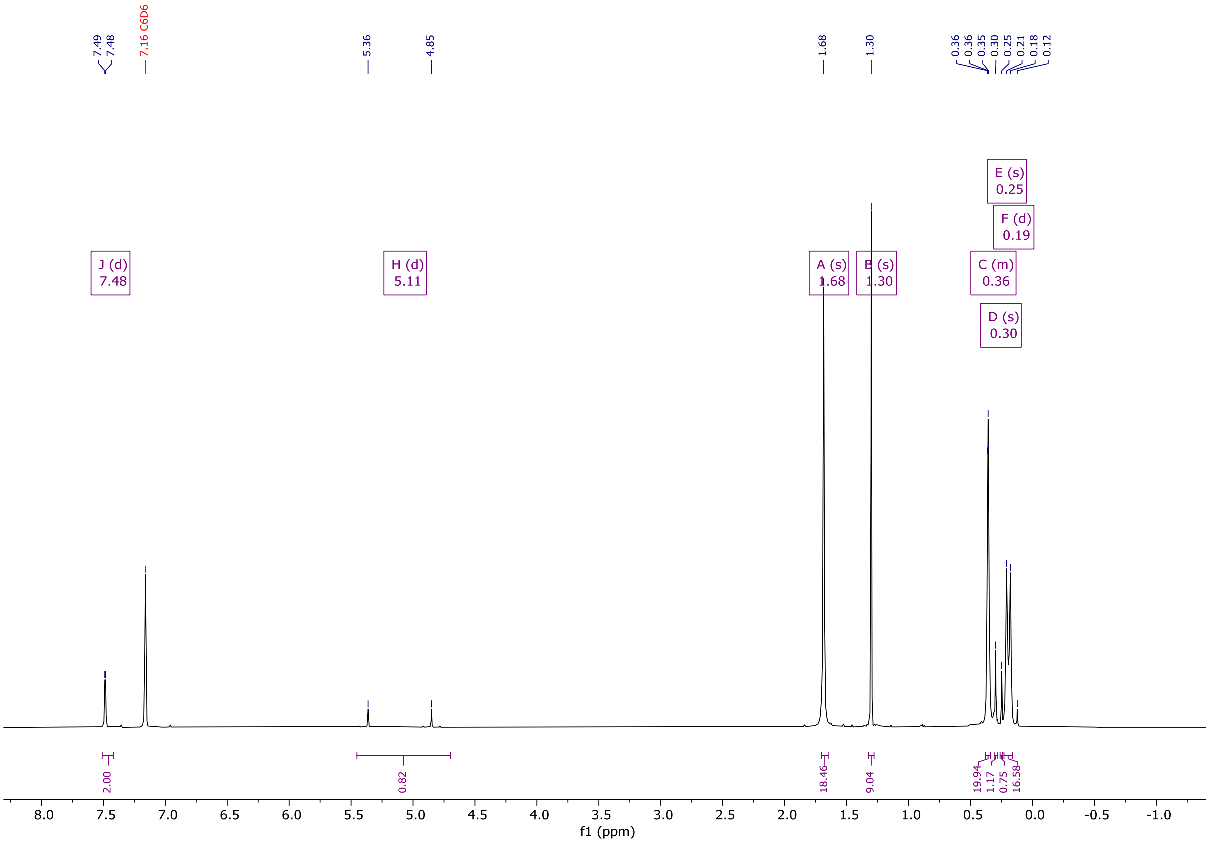

Figure S29. ^{1}H NMR spectrum (400 MHz, 293 K) of **4b** in C_6D_6.

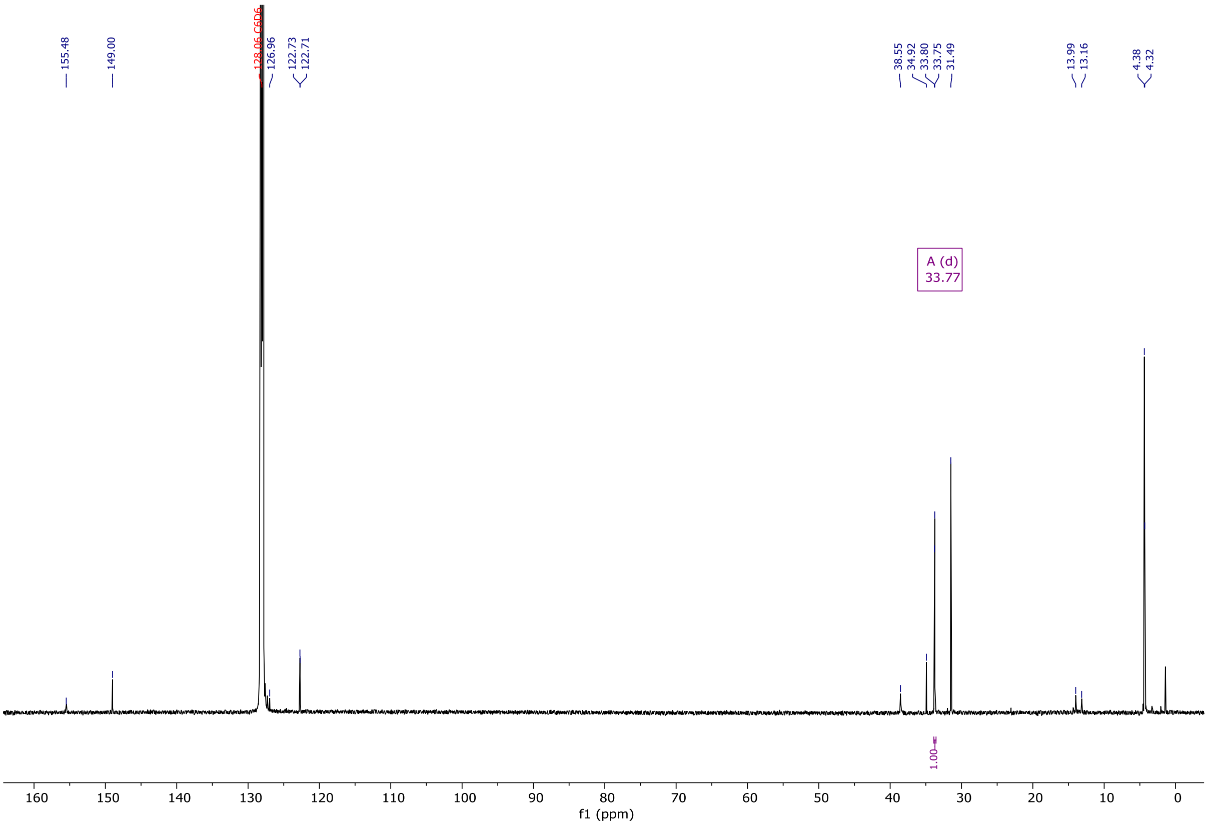

Figure S30. ^{13}C NMR spectrum (151 MHz, 293 K) of **4b** in C_6D_6.

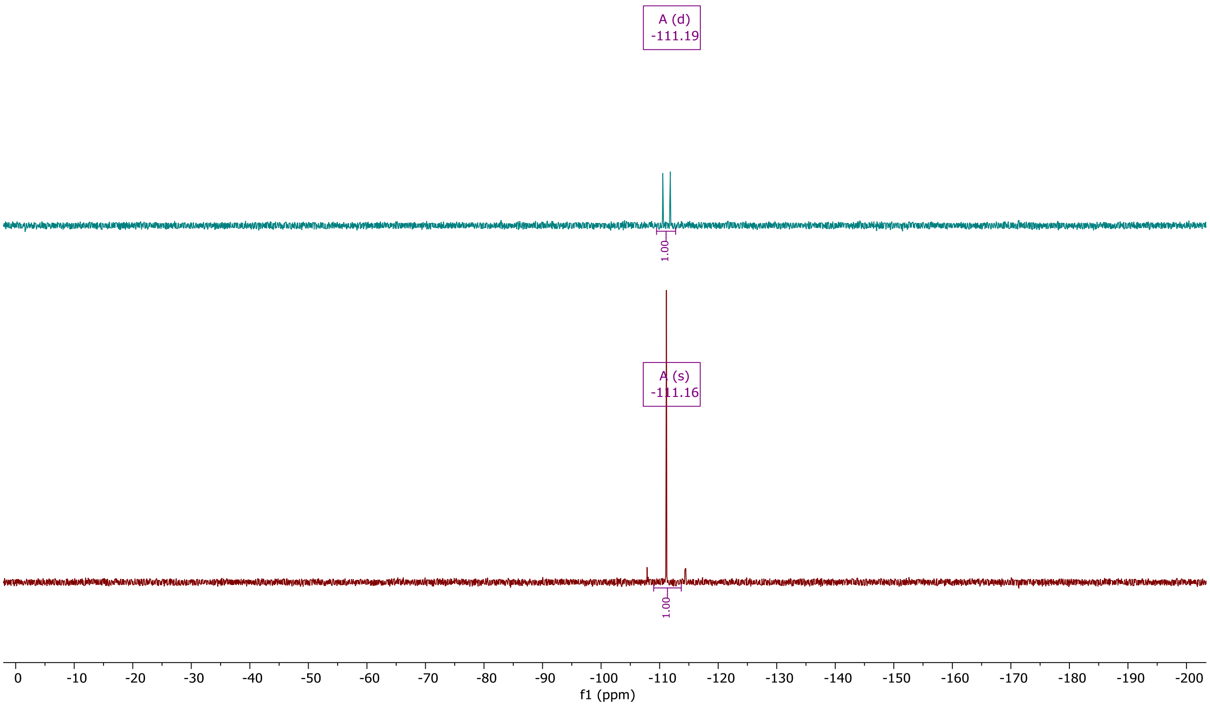

Figure S31. ^{31}P (top) and $^{31}P\{^1H\}$(bottom) NMR spectra (162 MHz, 293 K) of **4b** in C_6D_6.

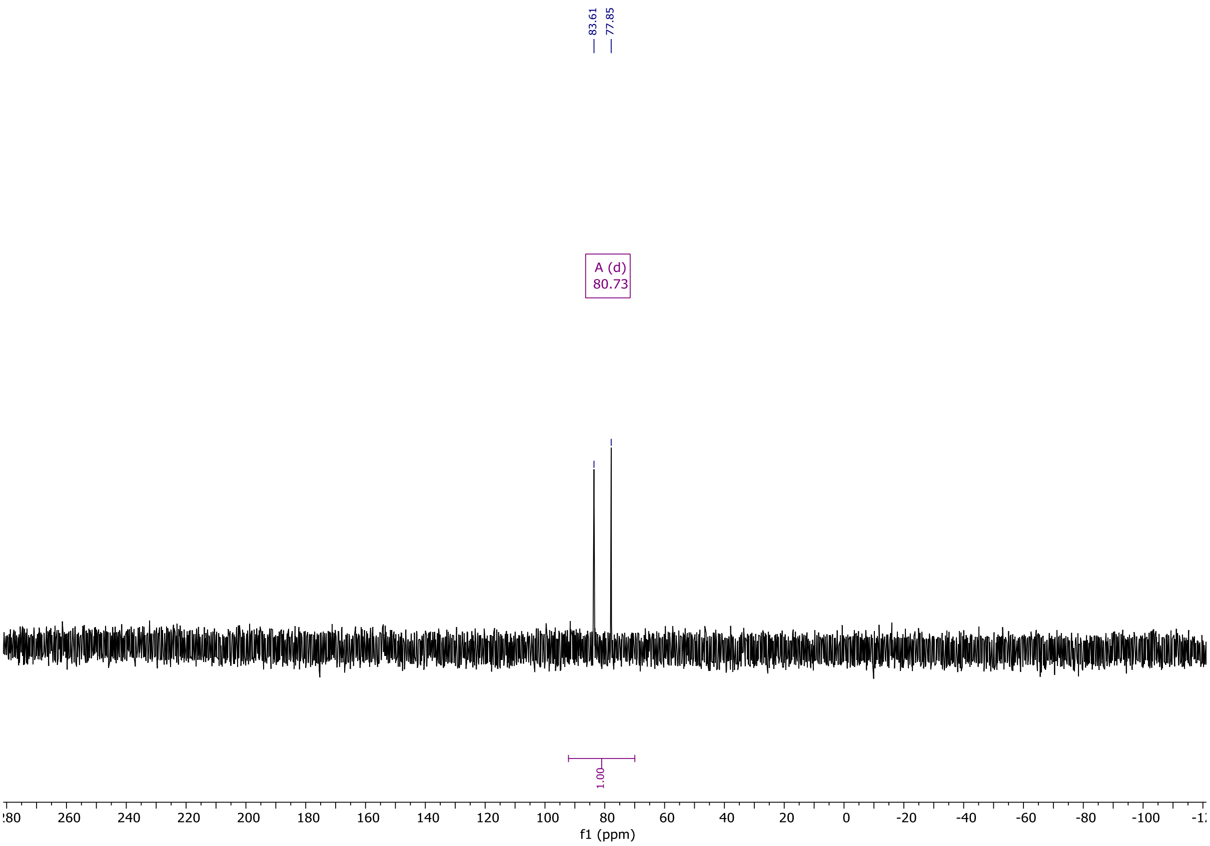

Figure S32. ^{119}Sn NMR spectrum (186 MHz, 293 K) of **4b** in C_6D_6.

1.1.9. Synthesis of [$(Me_3Si)_2CH]_2Sn(NH^iPr)P(H)Mes*$ (5b)

1b was generated *in situ* by stirring Sn[CH$(SiMe_3)_2]_2$ (75 mg, 0.171 mmol) and Me_3P–PMes* (60.4 mg, 0.171 mmol) in toluene (2 mL) until the solids dissolved. The volatiles were removed *in vacuo* followed by redissolving the remaining solids in toluene (2 mL). Isopropylamine (0.05 mL, 0.582 mmol, in excess) was added and the solution heated at 80 °C for 20 minutes, causing the colour to fade. After stirring at room temperature for a further 30 minutes, the volatiles were removed *in vacuo* and the solids extracted with pentane (1 mL × 3). A small amount of hexamethyldisiloxane (0.1 mL) was added. Slow evaporation of the pentane/hexamethyldisiloxane solution at room temperature gave an orange oil that was cooled to −35 °C for 24 hours. Pentane (3 drops) was added, crystals were formed, and the solution cooled again to −35 °C for 2 hours. The liquid was decanted and the resulting crystals were washed sequentially with a small amount of cold toluene then cold HMDSO to yield pale yellow/orange crystals. (16.3 mg, 0.021 mmol, 12.3% yield). Crystals suitable for X-ray diffraction were grown by slow evaporation of a pentane/HMDSO solution at room temperature. Anal. calculated for $C_{35}H_{76}N_1P_1Si_4Sn_1$: C, 54.38; H, 9.91; N, 1.81. Found: C, 53.19; H, 9.70; N, 1.68. Note: We observed reformation of **1b** (ca. 10% by $^{31}P\{^1H\}$ NMR spectroscopy) after leaving samples of **5b** either under a dynamic vacuum for extended periods or upon recrystalisation, which is accompanied by a colour change to red/orange. We were thus unable to obtain samples of **5b** pure enough to obtain satisfactory elemental analysis results.

^{1}H NMR (400 MHz, C_6D_6): δ (ppm) 7.45 (d, $^4J_{P–H}$ = 2.5 Hz, 2H; Mes*ArC*H*), 5.01 (d, $^1J_{P–H}$ = 208 Hz, 1H; P*H*), 3.25 (m, 1H; N*H*), 1.70 (s, 18H; Mes* *ortho*-C(CH_3)$_3$), 1.34 (s, 9H; Mes* *para*-C(CH_3)$_3$), 1.22 (dd, $^2J_{H–H}$ = 11.6 Hz, $^3J_{H–H}$ = 6.1 Hz, 6H; N(H)C(H)(CH_3)$_2$), 0.39 (s, 9H; Si(CH_3)$_3$), 0.36 (s(br), 18H; Si(CH_3)$_3$), 0.18 (s, 9H; Si(CH_3)$_3$). Note: SnC*H* protons were observed in 2D spectra but were not assigned in 1D spectra due to overlap with peaks at 0.18 ppm.

^{13}C NMR (151 MHz, C_6D_6): δ (ppm) 154.37 (d, $^2J_{P–C}$ = 6.5 Hz; Mes* *ortho*-Ar*C*), 147.72 (Mes* *para*-Ar*C*), 129.77 (d, $^1J_{P–C}$ = 50.5 Hz; Mes* *ipso*-Ar*C*), 122.20 (s(br); Mes* *meta*-Ar*C*), 48.18 (N(H)*C*(H)(CH$_3$)$_2$), 38.53 (Mes* *ortho*-*C*(CH$_3$)$_3$), 34.79 (Mes* *para*-*C*(CH$_3$)$_3$), 33.74 (d, $^4J_{P–C}$ = 7.0 Hz; Mes* *ortho*- C(*C*H$_3$)$_3$), 31.70 (Mes* *para*- C(*C*H$_3$)$_3$), 30.29 (NC(H)(*C*H$_3$)$_2$), 29.97 (NC(H)(*C*H$_3$)$_2$), 8.81 (Sn*C*H), 8.01 (Sn*C*H), 5.22 (Si(*C*H$_3$)$_3$), 5.14 (Si(*C*H$_3$)$_3$), 4.98 (Si(*C*H$_3$)$_3$).

^{31}P NMR (162 MHz): δ (ppm) −103.2 (d, $^1J_{P–H}$ = 207 Hz).

^{119}Sn NMR (186 MHz, C_6D_6): δ (ppm) 40.4 (d, $^1J_{Sn–P}$ = 959 Hz).

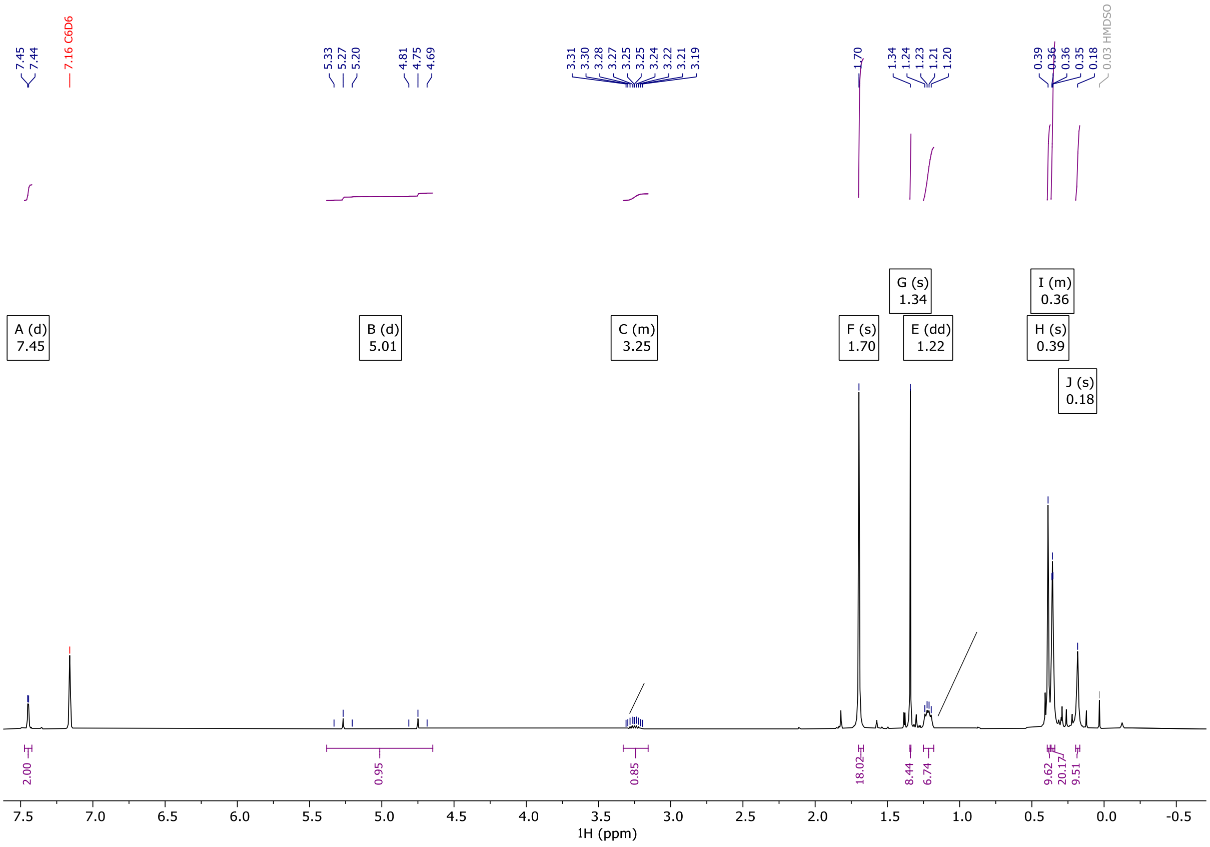

Figure S33. ^{1}H NMR spectrum (400 MHz, 293 K) of **5b** in C_6D_6.

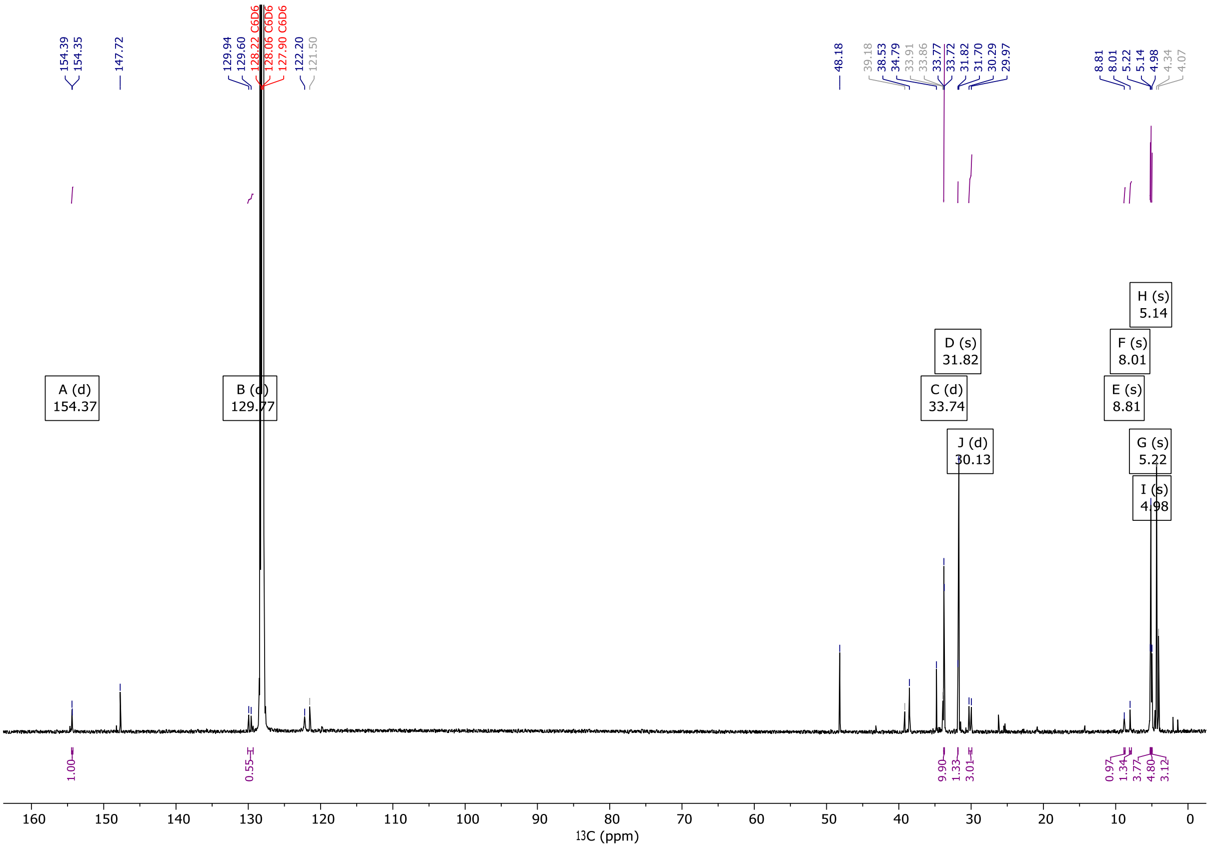

Figure S34. ^{13}C NMR spectrum (151 MHz, 293 K) of **5b** in C_6D_6.

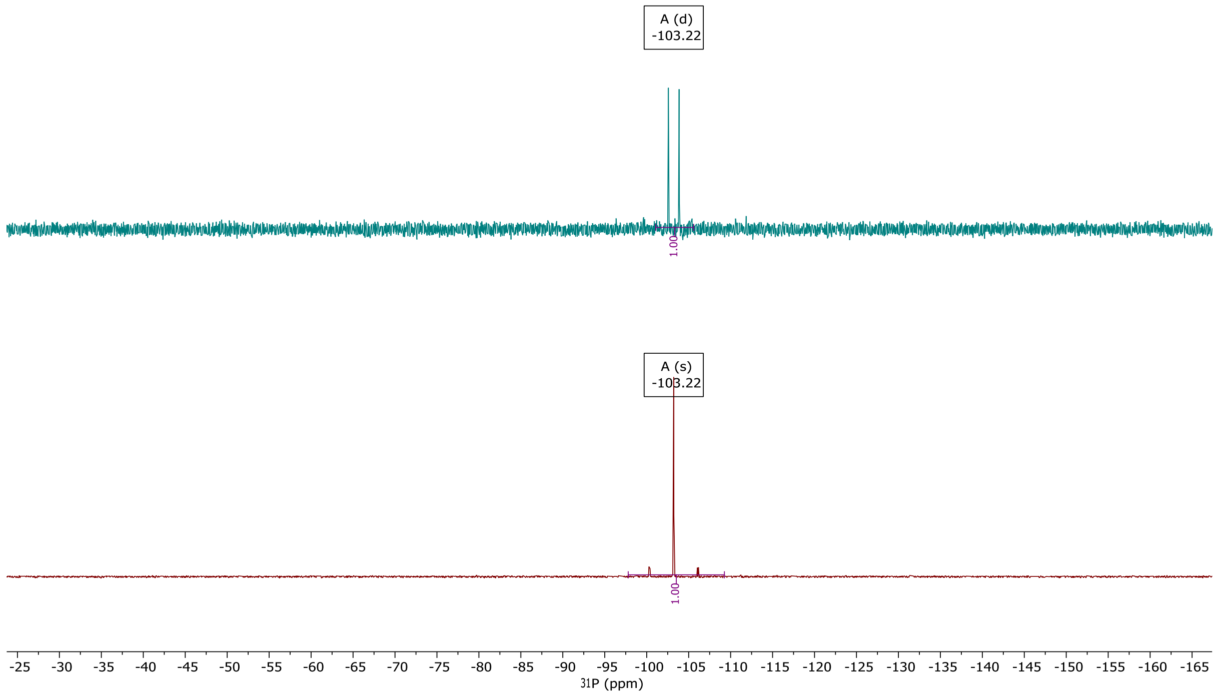

Figure S35. ^{31}P (top) and $^{31}P\{^{1}H\}$ (bottom) NMR spectra (162 MHz, 293 K) of **5b** in C_6D_6.

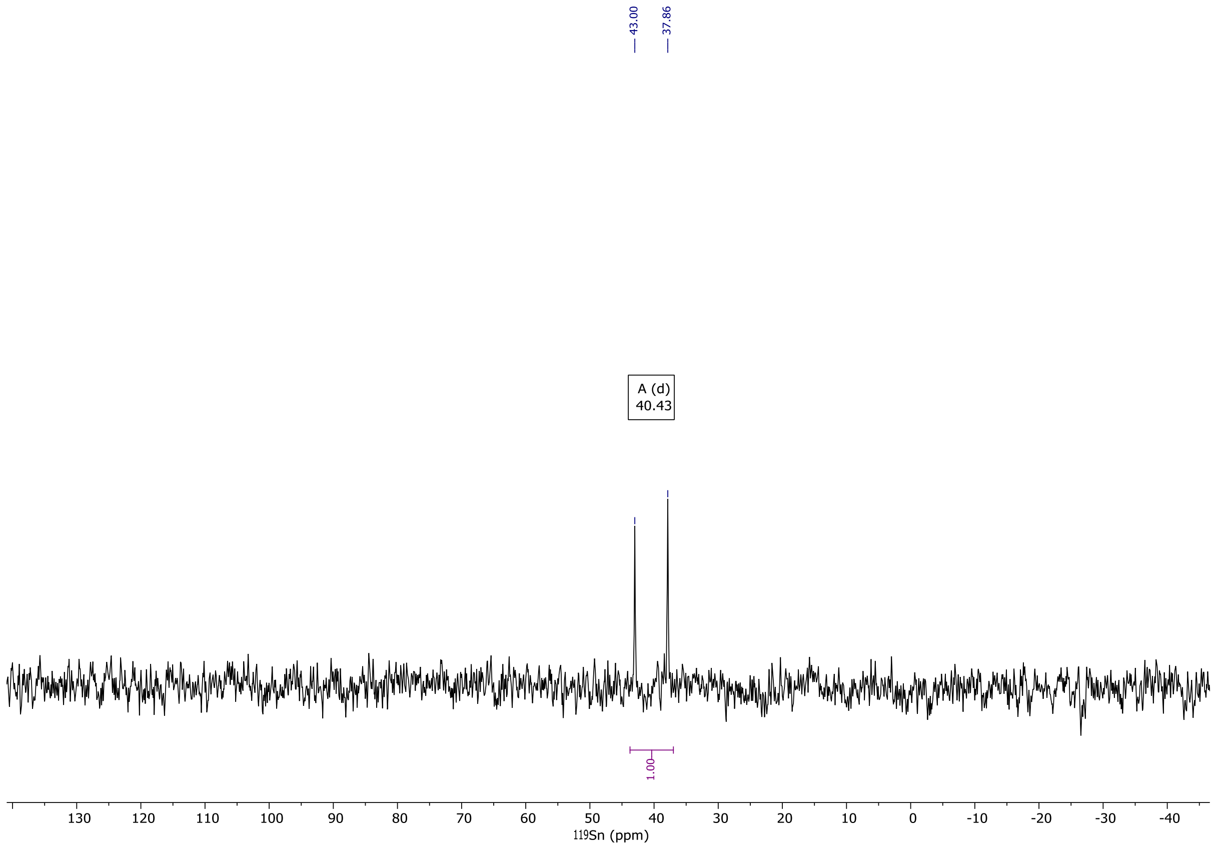

Figure S36. ^{119}Sn NMR spectrum (186 MHz, 293K) of **5b** in C_6D_6.

1.1.10 Synthesis of $[(Me_3Si)_2CH]_2Sn(NHC_6H_4OMe)P(H)Mes^*$ (6b)

1b (30 mg, 0.042 mmol) and *p*-anisidine (5.2 mg, 0.042 mmol) were dissolved in toluene (0.5 mL) and heated at 80 °C for 11 hours, causing the colour to fade to yellow/orange. The volatiles were removed *in vacuo* followed by extraction of the solids with pentane (0.5 mL × 3). A small amount of hexamethyldisiloxane (0.1 mL) was added. Slow evaporation of the pentane/hexamethyldisiloxane solution at room temperature yielded off-white crystals which were washed with cold HMDSO and dried *in vacuo* (10.2 mg, 0.012 mmol, 28.6% yield). Crystals suitable for X-ray diffraction were grown by slow evaporation of a pentane/HMDSO solution at room temperature. Anal. calculated for $C_{39}H_{76}N_1O_1P_1Si_4Sn_1$: C, 55.96; H, 9.15; N, 1.67. Found: C, 54.89; H, 8.81; N, 1.38.

^{1}H NMR (400 MHz, C_6D_6): δ (ppm) 7.45 (d, $^4J_{P–H}$ = 2.5 Hz, 2H; Mes*Ar*H*), 6.86 (m, 2H; *p*-anisidine *meta*-C*H*), 6.67 – 6.62 (m, 2H; *p*-anisidine *ortho*-C*H*), 5.68 – 4.96 (d, $^1J_{P–H}$ = 207.8 Hz, 1H; P*H*), 3.41 (s, 3H; *p*-anisidine OCH_3), 3.12 (s, 1H; SnN*H*), 1.62 (s, 18H; Mes* *ortho*-C(CH_3)$_3$), 1.31 (s, 9H; Mes* *para*-C(CH_3)$_3$), 0.39 (s, 2H; SnC*H*), 0.33 (s, 9H; Si(CH_3)$_3$), 0.28 (s, 18H; Si(CH_3)$_3$), 0.21 (s, 9H; Si(CH_3)$_3$).

^{13}C NMR (151 MHz, C_6D_6): δ (ppm) 155.63 (d, $^2J_{P–C}$ = 7.3 Hz; Mes* *ortho*-Ar*C*), 152.25 (*p*-anisidine Ar*C*OCH_3), 148.32 (Mes* *para*-Ar*C*), 145.36 (*p*-anisidine Ar*C*NH), 127.58 (d, $^1J_{P–C}$ = 50.0 Hz; Mes* *ipso*-Ar*C*), 122.54 (Mes* *meta*-Ar*C*), 117.89 (*p*-anisidine *ortho*-Ar*C*), 115.17 (*p*-anisidine *meta*-Ar*C*), 55.40 (*p*-anisidine O*C*H_3), 38.64 (Mes* *ortho*-*C*(CH_3)$_3$), 34.84 (Mes* *para*-*C*(CH_3)$_3$), 33.80 (d, $^4J_{P–C}$ = 7.2 Hz; Mes* *ortho*-C(*C*H_3)$_3$), 31.59 (Mes* *para*-C(*C*H_3)$_3$), 10.11 (Sn*C*H), 9.22 (Sn*C*H), 5.06 (m, Si(*C*H_3)$_3$), 4.79 (d, *J* = 9.9 Hz, Si(*C*H_3)$_3$).

^{31}P NMR (162 MHz, C_6D_6): δ (ppm) –117.9 (d, $^1J_{P–H}$ = 207 Hz).

^{119}Sn NMR (186 MHz, C_6D_6): δ (ppm) 22.4 (d, $^1J_{Sn–P}$ = 1010 Hz).

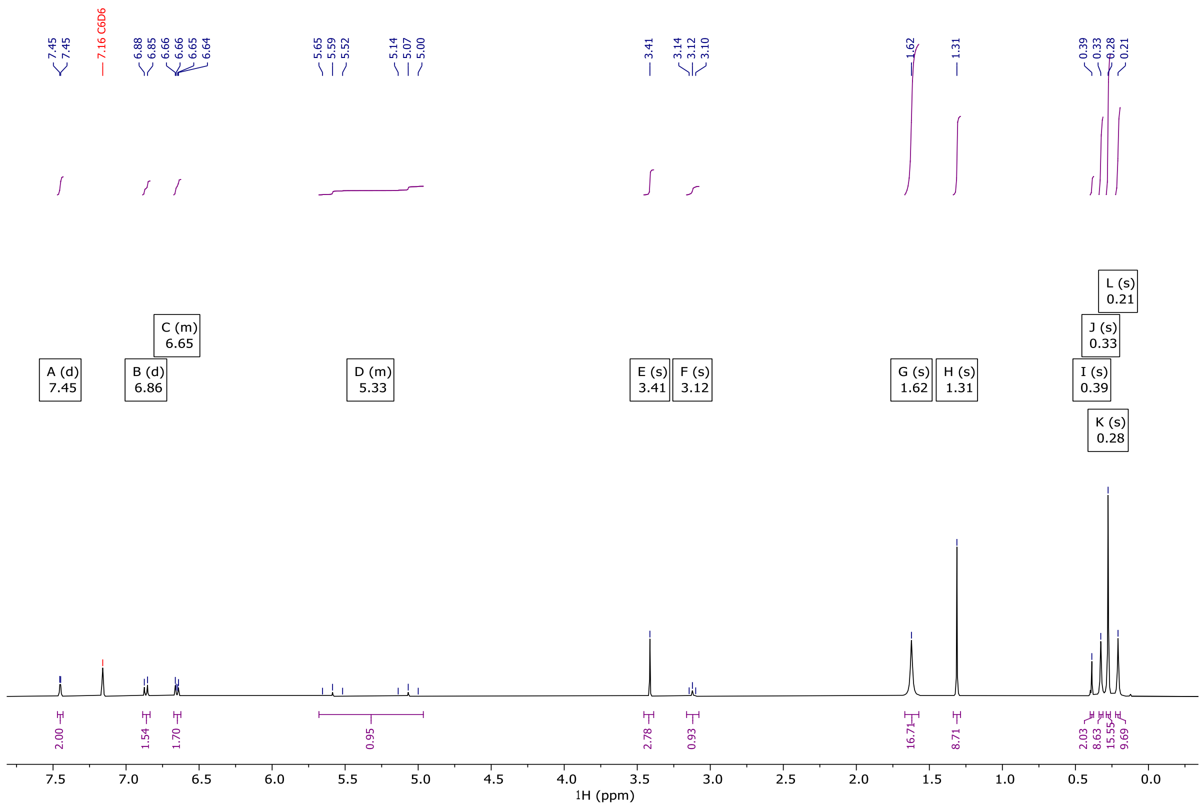

Figure S37. ^{1}H NMR spectrum (400 MHz, 293 K) of **6b** in C_6D_6.

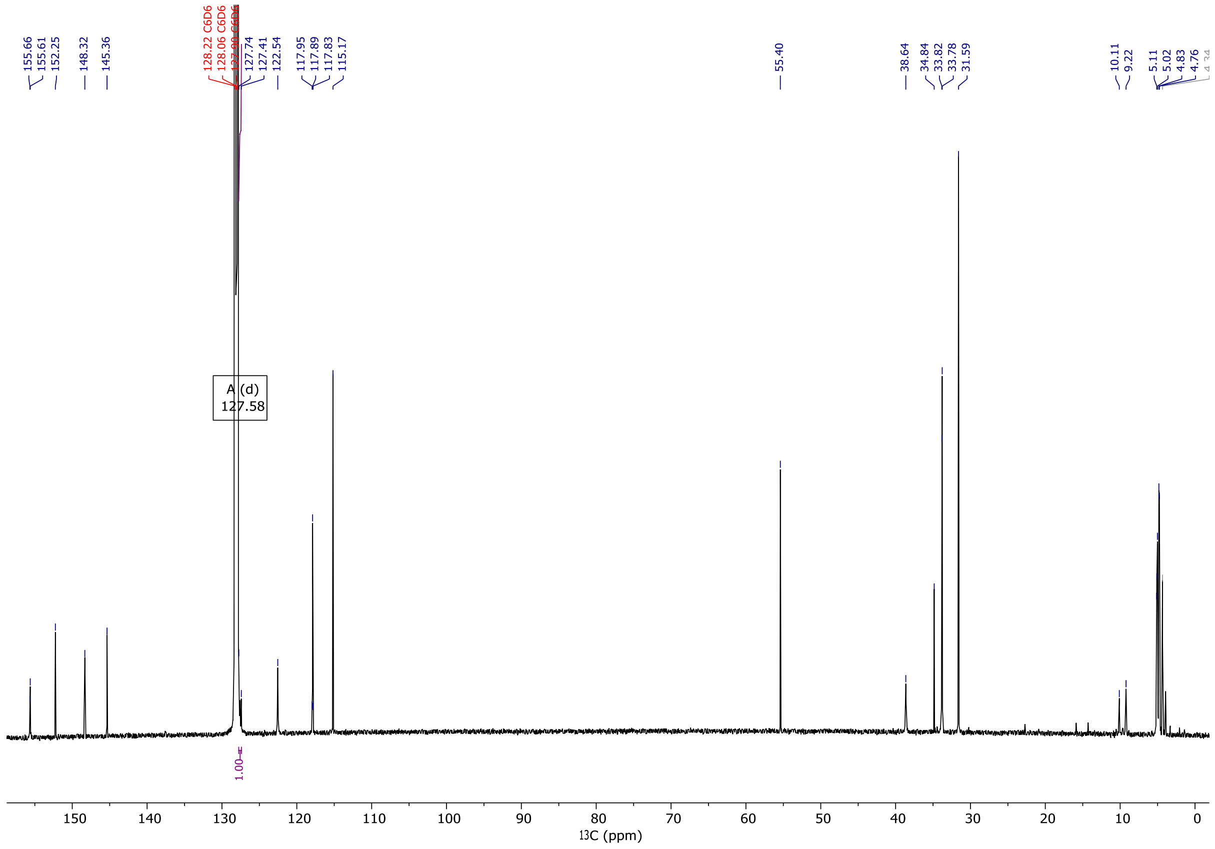

Figure S38. ^{13}C NMR spectrum (151 MHz, 293 K) of **6b** in C_6D_6.

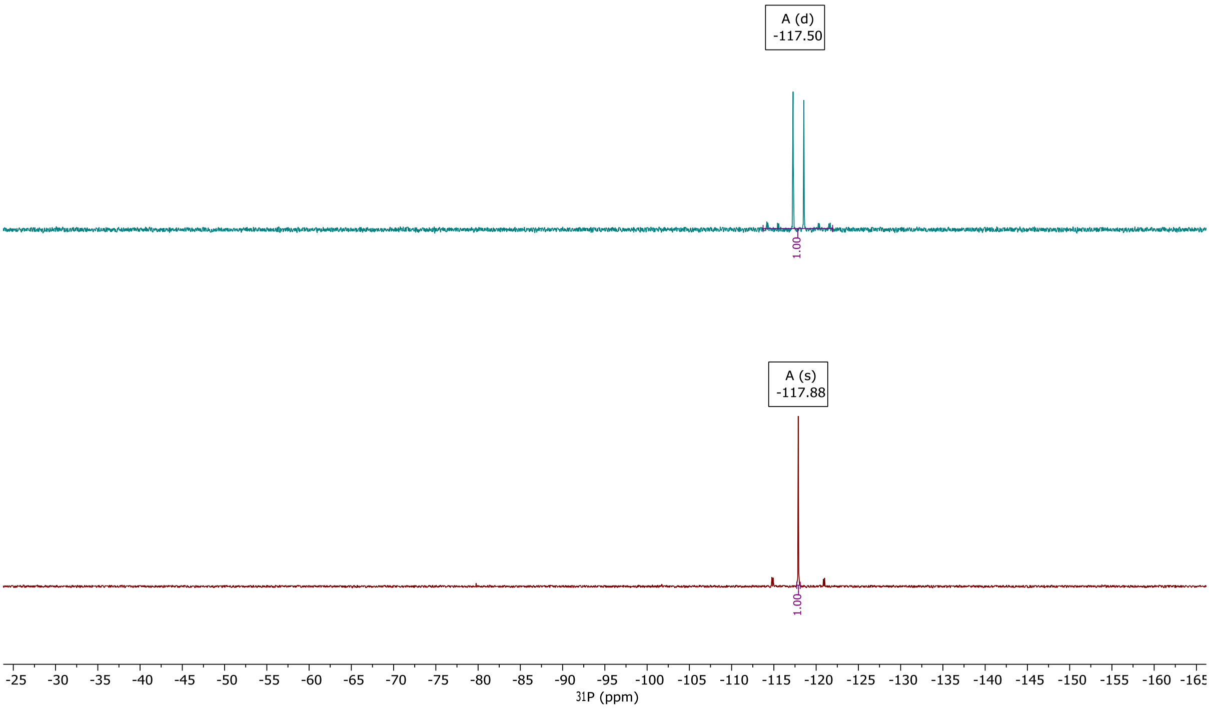

Figure S39. ^{31}P (top) and ^{31}P{^{1}H} (bottom) NMR spectra (162 MHz, 293 K) of **6b** in C_6D_6.

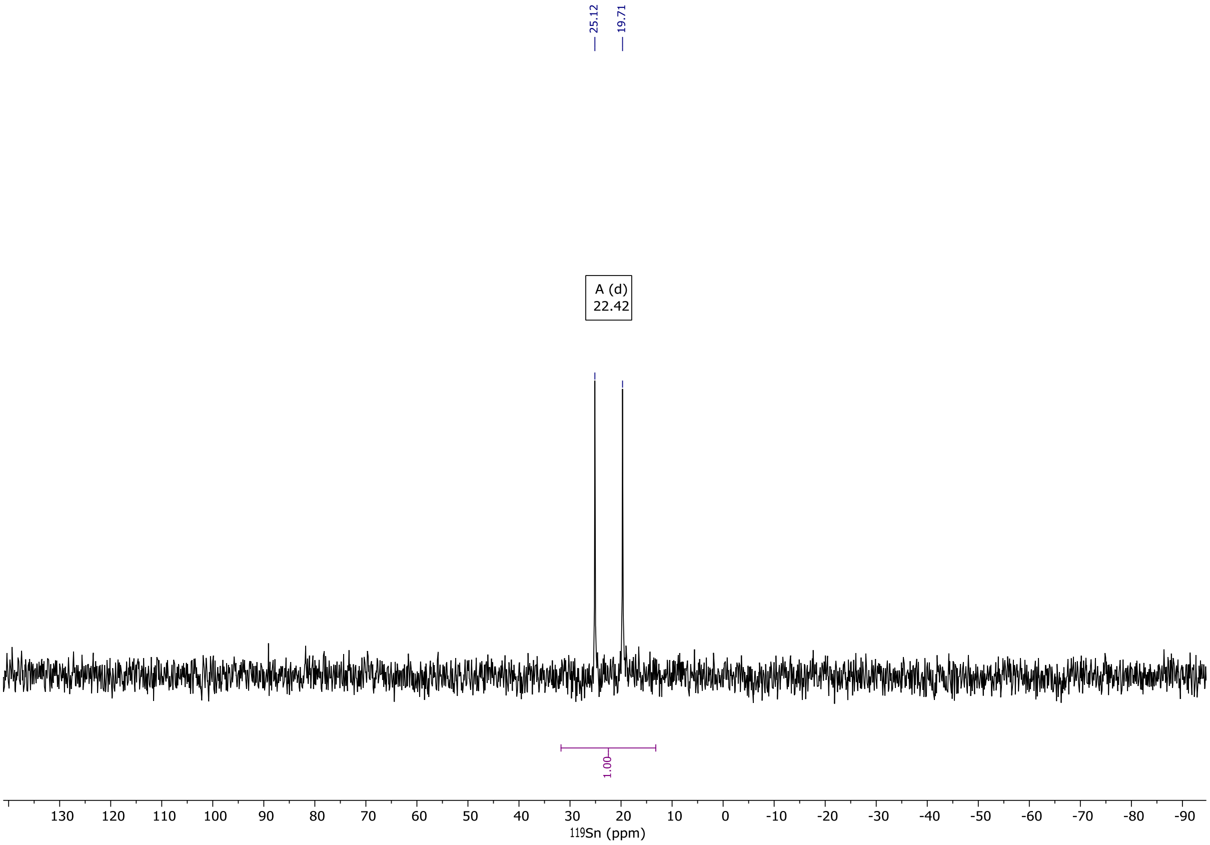

Figure S40. ^{119}Sn NMR spectrum (186 MHz, 293 K) of **6b** in C_6D_6.

1.1.11. Attempted synthesis of $[(Me_3Si)_2CH]_2Ge(C_3H_3N_2)P(H)Mes^*$ (7a)

The preparation of **7a** was successful in small scale experiments but attempts to repeat this synthesis on preparative scales to obtain data for characterisation were unsuccessful, resulting in a mixture of products with **7a** as a minor product (Figure S42).

Method A (NMR scale): **1a** (10 mg, 0.015 mmol) was dissolved in C_6D_6 in a J. Young NMR tube. Imidazole (1.5 mg, 0.022 mmol) was added and the solution heated at 80 °C for 14 days. The ^{31}P NMR spectrum was recorded without further purification, showing almost full conversion to **7a** in addition to small amount of unreacted **1a** (< 10%) (Figure S41). The volatiles were removed *in vacuo* followed by extraction of the solids with hexane (0.5 mL × 3). Crystals suitable for single crystal X-ray diffraction were grown from a concentrated hexane solution at room temperature, but too few could be isolated for a yield to be recorded.
^{31}P NMR (162 MHz, C_6D_6): δ (ppm) −89.1 (d, $^1J_{P-H}$ = 219 Hz).

Method B (Preparative scale): **1a** was prepared *in situ* by combining $Ge[CH(SiMe_3)_2]_2$ (75 mg, 0.192 mmol) and PMe_3–PMes* (67.5 mg, 0.192 mmol) in toluene (2 mL) and stirring until all of the solids dissolved. The volatiles were removed *in vacuo* and the solid residue redissolved in toluene (2 mL). Imidazole (13 mg, 0.191 mmol) was then added and the solution heated at 80 °C for 14 days. An aliquot (0.5 mL) of the solution was taken for ^{31}P{^{1}H} NMR spectroscopy, which revealed the formation of a mixture of products including Mes*P=PMes* and Mes^*PH_2, in addition to small amounts of **7a** and incomplete consumption of **1a**.

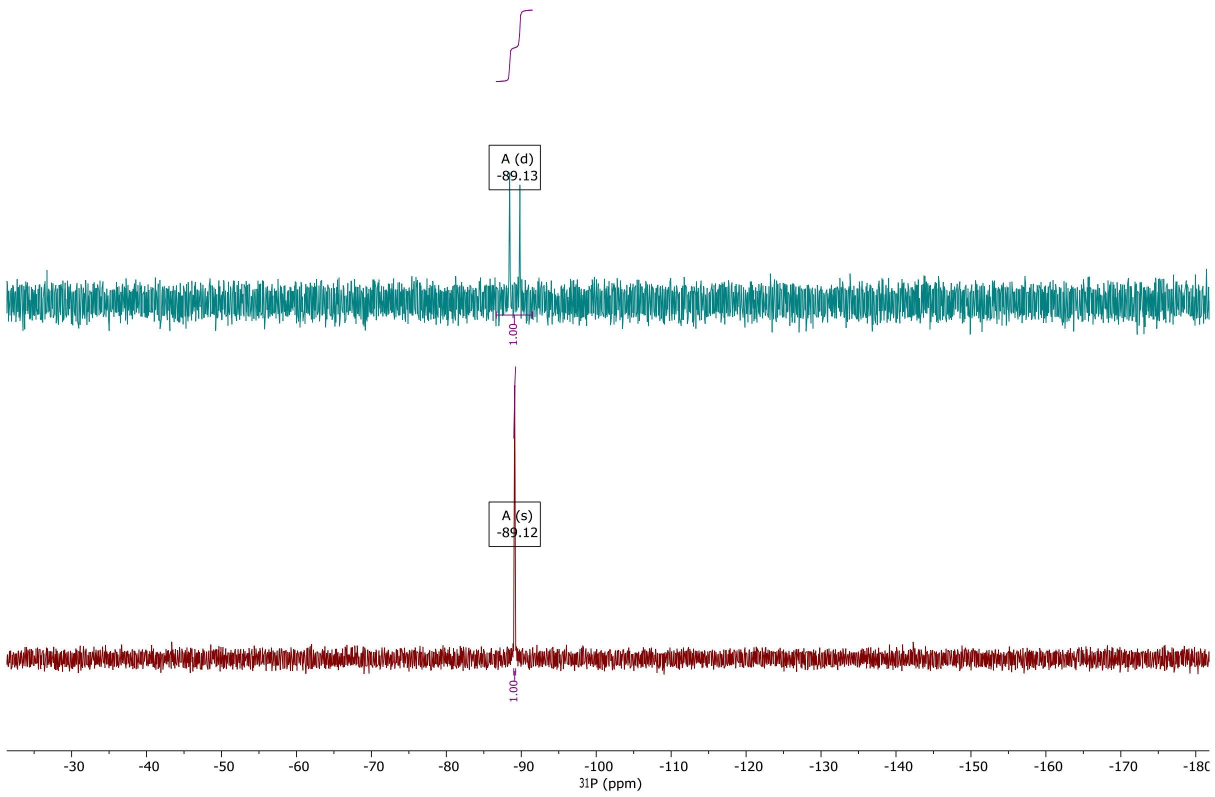

Figure S41. $^{31}P\{^1H\}$ and ^{31}P (inset) NMR spectra (162 MHz, 293 K) of **7a** in C_6D_6 after 14 days at 80 °C, according to Method A.

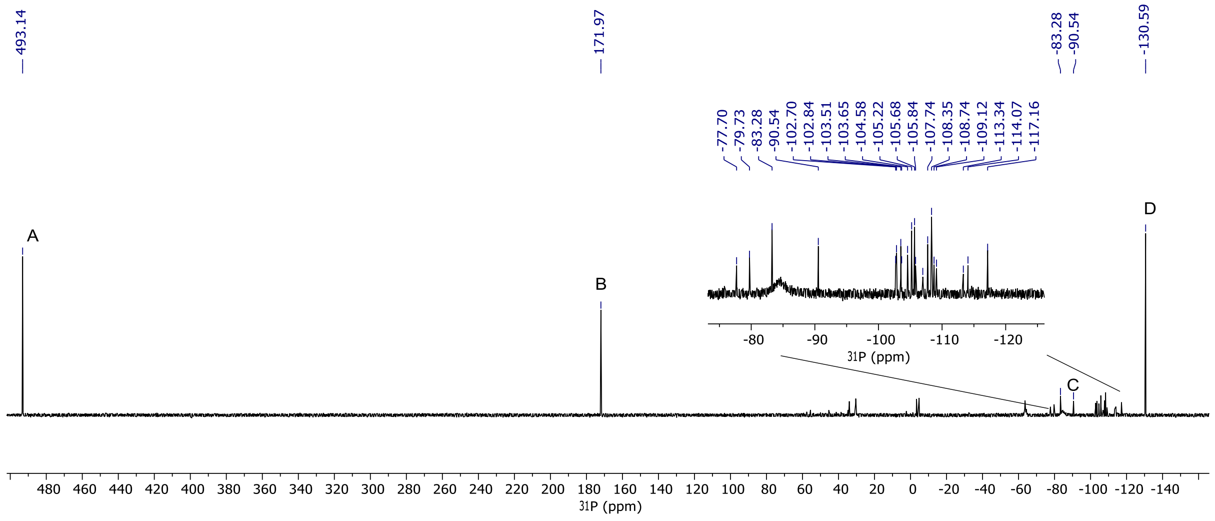

Figure S42. $^{31}P\{^1H\}$ NMR spectrum (162 MHz, 293 K) of the reaction between *in-situ* generated **1a** and imidazole in C_7H_8 (A: Mes*P=PMes*; B: **1a**; C: **7a**; D: Mes*PH_2).

1.1.12. Attempted synthesis of $[(Me_3Si)_2CH]_2Sn(C_3H_3N_2)P(H)Mes^*$ (7b)

Despite repeated attempts, the isolation of a product proposed as **7b** on the basis of NMR spectroscopy was unsuccessful as it appears to decompose slowly at room temperature and more rapidly at elevated temperatures to form **8**. The mechanism of this process was probed by control experiments (*vide infra*).

Method A (NMR scale): **1b** (10 mg, 0.014 mmol) was combined in an NMR tube with an excess of imidazole (3 mg, 0.044 mmol) and dissolved in C_6D_6. The solution immediately decolourised. The ^{31}P NMR spectrum of the solution was taken without further purification (Figure S41). Heating the solution at 80 °C for 4 hours results in complete conversion to **8** by ^{31}P{^{1}H} NMR spectroscopy.

^{31}P NMR (162 MHz, C_6D_6): δ (ppm) −101.7 (d, $^{1}J_{P-H}$ = 209 Hz).

Method B (Preparative scale): **1b** (30 mg, 0.042 mmol) was dissolved in toluene (1 mL). 0.5 mL of a freshly prepared imidazole solution in DFB (5.7 mg mL^{-1}, 0.042 mmol) was added dropwise and the solution immediately decolourised. Isolation of the product was attempted by removal of the volatiles *in vacuo* followed by extraction of the solids with *n*-pentane (1 mL × 3). A small amount of hexamethyldisiloxane (0.1 mL) was added. Slow evaporation of the pentane/hexamethyldisiloxane solution at room temperature yielded colourless solids which were washed with a small amount of cold HMDSO (Yield: 7.0 mg). The NMR spectra of the solids showed **8** as the primary product along with smaller amounts of **1b** and Mes*PH_2.

The isolation of clean samples of **7b** was repeatedly attempted but in each case, a mixture containing several other species were formed upon crystallisation; the composition of the mixture differed markedly from those obtained from *in situ* NMR measurements prior to attempted purification. The decomposition of **7b** upon standing at room temperature over several months was further investigated (see Method C).

Method C (room temperature decomposition): **1b** (10 mg, 0.014 mmol) and imidazole (10 mg, 0.147 mmol, in excess) were combined in C_6D_6. After shaking for 30 seconds, the solution decolourised. The ^{31}P{^{1}H} NMR spectrum was then recorded without further purification. The mixture was left to stand at room temperature for 9 days. The ^{1}H{^{119}Sn} HMBC and ^{31}P{^{1}H} spectra were then recorded, revealing a mixture of compounds including **4b*** and an unknown tin-containing decomposition product. The mixture was then left to stand at room temperature

for a further 50 days and monitored by $^{31}P\{^{1}H\}$ and ^{1}H NMR spectroscopy, initially revealing the slow conversion of **7b** to **8** followed by gradual decomposition to an intractable mixture of products, including $CH_2(SiMe_3)_2$ and Mes*PH_2.

*A small amount of **4b** was presumably formed through activation of adventitious water.

^{119}Sn NMR (186 MHz, C_6D_6): δ(ppm) −16.4 (s; unknown), 42.0 (d; unknown), 80.7 (s (br); **4b**).

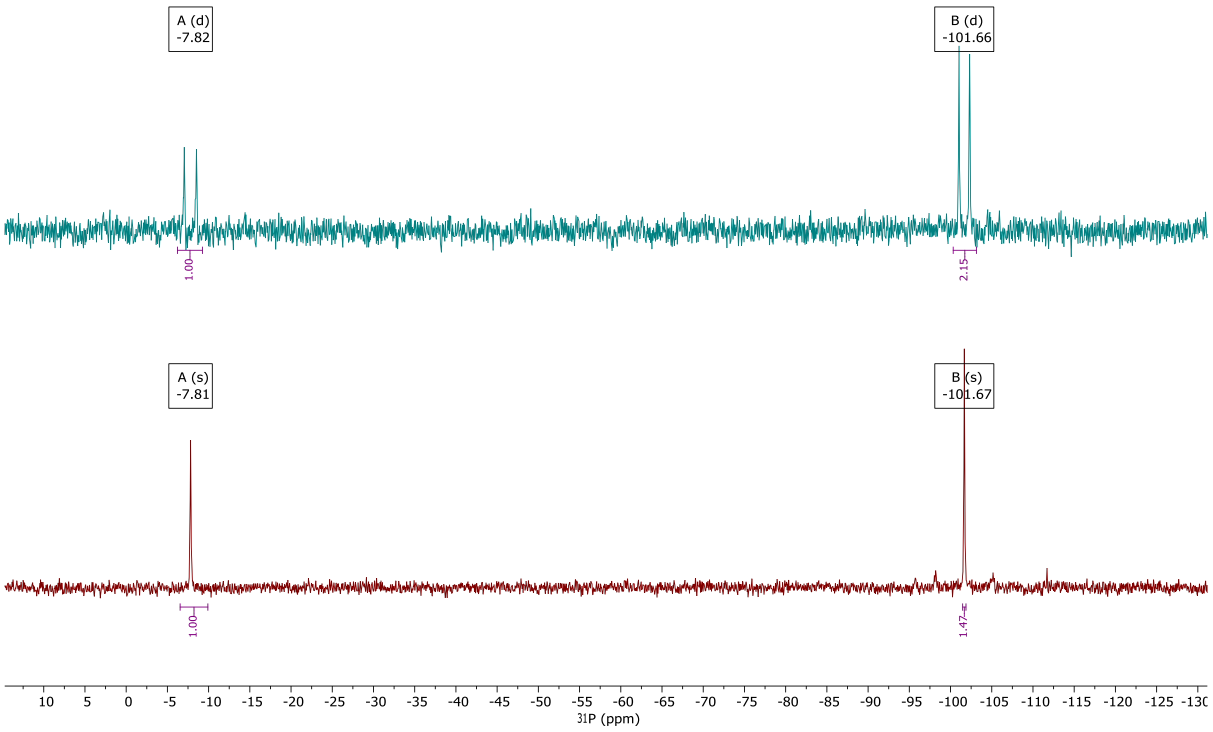

Figure S43. ^{31}P (top) and $^{31}P\{^{1}H\}$ (bottom) NMR spectra (162 MHz, 293 K) of a solution of **1b** in C_6D_6 immediately after the addition of imidazole according to Method A (A: **8**; B: **7b**).

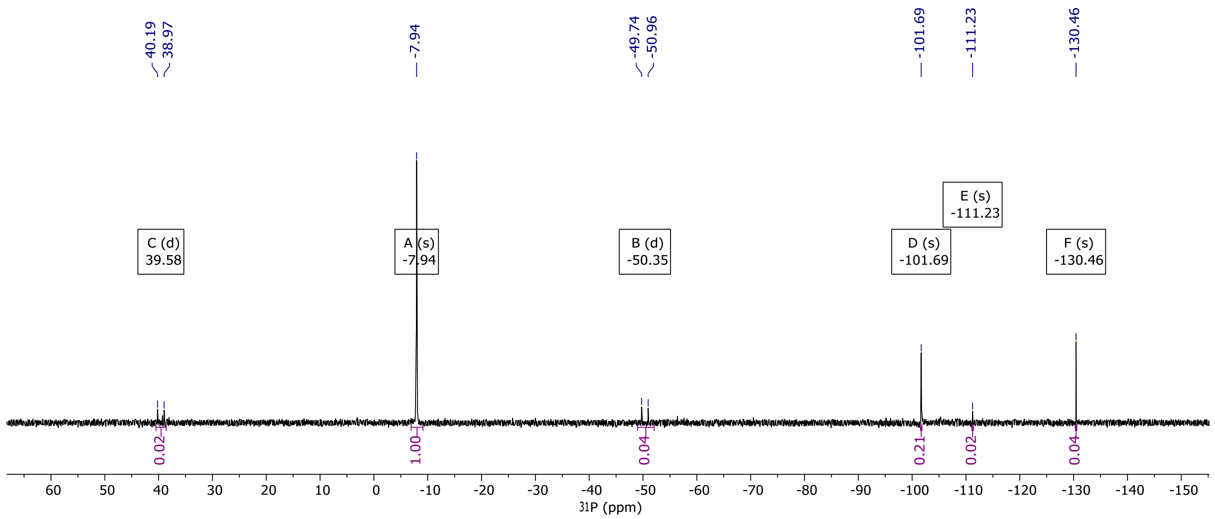

Figure S44. $^{31}P\{^{1}H\}$ NMR spectrum (162 MHz, 293 K) in C_6D_6 of the crystals obtained following recrystallisation of the reaction between **1b** and a DFB solution of imidazole in toluene. Similar mixtures were obtained for each of the attempts to synthesise pure samples of **7b** (A: **8**; D: **7b**; E: **4b**; F: Mes*PH_2).

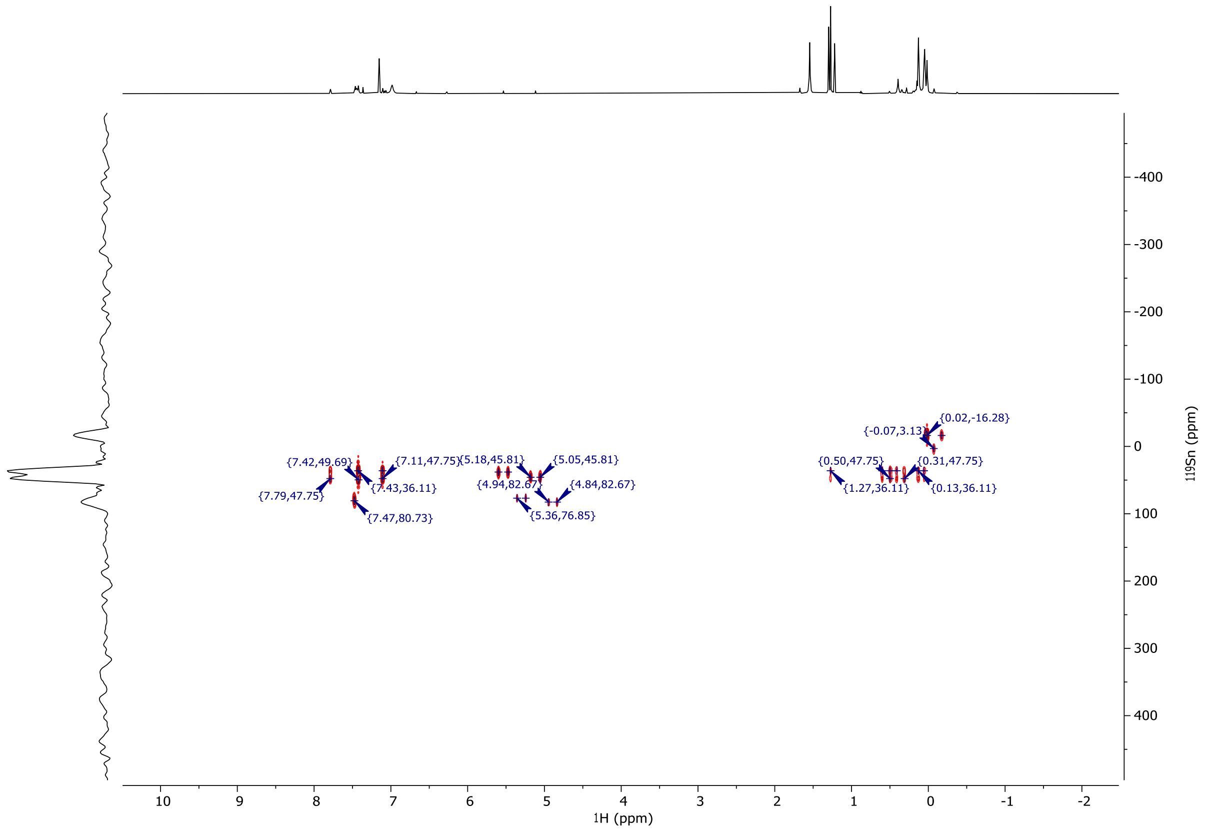

Figure S45. $^{1}H\{^{119}Sn\}$ HMBC spectrum (186 MHz, 293 K) in C_6D_6 of the reaction between **1b** and excess imidazole, recorded after 9 days at room temperature.

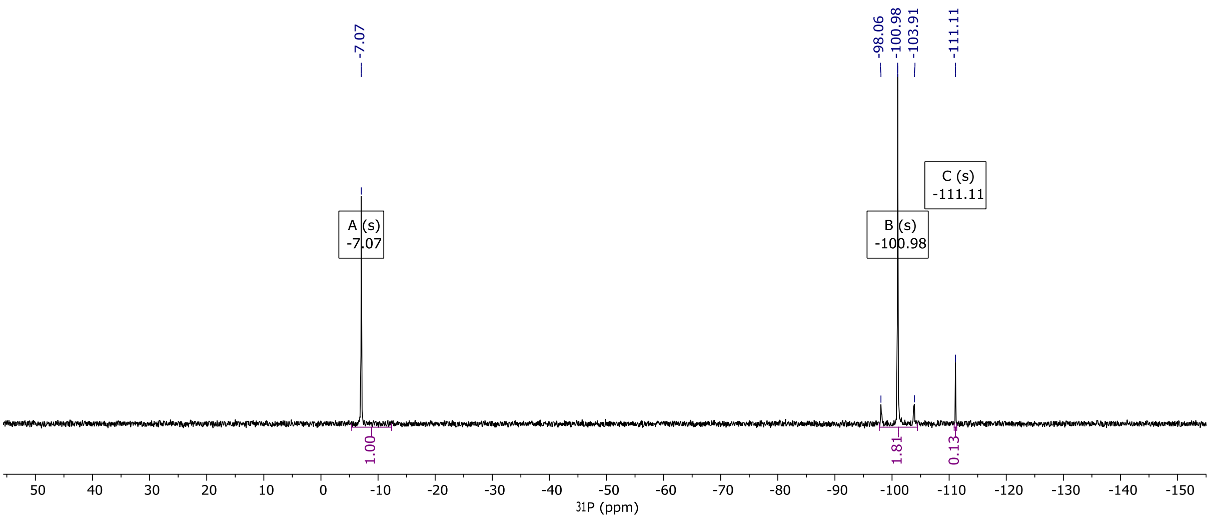

Figure S46. $^{31}P\{^{1}H\}$ NMR spectrum (202 MHz, 293K) of the reaction between **1b** and excess imidazole in C_6D_6 recorded after 9 days at room temperature.

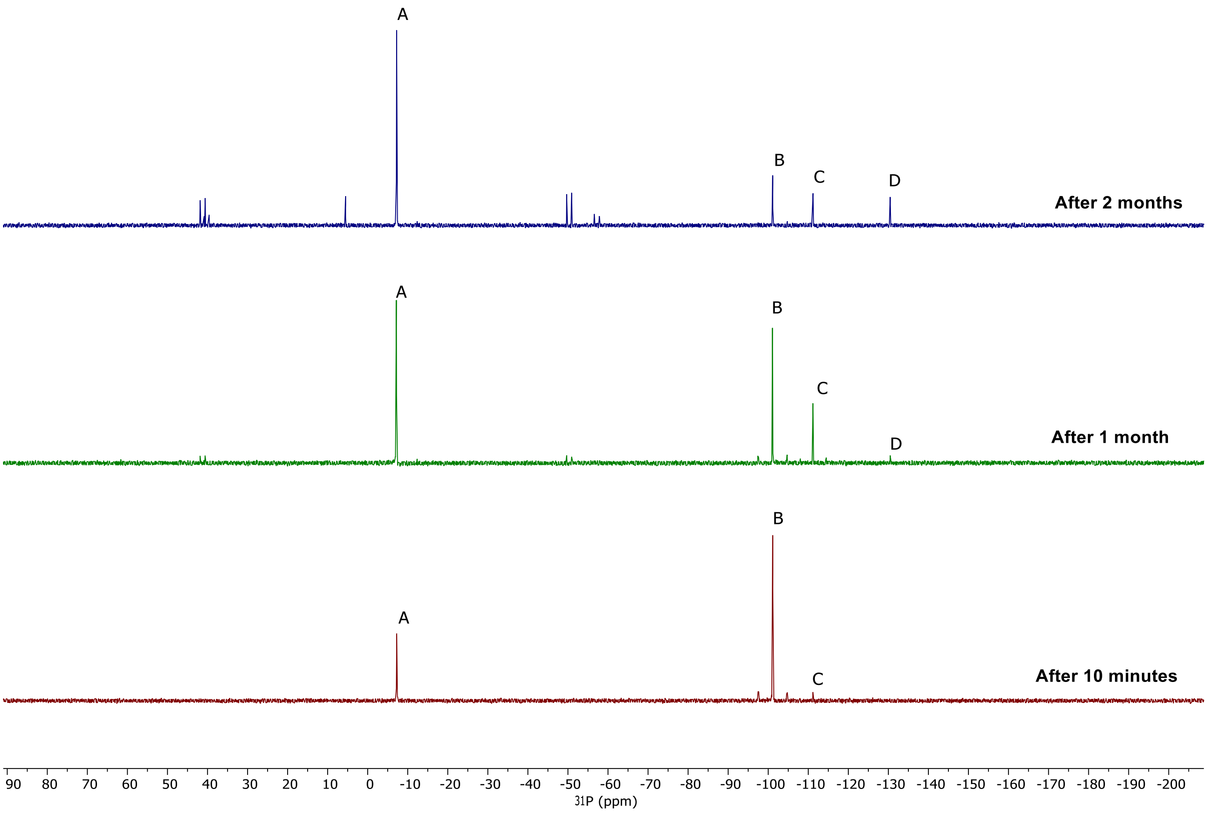

Figure S47. $^{31}P\{^{1}H\}$ NMR spectra (162 MHz, 293 K) of the reaction between **1b** and excess imidazole in C_6D_6 (A: **8**; B: **7b**; C: **4b**; D: Mes*PH_2).

1.1.13. Synthesis of Mes*P(H)[N(CH)$_2$NCH] (8)

Me_3P–PMes* (40 mg, 0.114 mmol) and imidazole (7.8 mg, 0.115 mmol) were combined in toluene and sonicated at room temperature for 30 minutes. Removal of the volatiles *in vacuo* followed by recrystallisation from hexane yielded colourless crystals which were suitable for X-ray diffraction (19.5 mg, 0.057 mmol, 50% yield). Anal. calculated for $C_{21}H_{33}N_2P_1$: C, 73.22; H, 9.66; N, 8.13. Found: C, 72.58; H, 10.18; N, 8.14.

^{1}H NMR (400 MHz, C_6D_6): δ (ppm) 7.47 (d, $^4J_{P–H}$ = 2.7 Hz, 2H; Mes* ArC*H*), 6.91 (d, J = 238.6 Hz, 1H; P*H*), 6.32 – 6.29 (m, 1H; NC*H*N(CH)$_2$), 1.31 (s, 18H; Mes* *ortho*-C(C*H*$_3$)$_3$), 1.23 (s, 9H; Mes* *para*-C(C*H*$_3$)$_3$). Imidazole C4 and C5 protons were observed in 2D spectra but were not assigned in 1D spectra due to overlap with the residual solvent peak.

^{13}C NMR (151 MHz, C_6D_6): δ (ppm) 156.49 (d, $^2J_{P–C}$ = 8.8 Hz; Mes* *ortho*-Ar*C*), 152.26 (Mes* *para*-Ar*C*), 141.62 (d, $^2J_{P–C}$ = 11.3 Hz; imidazole *C*5), 131.31 (d, $^3J_{P–C}$ = 3.3 Hz; imidazole *C*4), 127.54 (d, $^1J_{P–C}$ = 27.0 Hz; Mes* *ipso*-Ar*C*), 123.08 (br; Mes* *meta*-Ar*C*), 122.07 (d, $^2J_{P–C}$ = 7.9 Hz; imidazole *C*2), 38.45 (br; Mes* *ortho*-*C*(CH$_3$)$_3$), 35.11 (Mes* *para*-*C*(CH$_3$)$_3$), 33.57 (d, $^4J_{P–C}$ = 7.3 Hz; Mes* *ortho*-C(*C*H$_3$)$_3$), 31.23 (Mes* *para*-C(*C*H$_3$)$_3$).

^{31}P NMR (162 MHz, C_6D_6): δ (ppm) −8.0 (d, $^1J_{P–H}$ = 239 Hz).

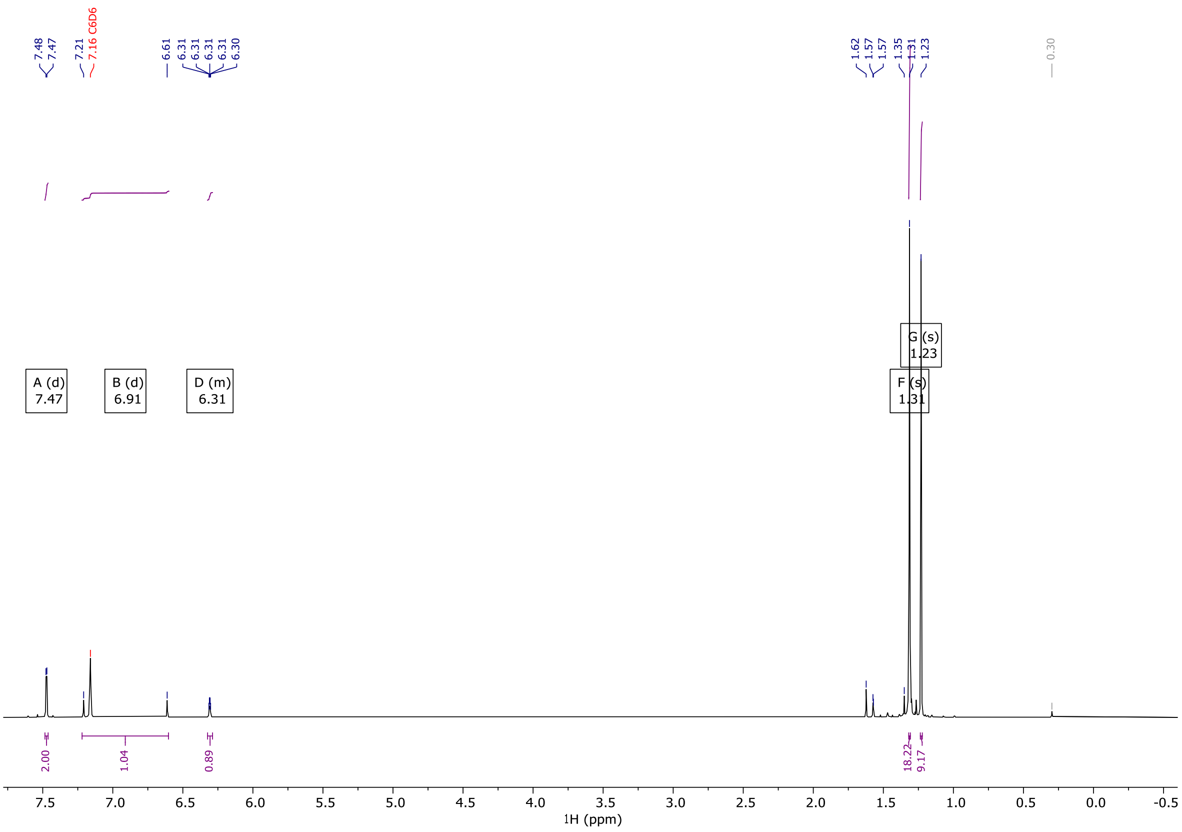

Figure S48. ^{1}H NMR spectrum (400 MHz, 293 K) of **8** in C_6D_6.

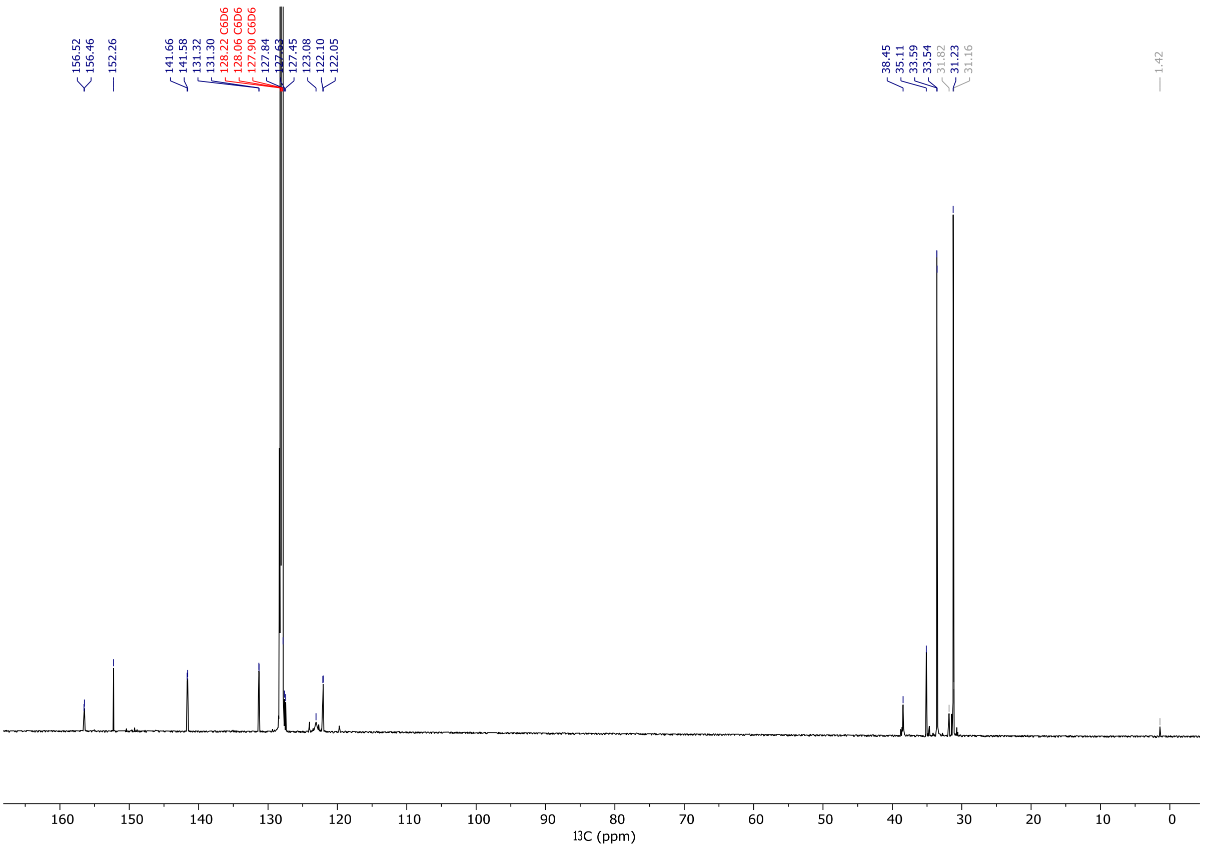

Figure S49. ^{13}C NMR spectrum (151 MHz, 293 K) of **8** in C_6D_6.

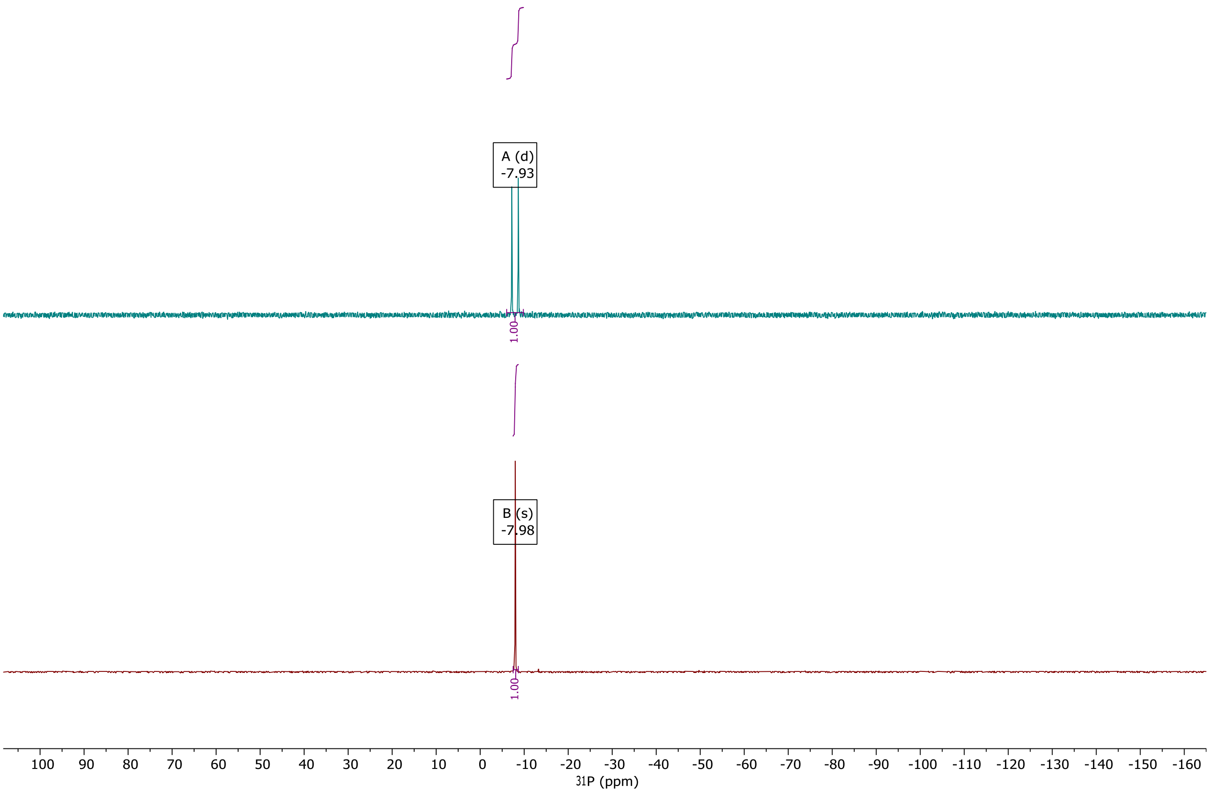

Figure S50. ^{31}P (top) and $^{31}P\{^1H\}$ (bottom) NMR spectra (162 MHz, 293 K) of **8** in C_6D_6.

1.1.14. The reaction between 1b and $PhSiH_3$

1b (10 mg, 0.014 mmol) was dissolved in toluene (0.4 mL). $PhSiH_3$ (0.2 mL, 1.623 mmol, in excess) was then added and the mixture heated at 80 °C for 3 days, during which time the colour changed from red to pale brown. The volatiles were removed *in vacuo* to yield a dark brown oil which was redissolved in C_6D_6. The ^{31}P NMR spectrum was recorded without further purification. The formation of a mixture of Mes*P=PMes*, $Mes*PH_2$, 3,3-dimethyl-5,7-di-*tert*-butylphosphaindane, and a product assigned as $Mes*P(H)(SiH_2Ph)$ was observed.

^{31}P (162 MHz, C_6D_6): δ (ppm) –147.6 (dm, $^1J_{P\text{–}H}$ = 215 Hz; $P(H)(SiH_2Ph)$).

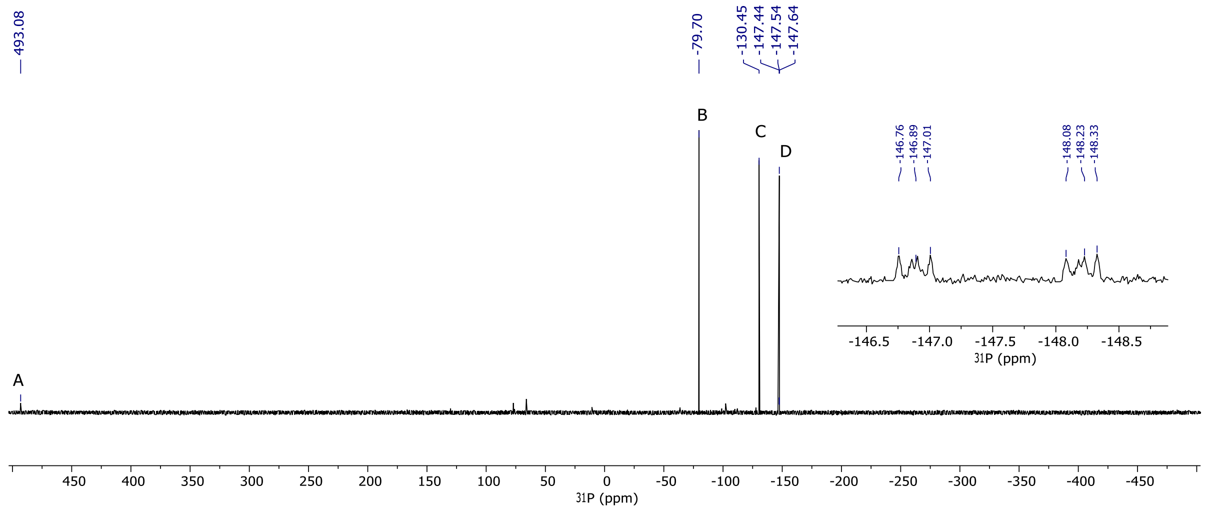

Figure S51. $^{31}P\{^1H\}$ and ^{31}P (inset) NMR spectra (162 MHz, 293 K) of the reaction between **1b** and $PhSiH_3$ in C_6D_6 after heating to 80 °C for 3 days (A: Mes*P=PMes*; B: 3,3-dimethyl-5,7-di-*tert*-butylphosphaindane; C: $Mes*P(H)(SiH_2Ph)$; D: $Mes*PH_2$).

1.1.15. Generation of PMes* from thermolysis or photolysis of 1b

Method A: **1b** (10 mg, 0.014 mmol) was dissolved in an NMR tube in C_6D_6 and heated to 80 °C. The $^{31}P\{^1H\}$ NMR spectrum of the reaction was recorded after 6 hours. The transient generation of the phosphinidine [Mes*P] upon heating was observed by the formation of the known decomposition product 3,3-dimethyl-5,7-di-*tert*-butylphosphaindane.

Method B: **1b** (10 mg, 0.014 mmol) was dissolved in C_6D_6 in a quartz J-Young's NMR tube and irradiated for 20 minutes by a 125 W mercury lamp, during which time the colour changed to bright purple. The NMR spectra of the reaction were then recorded without further purification, showing the formation of $Sn[CH(SiMe_3)_2]_2$ and 3,3-dimethyl-5,7-di-*tert*-butylphosphaindane.

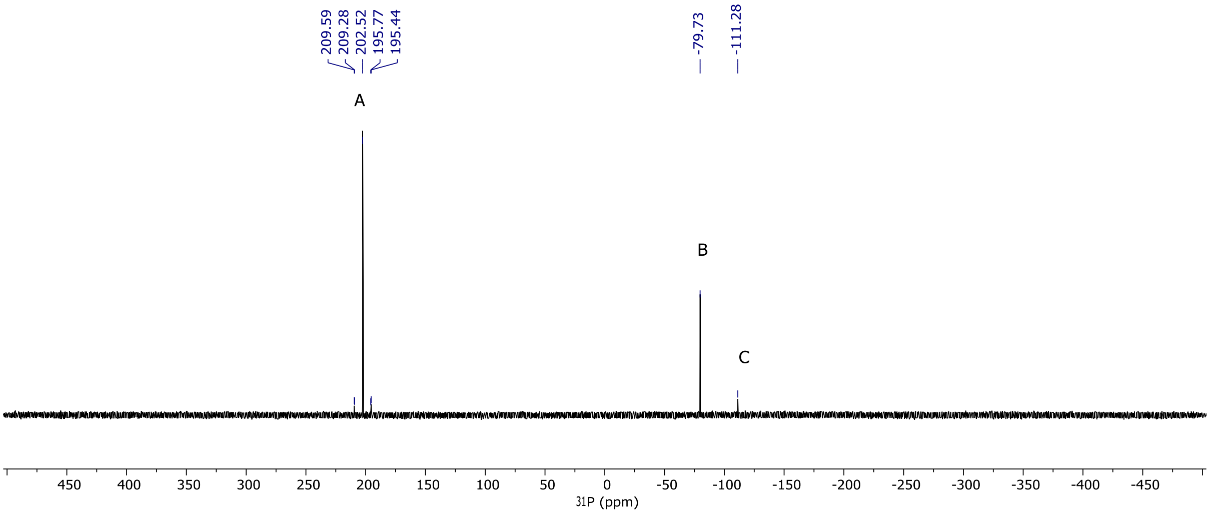

Figure S52. $^{31}P\{^{1}H\}$ NMR spectrum (162 MHz, 293 K) in C_6D_6 after heating **1b** at 80 °C for 6 hours (A: **1b**; B: 3,3-dimethyl-5,7-di-*tert*-butylphosphaindane; C: **4b**).

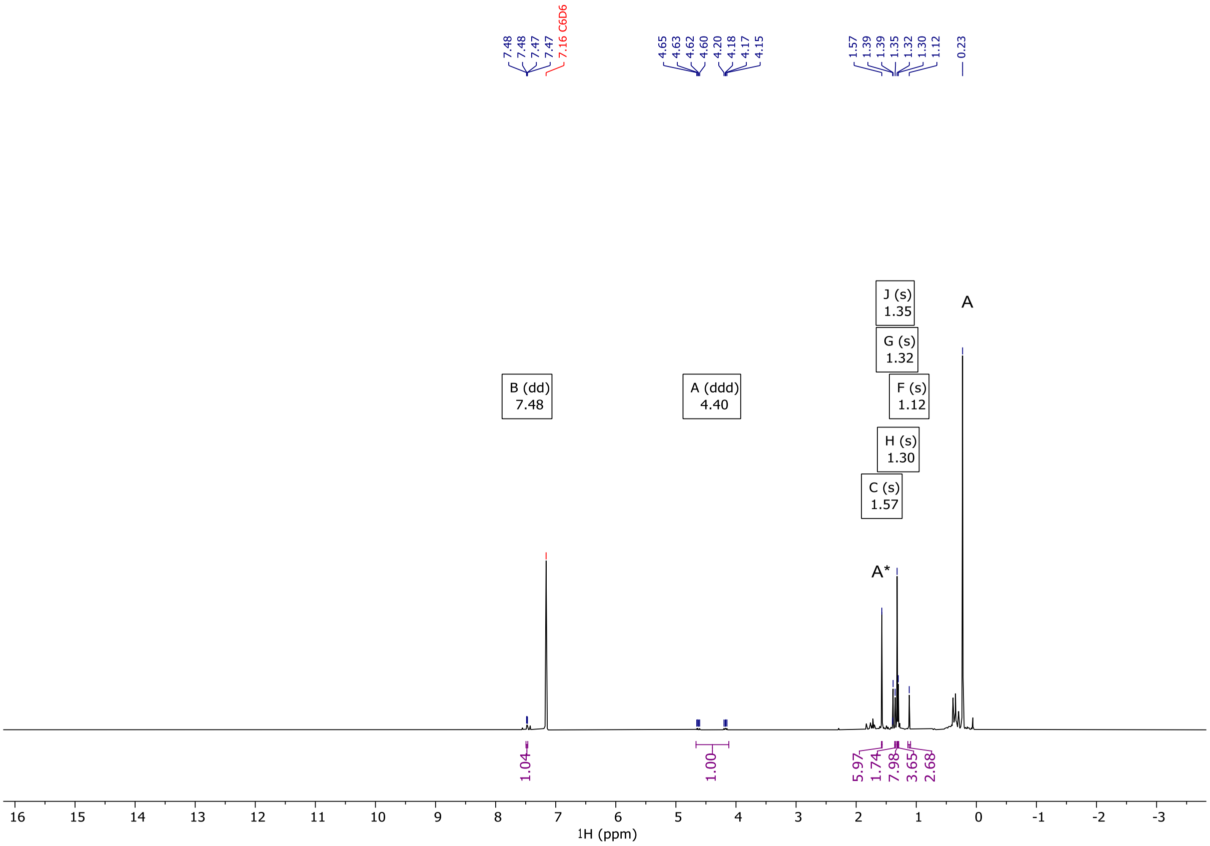

Figure S53. ^{1}H NMR spectrum (400 MHz, 293 K) after irradiation of **1b** with a 125W mercury lamp for 20 minutes in C_6D_6. (A: $Sn[CH(SiMe_3)_2]_2$). *Resonance overlaps with $ArP(H)CH_2C(CH_3)_2$ at 1.57 ppm.

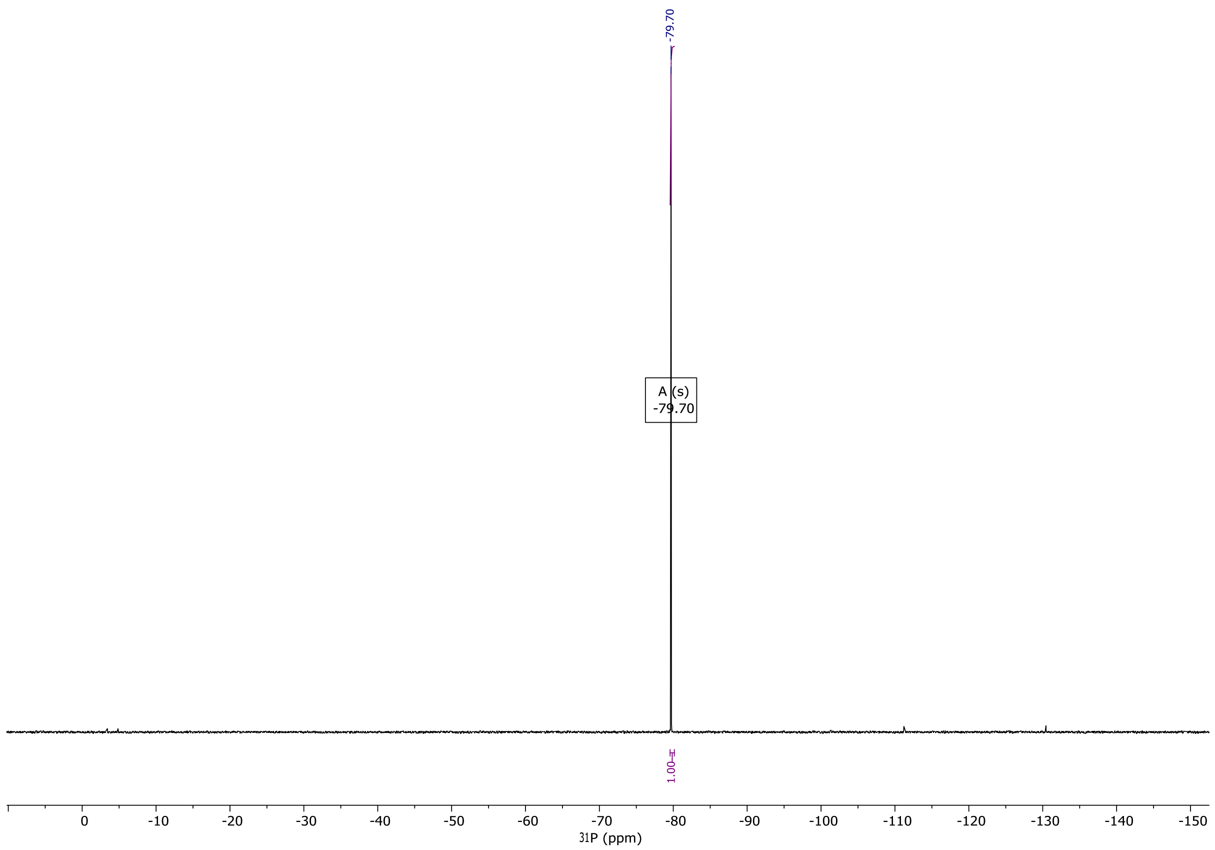

Figure S54. $^{31}P\{^{1}H\}$ NMR spectrum (162 MHz, 293 K) after irradiation of **1b** with a 125W mercury lamp for 20 minutes in C_6D_6.

1.1.16. Generation of PMes* from photolysis of 1a

1a (10 mg, 0.015 mmol) was dissolved in C_6D_6 in a quartz J-Young's NMR tube and irradiated for 20 minutes by a 125 W mercury lamp. The NMR spectra of the reaction were then recorded without further purification, showing the partial formation of $Ge[CH(SiMe_3)_2]_2$ and 3,3-dimethyl-5,7-di-*tert*-butylphosphaindane along with remaining **1a**. The solution was then irradiated for a further 105 minutes, with $^{31}P\{^{1}H\}$ NMR showing full consumption of **1a**.

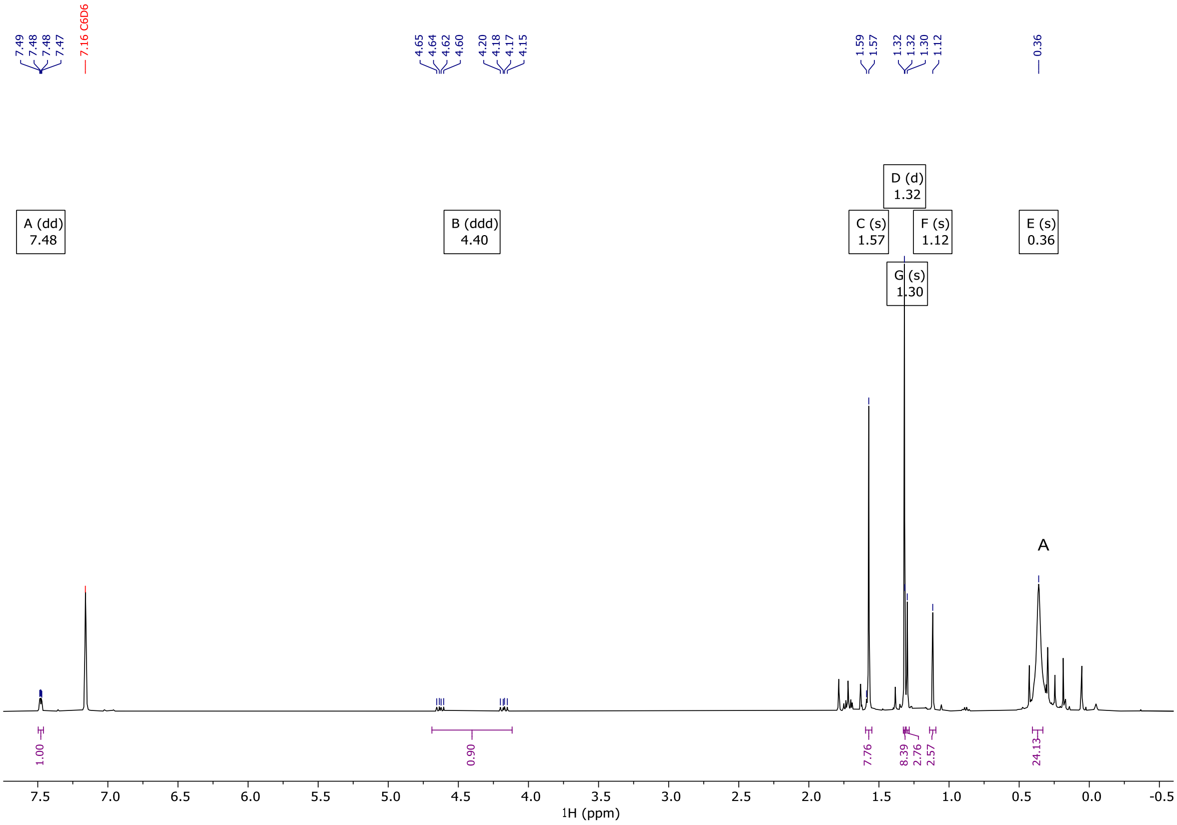

Figure S55. ^{1}H NMR spectrum (400 MHz, 293 K) after irradiation of **1a** with a 125W mercury lamp for 20 minutes in C_6D_6. (A: $Ge[CH(SiMe_3)_2]_2$).

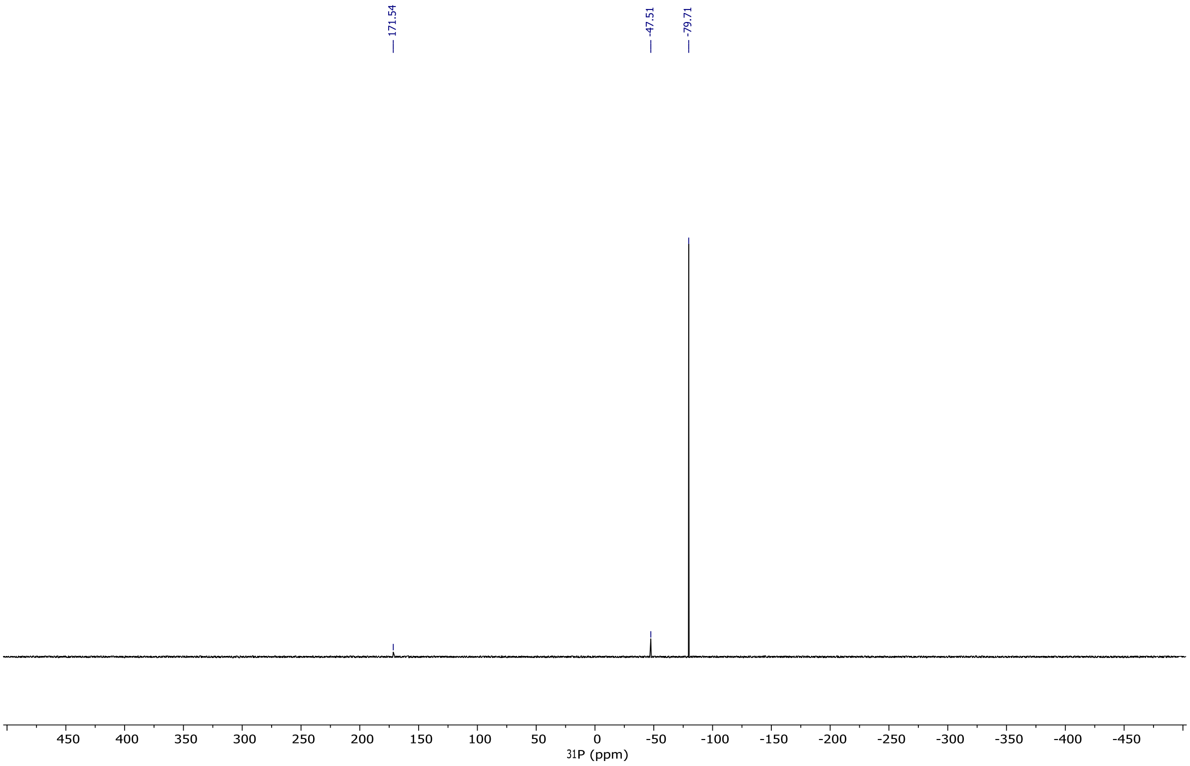

Figure S56. $^{31}P\{^{1}H\}$ NMR spectrum (162 MHz, 293 K) after irradiation of **1a** with a 125W mercury lamp for 125 minutes in C_6D_6.

1.1.17. Reaction of 1b with IMe_4 to form Mes*P–IMe_4

1b (10 mg, 0.014 mmol) and IMe_4 (7.8 mg, 0.063 mmol, in excess) were dissolved in C_6D_6. The mixture immediately became yellow/orange. $^{31}P\{^1H\}$ NMR spectroscopy revealed the formation of the previously reported Mes*P–IMe_4 by comparison to literature data.[2]

$^{31}P\{^1H\}$ NMR (162 MHz, C_6D_6): δ (ppm) −47.6 (s).

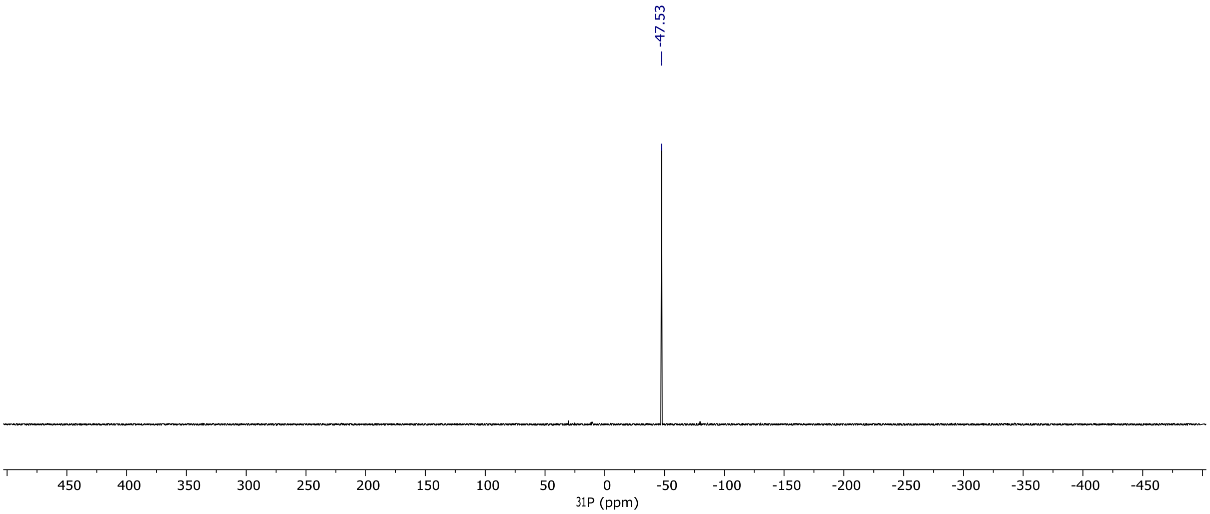

Figure S57. $^{31}P\{^1H\}$ NMR spectrum of **1b** in C_6D_6 immediately after the addition of IMe_4.

1.1.18. Metathesis of 1b with $Ge[CH(SiMe_3)_2]_2$ to give 1a

1b (10 mg, 0.014 mmol) and $Ge[CH(SiMe_3)_2]_2$ (10 mg, 0.026 mmol, in excess) were dissolved in C_6D_6 and allowed to stand at room temperature for 3 days. No reaction was observed by ^1H or $^{31}P\{^1H\}$ NMR spectroscopy. The mixture was heated at 80 °C for 18 h and monitored routinely by ^1H and $^{31}P\{^1H\}$ NMR spectroscopy during this time, which showed the gradual conversion of **1b** into **1a** in addition to small amounts of 3,3-dimethyl-5,7-di-*tert*-butylphosphaindane and **4b**, presumably through activation of adventitious water by **1b**.

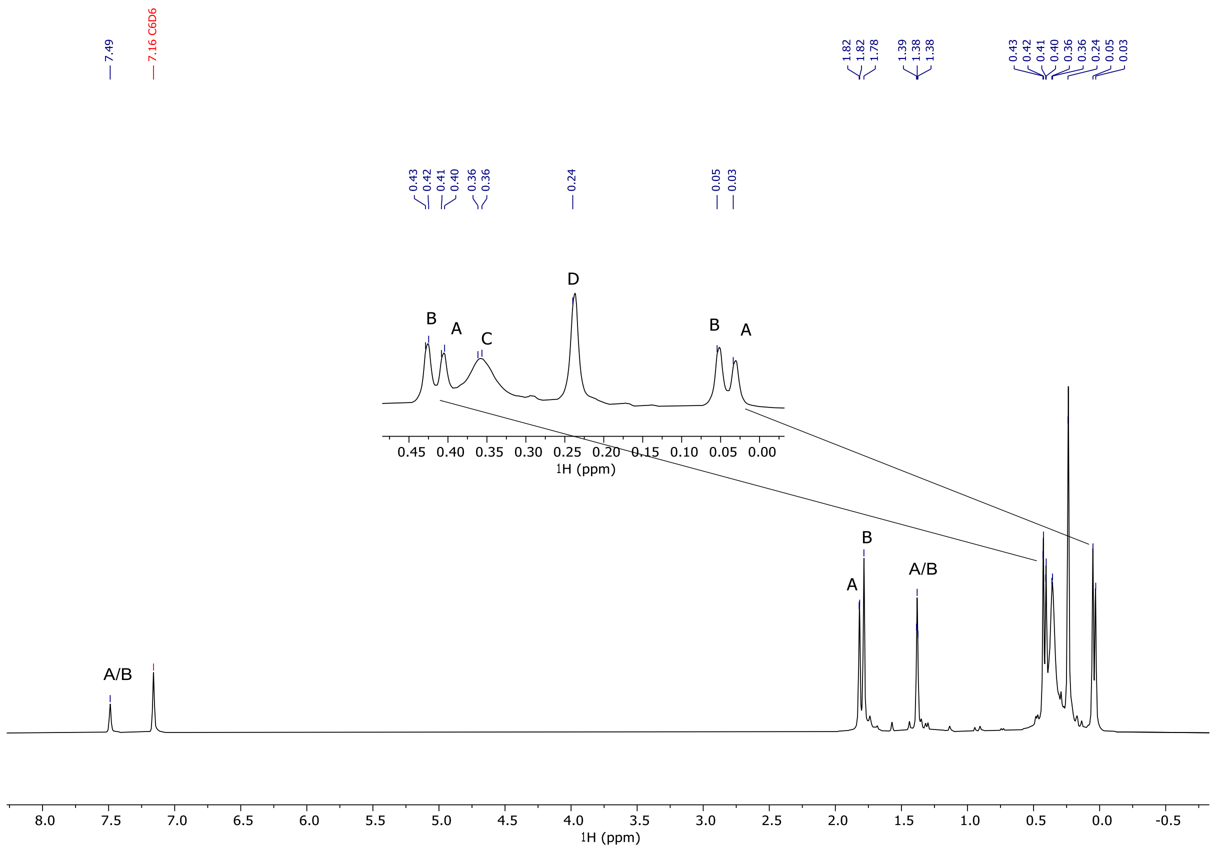

Figure S58. ^{1}H NMR spectrum in C_6D_6 of the reaction between **1b** and $Ge[CH(SiMe_3)_2]_2$ after 3 hours at 80 °C (A: **1b**; B: **1a**; C: $Ge[CH(SiMe_3)_2]_2$; D: $Sn[CH(SiMe_3)_2]_2$).

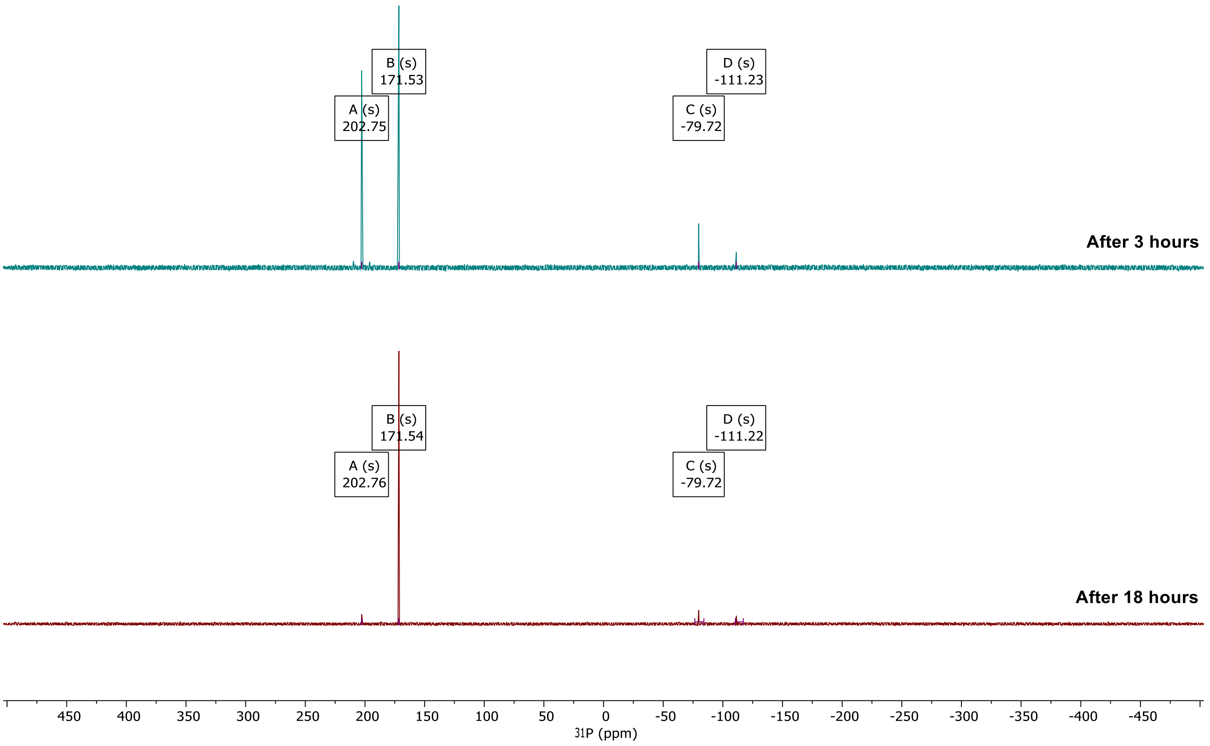

Figure S59. $^{31}P\{^{1}H\}$ NMR spectra of the reaction between **1b** and $Ge[CH(SiMe_3)_2]_2$ in C_6D_6 after heating for 3 hours (top) and 18 hours (bottom) at 80 °C (A: **1b**; B: **1a**; C: 3,3-dimethyl-5,7-di-*tert*-butylphosphaindane; D: **4b**).

1.2. Mechanistic investigations into the formation of 7b and 8

We undertook several control experiments in an attempt to identify the mechanism through which **8** is formed from the reaction with **1b** and imidazole, as we did not observe the corresponding aminophosphines when **1b** was reacted with any of the other amines investigated.

First, we investigated whether **8** was formed in a secondary reaction from **7b** with excess imidazole as the relative proportion of **8** generated was inconsistent between repeated experiments by ^{31}P NMR spectroscopy. The addition of 0.125 equivalents of imidazole (prepared as a DFB solution) to **1b** showed only remaining **1b** and **7b**, with no observation of **8**. Once 0.5 equivalents of imidazole had been added, another, smaller resonance at −80.4 ppm was observed, corresponding to 3,3-dimethyl-5,7-di-*tert*-butylphosphaindane by comparison to literature data. This species is the product of C–H activation of a *tert*-butyl group from the

transiently generated phosphinidine Mes*P, which we do not observe for **1b** at room temperature in the absence of imidazole.

We noted that leaving solutions of **1b** and imidazole for several months at room temperature led to the slow conversion of **7b** to **8**, which could be accelerated by heating at 80 °C for 4 hours. Additionally, even with 0.5 equivalents of imidazole we observed formation of $CH_2(SiMe_3)_2$, suggesting decomposition of the tin fragment. The reaction of $Sn[CH(SiMe_3)_2]_2$ with imidazole showed similar formation of $CH_2(SiMe_3)_2$ as part of an intractable mixture of products over several weeks at room temperature.

Next, we probed the presence of an equilibrium process to interconvert **7b** and **8**. The addition of $Sn[CH(SiMe_3)_2]_2$ to an independently prepared sample of **8** showed the formation of **7b** as the primary product, but decomposition to a mixture including $CH_2(SiMe_3)_2$ could be observed after several weeks. The corresponding reaction between Mes*P(H)(NH^{i}Pr) and $Sn[CH(SiMe_3)_2]_2$ did not occur at room temperature, implying that the mechanism forming **5b** does not proceed via an aminophosphine intermediate. Decomposition to Mes*PH_2 was observed upon heating.

Finally, we investigated the reaction of other activation products with imidazole. Addition of excess imidazole to **5b** led to the formation of Mes*P(H)(NH^{i}Pr) along with smaller amounts of **7b**. Over several months, decomposition to a complex mixture of products was observed, including Mes*PH_2 and $CH_2(SiMe_3)_2$. While we cannot discount a direct pathway between **7b** and **8**, DFT calculations suggest an intramolecular reaction is thermally inaccessible. Relaxed surface scans along the P–N and Sn–N coordinates suggest a barrier in excess of ~60 kcal mol^{-1}. The observation of 3,3-dimethyl-5,7-di-*tert*-butylphosphaindane suggests a phosphinidine intermediate allows the interconversion of **7b** and **8**. The decomposition of the tin fragment prevents an equilibrium from being established, leading to slow conversion to **8**.
Our tentative mechanistic proposal is summarised in Figure S60:

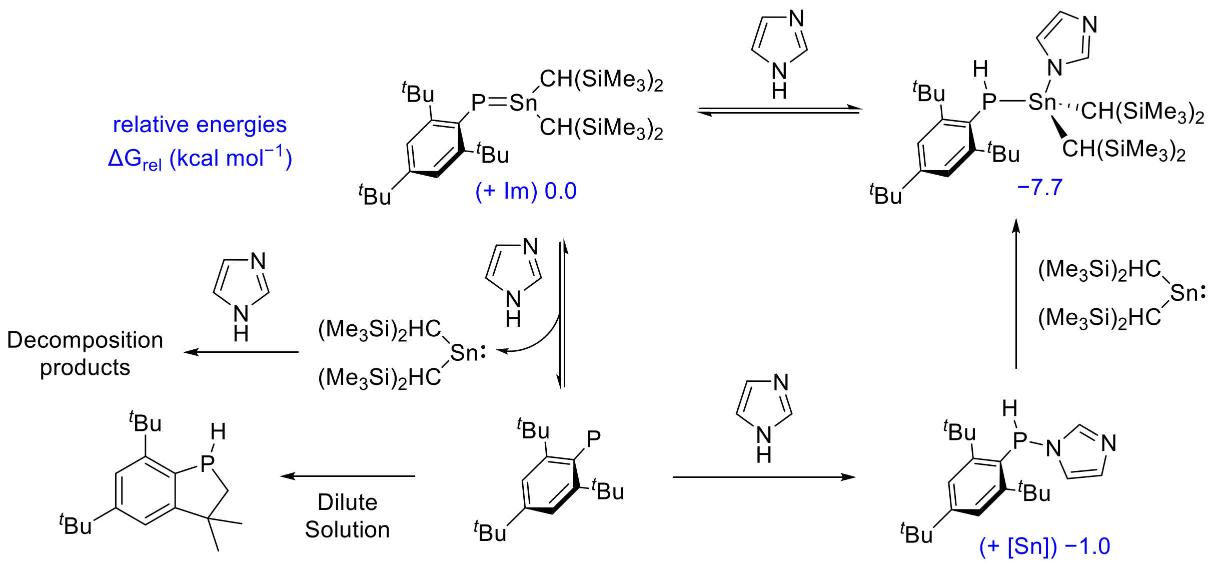

Figure S60. Proposed mechanistic pathway for the formation of **8** from **7b** along with their relative energies in kcal mol^{-1}.

1.2.1. The reaction of 1b with sub-stoichiometric amounts of imidazole in DFB

To a J. Young NMR tube charged with a C_6D_6 capillary was added **1b** (10 mg, 0.014 mmol) as a solution in DFB (0.4 mL). 0.05 mL of a freshly prepared imidazole solution in DFB (2.4 mg mL^{-1}, 0.002 mmol) was added by syringe. The solution became orange. $^{31}P\{^{1}H\}$ NMR spectroscopy showed the formation of a small amount of **7b** and no formation of **8**. A further 0.15 mL of the imidazole/DFB solution (0.006 mmol) was added by syringe and the solution became pale orange. $^{31}P\{^{1}H\}$ NMR spectroscopy showed further formation of **7b**; a small amount of 3,3-dimethyl-5,7-di-*tert*-butylphosphaindane was also observed. After several months, the decomposition products $CH_2(SiMe_3)_2$ and Mes*PH_2 could be observed.

$^{31}P\{^{1}H\}$ NMR (162 MHz, $C_6H_4F_2/C_6D_6$): δ (ppm) 198.8 (s; **1b**), −80.4 (s; **8**), −97.8 (s; **7b**), −111.4 (s; **4b**).

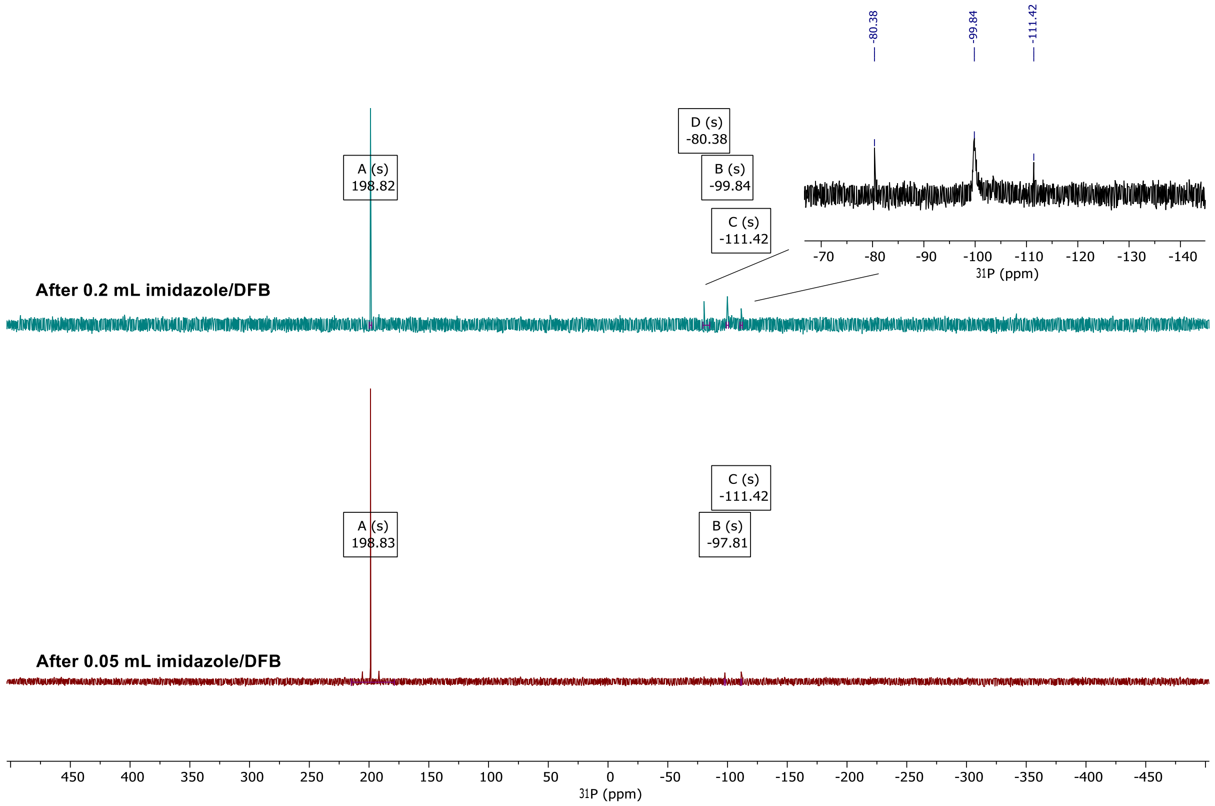

Figure S61. $^{31}P\{^{1}H\}$ NMR spectra (162 MHz, 293 K) of **1b** in DFB/C_6D_6 following the addition of sub-stoichiometric amounts of imidazole in DFB.

1.2.2. The reaction between $Sn[CH(SiMe_3)_2]_2$ and imidazole

$Sn[CH(SiMe_3)_2]_2$ (10 mg, 0.023 mmol) and imidazole (1.5 mg, 0.022 mmol) were dissolved in C_6D_6 in an NMR tube. After 10 minutes of vigorous shaking, the solution had decolourised. The $^{1}H\{^{119}Sn\}$ HMBC spectrum could not confirm the identity of the primary tin-containing product (21.5 ppm), which showed no correlation to imidazole protons (Figure S62). Decomposition to $CH_2(SiMe_3)_2$ as part of an intractable mixture of products was observed after several weeks at room temperature.

^{119}Sn (186 MHz, C_6D_6): δ (ppm) 21.5 (s), 157.2 (s).

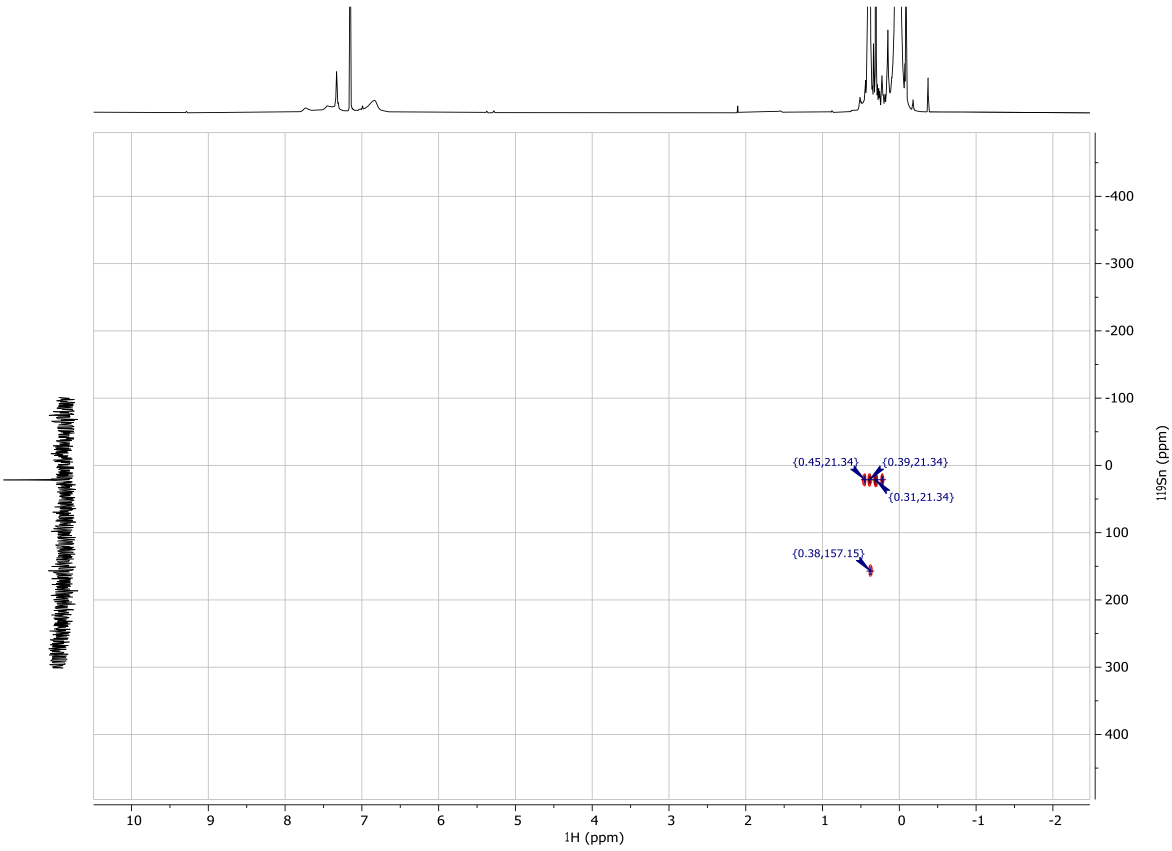

Figure S62. The $^{1}H\{^{119}Sn\}$ HMBC spectrum (186 MHz, 293 K) of the reaction between $Sn[CH(SiMe_3)_2]_2$ and imidazole in C_6D_6.

1.2.3. The reaction of 8 and $Sn[CH(SiMe_3)_2]_2$

8 (9.0 mg, 0.026 mmol) was dissolved in C_6D_6 (0.5 mL) in a J. Young NMR tube. $Sn[CH(SiMe_3)_2]_2$ (11.4 mg, 0.026 mmol) was added and the purple colour immediately returned to colourless. The ^{1}H and ^{31}P NMR spectra were then recorded without further purification, revealing the complete consumption of **8** and the formation of **7b** as the major product. The mixture decomposes slowly over several days, forming $CH_2(SiMe_3)_2$.

^{1}H NMR (400 MHz, C_6D_6): δ (ppm) 7.95 (s, 1H; imidazole C*H*), 7.50 (s, 1H; imidazole C*H*), 7.44 (d, *J* = 2.8 Hz, 2H; Mes* ArC*H*); 7.04 (s, 1H; imidazole C*H*), 5.35 (d, $^{1}J_{P\text{–}H}$ = 210 Hz; ArP**H**), 1.57 (s, 18H; Mes* *ortho*-C(CH_3)$_3$), 1.29 (s, 9H; Mes* *para*-C(CH_3)$_3$), 0.14 (d, *J* = 9.4 Hz, 18H; SiC**H**$_3$), 0.07 (d, *J* = 7.7 Hz, 18H; SiCH_3).

^{31}P NMR (162 MHz, C_6D_6): δ (ppm) −100.4 (d, $^{1}J_{P\text{–}H}$ = 210 Hz).

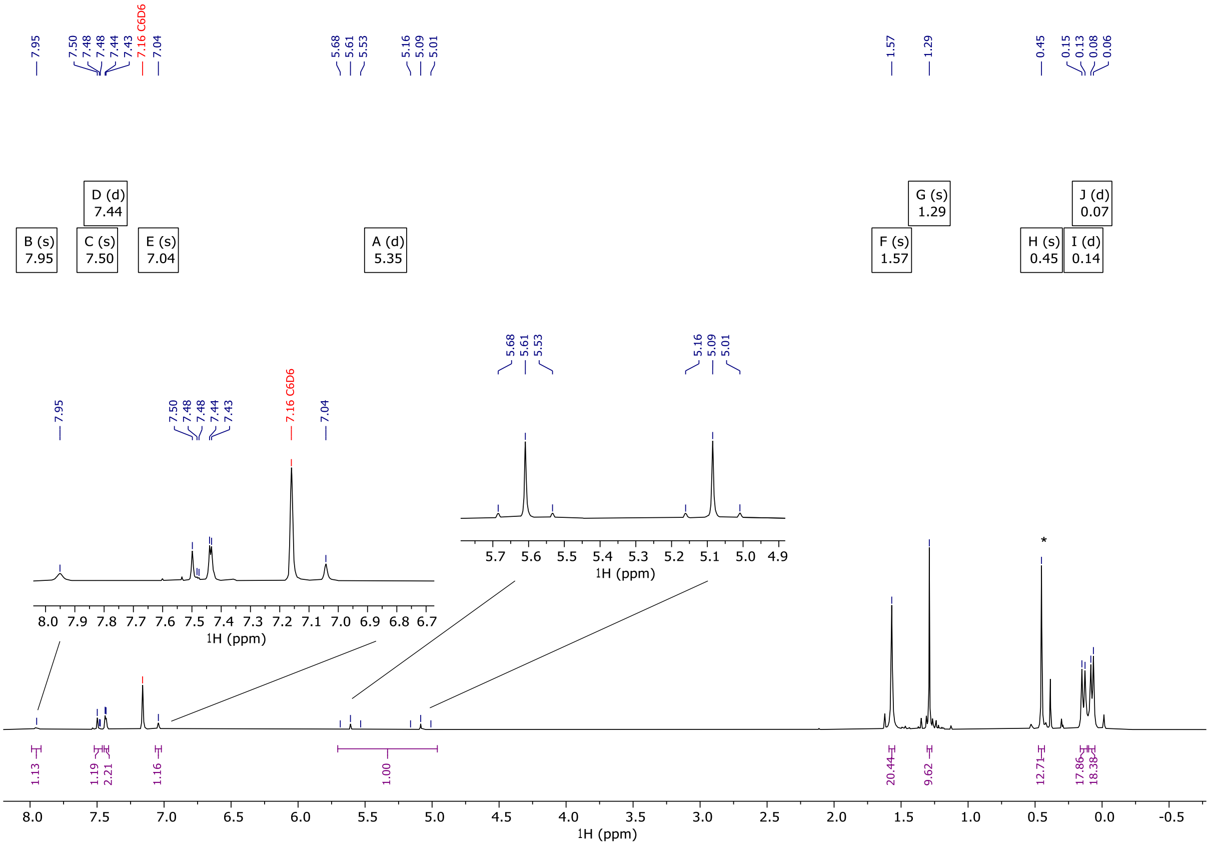

Figure S63. ^{1}H NMR spectrum (400 MHz, 293 K) of the solution of **8** in C_6D_6 immediately after addition of $Sn[CH(SiMe_3)_2]_2$ (* Unknown impurity).

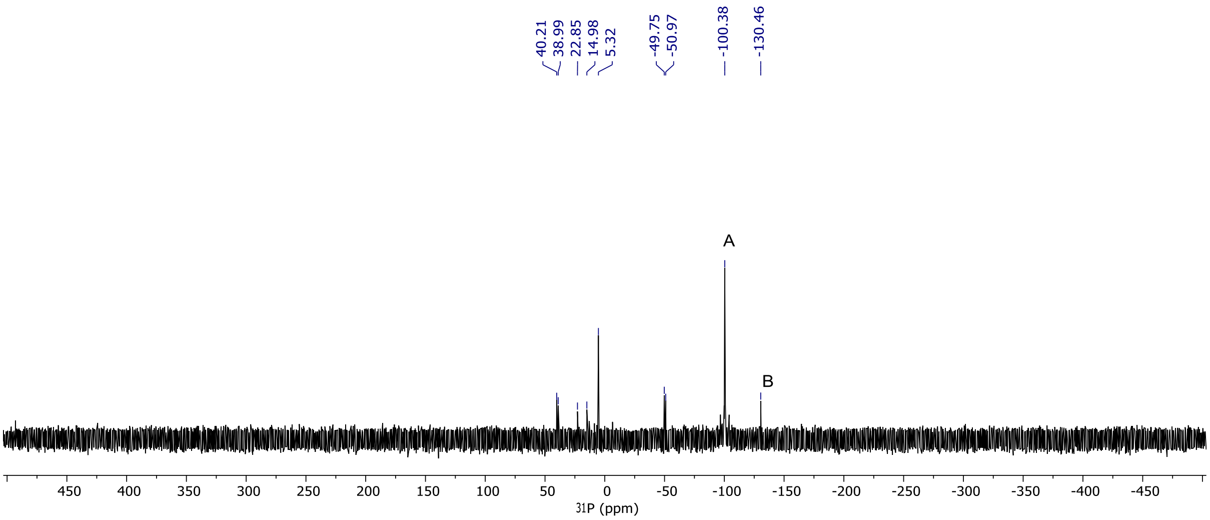

Figure S64. $^{31}P\{^{1}H\}$ NMR spectrum (162 MHz, 293 K) of the solution of **8** in C_6D_6 immediately after addition of $Sn[CH(SiMe_3)_2]_2$ (A: **7b**; B: Mes*PH_2).

1.2.4. The reaction of 8 and Ge[CH(SiMe$_3$)$_2$]$_2$

8 (8.3 mg, 0.024 mmol) was dissolved in C_6D_6 (0.5 mL) in a J-Young's NMR tube. Ge[CH(SiMe$_3$)$_2$]$_2$ (9.3 mg, 0.024 mmol) was added and the solution returned to colourless over several minutes of shaking. The ^{31}P{^{1}H} NMR spectrum was then recorded without further purification, revealing partial consumption of **8** and the formation of **7a** as part of a mixture of products, including Mes*PH$_2$. Excess Ge[CH(SiMe$_3$)$_2$]$_2$ (20 mg, 0.051 mmol) was then added and **7a** was observed as the major product, with CH$_2$(SiMe$_3$)$_2$ observed in the ^{1}H NMR spectrum.

^{31}P{^{1}H} NMR (162 MHz, C_6D_6): δ (ppm) −89.1 (s).

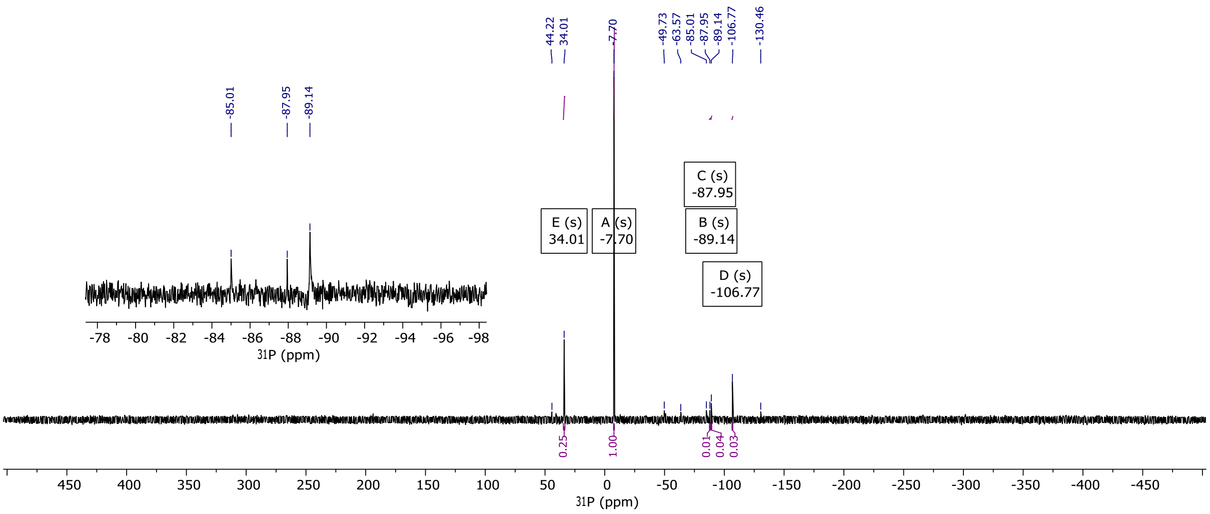

Figure S65. ^{31}P{^{1}H} NMR spectrum of the reaction between **8** and Ge[CH(SiMe$_3$)$_2$]$_2$ immediately after the solution decolourised (A: **8**; B: **7a** (see inset for expansion).

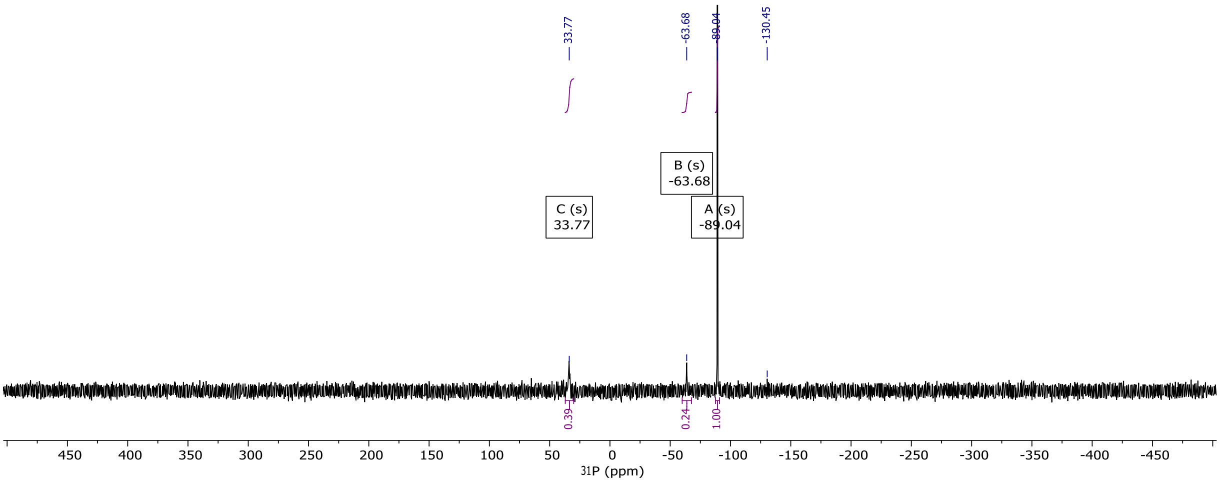

Figure S66. ^{31}P{^{1}H} NMR spectrum of the reaction between **8** and Ge[CH(SiMe$_3$)$_2$]$_2$, after excess germylene was added (A: **7a**).

1.2.5. The reaction of Mes*P(H)(NH^{i}Pr) and $Sn[CH(SiMe_3)_2]_2$

Mes*P(H)(NH^{i}Pr) was prepared *in situ* by reacting Me_3P–PMes* (10 mg, 0.028 mmol) with ^{i}PrNH$_2$ (0.01 mL, 0.116 mmol, in excess) in toluene (0.5 mL) and sonicating the mixture for 40 minutes. Following removal of the volatiles *in vacuo*, $Sn[CH(SiMe_3)_2]_2$ (12 mg, 0.027 mmol) was added and the solids dissolved in C_6D_6 (0.5 mL) to give a purple-red solution. No reaction was observed immediately or after leaving the mixture to stand at room temperature overnight by $^{31}P\{^1H\}$ NMR spectroscopy. Heating the mixture at 80 °C for 11 hours caused decomposition to Mes*PH_2; decomposition of $Sn[CH(SiMe_3)_2]_2$ to $CH_2(SiMe_3)_2$ was also observed.

^{1}H NMR (400 MHz, C_6D_6): δ (ppm) 7.51 (d, $^4J_{P–H}$ =1.8 Hz, 2H; Mes*ArC*H*), 6.50 (dd, $^1J_{P–H}$ = 225 Hz, $^2J_{P–H}$ = 7 Hz, 1H; P*H*), 2.78 (sept, $^3J_{H–H}$ = 6.6 Hz, 1H; NC***H***$(CH_3)_2$), 1.64 (s, 18H; Mes* *ortho*-C(C*H*$_3$)$_3$, 1.31 (s, 9H; Mes* *para*-C(C*H*$_3$)$_3$), 0.88 (m, 1H; N*H*), 0.75 (dd, $^2J_{H–H}$ = 40 Hz, $^4J_{H–H}$ = 6.5 Hz, 6H; NCH(C*H*$_3$)$_2$).

^{31}P NMR (162 MHz, C_6D_6): δ (ppm) −9.2 (dd, $^1J_{P–H}$ = 225 Hz, $^2J_{P–H}$ = 7 Hz; *P*H).

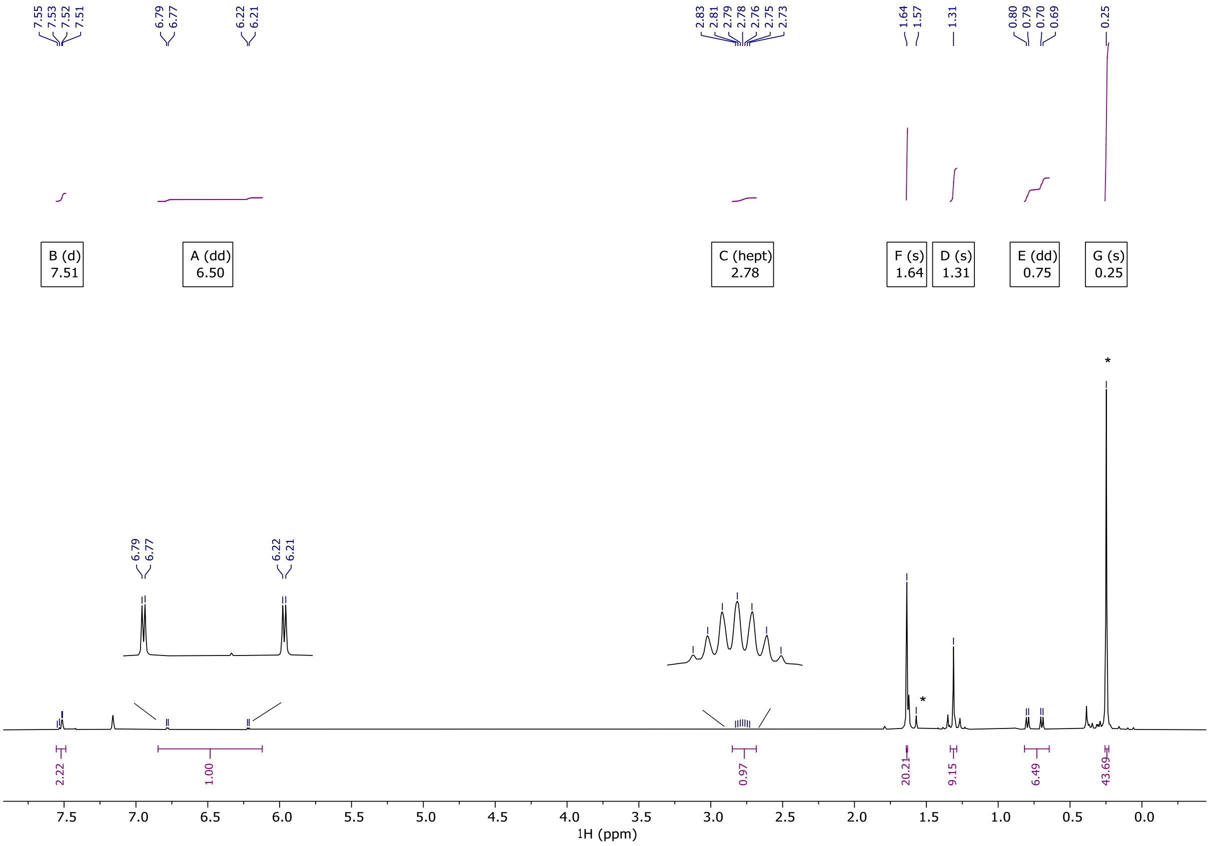

Figure S67. ^{1}H NMR spectrum (400 MHz, 293 K) in C_6D_6 of Mes*P(H)(NH^{i}Pr) immediately after the addition of $Sn[CH(SiMe_3)_2]_2$ (denoted by *).

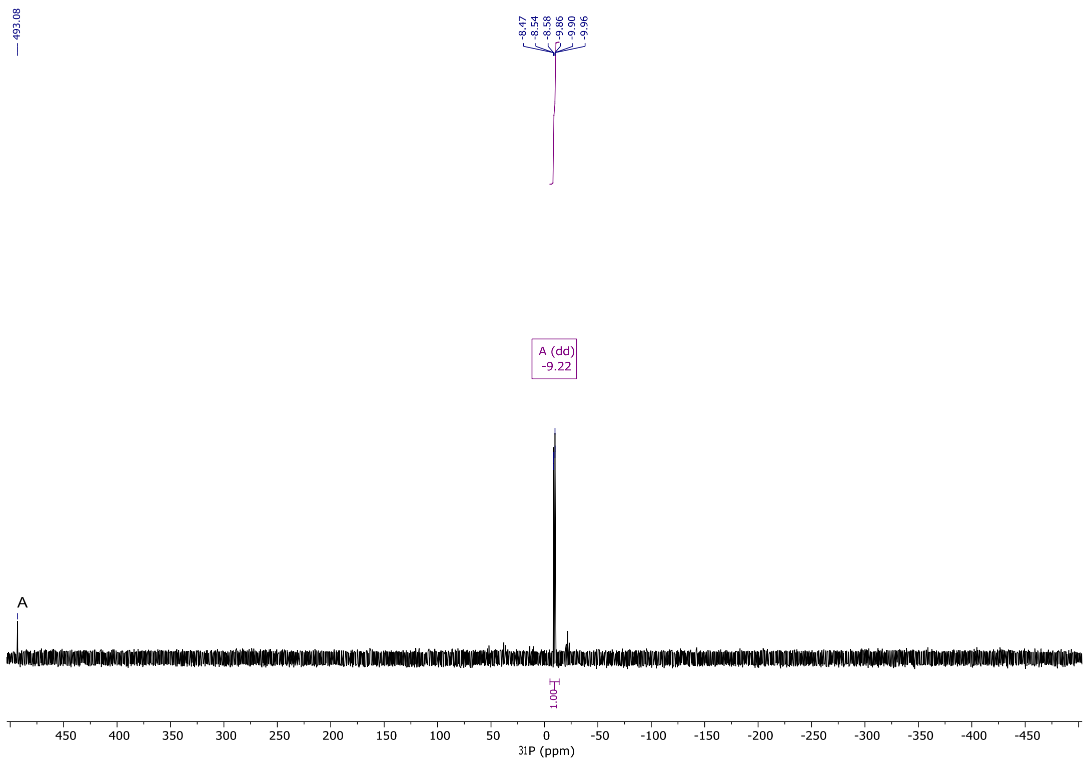

Figure S68. ^{31}P NMR spectrum (162 MHz, 293 K) in C_6D_6 of Mes*P(H)(NH^{i}Pr) immediately after the addition of Sn[CH($SiMe_3$)$_2$]$_2$ (A: Mes*P=PMes*).

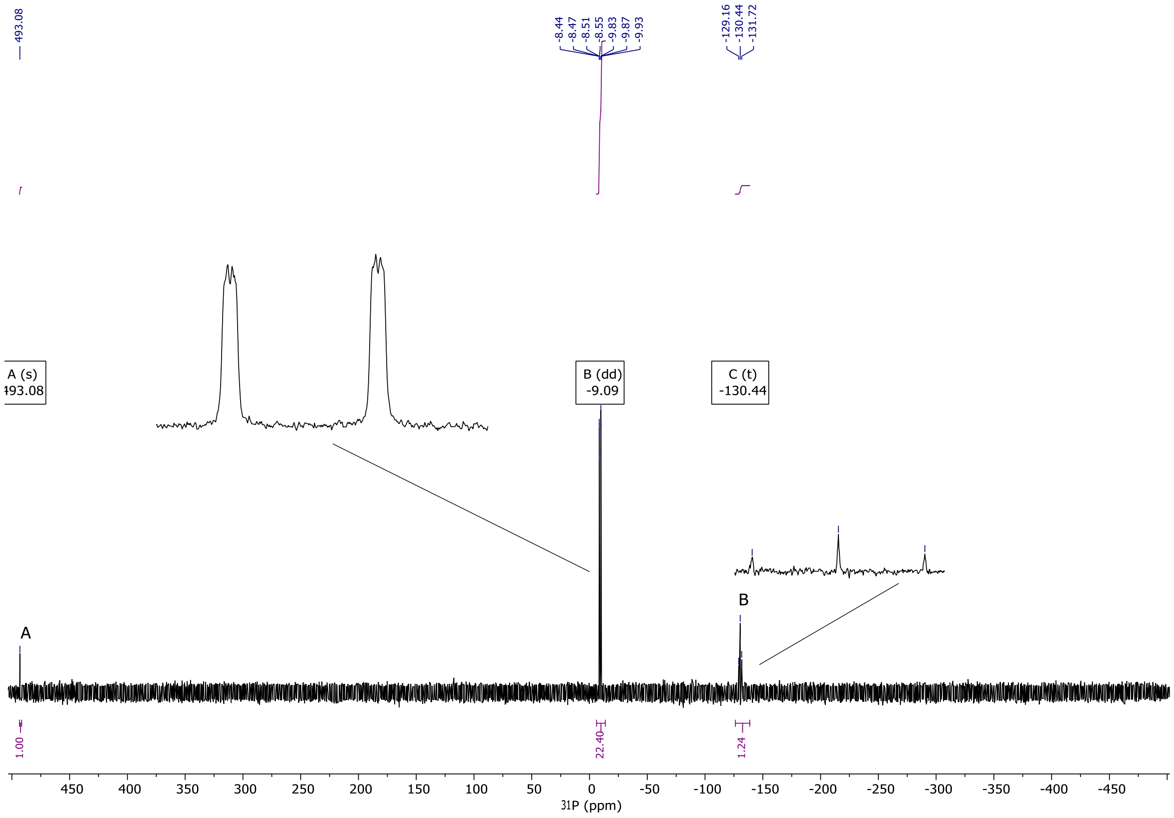

Figure S69. ^{31}P NMR spectrum (162 MHz, 293 K) of the reaction between Mes*P(H)(NHiPr) and Sn[CH($SiMe_3$)$_2$]$_2$ in C_6D_6 after heating to 80 °C for 11 hours (A: Mes*P=PMes*; B: Mes*PH_2).

1.2.6. The reaction between 5b and imidazole

5b was prepared *in situ* by heating a toluene solution of **1b** (10 mg, 0.014 mmol) with iPrNH$_2$ (0.01 mL, 0.116 mmol, in excess) to 80 °C for 30 minutes, followed by removal of the volatiles *in vacuo*. The remaining material was re-dissolved in C_6D_6 and imidazole (1.5 mg, 0.022 mmol) was added. The mixture was shaken overnight (repeatedly inverted by rotor) and the $^{31}P\{^1H\}$ NMR spectrum recorded without further purification. The formation of Mes*P(H)(NH^{i}Pr) was observed, along with **7b** and **4b** as part of a mixture of products. The solution was subsequently left to stand at room temperature and monitored routinely by ^1H and $^{31}P\{^1H\}$ NMR spectroscopy. After one week, partial decomposition to $CH_2(SiMe_3)_2$ was observed. Additional imidazole (10 mg, 0.147 mmol, in excess) was then added and the reaction monitored by NMR spectroscopy. The mixture decomposed to form Mes*PH$_2$; $CH_2(SiMe_3)_2$ is observed as the predominant product in the ^1H NMR spectrum.

$^{31}P\{^1H\}$ NMR (162 MHz, C_6D_6): δ (ppm) −9.2 (s).

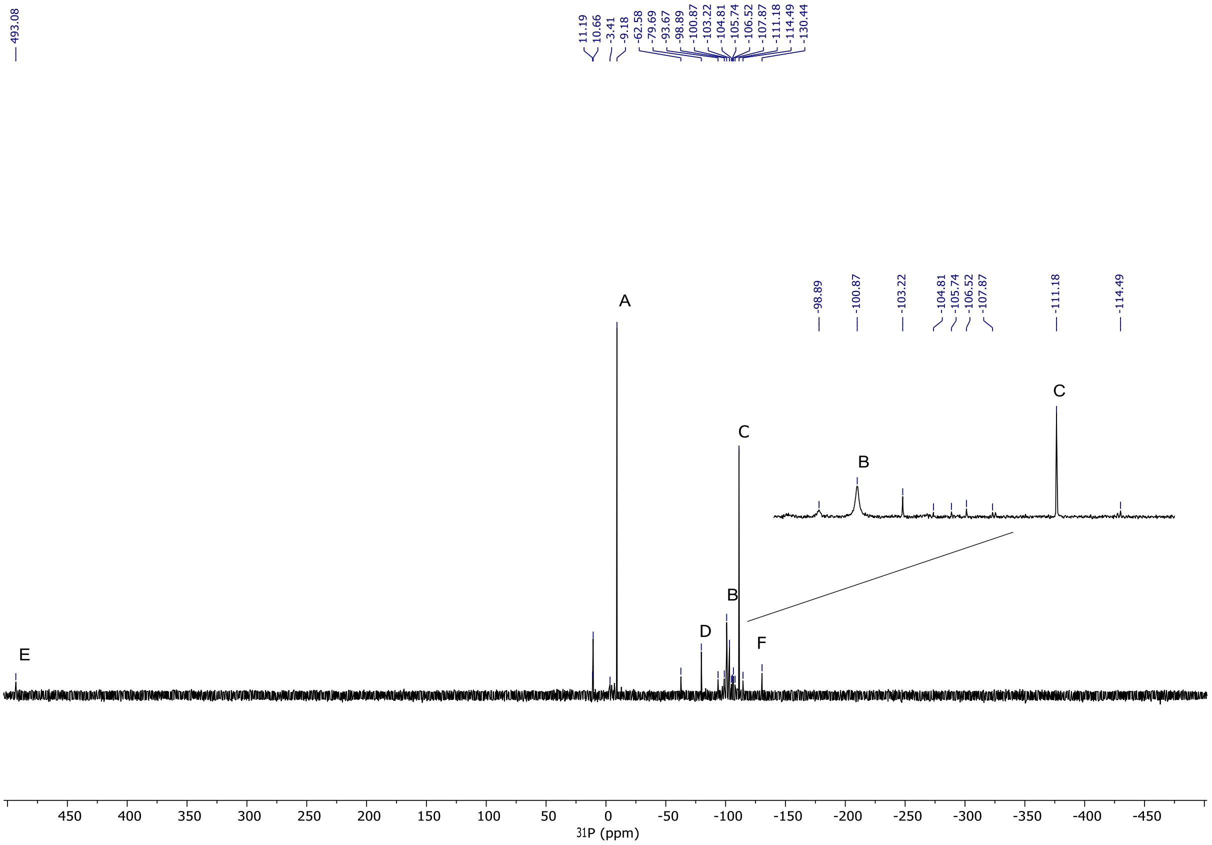

Figure S70. $^{31}P\{^1H\}$ NMR spectrum in C_6D_6 for the reaction of **5b** with imidazole, after being shaken overnight (A: Mes*P(H)(NH^{i}Pr); B: **7b**; C: **4b**; D: 3,3-dimethyl-5,7-di-*tert*-butylphosphaindane; E: Mes*P=PMes* F: Mes*PH$_2$).

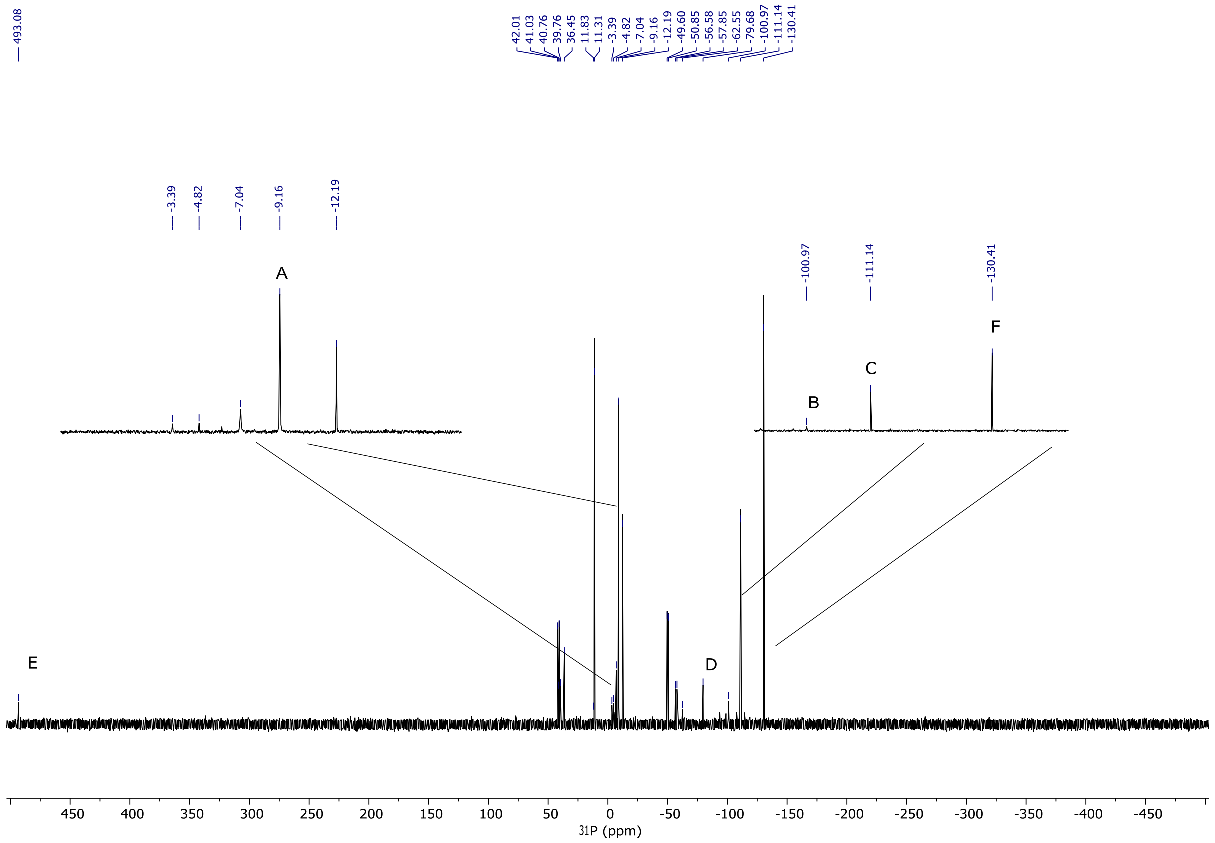

Figure S71. $^{31}P\{^1H\}$ NMR spectrum in C_6D_6 for the reaction of **5b** with excess imidazole, after being left to stand for several weeks at room temperature, showing significant decomposition (A: Mes*P(H)(NH^{i}Pr); B: **7b**; C: **4b**; D: 3,3-dimethyl-5,7-di-*tert*-butylphosphaindane; E: Mes*P=PMes* F: Mes*PH_2).

2. Single crystal X-ray diffraction data

Single-crystal X-ray diffraction data were collected using an Oxford Diffraction Supernova dual-source diffractometer equipped with a 135 mm Atlas CCD area detector. Crystals were selected under Paratone-N oil, mounted on micromount loops and quench-cooled using an Oxford Cryosystems open flow N_2 cooling device. Data were collected using mirror monochromated Cu Kα (λ = 1.54184 Å) radiation and processed using the CrysAlisPro package, including unit cell parameter refinement and inter-frame scaling (which was carried out using SCALE3 ABSPACK within CrysAlisPro).[41] Structures were subsequently solved using direct methods and refined on F^2 using the SHELXL package.[42]

Table S1. Selected X-ray data collection/refinement parameters for **1a**, **1b** and **2a**·C_6H_6.

	**1a**	**1b**	**2a**·C_6H_6
Formula	$C_{32}H_{67}GePSi_4$	$C_{32}H_{67}PSi_4Sn$	$C_{35}H_{73}GeNPSi_4$
CCDC	2263036	2263037	2263038
Fw [g mol^{-1}]	667.77	713.87	723.86
Crystal system	orthorhombic	orthorhombic	triclinic
Space group	P*ca*2$_1$	P*ca*2$_1$	*P*–1
a (Å)	22.9242(3)	23.1070(2)	12.2592(4)
b (Å)	9.7034(1)	9.7206(1)	12.2869(5)
c (Å)	18.0784(2)	18.2697(2)	15.6197(4)
α (°)	90	90	110.991(3)
β (°)	90	90	96.809(3)
γ (°)	90	90	95.915(3)
V (Å^3)	4021.41(8)	4103.63(7)	2154.09(13)
Z	4	4	2
Radiation, λ (Å)	Cu Kα, 1.54184	Cu Kα, 1.54184	Cu Kα, 1.54184
Temp (K)	150(2)	150(2)	150(2)
ρ_{calc} (g cm^{-3})	1.103	1.155	1.116
μ (mm^{-1})	2.673	6.558	2.536
Reflections collected	43174	44149	31351
Indep. reflections	7573	8524	8866
Parameters	364	364	411
R(int)	0.0463	0.0504	0.0452
R1/wR2,[a] I ≥ 2σI (%)	2.73/6.48	2.82/7.23	3.55/9.17
R1/wR2,[a] all data (%)	2.99/6.69	2.96/7.40	4.59/10.01
GOF	1.035	1.058	1.016

[a] $R1 = [\Sigma||F_o| - |F_c||]/\Sigma|F_o|$; $wR2 = \{[\Sigma w[(F_o)^2 - (F_c)^2]^2]/[\Sigma w(F_o^2)^2\}^{1/2}$; $w = [\sigma^2(F_o)^2 + (AP)^2 + BP]^{-1}$, where $P = [(F_o)^2 + 2(F_c)^2]/3$ and the A and B values are 0.0342 and 0.94 for **1a**, 0.0457 and 1.24 for **1b**, and 0.0530 and 0.32 for **2a**·0.5C_6H_6.

Table S2. Selected X-ray data collection/refinement parameters for **2b**, **3a** and **3b**.

	**2b**	**3a**	**3b**
Formula	$C_{32}H_{70}NPSi_4Sn$	$C_{35}H_{76}GeNPSi_4$	$C_{35}H_{76}NPSi_4Sn$
CCDC	2263039	2263040	2263041
Fw [g mol^{-1}]	730.91	726.88	772.98
Crystal system	triclinic	orthorhombic	orthorhombic
Space group	*P*–1	*Pbca*	*Pbca*
a (Å)	12.1685(4)	19.8880(3)	19.8623(1)
b (Å)	12.3376(4)	19.5445(2)	19.5191(1)
c (Å)	15.7132(3)	22.8970(3)	22.6808(1)
α (°)	97.079(2)	90	90
β (°)	109.517(2)	90	90
γ (°)	96.021(3)	90	90
V (Å^3)	2179.46(11)	8900.1(2)	8793.21(7)
Z	2	8	8
Radiation, λ (Å)	Cu Kα, 1.54184	Cu Kα, 1.54184	Cu Kα, 1.54184
Temp (K)	150(2)	150(2)	150(2)
ρ_{calc} (g cm^{-3})	1.114	1.085	1.168
μ (mm^{-1})	6.190	2.456	6.162
Reflections collected	23087	92431	75936
Indep. reflections	9018	9296	9148
Parameters	394	435	415
R(int)	0.0392	0.0510	0.0380
R1/wR2,[a] I ≥ 2σI (%)	3.14/7.95	4.32/11.30	2.33/5.94
R1/wR2,[a] all data (%)	3.69/8.40	5.17/12.37	2.80/6.35
GOF	1.051	1.020	1.060

[a] $R1 = [\Sigma||F_o| - |F_c||]/\Sigma|F_o|$; $wR2 = \{[\Sigma w[(F_o)^2 - (F_c)^2]^2]/[\Sigma w(F_o^2)^2\}^{1/2}$; $w = [\sigma^2(F_o)^2 + (AP)^2 + BP]^{-1}$, where $P = [(F_o)^2 + 2(F_c)^2]/3$ and the A and B values are 0.050 and 0.00 for **2b**, 0.0584 and 5.93 for **3a**, and 0.0299 and 3.49 for **3b**.

Table S3. Selected X-ray data collection/refinement parameters for **4a**·C_6H_6, **4b**·C_6H_6 and **5b**.

	**4a**·C_6H_6	**4b**·C_6H_6	**5b**
Formula	$C_{35}H_{76}GeOPSi_4$	$C_{35}H_{72}OPSi_4Sn$	$C_{35}H_{76}NPSi_4Sn$
CCDC	2263042	2263043	2263044
Fw [g mol^{-1}]	728.87	770.94	772.98
Crystal system	triclinic	triclinic	orthorhombic
Space group	*P*–1	*P*–1	*Pbca*
a (Å)	9.7147(3)	12.2815(2)	20.0280(1)
b (Å)	14.0348(4)	12.3157(3)	19.4129(1)
c (Å)	17.2944(6)	15.7550(3)	22.8813(2)
α (°)	111.136(3)	97.615(2)	90
β (°)	95.668(2)	110.289(2)	90
γ (°)	94.840(2)	96.077(2)	90
V (Å^3)	2170.24(13)	2185.72(8)	8896.29(10)
Z	2	2	8
Radiation, λ (Å)	Cu Kα, 1.54184	Cu Kα, 1.54184	Cu Kα, 1.54184
Temp (K)	150(2)	150(2)	150(2)
ρ_{calc} (g cm^{-3})	1.115	1.171	1.154
μ (mm^{-1})	2.530	6.209	6.091
Reflections collected	22306	25446	176660
Indep. reflections	8974	9024	9326
Parameters	408	421	430
R(int)	0.0314	0.0278	0.0490
R1/wR2,[a] I ≥ 2σI (%)	2.89/7.36	2.56/6.83	2.67/6.32
R1/wR2,[a] all data (%)	3.54/7.80	2.64/6.92	3.37/6.84
GOF	1.038	1.051	1.022

[a] $R1 = [\Sigma||F_o| - |F_c||]/\Sigma|F_o|$; $wR2 = \{[\Sigma w[(F_o)^2 - (F_c)^2]^2]/[\Sigma w(F_o{}^2)^2]\}^{1/2}$; $w = [\sigma^2(F_o)^2 + (AP)^2 + BP]^{-1}$, where $P = [(F_o)^2 + 2(F_c)^2]/3$ and the A and B values are 0.0400 and 0.56 for **4a**·C_6H_6, 0.0433 and 0.42 for **4b**·C_6H_6, and 0.0275 and 8.62 for **5b**.

Table S4. Selected X-ray data collection/refinement parameters for **6b**, **7a** and **8**.

	**6b**	**7a**	**8**
Formula	$C_{39}H_{76}NOPSi_4Sn$	$C_{35}H_{71}GeN_2PSi_4$	$C_{21}H_{33}N_2P$
CCDC	2263045	2263046	2263047
Fw [g mol^{-1}]	837.02	735.85	344.46
Crystal system	monoclinic	triclinic	orthorhombic
Space group	$P2_1/c$	P–1	$Ama2$
a (Å)	13.1874(1)	10.2505(2)	13.3603(10)
b (Å)	20.5131(2)	12.3185(2)	25.4799(12)
c (Å)	17.4922(2)	18.1941(4)	5.8949(4)
α (°)	90	98.415(2)	90
β (°)	92.684(1)	94.908(2)	90
γ (°)	90	110.422(2)	90
V (Å^3)	4726.70(8)	2106.55(8)	2006.7(2)
Z	4	2	4
Radiation, λ (Å)	Cu Kα, 1.54184	Cu Kα, 1.54184	Cu Kα, 1.54184
Temp (K)	150(2)	150(2)	150(2)
ρ_{calc} (g cm^{-3})	1.176	1.160	1.140
μ (mm^{-1})	5.788	2.610	1.223
Reflections collected	42435	50129	9792
Indep. reflections	9817	8757	1826
Parameters	453	413	141
R(int)	0.0250	0.0230	0.0732
R1/wR2,[a] I ≥ 2σI (%)	3.23/8.60	2.53/6.55	5.91/15.49
R1/wR2,[a] all data (%)	3.40/8.79	6.58/2.58	6.47/15.95
GOF	1.063	1.038	1.110

[a] $R1 = [\Sigma||F_o| - |F_c||]/\Sigma|F_o|$; $wR2 = \{[\Sigma w[(F_o)^2 - (F_c)^2]^2]/[\Sigma w(F_o^2)^2]\}^{1/2}$; $w = [\sigma^2(F_o)^2 + (AP)^2 + BP]^{-1}$, where $P = [(F_o)^2 + 2(F_c)^2]/3$ and the A and B values are 0.0598 and 0.79 for **6b**, 0.0324 and 1.26 for **7a**, and 0.1111 and 0.00 for **8**.

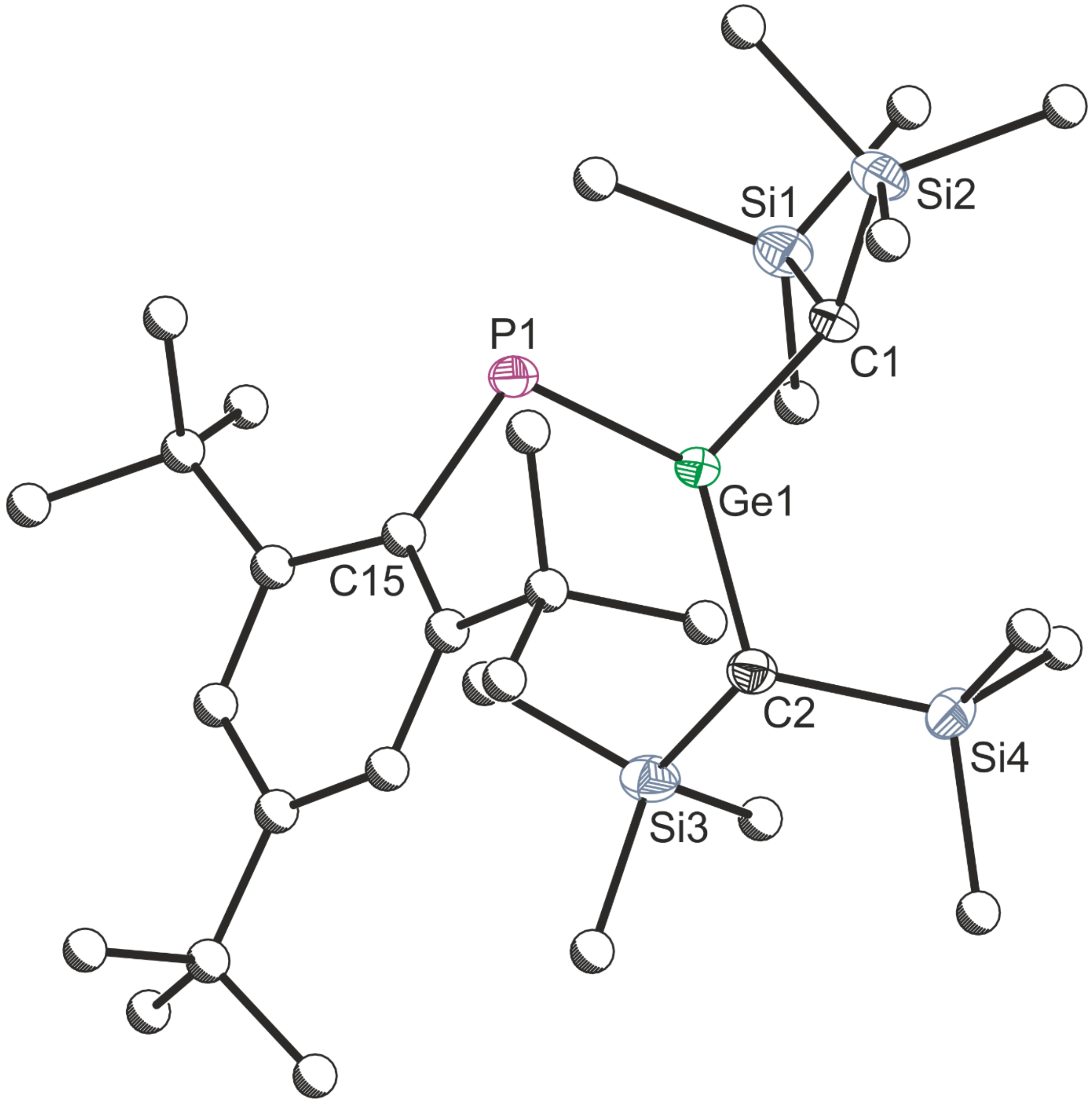

Figure S72. Single crystal X-ray structure of **1a**. Anisotropic displacement ellipsoids depicted at 50% probability. All hydrogen atoms removed for clarity. Carbon atoms of Mes* and Me substituents pictured as spheres of arbitrary radius. Selected bond lengths (Å) and angles (°): P1–Ge1 2.157(1), P1–C15 1.861(3), Ge1–C1 1.974(3), Ge1–C2 1.966(3); C15-P1-Ge1 104.84(9), P1-Ge1-C1 113.41(9), P1-Ge1-C2 128.37(8), C1-Ge1-C2 117.93(12).

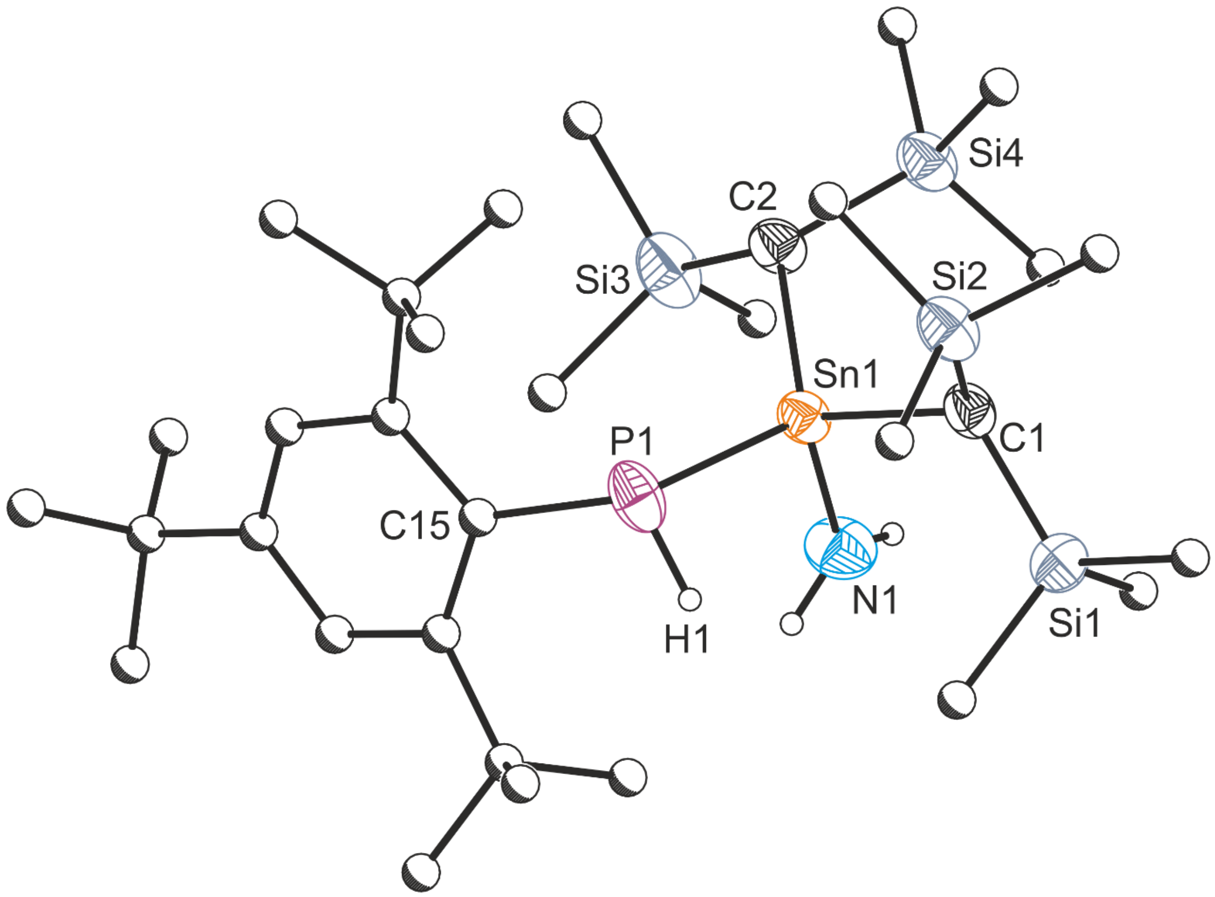

Figure S73. Single crystal X-ray structure of **2b**. Anisotropic displacement ellipsoids depicted at 50% probability. All hydrogen atoms (except H1 and on those on the NH_2 moiety) removed for clarity. Carbon atoms of Mes* and Me substituents pictured as spheres of arbitrary radius. Selected bond lengths (Å) and angles (°): P1–Sn1 2.567(1), P1–C15 1.862(2), P1–H1 1.26(4), Sn1–N1 2.074(2), Sn1–C1 2.189(2), Sn1–C2 2.173(2); C15-P1-Sn1 114.55(8), C15-P1-H1 106.0(18), P1-Sn1-N1 111.48(8), P1-Sn1-C1 101.22(6), P1-Sn1-C2 121.68(7).

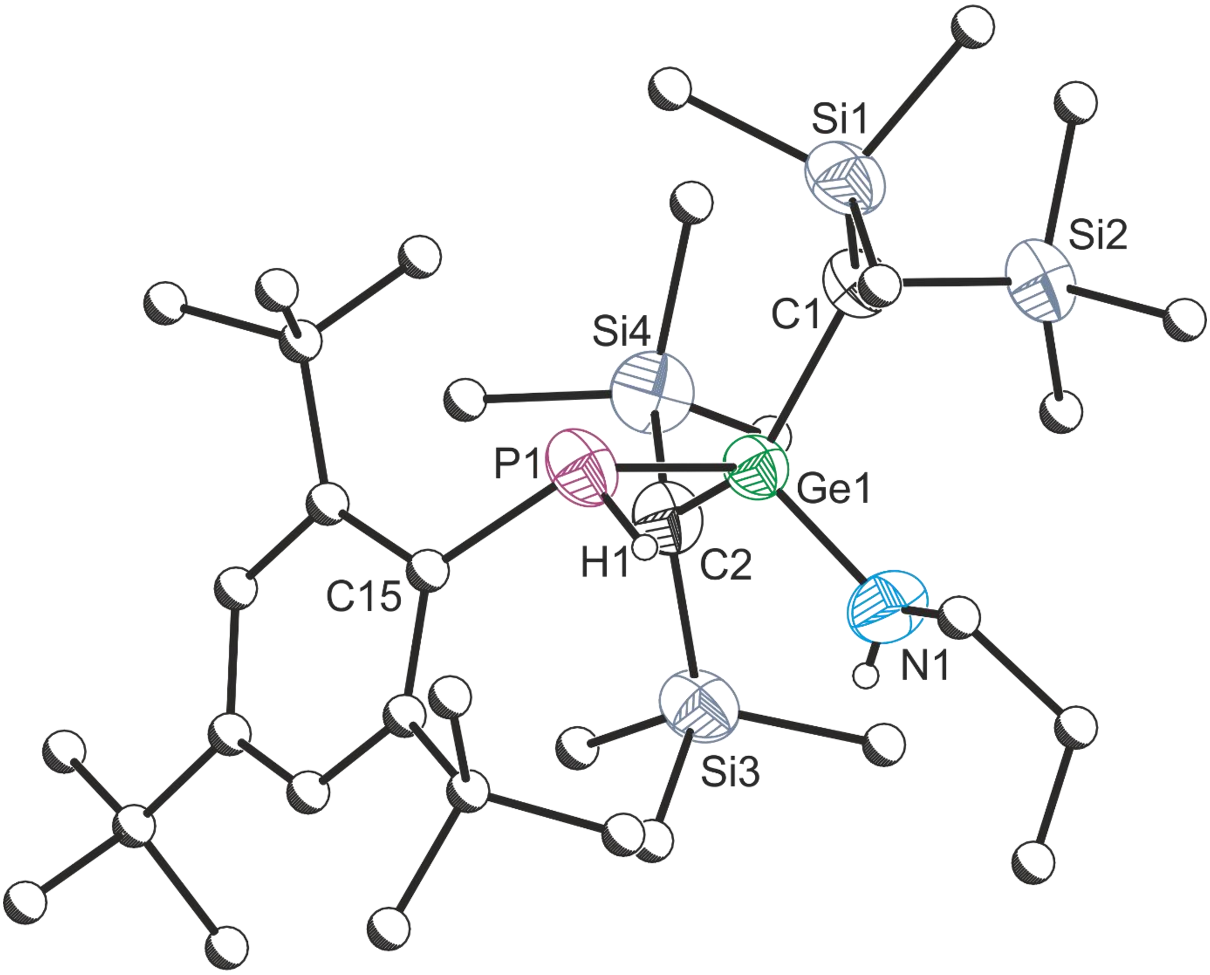

Figure S74. Single crystal X-ray structure of **3a**. Anisotropic displacement ellipsoids depicted at 50% probability. All hydrogen atoms (except H1 and amide proton) removed for clarity. Carbon atoms of Mes*, ^{n}Pr and Me substituents pictured as spheres of arbitrary radius. Selected bond lengths (Å) and angles (°): P1–Ge1 2.410(1), P1–C15 1.848(2), P1–H1 1.10(3), Ge1–N1 1.855(2), Ge1–C1 1.999(2), Ge1–C2 2.002(2); C15-P1-Ge1 108.13(7), C15-P1-H1 102.7(17), P1-Ge1-N1 108.44(8), P1-Ge1-C1 105.69(7), P1-Ge1-C2 110.77(7).

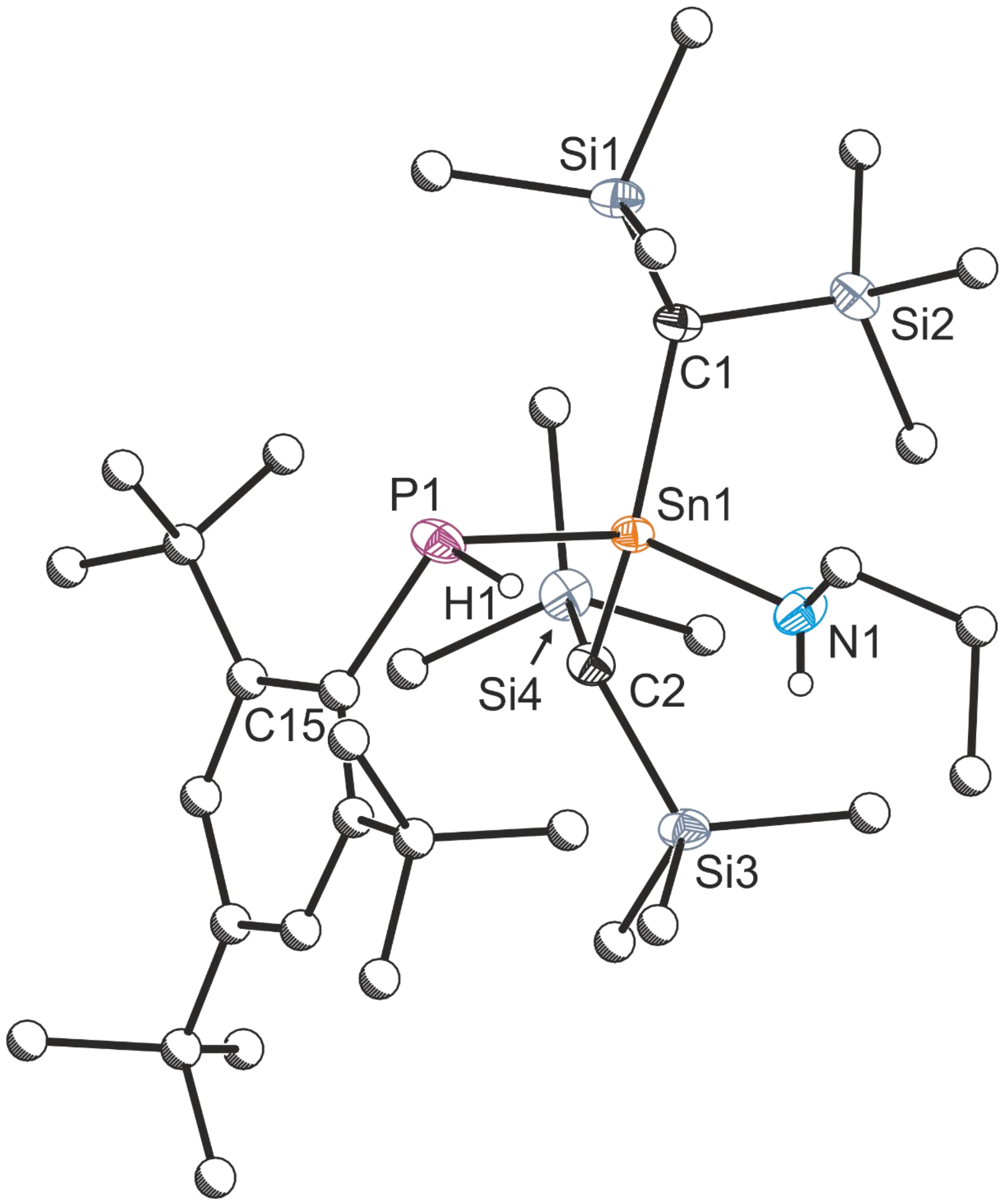

Figure S75. Single crystal X-ray structure of **3b**. Anisotropic displacement ellipsoids depicted at 50% probability. All hydrogen atoms (except H1 and amide proton) removed for clarity. Carbon atoms of Mes*, ^{n}Pr and Me substituents pictured as spheres of arbitrary radius. Selected bond lengths (Å) and angles (°): P1–Sn1 2.581(1), P1–C15 1.855(2), P1–H1 1.19(3), Sn1–N1 2.057(2), Sn1–C1 2.187(2), Sn1–C2 2.187(2); C15-P1-Sn1 104.58(4), C15-P1-H1 101.7(13), P1-Sn1-N1 108.04(4), P1-Sn1-C1 106.81(4), P1- Sn1-C2 111.08(4).

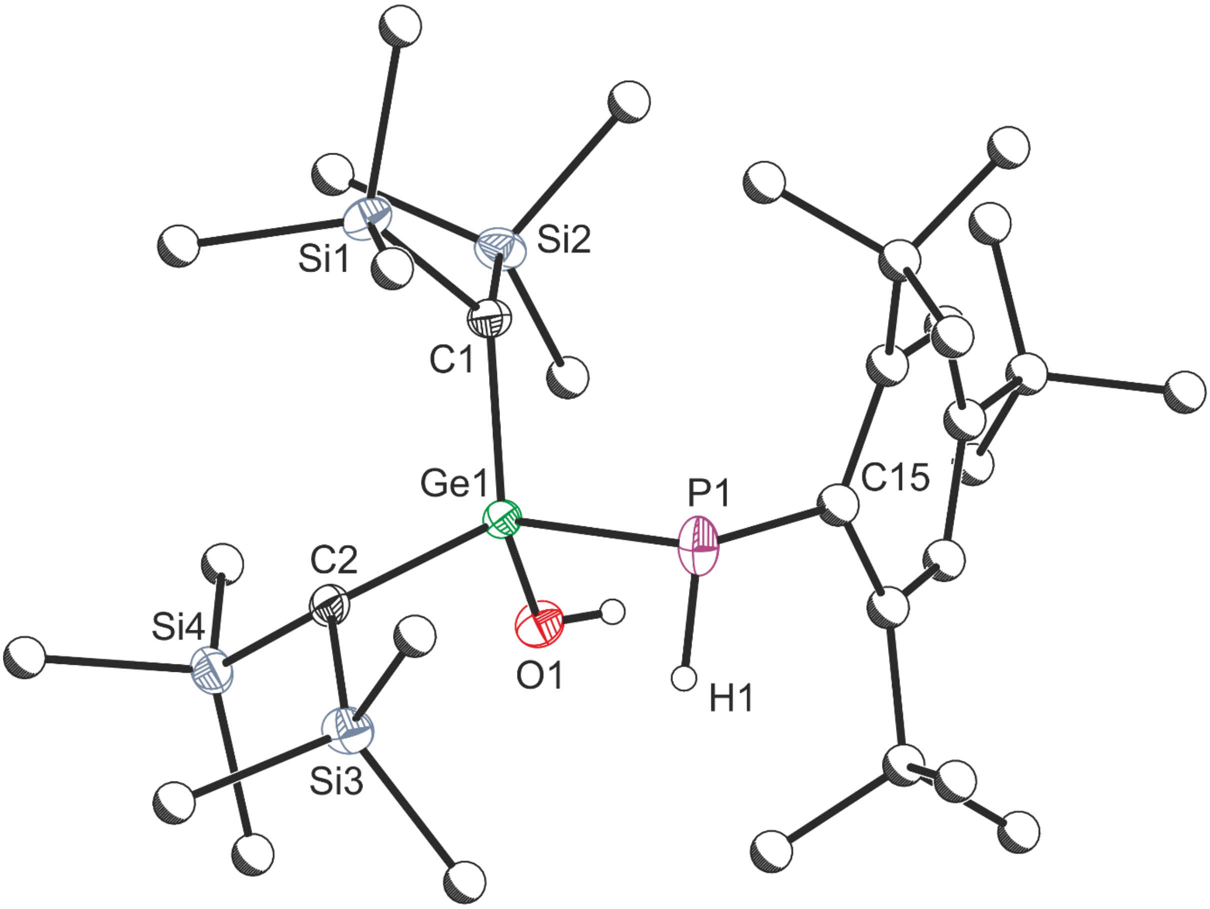

Figure S76. Single crystal X-ray structure of **4a**. Anisotropic displacement ellipsoids depicted at 50% probability. All hydrogen atoms (except H1 and hydroxide proton) removed for clarity. Carbon atoms of Mes* and Me substituents pictured as spheres of arbitrary radius. Selected bond lengths (Å) and angles (°): P1–Ge1 2.379(1), P1–C15 1.850(2), P1–H1 1.28(4), Ge1–O1 1.790(2), Ge1–C1 1.978(2), Ge1–C2 1.974(2); C15-P1-Ge1 98.94(5), C15-P1-H1 105.6(16), P1-Ge1-O1 105.23(4), P1-Ge1-C1 106.78(5), P1-Ge1-C2 114.30(5).

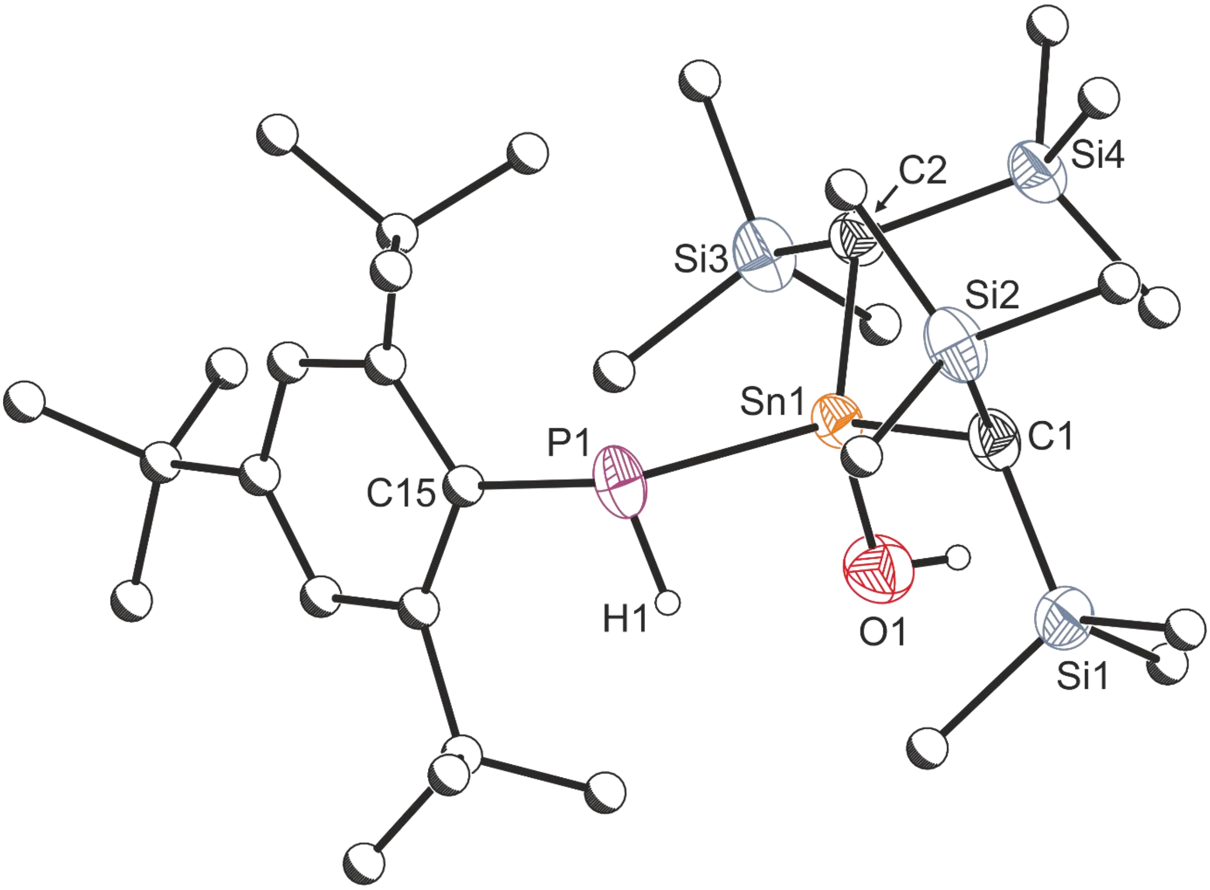

Figure S77. Single crystal X-ray structure of **4b**. Anisotropic displacement ellipsoids depicted at 50% probability. All hydrogen atoms (except H1 and hydroxide proton) removed for clarity. Carbon atoms of Mes* and Me substituents pictured as spheres of arbitrary radius. Selected bond lengths (Å) and angles (°): P1–Sn1 2.556(1), P1–C15 1.854(2), P1–H1 1.27(4), Sn1–O1 2.027(2), Sn1–C1 2.180(2), Sn1–C2 2.162(2); C15-P1-Sn1 113.96(5), C15-P1-H1 108.6(17), P1-Sn1-O1 109.76(5), P1-Sn1-C1 102.16(5), P1-Sn1-C2 123.35(5).

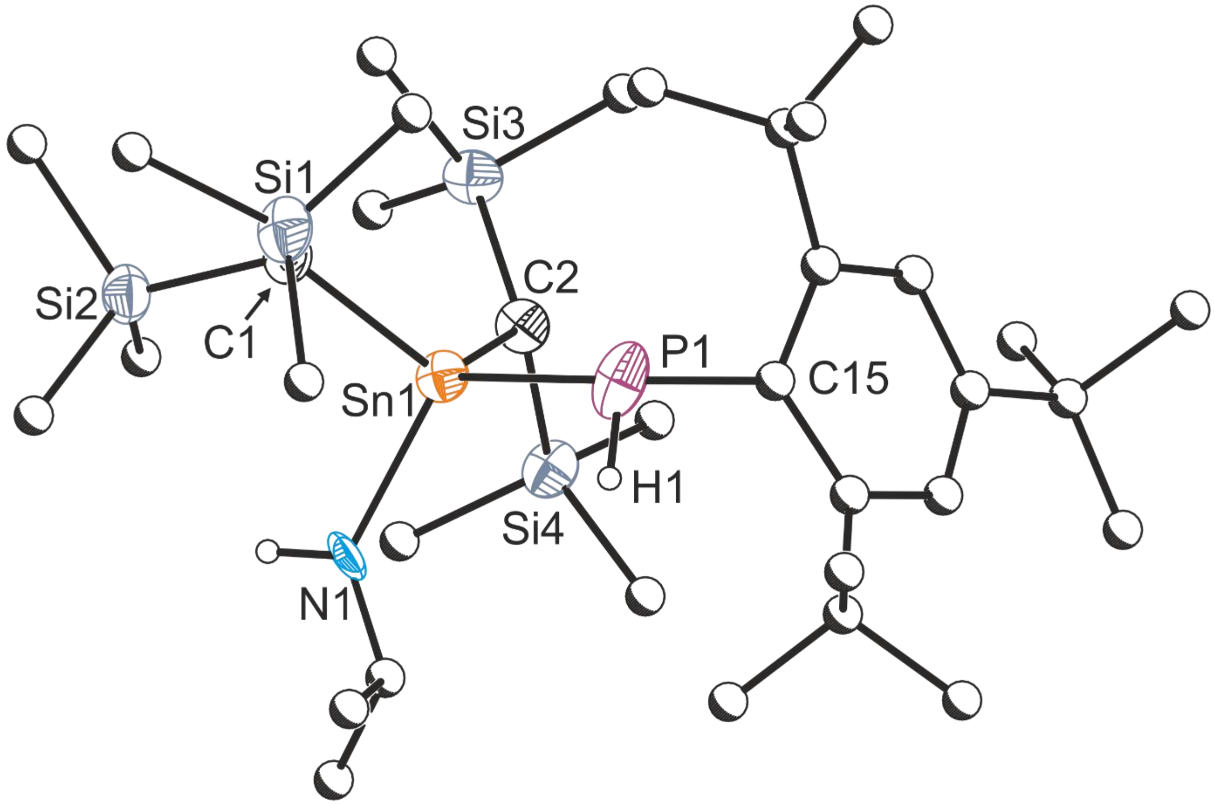

Figure S78. Single crystal X-ray structure of **5b**. Anisotropic displacement ellipsoids depicted at 50% probability. All hydrogen atoms (except H1 and amide proton) removed for clarity. Carbon atoms of Mes*, ^{i}Pr and Me substituents pictured as spheres of arbitrary radius. Selected bond lengths (Å) and angles (°): P1–Sn1 2.583(1), P1–C15 1.849(2), P1–H1 1.053(19), Sn1–N1 2.121(5), Sn1–C1 2.190(2), Sn1–C2 2.183(2); C15-P1-Sn1 106.54(6), C15-P1-H1 102(2), P1-Sn1-N1 110.92(17), P1-Sn1-C1 106.58(6), P1-Sn1-C2 110.52(6).

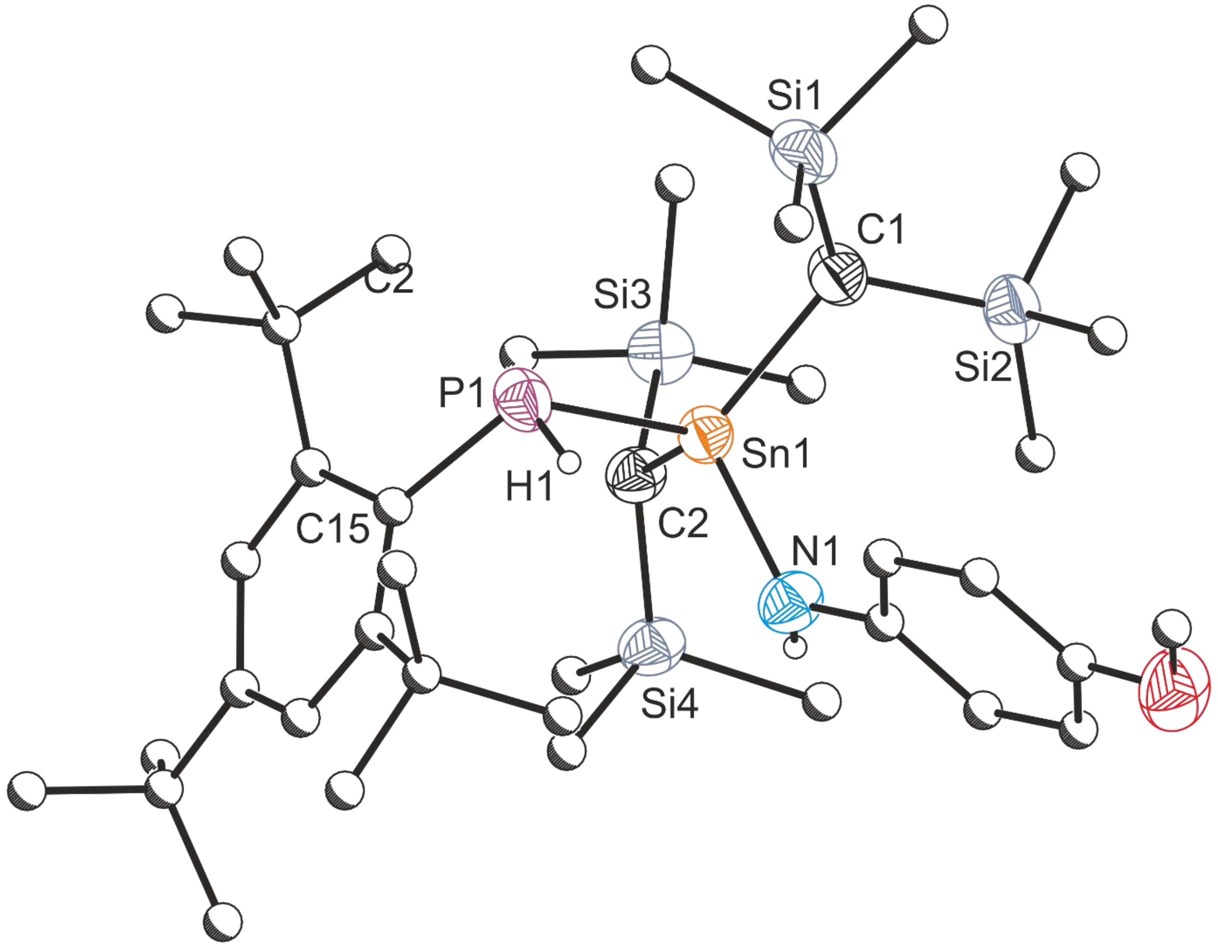

Figure S79. Single crystal X-ray structure of **6b**. Anisotropic displacement ellipsoids depicted at 50% probability. All hydrogen atoms (except H1 and amide proton) removed for clarity. Carbon atoms of Mes*, C_4H_5OMe and Me substituents pictured as spheres of arbitrary radius. Selected bond lengths (Å) and angles (°): P1–Sn1 2.564(1), P1–C15 1.849(2), P1–H1 1.09(3), Sn1–N1 2.059(2), Sn1–C1 2.185(2), Sn1–C2 2.187(2); C15-P1-Sn1 105.34(6), C15-P1-H1 91.0(16), P1-Sn1-N1 104.84(5), P1-Sn1-C1 108.11(5), P1-Sn1-C2 112.25(5).

3. Computational details

3.1. General computational methods

Density functional theory (DFT) calculations were performed using the ORCA 5.0.2 software package.[43–45] All methods were used as implemented. Geometries were optimized using the B97-D3 functional, corrected for relativistic effects using the zeroth order regular approximation (ZORA), the Resolution of Identity approximation (RIJCOSX), and using the segmented all-electron relativistically contracted split-valence basis set SARC-ZORA-SVP for tin and the relativistically contracted split-valence basis set ZORA-def2-SVP for all other atoms, along with the SARC/J auxiliary basis set.[46–49] Analytical frequency calculations were carried out to verify all geometries were true minima or saddle points. Single point calculations on all compounds and fragments were performed using the ωB97X-D3 functional and the Resolution of Identity approximation (RIJCOSX).[50] The segmented all-electron relativistically contracted basis set SARC-ZORA-TZVPP was used for tin, and the relativistically contracted triple-zeta basis set ZORA-def2-TZVPP was used for all other atoms, along with the SARC/J auxiliary basis set.[51,52] Transition state geometries were optimized using an eigenvector following algorithm.[53] Minimum energy crossing points (MECPs) between the singlet and triplet potential energy surfaces were located and optimized following the principles suggested by Harvey et al.[54] Natural Bond Order analysis and Natural Population Analysis were carried out using the NBO 7.0 program.[55]

3.2. Electronic structure calculations

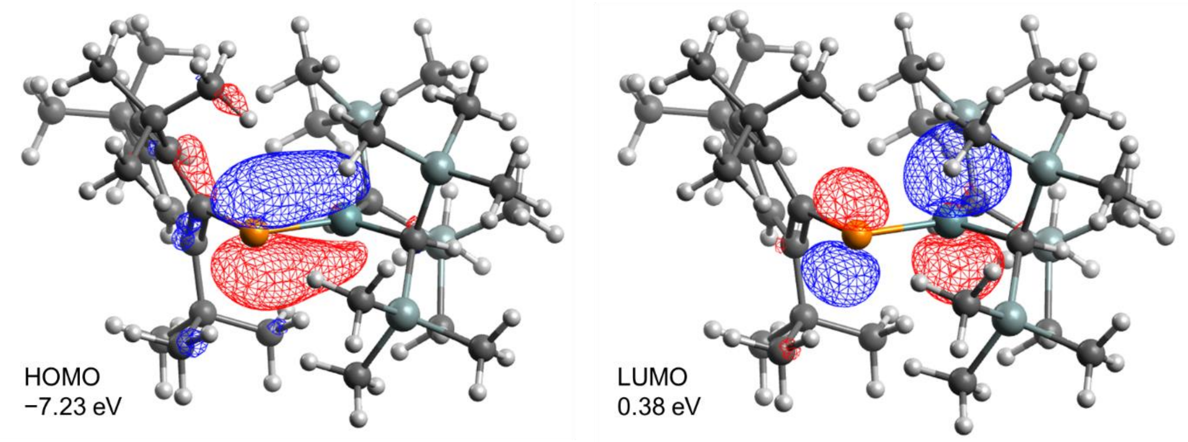

Figure S80. Frontier Kohn-Sham molecular orbitals of **1a**.

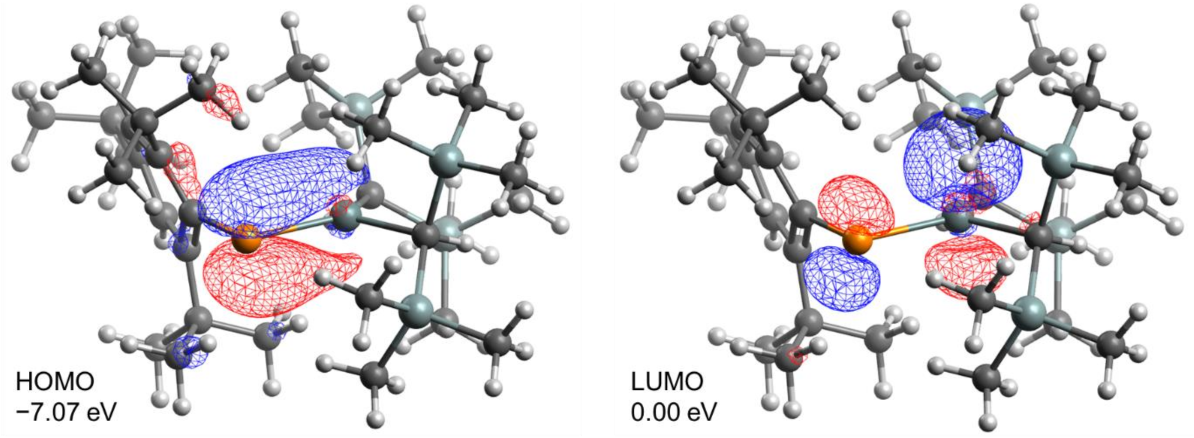

Figure S81. Frontier Kohn-Sham molecular orbitals of **1b**.

Table S5. Summary of NBO and NPA analysis of **1a** and **1b**.

	NPA Charge		NBO π(P=E)		Wiberg Bond Index
**1a**	P	Ge	%P	%Ge	P–Ge
	–0.16	+1.24	69.81	30.19	1.66
**1b**	P	Sn	%P	%Sn	P–Sn
	–0.34	+1.48	74.64	25.36	1.48

3.3. Computed mechanisms

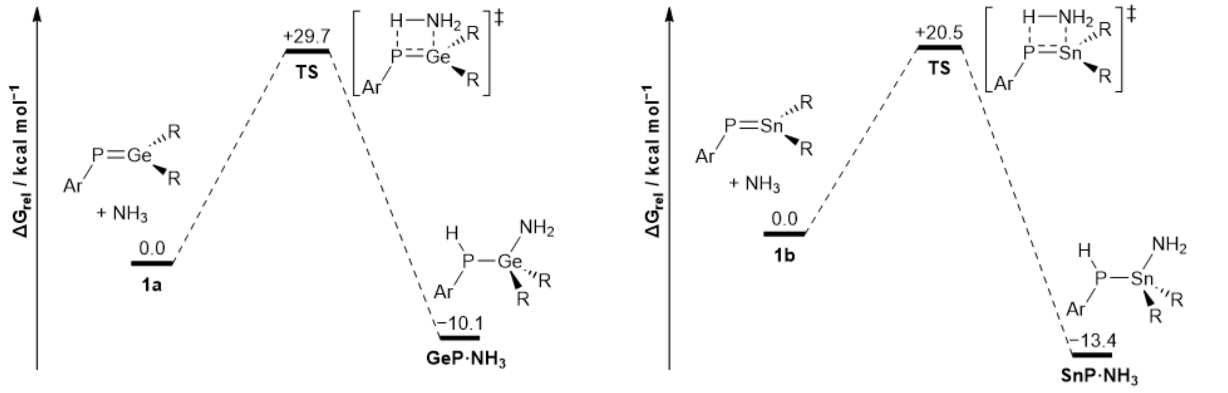

Figure S82. Computed mechanisms for the activation of ammonia by **1a** and **1b**.

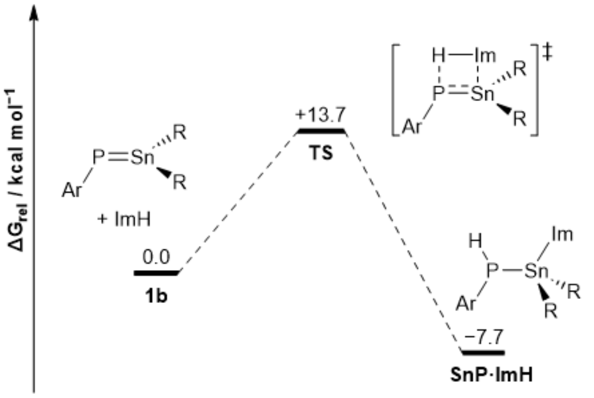

Figure S83. Computed mechanism for the activation of imidazole by **1b**.

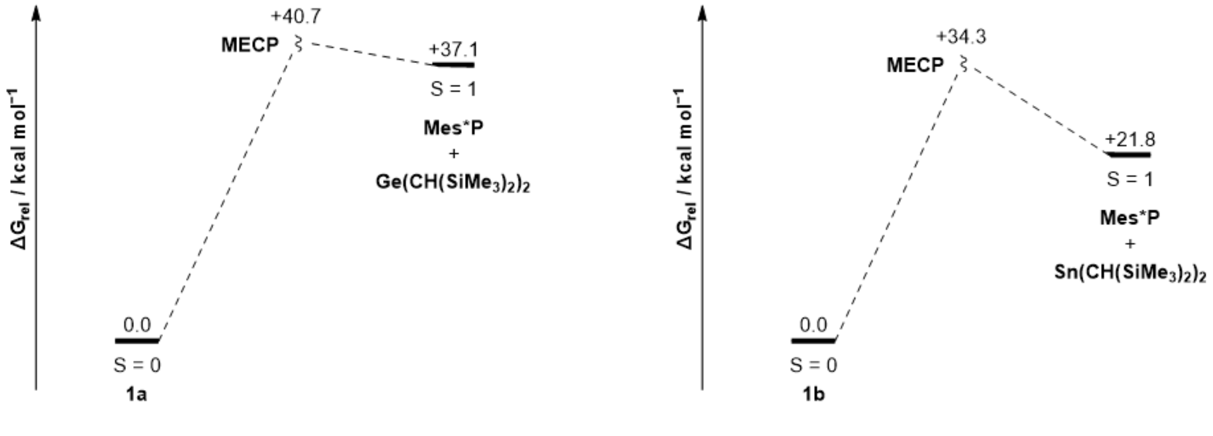

Figure S84. Dissociation of **1a** and **1b** to triplet phosphinidene Mes*P and the corresponding tetrylene.

3.4. XYZ coordinates

Mes*P=Ge[CH(SiMe$_3$)$_2$]$_2$

P	0.0441529819	-0.0540589192	0.1169990214
Ge	0.0420369936	-0.0237195347	2.2884326197
Si	-0.3553383113	-3.2966414127	2.3017400194
Si	-2.7089341504	-1.3186028862	3.3751112337
Si	1.7000005671	0.3703933785	5.1197545433
Si	0.2055616635	2.9532065174	3.8209655530
C	-0.8305804899	-1.6147382647	3.1088028285
H	-0.4112656724	-1.6572146691	4.1287552192
C	0.9725205126	1.2039375896	3.5570513155
H	1.8600546638	1.4600903723	2.9513197643
C	1.4091183864	-3.3359108081	1.5985685046
H	2.1714359617	-3.0391454999	2.3329766730
H	1.5013776782	-2.6749909069	0.7263454810
H	1.6356182018	-4.3657111039	1.2769654436
C	-1.5205586759	-3.7349018329	0.8712961762
H	-1.4812982819	-2.9477156816	0.1026452271
H	-2.5654744469	-3.8584963787	1.1906576665
H	-1.1944646583	-4.6789216080	0.4065737734
C	-0.4281452667	-4.6289070090	3.6591542143
H	-0.1201731675	-5.6012467424	3.2419583829
H	-1.4316288225	-4.7474387107	4.0898762191
H	0.2669495415	-4.3843818218	4.4786822044
C	-2.9372062266	0.1319962493	4.5824664936
H	-2.4616629600	1.0453759904	4.1975967535
H	-2.5036502793	-0.0846072535	5.5697363065
H	-4.0090104437	0.3460491739	4.7202939217
C	-3.6098931059	-0.8671871443	1.7724655422
H	-4.6960737605	-0.8449285937	1.9569703352
H	-3.4139332199	-1.5759606925	0.9570584538
H	-3.3058788079	0.1300680564	1.4255519938
C	-3.5321997404	-2.8430894978	4.1574266071
H	-4.5802291560	-2.6048614555	4.4007246340
H	-3.0271847985	-3.1342654340	5.0913113964
H	-3.5328238528	-3.7115467289	3.4827860058
C	2.9423363150	1.5717139402	5.9137470662
H	2.4581640844	2.4814713504	6.2966991326
H	3.7111601707	1.8760102100	5.1864356605
H	3.4511675129	1.0784145141	6.7573213289
C	2.6570717815	-1.2129847859	4.6897424536
H	3.1024940972	-1.6283411240	5.6080355860
H	3.4708076820	-1.0190443826	3.9764193106
H	2.0042194754	-1.9840508069	4.2583552838
C	0.4038606101	-0.1034295999	6.4248153506
H	0.9284012172	-0.4845941247	7.3159578963
H	-0.2698984238	-0.8984226184	6.0746723491
H	-0.2134968027	0.7490761438	6.7351251683
C	1.6392129715	4.1859280561	3.6528592172
H	1.2775580487	5.2199476548	3.7661807123
H	2.0929466516	4.0890653727	2.6556601644
H	2.4216661884	4.0145644976	4.4058843337
C	-1.0813391680	3.3533381062	2.4956935046
H	-1.9618768608	2.6969557040	2.5599264187
H	-0.6475574146	3.2449890399	1.4934505265
H	-1.4261462289	4.3938160417	2.6083378137
C	-0.6017045886	3.2010791683	5.5240935630
H	-1.0071126670	4.2251403342	5.5662090888
H	0.1161922297	3.0943681864	6.3502193469
H	-1.4350782409	2.5059959142	5.6983343378
C	0.8629822510	1.5775111065	-0.3420131950
C	0.0565973336	2.6364749081	-0.8921383401
C	0.5683700206	3.9435769059	-0.8486425081
H	-0.0468614657	4.7593326694	-1.2195053151
C	1.8374055289	4.2578313280	-0.3363199356
C	2.6742793419	3.1785466350	-0.0277966136
H	3.7050704566	3.3836427132	0.2461995606
C	2.2523707764	1.8332079036	-0.0950326913
C	-1.3020105897	2.4045888770	-1.6249782989
C	-1.7251822211	3.6499909586	-2.4418186717
H	-2.6301275200	3.4072713749	-3.0186715421
H	-0.9451282872	3.9590235627	-3.1537476123
H	-1.9731485678	4.5080808331	-1.7996270171
C	-1.1385082448	1.2526621236	-2.6503669152
H	-0.9401747528	0.2874319233	-2.1658931531
H	-0.3089064686	1.4669670249	-3.3409643638
H	-2.0615708163	1.1423379906	-3.2407767656
C	-2.4628014654	2.0960285723	-0.6517597148
H	-3.3921950049	1.9258942393	-1.2183109182
H	-2.6292824351	2.9375206955	0.0357430527
H	-2.2548247780	1.2006752870	-0.0559939679
C	2.2921824855	5.7267421034	-0.2645376512
C	1.2379226011	6.5619004690	0.5029353156
H	0.2581587701	6.5400224161	0.0047632278
H	1.5564118878	7.6143034057	0.5668637296
H	1.1033959936	6.1789566390	1.5238155377
C	3.6499456254	5.8851977005	0.4495327624
H	3.9133693892	6.9516723178	0.5149621094
H	4.4608010109	5.3794009859	-0.0955626095
H	3.6183201008	5.4830548294	1.4729364126
C	2.4294657708	6.2760882546	-1.7074192602
H	1.4744657489	6.2197503131	-2.2496023520
H	3.1735366622	5.6962120627	-2.2744657186
H	2.7509927903	7.3298450872	-1.6913210045
C	3.3633539889	0.7319351255	-0.0582197518
C	3.5639137409	0.1029314305	1.3370306185
H	4.4011990067	-0.6126530442	1.3135419327
H	2.6702773635	-0.4404251854	1.6614010517
H	3.8012263557	0.8742186354	2.0858491115

C	4.7320733933	1.3299483576	-0.4806085508
H	5.1474948896	2.0165163456	0.2713720716
H	4.6622518534	1.8665880597	-1.4384069632
H	5.4575999227	0.5115451766	-0.6007025055
C	3.0529015821	-0.3796114684	-1.0900199534
H	2.9183240623	0.0488661435	-2.0944068996
H	2.1428175265	-0.9389782931	-0.8367303519
H	3.8869568878	-1.0972290300	-1.1252815239

Mes*P=Sn[CH(SiMe$_3$)$_2$]$_2$

P	0.1161486467	-0.1065891288	-0.0991472676
Sn	0.0242815092	-0.0337314529	2.2639461008
Si	-0.4415808824	-3.4301062857	2.3559186292
Si	-2.7811502619	-1.4358526872	3.4544558344
Si	1.7617636303	0.4665968590	5.1942108818
Si	0.2511382046	3.0462892593	3.8767606015
C	-0.9217572440	-1.7822691983	3.1998747235
H	-0.4745339049	-1.8085799937	4.2086162486
C	1.0552278278	1.3250671064	3.6500330136
H	1.9354147597	1.5550164555	3.0225542516
C	1.3538624733	-3.4487693891	1.7299733501
H	2.0792982369	-3.1865884118	2.5144603303
H	1.4898638127	-2.7508222738	0.8913584250
H	1.5985596996	-4.4630467354	1.3744229353
C	-1.5549769180	-3.7837021499	0.8620105791
H	-1.4966834860	-2.9570168220	0.1368409088
H	-2.6086967668	-3.9246576725	1.1441992945
H	-1.2158227499	-4.7010923009	0.3552359544
C	-0.5891921055	-4.8235884185	3.6436642744
H	-0.2720552800	-5.7812834735	3.2003292276
H	-1.6147165176	-4.9448469662	4.0189073014
H	0.0664138048	-4.6217829683	4.5062707446
C	-2.9826074340	0.0208142676	4.6629411089
H	-2.4885425415	0.9267602292	4.2803166087
H	-2.5558356345	-0.2012527880	5.6521576756
H	-4.0501699532	0.2569440259	4.7978676439
C	-3.6484296723	-0.9440912567	1.8412113622
H	-4.7302657092	-0.8413692759	2.0241238243
H	-3.5056117704	-1.6821003568	1.0403895808
H	-3.2783612607	0.0226324011	1.4692371285
C	-3.6681875110	-2.9401509255	4.2051075257
H	-4.7094023980	-2.6734656808	4.4479630974
H	-3.1772792468	-3.2704287875	5.1335564805
H	-3.6928866084	-3.7923797896	3.5099686242
C	2.9681016070	1.6582327736	6.0560133264
H	2.4550841889	2.5545313067	6.4346119733
H	3.7582114687	1.9873912949	5.3630441684
H	3.4522959148	1.1586617055	6.9104183557
C	2.7393013845	-1.0897329617	4.7101763021
H	3.2217453821	-1.5200890628	5.6023684724
H	3.5250549779	-0.8626141201	3.9749064286
H	2.0878707493	-1.8639004779	4.2795737636
C	0.4354816315	-0.0560624970	6.4478925027
H	0.9245832746	-0.5475897451	7.3042261859
H	-0.2790918436	-0.7708062240	6.0168013883
H	-0.1354543193	0.8003980811	6.8303368038
C	1.6627567419	4.3124006952	3.7768118310
H	1.2800530190	5.3409454255	3.8686206557
H	2.1633500228	4.2194478081	2.8014598594
H	2.4141058035	4.1553656703	4.5642433646
C	-0.9742180104	3.4253278814	2.4832441898
H	-1.8265612546	2.7281072798	2.4740816869
H	-0.4777641887	3.3676365002	1.5043951392
H	-1.3767248090	4.4447685883	2.5973804080
C	-0.6487306505	3.2549088396	5.5370529468
H	-1.0791983111	4.2685722415	5.5789449366
H	0.0307746315	3.1468309660	6.3951946865
H	-1.4733843689	2.5377213534	5.6589538119
C	0.9093205763	1.5597648871	-0.4871749807
C	0.0850939793	2.6317986129	-0.9851541157
C	0.5786607509	3.9429282234	-0.8871332676
H	-0.0499919689	4.7643196679	-1.2205336113
C	1.8447835381	4.2552471512	-0.3663809312
C	2.6940882855	3.1768733329	-0.0925204789
H	3.7193390152	3.3867984459	0.1980570634
C	2.2924088026	1.8289054741	-0.2151977701
C	-1.2832502040	2.4213263465	-1.7064552695
C	-1.7213481690	3.6934837747	-2.4738652394
H	-2.6298243372	3.4657230286	-3.0511622724
H	-0.9496513107	4.0334571115	-3.1808379348
H	-1.9690684148	4.5261979550	-1.7990485790
C	-1.1377695942	1.3064438306	-2.7736774752
H	-0.9210400469	0.3280186215	-2.3251187321
H	-0.3266469187	1.5504316546	-3.4763217262
H	-2.0740015356	1.2083077297	-3.3452623707
C	-2.4279624580	2.0820509887	-0.7230536402
H	-3.3748216379	1.9685469955	-1.2749229701
H	-2.5560535755	2.8866774028	0.0156082843
H	-2.2290927417	1.1489977906	-0.1847291751
C	2.2844385192	5.7263148344	-0.2542231810
C	1.2227215004	6.5328938057	0.5329365912
H	0.2432868958	6.5144956565	0.0340139259
H	1.5319007929	7.5861981802	0.6222747737
H	1.0912312236	6.1251227749	1.5443226085
C	3.6414959157	5.8799864340	0.4621647534
H	3.8929034322	6.9471279070	0.5570266592
H	4.4571149418	5.3988214871	-0.0979368298
H	3.6171477409	5.4496310925	1.4741395248
C	2.4166039382	6.3126564523	-1.6832219399
H	1.4619517304	6.2613886347	-2.2264782722

H	3.1654559347	5.7532408530	-2.2643346435
H	2.7291820649	7.3684026320	-1.6410320619
C	3.4225688884	0.7476817207	-0.1768401248
C	3.6279880549	0.1380332155	1.2271568138
H	4.4819430423	-0.5580608364	1.2200842845
H	2.7441899936	-0.4245983863	1.5490670163
H	3.8378483007	0.9228608411	1.9699718690
C	4.7804472042	1.3724615731	-0.5986143333
H	5.1839117831	2.0662077952	0.1530538786
H	4.6979608823	1.9093417339	-1.5552827537
H	5.5221686277	0.5690783696	-0.7211234154
C	3.1459056897	-0.3794902572	-1.2005580903
H	2.9978299482	0.0378976445	-2.2077783704
H	2.2545969980	-0.9671492545	-0.9438003019
H	4.0031541310	-1.0695426668	-1.2320933800

NH_3

N	-0.0000000000	-0.0160816894	-0.0453950036
H	0.0000000000	1.0032924793	0.0229423291
H	-0.8126178305	-0.3236053949	0.4920563373
H	0.8126178305	-0.3236053949	0.4920563372

[GeP.NH_3] TS

P	0.1152384316	0.0330061793	2.5606730858
H	-0.3514868049	-1.4336721492	1.7375186875
Ge	0.0374416829	0.1344553612	0.1873879449
N	-0.1991412682	-1.8800002571	0.4977795541
H	-0.9941672970	-2.3226477342	0.0408557955
H	0.6129512907	-2.4869934134	0.4012587784
C	-1.2395028128	1.1000628809	3.3231980741
C	-2.6417230552	0.7704504029	3.2768509383
C	-3.5712041913	1.8159018691	3.4129717434
H	-4.6291002552	1.5923772937	3.2916338109
C	-3.2077707935	3.1341344558	3.7297494986
C	-1.8680121844	3.3426028515	4.0752694859
H	-1.5784788038	4.3166076336	4.4553123928
C	-0.8735456524	2.3552460698	3.9231080061
C	-3.2178780396	-0.6812604943	3.2436246286
C	-2.3547858317	-1.6152709561	4.1276483226
H	-1.3271753434	-1.7022605519	3.7572629411
H	-2.7982417452	-2.6233601554	4.1529717879
H	-2.3141578976	-1.2315321148	5.1581174091
C	-3.3335700954	-1.2371808890	1.8108980328
H	-3.9914839816	-0.5998510156	1.2003294685
H	-3.7599256038	-2.2535155673	1.8221874085
H	-2.3569260808	-1.2859902347	1.3312770719
C	-4.6439739180	-0.7289688122	3.8545953927
H	-4.6721605002	-0.2696615219	4.8535250060
H	-4.9536878465	-1.7803995735	3.9538975449
H	-5.3964521354	-0.2332302329	3.2240134847
C	-4.2756160020	4.2400310914	3.7892978089
H	-4.7357165108	3.8732703450	5.9015969861
C	0.5391256927	2.6680113592	4.5104459635
C	1.0166459935	1.4730254954	5.3761130005
H	0.2778529666	1.2497676246	6.1608521473
H	1.9737724883	1.7206328428	5.8620787798
H	1.1691373918	0.5687659151	4.7737685145
C	1.5697020027	2.9870399363	3.4072868237
H	2.5620552265	3.1727722290	3.8489266397
H	1.2690000536	3.8900134381	2.8554307369
H	1.6544847613	2.1519006748	2.7039541424
C	0.5049214452	3.8937556513	5.4575510749
H	-0.2383098529	3.7733623539	6.2601832980
H	0.2954359681	4.8337332705	4.9257601867
H	1.4934232160	4.0050738139	5.9278637546
C	-3.6563909677	5.6302634885	4.0374876778
H	-3.1613328993	5.6893109891	5.0182113576
H	-2.9166009506	5.8824219287	3.2626252236
H	-4.4440528146	6.3987455855	4.0181906466
C	-5.0421894774	4.2885431857	2.4447953990
H	-4.3584921473	4.5153281346	1.6140457381
H	-5.5349083039	3.3318336644	2.2203583648
H	-5.8193625390	5.0684619007	2.4742179071
C	-5.2660092283	3.9284247184	4.9387600563
H	-6.0352495286	4.7140747070	5.0122754781
H	-5.7783000065	2.9678299247	4.7818986703
C	1.7175278616	0.3273929166	-0.9118102700
Si	2.8480784280	1.8455195444	-0.5781065474
C	4.0393209857	1.4887870323	0.8568957288
H	3.4869618125	1.1200300641	1.7335986957
H	4.5565493161	2.4160832875	1.1493386869
H	4.8050793168	0.7434625019	0.6003546621
C	1.9630137644	3.4721929367	-0.1795144535
H	1.4507361179	3.4527879838	0.7854959341
H	2.7353707077	4.2571277674	-0.1289338478
H	1.2410369229	3.7647362283	-0.9519873271
C	3.7922034552	2.2369913235	-2.1861775109
H	4.4126642649	3.1346815348	-2.0324165796
H	4.4453740285	1.4291103606	-2.5366176187
H	3.0740735111	2.4678067709	-2.9901818386
Si	2.7396759731	-1.2960925580	-0.9631223077
C	3.0784320824	-1.9188996070	0.8104141137
H	2.3841644389	-1.5123541541	1.5615894552
H	4.0887585389	-1.6129490315	1.1202717706
H	3.0432231732	-3.0191764748	0.8615265862
C	4.4303925980	-1.1436999741	-1.8259664734
H	5.1248977255	-0.4575013470	-1.3233680506
H	4.8916412829	-2.1452112287	-1.8261024258
H	4.3243715681	-0.8264603695	-2.8741500442
C	1.8611800754	-2.6355350429	-2.0043121052

H	0.7853829534	-2.7254140202	-1.8119517227
H	2.3285082140	-3.6151636598	-1.8126117442
H	1.9908236801	-2.4178580371	-3.0760606723
H	1.3268147599	0.4874168703	-1.9311721627
C	-1.6190597247	0.6124999649	-0.8414646560
H	-2.4064086295	0.3035221908	-0.1324672313
Si	-1.8947785282	2.5031660207	-0.9770433476
C	-1.3815142235	3.3547311946	0.6302167620
H	-0.4737944036	2.9463037668	1.0858794288
H	-2.1839600057	3.2228709984	1.3677340918
H	-1.2328383217	4.4350149838	0.4778235994
C	-3.7461085933	2.9101472163	-1.1522107627
H	-4.3265395958	2.4035816199	-0.3650970786
H	-3.8799332407	3.9947622359	-1.0088410811
H	-4.1736436551	2.6428229203	-2.1261481202
C	-0.9800204135	3.1943620497	-2.4959820772
H	0.0621922642	2.8483032252	-2.5469386657
H	-0.9704536867	4.2954285804	-2.4724457799
H	-1.4808970215	2.8848530145	-3.4259725016
Si	-1.9722566587	-0.3762474583	-2.4381407604
C	-2.3390250586	-2.2142043993	-2.0688892887
H	-3.0080467683	-2.3265773870	-1.2007267843
H	-1.4335521164	-2.8143650414	-1.8981717705
H	-2.8535780085	-2.6554871617	-2.9373222907
C	-3.5459466711	0.2543551497	-3.3054033573
H	-4.4110583411	0.2729979617	-2.6258847861
H	-3.4191351826	1.2609996580	-3.7279265686
H	-3.7837749764	-0.4284641318	-4.1371944615
C	-0.5756334824	-0.2868083013	-3.7215166656
H	0.3430825709	-0.7764453945	-3.3762355362
H	-0.3338688391	0.7525537961	-3.9858208281
H	-0.9014214906	-0.8004424841	-4.6403540854

GeP.NH_3

P	-0.1793893877	0.0752375505	0.1682455260
H	0.7998103011	-0.9450415137	0.1521184512
Ge	-0.0302623969	0.0149041816	2.5929747930
N	1.6922999500	-0.1917754721	3.3155393089
H	2.1751366295	-1.0206717786	2.9788475221
H	2.2950468096	0.6030731667	3.1152639266
Si	0.1502119724	-3.2487292581	3.0942958449
Si	-2.5987827940	-2.0444443342	1.9363468767
Si	0.5579123908	3.0162160521	3.7021968970
Si	-1.5095111251	1.1561196894	5.2886781045
C	-0.9954187366	-1.7176442911	2.9469547294
H	-1.3618423958	-1.5718631897	3.9779155916
C	-0.7300437571	1.5928534056	3.5911332343
H	-1.5624417416	1.9920390719	2.9903699233
C	1.2046871981	-3.1606393496	4.6749981915
H	1.7186519947	-2.1984575799	4.7978956942
H	1.9532032349	-3.9696933883	4.6679363130
H	0.5634945414	-3.3142046338	5.5578862478
C	1.2681141423	-3.4486419652	1.5684059774
H	1.9914427351	-2.6324419335	1.4409304484
H	0.6699431907	-3.5076507782	0.6471765110
H	1.8402134959	-4.3858999452	1.6590598136
C	-0.8124807374	-4.8836290100	3.2685855244
H	-0.0773563121	-5.6965686198	3.3869596409
H	-1.4427199762	-5.1258977838	2.4022211327
H	-1.4477033005	-4.8793493598	4.1671713915
C	-3.7933923959	-3.0751117626	3.0062991522
H	-4.7229652020	-3.2452540411	2.4391203277
H	-4.0652385169	-2.5427276566	3.9308700981
H	-3.3856384396	-4.0543354964	3.2866919461
C	-3.5583506943	-0.4706122920	1.4614824742
H	-4.6206916372	-0.7306170592	1.3269618908
H	-3.1871593888	-0.0581177743	0.5153844020
H	-3.5030134465	0.3152693200	2.2258544295
C	-2.2616323281	-2.9928640231	0.3240066476
H	-1.8059965431	-3.9789354225	0.4906784198
H	-1.6031554262	-2.4100421055	-0.3366954202
H	-3.2155185559	-3.1480956230	-0.2055503093
C	1.7081650361	2.8008945087	5.2023978840
H	2.4821079523	3.5848373515	5.1821003162
H	2.2076541529	1.8216572274	5.1682075008
H	1.1829914688	2.8755970328	6.1647300651
C	1.6836739526	3.1638826845	2.1814909289
H	1.1202834049	3.4479932867	1.2843432845
H	2.2441532701	2.2549916154	1.9265982465
H	2.4193507324	3.9598558803	2.3807190597
C	-0.3403280864	4.6901646964	3.8146244440
H	-0.9449156379	4.7959773659	4.7246509679
H	0.4048602235	5.5021452855	3.8059084412
H	-1.0012030231	4.8385411372	2.9462083513
C	-3.2319440490	0.3766470927	5.0769614755
H	-3.9043508264	1.0572967842	4.5314282399
H	-3.6707220504	0.2053286709	6.0732986318
H	-3.2241377770	-0.5825648396	4.5471826189
C	-0.4143562158	-0.0037844224	6.3118270117
H	-0.2864508749	-0.9832140715	5.8339324746
H	-0.8726971016	-0.1628001670	7.3011551132
H	0.5888384293	0.4179916823	6.4586229682
C	-1.8550676563	2.7154824393	6.3296893467
H	-0.9512387679	3.2701347024	6.6154473469
H	-2.3631245911	2.4042056734	7.2570665416
H	-2.5283882464	3.4054759080	5.7982445745
C	0.7638643171	1.4798592234	-0.6478991869
C	2.1633581501	1.4407557310	-0.9978998527
C	2.7965310044	2.6483514646	-1.3345960204
H	3.8633896545	2.6349128612	-1.5383197793

C	2.1323608587	3.8781963236	-1.4269669337
C	0.7420983834	3.8426167807	-1.2992906799
H	0.1900671795	4.7597488311	-1.4667232639
C	0.0254211340	2.6815196332	-0.9467912682
C	3.0308655552	0.1493265484	-1.1131667651
C	2.3123763619	-0.8962615033	-2.0079708184
H	1.3138601590	-1.1730407604	-1.6572645658
H	2.9134008452	-1.8169761114	-2.0681621228
H	2.1999364602	-0.4942365513	-3.0262065258
C	3.3961017498	-0.4063040393	0.2816545907
H	4.0238367533	0.3207055030	0.8197535713
H	3.9629220849	-1.3460232260	0.1893626636
H	2.5172864781	-0.5931149205	0.8997010100
C	4.3855394709	0.4096398807	-1.8234493970
H	4.2521453462	0.8555522516	-2.8200591543
H	4.9039663139	-0.5520179460	-1.9544459747
H	5.0532599941	1.0565647854	-1.2360888289
C	2.9227653325	5.1668205827	-1.7095667756
H	2.8983089726	4.8937014449	-3.8863864593
C	-1.5322861007	2.8002837441	-0.9546191937
C	-2.1460627286	1.7176962983	-1.8799678876
H	-1.7722301949	1.8436333020	-2.9073761499
H	-3.2426201217	1.8178211745	-1.8976439146
H	-1.9042516580	0.7004378667	-1.5510216040
C	-2.1007970457	2.7075705606	0.4763667900
H	-3.1937227327	2.8396965671	0.4714155306
H	-1.6590216475	3.4891208081	1.1106435669
H	-1.8923820064	1.7391649601	0.9363114077
C	-2.0198759933	4.1579357563	-1.5197176011
H	-1.6515119726	4.3389737981	-2.5401906651
H	-1.7295570186	5.0081786842	-0.8847562990
H	-3.1190703043	4.1446913016	-1.5605198314
C	2.0156104968	6.4130032021	-1.7375842381
H	1.2674782334	6.3552792059	-2.5424919281
H	1.4849326423	6.5478505535	-0.7828281671
H	2.6240021181	7.3128154698	-1.9139543232
C	3.9792249454	5.3600819261	-0.5931228458
H	3.4931534146	5.4354802842	0.3909712200
H	4.6856244005	4.5186372029	-0.5547284710
H	4.5584621082	6.2808260790	-0.7663176618
C	3.6326268697	5.0458289434	-3.0808109723
H	4.2034055236	5.9617548995	-3.3017135189
H	4.3338791431	4.1992081029	-3.1005582133

[SnP.NH_3] TS

P	0.0805668809	-0.0409994895	2.7310437591
H	-0.4287076209	-1.4666020812	1.8776940594
Sn	0.0601226455	0.1795286900	0.1932747663
N	-0.2368454761	-1.9848840340	0.5913674183
H	-1.0165688824	-2.4471812441	0.1249839223
H	0.5777266017	-2.5950650955	0.5325210705
C	-1.2653117944	1.0723426243	3.4390254665
C	-2.6692620448	0.7509961903	3.4110635832
C	-3.5936994070	1.8014620355	3.5393238241
H	-4.6536316830	1.5775207937	3.4417437217
C	-3.2226996622	3.1287976097	3.8023393519
C	-1.8732899338	3.3507682287	4.0957225628
H	-1.5734313603	4.3410568079	4.4210018582
C	-0.8828827943	2.3560061784	3.9670069502
C	-3.2535360992	-0.6946430747	3.3539233248
C	-2.4154345770	-1.6507544145	4.2384063091
H	-1.3795180970	-1.7426700261	3.8933822152
H	-2.8650338677	-2.6563136733	4.2353715365
H	-2.3968156292	-1.2862797526	5.2765655582
C	-3.3486966006	-1.2119669412	1.9034239770
H	-4.0054239257	-0.5594245527	1.3078452031
H	-3.7675241468	-2.2311009675	1.8798537561
H	-2.3680635096	-1.2400033903	1.4280959177
C	-4.6904649370	-0.7459545808	3.9381632920
H	-4.7345626007	-0.3065303913	4.9455751248
H	-5.0073275842	-1.7972823246	4.0114066412
H	-5.4289700922	-0.2342491726	3.3039680412
C	-4.2887644014	4.2369223517	3.8475077559
H	-4.7219689239	3.9254143622	5.9748320469
C	0.5446720849	2.7037323425	4.4973607359
C	1.0335893938	1.5787330688	5.4462234779
H	0.3225916571	1.4438222326	6.2756570104
H	2.0136927418	1.8472987473	5.8712303104
H	1.1435612247	0.6207774001	4.9234807750
C	1.5544375967	2.9316242497	3.3522292990
H	2.5480073063	3.1781369143	3.7599515291
H	1.2305158189	3.7714897014	2.7197354840
H	1.6520689548	2.0366987972	2.7285219781
C	0.5415159651	4.0013490763	5.3444943434
H	-0.1815916587	3.9513493482	6.1722211671
H	0.3245014177	4.8970183803	4.7435856923
H	1.5421615561	4.1408814717	5.7801231916
C	-3.6665525241	5.6328746018	4.0510276956
H	-3.1544409379	5.7155855913	5.0213108406
H	-2.9408103578	5.8678431772	3.2576810488
H	-4.4553018826	6.4000748859	4.0270186430
C	-5.0734197798	4.2496120425	2.5123440631
H	-4.4010879934	4.4532878552	1.6665856804
H	-5.5697557590	3.2873953895	2.3215347029
H	-5.8500138181	5.0304572162	2.5314396147
C	-5.2643376750	3.9564186657	5.0176800274
H	-6.0315649773	4.7448629760	5.0802256611
H	-5.7796435046	2.9930045449	4.8918705901
C	1.9216197038	0.3071257987	-1.0109112546
Si	3.0283486319	1.8197694883	-0.6397039677

C	4.1431588086	1.4872495045	0.8602699484
H	3.5553738728	1.1236412508	1.7158732511
H	4.6449407420	2.4182717984	1.1676542027
H	4.9225007761	0.7425002324	0.6425828025
C	2.0392781828	3.3991794383	-0.2893262937
H	1.4444056510	3.3122019985	0.6263927674
H	2.7424536948	4.2351229701	-0.1448428152
H	1.3651315204	3.6657427552	-1.1146953628
C	4.0782989773	2.2152327579	-2.1771285503
H	4.6416537402	3.1474043941	-2.0087770662
H	4.7979592460	1.4248944980	-2.4263313036
H	3.4285414430	2.3736850932	-3.0531318645
Si	2.8603684084	-1.3540793132	-0.9997177329
C	3.1528195438	-1.9526669296	0.7885847932
H	2.3931873937	-1.6104845470	1.5075718168
H	4.1193584609	-1.5750758024	1.1538934654
H	3.1961610638	-3.0530621260	0.8299293464
C	4.5698395098	-1.2585097233	-1.8333110590
H	5.2673552320	-0.5954766416	-1.3026179642
H	5.0096802394	-2.2693035902	-1.8415669368
H	4.4929166099	-0.9171534480	-2.8766848601
C	1.9216823205	-2.6691229724	-2.0119021660
H	0.8641973566	-2.7621829256	-1.7334798948
H	2.3998637763	-3.6530037505	-1.8784348125
H	1.9672705379	-2.4237357482	-3.0846071610
H	1.5340446764	0.4643170667	-2.0323168885
C	-1.7741723617	0.6678028820	-0.9479994381
H	-2.5356088451	0.3074444978	-0.2342666955
Si	-2.0704997757	2.5479556297	-1.0430705448
C	-1.5592816934	3.3677919092	0.5841017566
H	-0.5404716558	3.1271116346	0.9105387138
H	-2.2321588071	3.0345203915	1.3854326316
H	-1.6358711425	4.4642202000	0.5109480479
C	-3.9238652506	2.9387463527	-1.2286623818
H	-4.5046160431	2.4271792958	-0.4450236742
H	-4.0795950455	4.0220618263	-1.0986043457
H	-4.3358221797	2.6527930803	-2.2049823240
C	-1.1290485153	3.2806616887	-2.5243514443
H	-0.0829640129	2.9416971969	-2.5468004650
H	-1.1274949283	4.3810435812	-2.4812139104
H	-1.5969083497	2.9820907237	-3.4749795736
Si	-2.0463376123	-0.3292526259	-2.5445584957
C	-2.3694696139	-2.1660850205	-2.1462592900
H	-3.0635363406	-2.2749174580	-1.2975527778
H	-1.4421111318	-2.7089830069	-1.9132922240
H	-2.8271945642	-2.6619816837	-3.0168796104
C	-3.5899922052	0.2719379916	-3.4834499735
H	-4.4850118322	0.2582978110	-2.8431913456
H	-3.4683362044	1.2900106766	-3.8805635609
H	-3.7731932673	-0.4008440399	-4.3369679203
C	-0.5786578219	-0.2232614022	-3.7452213401
H	0.3041034925	-0.7457974980	-3.3540852367
H	-0.2981707925	0.8189759603	-3.9551376241
H	-0.8505089465	-0.6994514640	-4.7008679685

$SnP.NH_3$

P	0.1161486467	-0.1065891288	-0.0991472676
Sn	0.0242815092	-0.0337314529	2.2639461008
Si	-0.4415808824	-3.4301062857	2.3559186292
Si	-2.7811502619	-1.4358526872	3.4544558344
Si	1.7617636303	0.4665968590	5.1942108818
Si	0.2511382046	3.0462892593	3.8767606015
C	-0.9217572440	-1.7822691983	3.1998747235
H	-0.4745339049	-1.8085799937	4.2086162486
C	1.0552278278	1.3250671064	3.6500330136
H	1.9354147597	1.5550164555	3.0225542516
C	1.3538624733	-3.4487693891	1.7299733501
H	2.0792982369	-3.1865884118	2.5144603303
H	1.4898638127	-2.7508222738	0.8913584250
H	1.5985596996	-4.4630467354	1.3744229353
C	-1.5549769180	-3.7837021499	0.8620105791
H	-1.4966834860	-2.9570168220	0.1368409088
H	-2.6086967668	-3.9246576725	1.1441992945
H	-1.2158227499	-4.7010923009	0.3552359544
C	-0.5891921055	-4.8235884185	3.6436642744
H	-0.2720552800	-5.7812834735	3.2003292276
H	-1.6147165176	-4.9448469662	4.0189073014
H	0.0664138048	-4.6217829683	4.5062707446
C	-2.9826074340	0.0208142676	4.6629411089
H	-2.4885425415	0.9267602292	4.2803166087
H	-2.5558356345	-0.2012527880	5.6521576756
H	-4.0501699532	0.2569440259	4.7978676439
C	-3.6484296723	-0.9440912567	1.8412113622
H	-4.7302657092	-0.8413692759	2.0241238243
H	-3.5056117704	-1.6821003568	1.0403895808
H	-3.2783612607	0.0226324011	1.4692371285
C	-3.6681875110	-2.9401509255	4.2051075257
H	-4.7094023980	-2.6734656808	4.4479630974
H	-3.1772792468	-3.2704287875	5.1335564805
H	-3.6928866084	-3.7923797896	3.5099686242
C	2.9681016070	1.6582327736	6.0560133264
H	2.4550841889	2.5545313067	6.4346119733
H	3.7582114687	1.9873912949	5.3630441684
H	3.4522959148	1.1586617055	6.9104183557
C	2.7393013845	-1.0897329617	4.7101763021
H	3.2217453821	-1.5200890628	5.6023684724
H	3.5250549779	-0.8626141201	3.9749064286
H	2.0878707493	-1.8639004779	4.2795737636
C	0.4354816315	-0.0560624970	6.4478925027
H	0.9245832746	-0.5475897451	7.3042261859

H	-0.2790918436	-0.7708062240	6.0168013883
H	-0.1354543193	0.8003980811	6.8303368038
C	1.6627567419	4.3124006952	3.7768118310
H	1.2800530190	5.3409454255	3.8686206557
H	2.1633500228	4.2194478081	2.8014598594
H	2.4141058035	4.1553656703	4.5642433646
C	-0.9742180104	3.4253278814	2.4832441898
H	-1.8265612546	2.7281072798	2.4740816869
H	-0.4777641887	3.3676365002	1.5043951392
H	-1.3767248090	4.4447685883	2.5973804080
C	-0.6487306505	3.2549088396	5.5370529468
H	-1.0791983111	4.2685722415	5.5789449366
H	0.0307746315	3.1468309660	6.3951946865
H	-1.4733843689	2.5377213534	5.6589538119
C	0.9093205763	1.5597648871	-0.4871749807
C	0.0850939793	2.6317986129	-0.9851541157
C	0.5786607509	3.9429282234	-0.8871332676
H	-0.0499919689	4.7643196679	-1.2205336113
C	1.8447835381	4.2552471512	-0.3663809312
C	2.6940882855	3.1768733329	-0.0925204789
H	3.7193390152	3.3867984459	0.1980570634
C	2.2924088026	1.8289054741	-0.2151977701
C	-1.2832502040	2.4213263465	-1.7064552695
C	-1.7213481690	3.6934837747	-2.4738652394
H	-2.6298243372	3.4657230286	-3.0511622724
H	-0.9496513107	4.0334571115	-3.1808379348
H	-1.9690684148	4.5261979550	-1.7990485790
C	-1.1377695942	1.3064438306	-2.7736774752
H	-0.9210400469	0.3280186215	-2.3251187321
H	-0.3266469187	1.5504316546	-3.4763217262
H	-2.0740015356	1.2083077297	-3.3452623707
C	-2.4279624580	2.0820509887	-0.7230536402
H	-3.3748216379	1.9685469955	-1.2749229701
H	-2.5560535755	2.8866774028	0.0156082843
H	-2.2290927417	1.1489977906	-0.1847291751
C	2.2844385192	5.7263148344	-0.2542231810
C	1.2227215004	6.5328938057	0.5329365912
H	0.2432868958	6.5144956565	0.0340139259
H	1.5319007929	7.5861981802	0.6222747737
H	1.0912312236	6.1251227749	1.5443226085
C	3.6414959157	5.8799864340	0.4621647534
H	3.8929034322	6.9471279070	0.5570266592
H	4.4571149418	5.3988214871	-0.0979368298
H	3.6171477409	5.4496310925	1.4741395248
C	2.4166039382	6.3126564523	-1.6832219399
H	1.4619517304	6.2613886347	-2.2264782722
H	3.1654559347	5.7532408530	-2.2643346435
H	2.7291820649	7.3684026320	-1.6410320619
C	3.4225688884	0.7476817207	-0.1768401248
C	3.6279880549	0.1380332155	1.2271568138

H	4.4819430423	-0.5580608364	1.2200842845
H	2.7441899936	-0.4245983863	1.5490670163
H	3.8378483007	0.9228608411	1.9699718690
C	4.7804472042	1.3724615731	-0.5986143333
H	5.1839117831	2.0662077952	0.1530538786
H	4.6979608823	1.9093417339	-1.5552827537
H	5.5221686277	0.5690783696	-0.7211234154
C	3.1459056897	-0.3794902572	-1.2005580903
H	2.9978299482	0.0378976445	-2.2077783704
H	2.2545969980	-0.9671492545	-0.9438003019
H	4.0031541310	-1.0695426668	-1.2320933800

Imidazole

N	0.0222906400	-0.0098050045	1.7976271075
N	-0.8105517260	0.5383730270	3.7882652644
C	-0.9489694027	0.6796112956	2.4824396994
C	0.8291982959	-0.6289537115	2.7348849759
C	0.2901408339	-0.2724605455	3.9608610303
H	0.1313039785	-0.0603786596	0.7924744970
H	-1.7189661889	1.2581911569	1.9731228099
H	1.6804485517	-1.2425868539	2.4531608829
H	0.6414450176	-0.5599307045	4.9508137328

[SnP.Im] TS

Sn	-1.0194009262	1.5933659899	-0.8626742832
P	1.3678739132	2.4290537553	-0.9875678967
Si	-3.9392420690	0.8994952218	0.9423878492
Si	-3.0064893892	4.0080053088	0.7109200685
Si	-0.8039592763	-1.3694698562	-2.7327251489
Si	-2.5984109859	1.1017756768	-3.7992572396
N	1.4462063851	-1.8111173720	1.3174179696
N	0.5176928356	0.1694913663	0.7125606741
C	-5.1895005987	0.8716726463	-0.4870803122
C	-4.8551365643	1.3691602475	2.5434327323
C	-3.3034849071	-0.8722334314	1.1776857969
C	-4.7615877462	4.3575045977	0.0676959186
C	-2.9291233623	4.5721433820	2.5237685689
C	-1.8229600145	5.1015080204	-0.2771175567
C	-2.5188571125	2.1456267407	0.6666840492
C	-0.8674487077	-2.6086086534	-1.2977430885
C	-1.5205246886	-2.3291263091	-4.2159212268
C	0.9552353033	-0.8377183628	-3.2103426726
C	-4.1132902395	0.1776546722	-4.4760569786
C	-3.2107935787	2.8199914042	-3.2571180006
C	-1.3139579875	1.3073596968	-5.1797662371
C	-1.8772715558	0.1540446893	-2.3013424541
C	2.3749543608	9.1114911827	0.9851999933
C	-0.0724205889	8.6134655738	1.3964574740
C	0.6959058006	9.3869691032	-0.8581979423
C	1.6989111209	6.1794383438	-4.0519839397

C	0.2799948141	4.1858739546	-3.7111499497
C	2.8146500805	4.0559193732	-3.4630311925
C	1.5434469005	4.9007789273	-3.1894565177
C	1.0800529448	8.5302209902	0.3647691539
C	2.7467808081	4.6910689764	3.1245139984
C	0.9261182416	3.1294555611	2.4917400849
C	3.3096155829	2.8745729943	1.5658466440
C	2.1747871462	3.8576535899	1.9486626234
C	1.4340031514	5.2264792186	-1.6659314929
C	1.2831630076	6.5856149591	-1.3246607511
C	1.3138521593	7.0567661060	-0.0093936080
C	1.6289193094	6.1207012451	0.9849376745
C	1.7655316522	4.7453840447	0.7372718136
C	1.5297332500	4.2596189925	-0.6013781560
C	1.3614735002	-0.8954264248	0.3778853430
C	0.6266820558	-1.3558605544	2.3443940022
C	0.0680428312	-0.1421491779	2.0041621744
H	-5.9737500268	0.1316701786	-0.2605779529
H	-5.6773210593	1.8428637675	-0.6407176686
H	-4.7203580487	0.5745879738	-1.4342475827
H	-5.6203688275	0.6094521375	2.7700561301
H	-5.3582301071	2.3440750147	2.4722411210
H	-4.1572093541	1.4079963801	3.3944474364
H	-4.1643590477	-1.5382272899	1.3513455708
H	-2.7621525435	-1.2526410374	0.3019621369
H	-2.6279409538	-0.9593570974	2.0402280105
H	-5.5415569573	3.8276724826	0.6324466575
H	-4.9500353884	5.4382041056	0.1740821992
H	-4.8738630265	4.1074883279	-0.9968886264
H	-3.6262931545	4.0087271472	3.1606384279
H	-3.1859946115	5.6411468703	2.5969475669
H	-1.9144279554	4.4428518575	2.9288375020
H	-2.1463142894	6.1508441780	-0.1918729577
H	-0.7961321105	5.0390933088	0.1032121894
H	-1.7999356331	4.8560495681	-1.3479251704
H	-1.8804143431	2.0520318935	1.5645717316
H	-2.7475062479	-0.2299291674	-1.7427785899
H	-1.8558870740	-3.0959926482	-1.2880381088
H	-0.1093022258	-3.3950677888	-1.4363819408
H	-0.6965915004	-2.1630309256	-0.3115384945
H	-2.5514568529	-2.6609841713	-4.0192613959
H	-0.9065013294	-3.2317167802	-4.3702348158
H	-1.5127649922	-1.7584602668	-5.1548244287
H	1.6441499207	-1.6954779383	-3.1597918358
H	0.9598576265	-0.4652976623	-4.2458039799
H	1.3566607121	-0.0302856155	-2.5797972192
H	-4.8838887439	0.0461623526	-3.7012422755
H	-3.8498107917	-0.8139864790	-4.8679497561
H	-4.5611603186	0.7598341631	-5.2978166458
H	-3.9495876778	2.7371226274	-2.4481011759
H	-3.6964174979	3.3318564273	-4.1031661931
H	-2.3968136669	3.4749668488	-2.9150016880
H	-1.1912685963	0.3774666857	-5.7530277643
H	-1.6374567720	2.0939956756	-5.8792224239
H	-0.3313029372	1.5931358478	-4.7826569758
H	2.6692065250	8.5664338618	1.8936773661
H	2.2318452336	10.1695383429	1.2567140367
H	-0.2582735609	9.6613095006	1.6806838984
H	0.1585743660	8.0517469378	2.3128371562
H	-1.0016349200	8.2007454485	0.9760041480
H	0.5072117989	10.4243407447	-0.5431143850
H	-0.2178670980	9.0100183591	-1.3419987577
H	1.4998815840	9.4087011442	-1.6089075713
H	1.8594525060	5.8781066220	-5.0977471219
H	0.8019950484	6.8168041804	-4.0361826725
H	2.5640678805	6.7854172636	-3.7441613438
H	0.1331720373	3.2242754869	-3.2082531058
H	-0.6103261157	4.8113231956	-3.5457166533
H	0.3641237138	3.9960698635	-4.7922714528
H	2.7763472126	3.0796025010	-2.9653838025
H	2.9226599350	3.8831612903	-4.5455762454
H	3.7092696748	4.5911364937	-3.1099647822
H	3.2089588217	9.0482156455	0.2700019695
H	1.9953451738	5.3444478536	3.5913269822
H	3.1003728129	4.0033269206	3.9071485250
H	3.6003178257	5.3100879684	2.8098087821
H	0.3773814283	2.6397922917	1.6842124731
H	1.2022983569	2.3691292022	3.2372567158
H	0.2477147465	3.8540848069	2.9652336511
H	4.1892162341	3.4342876763	1.2132930509
H	3.6068455941	2.2825730740	2.4451212785
H	3.0186344707	2.1711970833	0.7774629204
H	1.1359899676	7.3061035135	-2.1209799723
H	1.7605057686	6.4825700810	2.0009192149
H	1.9064743603	-0.9021549972	-0.5637931533
H	0.4962391239	-1.9259882667	3.2636533635
H	-0.5979117884	0.5162185364	2.5525384985
H	1.0189908294	1.2523500118	0.3451380140

SnP.Im

Sn	0.1667712958	-0.1964466699	2.5764907908
P	0.1053683390	-0.1245822536	-0.0213485123
Si	-1.5062865487	1.6514238344	4.9871199363
Si	1.1344836314	2.9949932286	3.5919191112
Si	-2.5523823433	-2.3472094549	1.9635088548
Si	-0.0140051079	-3.4955252773	3.5345081393
N	4.0942555906	-1.3680777899	4.0865773216
N	2.0443816238	-0.6230194446	3.5203269205
C	3.6236280389	5.5350984844	-2.3154224041
C	3.9145728690	5.4242557985	0.1961058663

C	1.9019689550	6.5371823430	-0.7932573600
C	-1.9391790624	4.0115566892	-1.3710433103
C	-2.1365363319	2.3572837924	0.4510676880
C	-1.8514220164	1.6182977402	-1.9732079740
C	-1.4179082575	2.6348540984	-0.8844747791
C	2.8893191719	5.3646241454	-0.9624947267
C	4.5788804772	0.7864463229	-2.0387082451
C	3.7090550724	-0.4438106209	-0.0822908130
C	2.5628178655	-0.5881134353	-2.3772787038
C	3.2705242680	0.3280696964	-1.3443582332
C	0.1400391465	2.6192269293	-0.7404042696
C	0.7924920435	3.8639743490	-0.8322208608
C	2.1793515356	4.0005165184	-0.9351677985
C	2.9110078084	2.8154844605	-1.0932557739
C	2.3482555166	1.5325177527	-0.9873786039
C	0.9529249291	1.4328592303	-0.6396510165
C	-1.9217240179	3.4112593234	5.5834630309
C	-0.8290685331	0.6370170433	6.4418419426
C	-3.1760611872	0.9105304691	4.4603939177
C	0.4196009777	4.6304372809	2.9295147266
C	2.6076569347	2.5371938423	2.5003238089
C	1.8046548954	3.2957450618	5.3491998857
C	-1.9923957208	-3.4551189483	0.5248776685
C	-3.4083092912	-0.8666167370	1.1356907401
C	-3.9145852554	-3.1829960997	2.9970783278
C	-1.0791276949	-5.0556849586	3.8005723724
C	1.1762301021	-3.8887882901	2.0950027897
C	0.8826709700	-3.2883517293	5.1908408891
C	-0.3159809169	1.7258543216	3.5016844777
C	-1.0747189726	-1.9455765195	3.1211580624
C	3.1508193695	-1.3567688642	3.1543009244
C	3.5896090307	-0.6124199692	5.1240856839
C	2.3252608524	-0.1472715249	4.7969789249
H	4.3927936375	4.7635131390	-2.4645515993
H	4.1210693618	6.5168121436	-2.3597531742
H	4.4607609336	6.3803510614	0.1750935027
H	4.6516427998	4.6111598014	0.1310217465
H	3.4063383645	5.3387688294	1.1672584494
H	2.4537255334	7.4891450935	-0.7861035447
H	1.3470360520	6.4648728105	0.1540111109
H	1.1760162528	6.5811056401	-1.6191060082
H	-3.0212900938	3.9325412707	-1.5520807104
H	-1.7957474773	4.8069115684	-0.6249941600
H	-1.4629153832	4.3221711728	-2.3122577701
H	-1.8508327637	1.3930451104	0.8782782903
H	-1.9034176670	3.1468172742	1.1796525442
H	-3.2273669848	2.3364411476	0.3069987886
H	-1.6011931253	0.5839018620	-1.7104025933
H	-2.9407717883	1.6723217163	-2.1222770072
H	-1.3601330364	1.8546753508	-2.9289357007
H	2.9136370200	5.4704560465	-3.1538820318
H	5.2430348010	1.3420466032	-1.3610217837
H	5.1319852149	-0.1049854071	-2.3693866730
H	4.3809604420	1.4071911469	-2.9250549933
H	2.8646842473	-0.8936065378	0.4425263878
H	4.4091540807	-1.2517906964	-0.3457273840
H	4.2093548831	0.2277075777	0.6296451127
H	2.3642934953	-0.0265310827	-3.3026406505
H	3.2101202965	-1.4426345528	-2.6284467960
H	1.6053508214	-0.9914458185	-2.0299731710
H	0.1880889406	4.7635561119	-0.8381433400
H	3.9750427378	2.8994486971	-1.2950642160
H	-2.3947903433	3.9881808250	4.7733532100
H	-2.6411049112	3.3542827697	6.4163018396
H	-1.0451955346	3.9740793615	5.9319363316
H	0.0634191621	1.0983792883	6.8900363297
H	-1.5979930612	0.5702515706	7.2280734496
H	-0.5645075891	-0.3892778685	6.1453435442
H	-3.1098625513	-0.1457312643	4.1707188289
H	-3.8913807111	0.9846020966	5.2951916866
H	-3.5963470011	1.4705957217	3.6104647841
H	0.1140747015	4.5074249687	1.8794892806
H	1.1853103174	5.4219469555	2.9635203059
H	-0.4497231416	4.9702246196	3.5110817827
H	3.3335514792	3.3644991441	2.5166946904
H	3.1170558340	1.6331655899	2.8571433411
H	2.3046870477	2.3848724383	1.4575418948
H	1.0265305910	3.3484810368	6.1226020499
H	2.5260273074	2.5177698087	5.6351899913
H	2.3398088536	4.2592818636	5.3517517794
H	-2.8476383494	-3.6395084474	-0.1446889282
H	-1.2162653607	-2.9417094846	-0.0639077088
H	-1.5952563950	-4.4287268643	0.8411687330
H	-3.6790692979	-0.0664554585	1.8362851383
H	-2.7907323448	-0.4456005296	0.3332108754
H	-4.3402565733	-1.2379610780	0.6785630440
H	-3.5837862520	-4.0985920064	3.5017927724
H	-4.2864423097	-2.4858231858	3.7653166712
H	-4.7647668092	-3.4389502124	2.3443558671
H	-1.7624292135	-4.9365928829	4.6552427885
H	-1.6685456454	-5.3557470377	2.9237189822
H	-0.3931255050	-5.8849921714	4.0403696882
H	2.1571292957	-4.2042446994	2.4802071978
H	0.7733641272	-4.7040070449	1.4758248325
H	1.3401601864	-3.0286479496	1.4303257559
H	0.1466162841	-3.0862490547	5.9862336771
H	1.3904829120	-4.2330156271	5.4432138677
H	1.6295477401	-2.4870298173	5.2062917419
H	-0.9498514458	2.1596657331	2.7124103695
H	-1.5100971434	-1.6225033978	4.0839084148

H	3.2205411394	-1.8722028776	2.2023602542
H	4.1493454865	-0.4502825700	6.0445876135
H	1.6117750307	0.4466726630	5.3542086433
H	1.1594183448	-1.0682961738	-0.0953074546

[GeP] MECP

P	1.0396346193	-0.2541586482	0.1626123073
Ge	-0.3831294538	0.1950671201	2.2932030678
Si	-1.0945137888	-3.0412371887	1.8783323493
Si	-2.8440592395	-1.1636160650	3.8405080325
Si	1.7078483792	0.0532936348	5.0062547392
Si	0.5755128563	2.8862284903	3.8822654310
C	-1.1350001449	-1.5503889745	3.0694603236
H	-0.4773591277	-1.8132621384	3.9155982646
C	0.9770492509	1.0377996809	3.5601259456
H	1.8120116296	1.1458035979	2.8448461117
C	0.6876774488	-3.6634022589	1.6507423132
H	1.0962430318	-4.0569977892	2.5943509405
H	1.3619102496	-2.8747816960	1.2850716811
H	0.7001589571	-4.4826949185	0.9136737008
C	-1.8099596385	-2.6298464223	0.1668163066
H	-1.1797748851	-1.9012316061	-0.3633869384
H	-2.8282306861	-2.2213336677	0.2236448290
H	-1.8444058545	-3.5469933362	-0.4431325124
C	-2.0863431935	-4.5112710562	2.5769349808
H	-1.9585794333	-5.3829473575	1.9146421723
H	-3.1633026843	-4.2983709931	2.6456258752
H	-1.7292218993	-4.7953066838	3.5788961500
C	-2.8743067617	0.5740067523	4.6198242885
H	-2.6616978524	1.3626209971	3.8818197061
H	-2.1394593420	0.6628247353	5.4314434178
H	-3.8715665842	0.7748180504	5.0433804872
C	-4.2147663593	-1.1986358249	2.5257219928
H	-5.1720772248	-0.8888531398	2.9742736348
H	-4.3539443671	-2.1994722891	2.0917811618
H	-3.9845981145	-0.4999343177	1.7058571206
C	-3.2496593247	-2.3851918809	5.2393852487
H	-4.2023961858	-2.0995993710	5.7142493878
H	-2.4693450290	-2.3591217402	6.0161414721
H	-3.3419731895	-3.4203719120	4.8845521299
C	3.1160864812	1.0299325695	5.8433384890
H	2.7617193395	1.9650009985	6.3016494464
H	3.9041982907	1.2849022529	5.1177770854
H	3.5758481052	0.4191645848	6.6373781429
C	2.4778034282	-1.5664880140	4.3760225003
H	2.8441828493	-2.1706165499	5.2216500008
H	3.3326660324	-1.3627374844	3.7141170453
H	1.7587963074	-2.1739523854	3.8111277338
C	0.4302782010	-0.3699770854	6.3493319255
H	0.9239740964	-0.9281830953	7.1609717297
H	-0.3851667861	-0.9953400287	5.9614975241
H	-0.0152055490	0.5349977873	6.7860972611
C	2.1788410995	3.8842321973	3.6522763758
H	2.0017893388	4.9558633438	3.8388143424
H	2.5355185041	3.7752112876	2.6169119203
H	2.9755936095	3.5456094510	4.3299476651
C	-0.6792416139	3.5653871152	2.6241047228
H	-1.6565447667	3.0642159155	2.6945322759
H	-0.3101907023	3.4442931107	1.5957209508
H	-0.8390221622	4.6407489883	2.8035402921
C	-0.1160836055	3.2169771918	5.6219842106
H	-0.3089014498	4.2963784239	5.7332064902
H	0.5850762126	2.9198467531	6.4154218515
H	-1.0663088108	2.6888622099	5.7877632693
C	1.3552710249	1.4910733878	-0.3582123155
C	0.3384397416	2.3098267911	-0.9639409658
C	0.5309086053	3.6976356760	-0.9896163932
H	-0.2502814146	4.3271480635	-1.4089857716
C	1.6892314206	4.3219497221	-0.4936824137
C	2.7361635325	3.4858737347	-0.0900518361
H	3.6766793231	3.9443109902	0.1948627668
C	2.6331825048	2.0804181820	-0.0527277419
C	-0.9193855283	1.7185668302	-1.6644823475
C	-1.6095676124	2.7620977632	-2.5759643295
H	-2.4312517347	2.2745585610	-3.1213870362
H	-0.9143120669	3.1837833063	-3.3173259395
H	-2.0502867731	3.5889686001	-2.0004359141
C	-0.4979291648	0.5471392488	-2.5852177705
H	-0.0766388617	-0.2965495272	-2.0169612596
H	0.2634263943	0.8739949852	-3.3090504242
H	-1.3682011207	0.1642841396	-3.1399306031
C	-1.9727979694	1.2492976084	-0.6397615627
H	-2.8510235085	0.8317648873	-1.1566121832
H	-2.3028742653	2.0882443265	-0.0103646078
H	-1.5793123845	0.4711552091	0.0282534265
C	1.7872278243	5.8572774684	-0.4709334698
C	0.6694797714	6.4155877409	0.4444325792
H	-0.3290088982	6.1122624958	0.0982547693
H	0.7033828028	7.5163650537	0.4618262605
H	0.7925763550	6.0487584476	1.4732771086
C	3.1442519808	6.3485289562	0.0712631921
H	3.1605664307	7.4486139674	0.0887536751
H	3.9813570274	6.0137355964	-0.5597816491
H	3.3207503389	5.9950519839	1.0980712867
C	1.6046949626	6.4066380190	-1.9077389221
H	0.6250018879	6.1345610622	-2.3258472228
H	2.3806705021	6.0079700587	-2.5787731337
H	1.6776312705	7.5056087931	-1.9101901927
C	3.9400760147	1.2598695919	0.1879252780
C	3.9408738307	0.4673627937	1.5147520558

H	4.8903386735	-0.0800762473	1.6246762708	H	3.9256634808	-0.1351831858	1.3172762492
H	3.1294119421	-0.2712674684	1.5498478020	H	2.2105452405	0.0364212662	1.7492472457
H	3.8363860165	1.1401669222	2.3780728448	H	2.9573122685	1.1987688302	0.6299365850
C	5.1907696734	2.1710891880	0.2314845305	C	3.7214738439	-0.5819666074	-1.3101980228
H	5.1787629722	2.8541999599	1.0941671788	H	3.8400952155	0.4643499109	-1.6289160102
H	5.3006734756	2.7668377794	-0.6864403668	H	3.5769399190	-1.2063495046	-2.2042425685
H	6.0864085100	1.5398990355	0.3278624964	H	4.6664370952	-0.8922442558	-0.8402962933
C	4.1381996711	0.2904650969	-1.0072955025	C	2.5666234272	-2.2529274124	0.1086930042
H	4.1656417278	0.8490747723	-1.9549813040	H	2.3421370018	-2.8806207134	-0.7669467840
H	3.3305866190	-0.4508503168	-1.0662959556	H	1.8182711124	-2.4764649264	0.8836642545
H	5.0897459676	-0.2527145577	-0.8969937577	H	3.5523749379	-2.5458141865	0.5027374431

Mes*P

P	-0.0382023802	-1.0012307434	1.6027068569
C	-0.0396469029	-0.4165400096	-0.1190131834
C	-1.2764417022	-0.0156391540	-0.7575735273
C	-1.2286061011	0.4279957599	-2.0847279991
H	-2.1537227666	0.7278943097	-2.5696604678
C	-0.0417884393	0.5091091893	-2.8332242308
C	1.1441833198	0.1175969251	-2.1983571238
H	2.0693260542	0.1739691353	-2.7612140245
C	1.1913334790	-0.3442207075	-0.8720435517
C	-2.6561455756	-0.0548251077	-0.0421537224
C	-3.8082930944	0.4248798413	-0.9568881023
H	-4.7536292431	0.3747572736	-0.3966419658
H	-3.9193386346	-0.2101591906	-1.8483635017
H	-3.6734150453	1.4670929010	-1.2825599515
C	-3.0069520619	-1.5065282106	0.3724592313
H	-2.2804068057	-1.9246396520	1.0850904288
H	-3.0286965068	-2.1638651287	-0.5099164388
H	-3.9977675737	-1.5350564987	0.8522952478
C	-2.6541249404	0.8884367341	1.1875686010
H	-3.6401163635	0.8746507855	1.6777824519
H	-2.4331097358	1.9215260235	0.8792788684
H	-1.9049929339	0.5936158006	1.9373179952
C	-0.0890900252	1.0086029588	-4.2879459908
C	-0.6783846080	2.4411786193	-4.3155764953
H	-1.6974890353	2.4693563483	-3.9044728541
H	-0.7220155823	2.8181058937	-5.3496027882
H	-0.0567010285	3.1295257009	-3.7232493889
C	1.3067660094	1.0458006865	-4.9416681054
H	1.2234857776	1.4126891824	-5.9757746454
H	1.7654557620	0.0462733579	-4.9790959431
H	1.9903185505	1.7193416652	-4.4030233927
C	-0.9911451573	0.0614682410	-5.1186904164
H	-2.0197410551	0.0358914699	-4.7311668073
H	-0.5979597105	-0.9662268262	-5.0990932154
H	-1.0337837925	0.3948101944	-6.1676764345
C	2.5727557752	-0.7544195720	-0.2876679877
C	2.9334485314	0.1391925888	0.9263274721

$Ge[CH(SiMe_3)_2]_2$

Ge	-0.0743093026	0.0914154325	0.1655057261
Si	-1.4109743460	2.9739812990	-0.2542065668
Si	1.1170434499	2.3400009940	-2.0912182298
Si	0.2863356616	-2.3469947846	-1.9593011833
Si	-2.7631770183	-1.4698085937	-1.3426720936
C	-2.9639077095	2.2344333929	0.5653407692
C	-2.0036908396	4.2922038010	-1.4903571866
C	-0.4456065690	3.7769172982	1.1738184895
C	1.8391605754	3.9365657926	-1.3512355757
C	2.6036215556	1.1565045617	-2.1899008264
C	0.5438058483	2.7174643932	-3.8658256129
C	2.0362947158	-2.2909108760	-1.2025107368
C	0.2297655663	-1.4886475497	-3.6522024495
C	-0.0565794391	-4.2033314977	-2.2023310844
C	-3.3018472587	-3.0755451206	-2.2186722222
C	-3.8821098520	-1.3373723437	0.1908828788
C	-3.1597222199	-0.0741446206	-2.5752930767
C	-0.3929728070	1.6192794898	-1.1508449919
C	-0.9502506426	-1.5283145663	-0.7520575325
H	-3.6270381614	1.7171808069	-0.1411829682
H	-2.6905079707	1.5212673396	1.3601474140
H	-3.5426871437	3.0429675112	1.0405753289
H	-1.1693220567	4.8428182645	-1.9483763470
H	-2.5875734851	3.8279337519	-2.3006736180
H	-2.6526975985	5.0224089055	-0.9803651168
H	0.0794305557	3.0127946482	1.7685775844
H	0.2959752065	4.5126461108	0.8379788447
H	-1.1529158081	4.2925596230	1.8429902639
H	1.1227773213	4.7694487553	-1.3274562157
H	2.2145666550	3.7751277393	-0.3297206933
H	2.6925514757	4.2466459471	-1.9759137624
H	3.0029798968	0.9321931451	-1.1880618268
H	2.3683340674	0.2071475145	-2.6876373765
H	3.4091580664	1.6438699895	-2.7628429134
H	0.1924240688	1.8078044215	-4.3768437357
H	-0.2811241784	3.4453596497	-3.8696451113
H	1.3736609211	3.1369838724	-4.4572509163

H	2.3607767097	-1.2899093355	-0.8866237661
H	2.0664768666	-2.9418237281	-0.3137390771
H	2.7808498670	-2.6696816487	-1.9211473908
H	-0.7088950088	-1.7323492705	-4.1732539912
H	0.2835397013	-0.3966199131	-3.5541120648
H	1.0641035769	-1.8175285801	-4.2914179916
H	-0.2204025894	-4.7034479519	-1.2347878272
H	-0.9273111997	-4.4012791262	-2.8403636596
H	0.8241937558	-4.6695322470	-2.6740565959
H	-2.8352532207	-3.1913013758	-3.2083105240
H	-3.0679009744	-3.9698229485	-1.6218914785
H	-4.3938999430	-3.0453253291	-2.3675682266
H	-3.8120291195	-2.2655980130	0.7808342656
H	-3.6057427685	-0.5013348257	0.8470144580
H	-4.9361326000	-1.2059022935	-0.1020314389
H	-3.1434975242	0.9247761371	-2.1212016973
H	-2.4614435744	-0.0723986891	-3.4255078041
H	-4.1724345489	-0.2409891881	-2.9768929144
H	-1.0343388638	1.2364070837	-1.9586213381
H	-0.9686097425	-2.2465332549	0.0944017339

[SnP] MeCP

P	0.1161486467	-0.1065891288	-0.0991472676
Sn	0.0242815092	-0.0337314529	2.2639461008
Si	-0.4415808824	-3.4301062857	2.3559186292
Si	-2.7811502619	-1.4358526872	3.4544558344
Si	1.7617636303	0.4665968590	5.1942108818
Si	0.2511382046	3.0462892593	3.8767606015
C	-0.9217572440	-1.7822691983	3.1998747235
H	-0.4745339049	-1.8085799937	4.2086162486
C	1.0552278278	1.3250671064	3.6500330136
H	1.9354147597	1.5550164555	3.0225542516
C	1.3538624733	-3.4487693891	1.7299733501
H	2.0792982369	-3.1865884118	2.5144603303
H	1.4898638127	-2.7508222738	0.8913584250
H	1.5985596996	-4.4630467354	1.3744229353
C	-1.5549769180	-3.7837021499	0.8620105791
H	-1.4966834860	-2.9570168220	0.1368409088
H	-2.6086967668	-3.9246576725	1.1441992945
H	-1.2158227499	-4.7010923009	0.3552359544
C	-0.5891921055	-4.8235884185	3.6436642744
H	-0.2720552800	-5.7812834735	3.2003292276
H	-1.6147165176	-4.9448469662	4.0189073014
H	0.0664138048	-4.6217829683	4.5062707446
C	-2.9826074340	0.0208142676	4.6629411089
H	-2.4885425415	0.9267602292	4.2803166087
H	-2.5558356345	-0.2012527880	5.6521576756
H	-4.0501699532	0.2569440259	4.7978676439
C	-3.6484296723	-0.9440912567	1.8412113622

H	-4.7302657092	-0.8413692759	2.0241238243
H	-3.5056117704	-1.6821003568	1.0403895808
H	-3.2783612607	0.0226324011	1.4692371285
C	-3.6681875110	-2.9401509255	4.2051075257
H	-4.7094023980	-2.6734656808	4.4479630974
H	-3.1772792468	-3.2704287875	5.1335564805
H	-3.6928866084	-3.7923797896	3.5099686242
C	2.9681016070	1.6582327736	6.0560133264
H	2.4550841889	2.5545313067	6.4346119733
H	3.7582114687	1.9873912949	5.3630441684
H	3.4522959148	1.1586617055	6.9104183557
C	2.7393013845	-1.0897329617	4.7101763021
H	3.2217453821	-1.5200890628	5.6023684724
H	3.5250549779	-0.8626141201	3.9749064286
H	2.0878707493	-1.8639004779	4.2795737636
C	0.4354816315	-0.0560624970	6.4478925027
H	0.9245832746	-0.5475897451	7.3042261859
H	-0.2790918436	-0.7708062240	6.0168013883
H	-0.1354543193	0.8003980811	6.8303368038
C	1.6627567419	4.3124006952	3.7768118310
H	1.2800530190	5.3409454255	3.8686206557
H	2.1633500228	4.2194478081	2.8014598594
H	2.4141058035	4.1553656703	4.5642433646
C	-0.9742180104	3.4253278814	2.4832441898
H	-1.8265612546	2.7281072798	2.4740816869
H	-0.4777641887	3.3676365002	1.5043951392
H	-1.3767248090	4.4447685883	2.5973804080
C	-0.6487306505	3.2549088396	5.5370529468
H	-1.0791983111	4.2685722415	5.5789449366
H	0.0307746315	3.1468309660	6.3951946865
H	-1.4733843689	2.5377213534	5.6589538119
C	0.9093205763	1.5597648871	-0.4871749807
C	0.0850939793	2.6317986129	-0.9851541157
C	0.5786607509	3.9429282234	-0.8871332676
H	-0.0499919689	4.7643196679	-1.2205336113
C	1.8447835381	4.2552471512	-0.3663809312
C	2.6940882855	3.1768733329	-0.0925204789
H	3.7193390152	3.3867984459	0.1980570634
C	2.2924088026	1.8289054741	-0.2151977701
C	-1.2832502040	2.4213263465	-1.7064552695
C	-1.7213481690	3.6934837747	-2.4738652394
H	-2.6298243372	3.4657230286	-3.0511622724
H	-0.9496513107	4.0334571115	-3.1808379348
H	-1.9690684148	4.5261979550	-1.7990485790
C	-1.1377695942	1.3064438306	-2.7736774752
H	-0.9210400469	0.3280186215	-2.3251187321
H	-0.3266469187	1.5504316546	-3.4763217262
H	-2.0740015356	1.2083077297	-3.3452623707
C	-2.4279624580	2.0820509887	-0.7230536402
H	-3.3748216379	1.9685469955	-1.2749229701

H	-2.5560535755	2.8866774028	0.0156082843
H	-2.2290927417	1.1489977906	-0.1847291751
C	2.2844385192	5.7263148344	-0.2542231810
C	1.2227215004	6.5328938057	0.5329365912
H	0.2432868958	6.5144956565	0.0340139259
H	1.5319007929	7.5861981802	0.6222747737
H	1.0912312236	6.1251227749	1.5443226085
C	3.6414959157	5.8799864340	0.4621647534
H	3.8929034322	6.9471279070	0.5570266592
H	4.4571149418	5.3988214871	-0.0979368298
H	3.6171477409	5.4496310925	1.4741395248
C	2.4166039382	6.3126564523	-1.6832219399
H	1.4619517304	6.2613886347	-2.2264782722
H	3.1654559347	5.7532408530	-2.2643346435
H	2.7291820649	7.3684026320	-1.6410320619
C	3.4225688884	0.7476817207	-0.1768401248
C	3.6279880549	0.1380332155	1.2271568138
H	4.4819430423	-0.5580608364	1.2200842845
H	2.7441899936	-0.4245983863	1.5490670163
H	3.8378483007	0.9228608411	1.9699718690
C	4.7804472042	1.3724615731	-0.5986143333
H	5.1839117831	2.0662077952	0.1530538786
H	4.6979608823	1.9093417339	-1.5552827537
H	5.5221686277	0.5690783696	-0.7211234154
C	3.1459056897	-0.3794902572	-1.2005580903
H	2.9978299482	0.0378976445	-2.2077783704
H	2.2545969980	-0.9671492545	-0.9438003019
H	4.0031541310	-1.0695426668	-1.2320933800

$Sn[CH(SiMe_3)_2]_2$

Sn	-0.3251683207	0.1516736734	0.3191572612
Si	-1.8196880074	3.0309574743	-0.4307602764
Si	0.8655943660	2.4561153239	-2.0588850561
Si	-0.0738493892	-2.3379579252	-1.9901687463
Si	-3.0888684455	-1.7105979490	-1.0009114230
C	-3.0946609161	2.2158738585	0.7401755073
C	-2.8268695003	4.0179994335	-1.7046930426
C	-0.8071131284	4.1967686553	0.6803113501
C	2.2769554387	2.5688066510	-0.7851047787
C	1.4612017637	1.4002360363	-3.5260238040
C	0.6149438154	4.1946150645	-2.8033793806
C	1.7331024126	-1.8838255316	-1.5516321522
C	-0.5298649919	-1.6329068486	-3.6915162213
C	-0.1052512716	-4.2378689040	-2.0775186352
C	-3.6299919643	-3.3687320476	-1.7699260726
C	-4.0298224893	-1.5516130883	0.6472405213
C	-3.6569039229	-0.3456034261	-2.1957182143
C	-0.7043438613	1.7286316332	-1.2647104746
C	-1.2292166220	-1.6853355774	-0.6343350017
H	-2.6154775996	1.6486665561	1.5575604122
H	-3.7795665867	1.5384828476	0.2116683492
H	-3.7046963070	2.9992125046	1.2189154354
H	-3.4153925174	3.3382242206	-2.3407770770
H	-2.1887521539	4.6277134163	-2.3587665084
H	-3.5310884554	4.6907057994	-1.1885480693
H	-0.0741385524	4.7987576163	0.1252980383
H	-0.2611846599	3.6211284848	1.4445802568
H	-1.4845982358	4.8896977947	1.2043257849
H	2.6016811979	1.5729706213	-0.4417916888
H	1.9766543088	3.1439467499	0.1034416598
H	3.1528302349	3.0658641910	-1.2319106348
H	0.7017521060	1.3751666569	-4.3230304081
H	1.6959690177	0.3635956530	-3.2531722619
H	2.3726590472	1.8529396129	-3.9491858009
H	0.3240727757	4.9521984302	-2.0622201353
H	-0.1533006188	4.1756905176	-3.5917981548
H	1.5595773254	4.5244576583	-3.2659465086
H	2.0329205974	-2.3849469888	-0.6170555343
H	1.8978029046	-0.8019050198	-1.4185510906
H	2.4253615041	-2.2134758925	-2.3431488926
H	-0.6628248498	-0.5438929348	-3.6510316591
H	-1.4684359092	-2.0781250160	-4.0551580160
H	0.2559377325	-1.8529663698	-4.4308175518
H	-1.0854829945	-4.6218980182	-2.3925453311
H	0.1314931128	-4.6757083577	-1.0946187044
H	0.6498092618	-4.5967403587	-2.7961330493
H	-3.3092454386	-4.2198061475	-1.1493903437
H	-3.2146998677	-3.5086052737	-2.7794643182
H	-4.7286796105	-3.4040078096	-1.8517554256
H	-3.7809147361	-0.6270569967	1.1887864790
H	-3.7820138462	-2.4009466155	1.3043327703
H	-5.1192774187	-1.5623820781	0.4826459548
H	-3.1452195445	-0.4241800393	-3.1655808354
H	-3.4754662734	0.6619709587	-1.8005836383
H	-4.7391484886	-0.4468193748	-2.3763882058
H	-1.2921894841	1.2490795694	-2.0648166652
H	-1.1092619435	-2.4029730735	0.2029200085

4. References

[41] CrysAlisPro, Agilent Technologies, Version 1.171.41.117a.

[42] (a) G. M. Sheldrick in SHELXL97, *Programs for Crystal Structure Analysis (Release 97-2)*, Institut für Anorganische Chemie der Universität, Tammanstrasse 4, D-3400 Göttingen, Germany, 1998; (b) G. M. Sheldrick, *Acta Crystallogr. Sect. A* **1990**, *46*, 467−473; (c) G. M. Sheldrick, *Acta Crystallogr. Sect. A* **2008**, *64*, 112−122.

[43] F. Neese, F. Wennmohs, U. Becker, C. Riplinger, *J. Chem. Phys.* **2020**, *152*, 224108.

[44] F. Neese, *Wiley Interdiscip. Rev.: Comput. Mol. Sci.* **2018**, *8*, 1–6.

[45] F. Neese, *Wiley Interdiscip. Rev.: Comput. Mol. Sci.* **2012**, *2*, 73–78.

[46] R. Izsák, F. Neese, *J. Chem. Phys.* **2011**, *135*, 144105.

[47] Neese, F. Wennmohs, A. Hansen, U. Becker, *Chem. Phys.* **2009**, *356*, 98–109.

[48] M. Bühl, C. Reimann, D. A. Pantazis, T. Bredow, F. Neese, *J. Chem. Theory Comput.* **2008**, *4*, 1449–1459.

[49] D. A. Pantazis, X. Y. Chen, C. R. Landis, F. Neese, *J. Chem. Theory Comput.* **2008**, *4*, 908–919.

[50] Y. S. Lin, G. De Li, S. P. Mao, J. Da Chai, *J. Chem. Theory Comput.* **2013**, *9*, 263–272.

[51] M. Bühl, C. Reimann, D. A. Pantazis, T. Bredow, F. Neese, *J. Chem. Theory Comput.* **2008**, *4*, 1449–1459.

[52] D. A. Pantazis, X. Y. Chen, C. R. Landis, F. Neese, *J. Chem. Theory Comput.* **2008**, *4*, 908–919.

[53] J. Baker, *J. Comput. Chem.* **1986**, *7*, 385–395.

[54] N. Harvey, M. Aschi, H. Schwarz, W. Koch, *Theor. Chem. Acc.* **1998**, *99*, 95–99.

[55] E. D. Glendening, J. K. Badenhoop, A. E. Reed, J. E. Carpenter, J. A. Bohmann, C. M. Morales, P. Karafiloglou, C. R. Landis, F. Weinhold, *NBO 7.0*, 2018.
